# Metal-organic framework boosts heterogeneous electron donor–acceptor catalysis

**DOI:** 10.1038/s41467-023-43577-5

**Published:** 2023-11-27

**Authors:** Jiaxin Lin, Jing Ouyang, Tianyu Liu, Fengxing Li, Herman Ho-Yung Sung, Ian Williams, Yangjian Quan

**Affiliations:** Department of Chemistry and the Hong Kong Branch of Chinese National Engineering Research Centre for Tissue Restoration & Reconstruction, The Hong Kong University of Science and Technology (HKUST), Kowloon, Hong Kong SAR China

**Keywords:** Heterogeneous catalysis, Inorganic chemistry

## Abstract

Metal-organic framework (MOF) is a class of porous materials providing an excellent platform for engineering heterogeneous catalysis. We herein report the design of MOF Zr-PZDB consisting of Zr_6_-clusters and PZDB (PZDB = 4,4’-(phenazine-5,10-diyl)dibenzoate) linkers, which served as the heterogeneous donor catalyst for enhanced electron donor–acceptor (EDA) photoactivation. The high local concentration of dihydrophenazine active centers in Zr-PZDB can promote the EDA interaction, therefore resulting in superior catalytic performance over homogeneous counterparts. The crowded environment of Zr-PZDB can protect the dihydrophenazine active center from being attacked by radical species. Zr-PZDB efficiently catalyzes the Minisci-type reaction of *N*-heterocycles with a series of C-H coupling partners, including ethers, alcohols, non-activated alkanes, amides, and aldehydes. Zr-PZDB also enables the coupling reaction of aryl sulfonium salts with heterocycles. The catalytic activity of Zr-PZDB extends to late-stage functionalization of bioactive and drug molecules, including Nikethamide, Admiral, and Myristyl Nicotinate. Systematical spectroscopy study and analysis support the EDA interaction between Zr-PZDB and pyridinium salt or aryl sulfonium salt, respectively. Photoactivation of the MOF-based EDA adduct triggers an intra-complex single electron transfer from donor to acceptor, giving open-shell radical species for cross-coupling reactions. This research represents the first example of MOF-enabled heterogeneous EDA photoactivation.

## Abstract (web summary)

Heterogeneous electron donor–acceptor (EDA) catalysis has seen limited progress to date. Here, the authors showcase a significant improvement in EDA interaction by employing a metal-organic framework (MOF) as the donor catalyst, underscoring the potential of MOFs in catalytic processes.

## Introduction

**Fig. 1:**
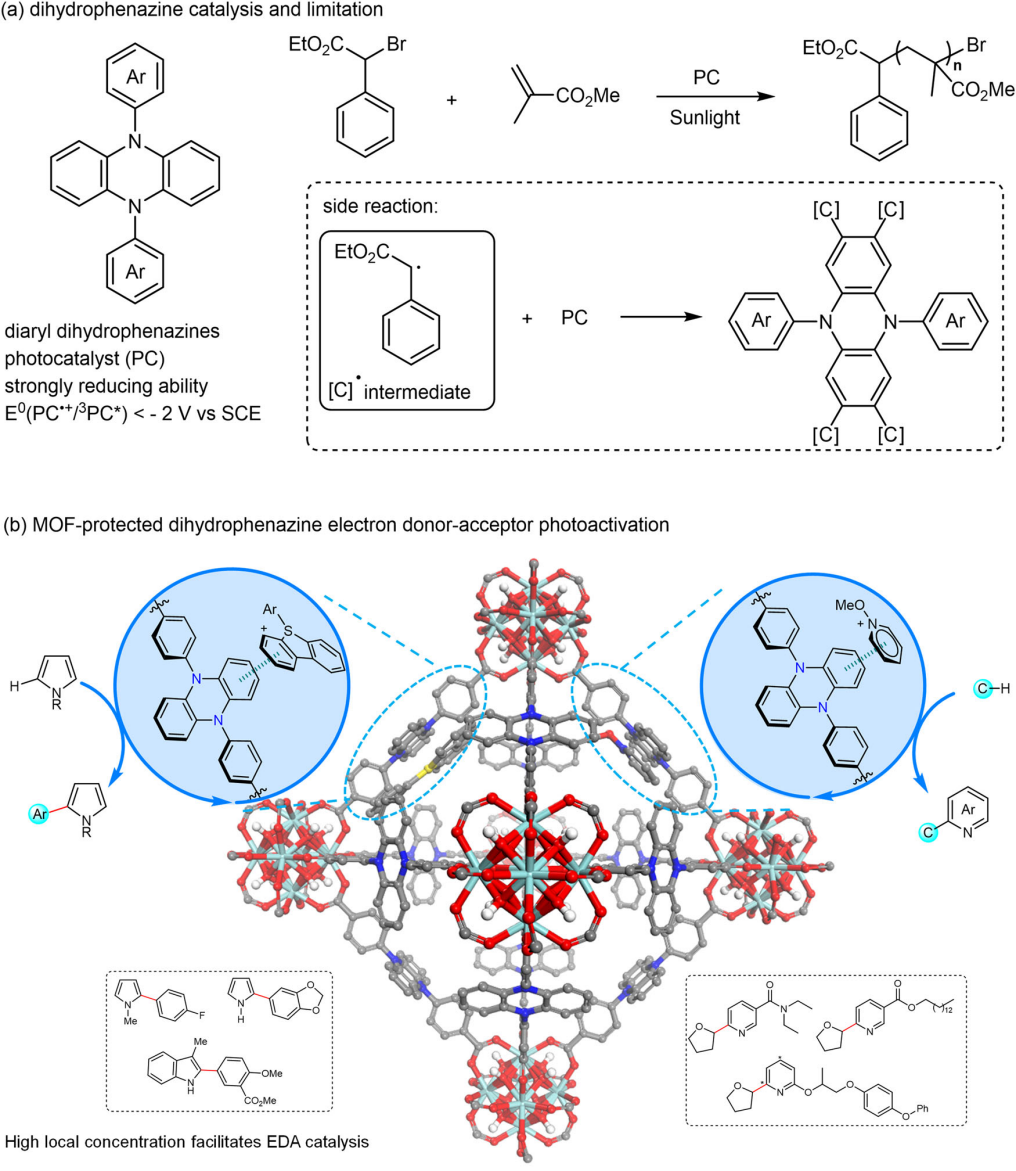
Diaryl dihydrophenazine catalysis. **a** Representative example of visible-light dihydrophenazine catalysis and its limitation. Ar aromatics, SCE saturated calomel electrode. **b** Enhancing the performance of dihydrophenazine EDA photoactivation by MOF platform. EDA electron donor–acceptor, MOF metal-organic framework; light blue: Zr, red: O, blue: N, gray: C.

Organic photosensitizers, a representative class of non-metal photocatalysts, have received increasing research interest^1–3^. In fact, nature evolves pigments to utilize solar energy for important chemical transformations^4^. Compared with inorganic photocatalysts (PCs), the organic ones possess a relatively broader redox window, and thus a stronger capability to active inert molecules^3^. However, their disadvantages of a shorter excited-state lifetime and lower stability lead to an inferior catalytic performance in some cases. For example, diaryl dihydrophenazines are visible-light PCs with strongly reducing ability (E^0^(PC^•+^/^3^PC^*^) < − 2 V versus saturated calomel electrode) upon excitation. Although they efficiently catalyzed the atom transfer radical polymerization^5^, the carbon radical intermediate was recently found to attack the PCs (Fig. 1a)^6,7^. The susceptibility of dihydrophenazine PCs to the reaction with open-shell radical species may restrict their applications in photoredox catalysis.

In addition to traditional photoredox catalysis enabled by photosensitizers, the recently developed electron donor–acceptor (EDA) photoactivation provides an alternative mode for photochemical synthesis^8,9^. The proper electron donor catalyst can interact with electron acceptor substrates to form a photoactive EDA complex. Upon light irradiation, intra-complex single electron transfer (SET) from donor to acceptor occurs to give active open-shell species for further transformations^10–13^. In most cases, the EDA interaction is “weak”, thus a relatively high concentration is preferred to promote the chances of generating EDA adducts. Despite fruitful achievements, the development of the corresponding heterogeneous version of EDA photoactivation remains inaccessible.

Metal-organic frameworks (MOFs)^14–26^, a class of 3D porous materials by bridging metal-containing second building units (SBUs) with organic linkers, have proved their superiority in engineering heterogeneous catalysis due to the wide synthetic tunability and unique porous structure^27–43^. Specific to MOF-based photocatalysis, factors including site isolation effect, confinement effect, high local concentration, and active-center protection endow MOF catalysts with superior catalytic performance compared with homogeneous counterparts^44–58^. A variety of metal-based PCs have been integrated into MOFs for photoinduced synthesis, while the merger of organic PCs remains less investigated because of their typically low symmetry and high steric demand^59–62^. Among the aforementioned factors, a high local concentration of active centers in MOF was surmised to promote the EDA interaction and related EDA photoactivation, which remains elusive in heterogeneous catalysis. Moreover, integrating diaryl dihydrophenazine into MOFs would reduce the risk of being attacked by radical intermediates and therefore expand their catalytic applications. Herein, we report the design of photoactive MOF Zr-PZDB (PZDB = 4,4’-(phenazine-5,10-diyl)dibenzoate), consisting of Zr_6_-SBUs and PZDB connecting ligands (Fig. 1b). Upon visible light irradiation, Zr-PZDB competently catalyzed the Minisci-type cross-coupling of *N*-heterocycles with ethers, alcohols, non-activated alkanes, amides, and aldehydes. Zr-PZDB also enabled the coupling reaction of aryl sulfonium salts with heterocycles. Furthermore, the late-stage functionalization of complex drug or bioactive molecules was attained by using Zr-PZDB as the catalyst^63^. In contrast, the homogeneous counterparts PZDB-H or PZDB-Me exhibited inferior catalytic efficiency. Our systematical spectroscopy study and analysis revealed the enhanced EDA interaction between Zr-PZDB and pyridinium salt or sulfonium salt. The subsequent photoinduced intra-complex SET allowed the generation of radicals and enabled the corresponding coupling reactions.

## Results and discussion

**Fig. 2:**
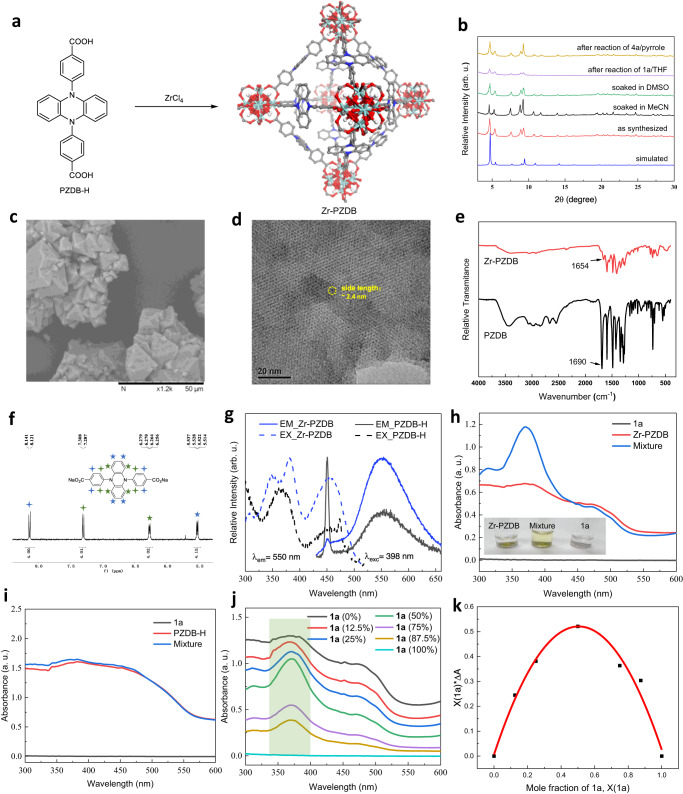
Synthesis and characterization of Zr-PZDB. **a** Schematic showing the synthesis of Zr-PZDB and the structure: X-ray single-crystal structure represented by an octahedron-like cage; disorder and H atoms are omitted for clarity; light blue: Zr, red: O, blue: N, gray: C. **b** PXRD patterns of Zr-PZDB; arb. u. = arbitrary units. **c**, **d** SEM (**c**) and HRTEM (**d**) of Zr-PZDB. **e** IR spectra of Zr-PZDB and PZDB-H. **f** Zoom-in ^1^H NMR of digested Zr-PZDB in DMSO-d_6_. **g** Normalized excitation and emission spectra of Zr-PZDB and PZDB-H in MeCN; arb. u. = arbitrary units. **h** UV-vis spectra of **1a**, Zr-PZDB, and their mixture in MeCN (2.5 × 10^−3 ^M); a. u. = absorbance units. **i** UV-vis spectra of **1a**, PZDB-H, and their mixture in MeCN (2.5 × 10^−3 ^M); a. u. = absorbance units. **j** UV-vis spectra of mixtures of **1a** and Zr-PZDB with different ratios of **1a** in MeCN (5 × 10^−3 ^M); a. u. = absorbance units. **k** Job’s plot based on UV-vis data in (**j**).

The PZDB-H linker was synthesized according to the reported procedure (see Supplementary Information for details)^64^. PZDB-H presents a rigid single-crystal structure (Supplementary Fig. S3), suggesting its potential to be bridging ligands for MOF synthesis. Solvothermal reaction of PZDB-H ligand with ZrCl_4_ in *N*,*N*-dimethylformamide (DMF) using trifluoroacetic acid as the modulator delivers octahedron single crystals of Zr-PZDB (Fig. 2a and Supplementary Fig. S4a; during the preparation of this manuscript, the Queen group reported a convenient synthesis of a series of MOFs with piperazine core^65^). The single-crystal structure suggests a formula of Zr_6_(μ_3_-O)_4_(μ_3_-OH)_4_(PZDB)_6_ for Zr-PZDB, which was further verified by TGA (thermogravimetric analysis, Supplementary Fig. S8) result with the residue weight of 23.7 wt% for ZrO_2_ close to the theoretical value of 23.1 wt% from the formula. Zr-PZDB exhibits a space group of Fm—3m (No. 225; see Supplementary Table S2) and fcu topology, wherein Zr_6_O_4_(OH)_4_ clusters are connected by 12 PZDB linkers in the face-centered-cubic array, in line with the iso-reticular structures of UiO-68^66^. The dihydrophenazine moiety in PZDB of Zr-PZDB retains the planar geometry. However, it is disordered due to the rotation around the PZDB backbone (Supplementary Fig. S4b). The shortest distance between two adjacent linkers is measured as ~2 Å, indicating the relatively steric demanding environment (Supplementary Fig. S5), which might account for the excellent protection of PZDB active centers by Zr-PZDB (vide infra).

Powder X-ray diffraction (PXRD) analyses indicated the high phase purity of Zr-PZDB. The corresponding PXRD pattern matches well with the simulated one from the single crystal structure (Fig. 2b). In addition, Zr-PZDB retained the crystalline structure after being soaked in a series of solvents, as evidenced by PXRD analyses (Fig. 2b and Supplementary Fig. S10). Scanning electron microscopy (SEM) imaging showed the octahedron morphology of Zr-PZDB (Fig. 2c). High-resolution transmission electron microscopy (HRTEM) imaging of Zr-PZDB revealed a distance of ~2.4 nm between two Zr_6_-SBUs (Fig. 2d), matching well with the distance of ~2.3 nm in the single crystal structure (Supplementary Fig. S6). IR spectra of PZDB-H and Zr-PZDB were collected. A stretching band at 1690 cm^−1^ assignable to the carbonyl group was observed for PZDB-H, while the carbonyl groups in Zr-PZDB showed the stretching band at 1654 cm^−1^ (Fig. 2e). The difference originated from the bonding of carboxylic group with metals. Only one set of signals assigned to PZDB-H was detected by ^1^H NMR analysis of the digested Zr-PZDB, indicating the inertness of the bridging ligand under MOF preparation conditions and the high purity of MOF (Fig. 2f). The porosity of Zr-PZDB was investigated by gas absorption analyses (Supplementary Fig. S9). The Brunauer–Emmett–Teller surface area of Zr-PZDB was measured as 715 m^2^/g with the calculated main pore window of ~1.3 nm. As shown in Fig. 2g, upon excitation at 398 nm, PZDB-H exhibited two characteristic emission peaks at 450 and 550 nm, respectively. However, the intensity of the 450 nm peak was significantly decreased, with a main emission peak at 550 nm for Zr-PZDB. Meanwhile, PZDB-H and Zr-PZDB showed similar excitation signals for the emission at 550 nm.

Functionalization of *N*-heterocycles is of great importance from their potential bioactivity viewpoints^67^. In addition to traditional synthetic routes, photoinduced derivatization via the intermediacy of open-shell radicals represents an alternative strategy. Taking this into consideration, the Minisci-type cross-coupling of pyridinium salt^68–70^ with tetrahydrofuran was chosen as the model reaction to evaluate the catalytic performance of Zr-PZDB. As shown in Table 1, Zr-PZDB competently catalyzed the cross-coupling reaction upon blue LED irradiation (Kessil PR160L-427, 390–470 nm, Supplementary Fig. S30) to give the target product **3a** in 80% isolated yield. Screening other solvents proved that CH_3_CN was the optimal choice (entries 2–4, Table 1). Base NaHCO_3_ outperformed other inorganic bases, due probably to its relatively better solubility (entries 5,6, and 8, Table 1). The use of NEt_3_ led to a reduced yield of 12% for **3a** (entries 5-8, Table 1), because of its potential to quench methoxy radical via hydrogen atom transfer reaction. The linker PZDB-H or PZDB-Me instead of Zr-PZDB as the catalyst delivered **3a** in only 6% and 13% yields, respectively (entries 9 and 10, Table 1). Loading PZDB-H into activated carbons as the catalyst provided a yield of 15% for **3a** (entry 17, Table 1 and Supplementary Fig. S36). These results indicated the important role of the MOF platform in the catalytic performance. Noteworthily, the use of several representative molecular photosensitizers, including Ir(ppy)_3_, Ru(bpy)_3_^2+^, and Eosin Y, afforded inferior results (entries 11–13, Table 1). The control experiments suggested that both light and MOF catalyst were indispensable for the efficient transformation (entries 14 and 15, Table 1). A combination of ZrCl_4_ and PZDB-H instead of Zr-PZDB led to trace amounts of **3a** (entry 16, Table 1).

**Table 1:** Optimization of reaction conditions^a^


Entry	Variations from “standard conditions”	Yield (%)
1	No variation	80
2	DCE instead of CH_3_CN	66
3	DCM instead of CH_3_CN	42
4	Toluene instead of CH_3_CN	56
5	Na_2_CO_3_ instead of NaHCO_3_	11
6	K_2_CO_3_ instead of NaHCO_3_	35
7	Et_3_N instead of NaHCO_3_	12
8	Cs_2_CO_3_ instead of NaHCO_3_	21
9	PZDB-Me instead of Zr-PZDB	13
10	PZDB-H instead of Zr-PZDB	6
11	Ir(ppy)_3_ instead of Zr-PZDB	8
12	Ru(bpy)_3_^2+^ instead of Zr-PZDB	41
13	Eosin Y instead of Zr-PZDB	70
14	Without light	Trace
15	Without Zr-PZDB	Trace
16	ZrCl_4_ and PZDB-H instead of Zr-PZDB	Trace
17	PZDB-H@C instead of Zr-PZDB	15

^a^Standard conditions: pyridinium salt (0.05 mmol), NaHCO_3_ (0.1 mmol), Zr-PZDB (2.5 μmol, 5 mol% based on the linker), THF (3.8 mmol, 0.3 mL), CH_3_CN (0.5 mL), N_2_, r.t., PR160L-427 (390–470 nm), 24 h; red: the newly formed bond; yields of isolated **3a**; ppy = 2-phenylpyridine, bpy = 2,2’-bipyridine, @C = loaded into activated carbons.

The individual solution of **1a** and suspension of Zr-PZDB were almost colorless; however, their mixture exhibited a light-yellow color (Fig. 2h). This phenomenon suggested the potential involvement of EDA interaction. Noteworthily, the Lakhdar lab pioneered the identification of an EDA interaction between the organic dye Eosin Y and *N*-ethoxy-2-methylpyridinium tetrafluoroborate^71^. To further verify the feasibility, a detailed spectroscopy study was then carried out. UV-vis spectrum of the mixture presented a significantly enhanced peak from 330 to 430 nm with respect to the absorption profiles of the individual **1a** and Zr-PZDB (Fig. 2h), indicating the formation of an EDA complex. In sharp contrast, the UV-vis spectrum of a mixture of **1a** and PZDB-H was almost identical to the sum of individual spectra of **1a** and PZDB-H (Fig. 2i). The obvious difference might be attributed to the high local concentration of dihydrophenazine active centers in Zr-PZDB, which are believed to benefit the formation of EDA adducts kinetically. UV-vis spectra of a series of mixtures with different Zr-PZDB/**1a** ratios were subsequently collected (the ratio of Zr-PZDB is based on the PZDB linker, Fig. 2j). The maximum absorption enhancement was observed for a Zr-PZDB/**1a** ratio of 1/1 (green line in Fig. 2j). The corresponding Job plot further verified the optimal ratio of 1/1 for the plausible EDA complex (Fig. 2k and Supplementary Table S3). In addition, no absorption enhancement was detected by mixing **1a** with NaHCO_3_ (Supplementary Fig. S13). To further illustrate the role of EDA interaction in this photoinduced Minisci-type transformation, a 420 nm band filter, which blocks lights with a wavelength smaller than 420 nm, was used with a Kessil PR160L-427 lamp (390-470 nm). No product **3a** was detected with **1a** being intact (Supplementary Figs. S32 and S33). Furthermore, the utilization of the Kessil PR160L-390 lamp (370-420 nm) provided a yield of ~50% for **3a** (Supplementary Fig. S32), with the detection of ~50% 4-methylpyrdine (Supplementary Fig. S34). The addition of a 380 nm band filter increased the yield to ~80% (Supplementary Figs. S32 and S35). These results indicate that (1) light with a wavelength larger than 420 nm cannot induce the reaction; (2) light less than 380 nm in wavelength might decrease the selectivity for **3a**; (3) the likely involvement of EDA interaction in this photoinduced transformation^72^. To demonstrate the considerable interaction between Zr-PZDB and pyridinium salt **1a**, an adsorption control experiment was designed and performed (Supplementary Fig. S38). After being stirred with 1 equiv of Zr-PZDB overnight, the CH_3_CN solution of **1a** only retained 5% of pyridinium salt. In other words, Zr-PZDB competently adsorbed 95% of **1a**, indicating their strong interaction.

**Fig. 3:**
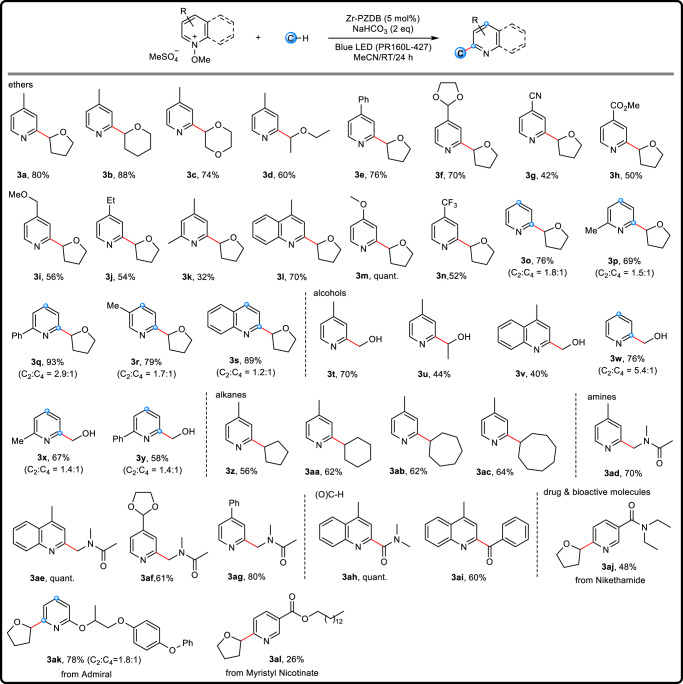
Zr-PZDB catalyzed Minisci-type cross-coupling reactions. Reactions were conducted at 0.05 mmol scale; yields of isolated products; red: the newly formed bond; blue: the reaction sites.

The scope of dehydrogenative cross-coupling was then explored, and the results were compiled in Fig. 3. With Zr-PZDB as the catalyst, five different kinds of C-H coupling partners, including ethers, alcohols, unactivated alkyls, amides, and aldehydes, reacted with pyridinium salts efficiently, evidencing the versatility of MOF catalyst. Both cyclic and linear ethers worked well to give products **3a**-**3d** in moderate to very good yields. Pyridinium salts bearing various substituents such as alkyl, phenyl, ester, cyanide, methoxy, and trifluoromethyl groups were compatible (**3e**-**3k** and **3m**-**3n**), and no obvious electronic effects were observed. The substrate 1-methoxylepidinium methyl sulfate afforded the corresponding **3** **l** in 70% yield. Pyridinium salts without a substituent on the C_4_ position delivered *ortho*- and *para*-functionalized products with C_2_ to C_4_ ratios of 1.2 to 2.9 (**3o**-**3s**). Noteworthily, relatively bulky substituents on the C_3_ position sterically protect the adjacent C_2_ and C_4_ positions, resulting in very good C_6_ selectivity (**3aj** and **3al**). Methanol and ethanol also served as good coupling partners, and the corresponding pyridinyl alcohols **3t**-**3v** were obtained. Without C_4_ protection, both *ortho*- and *para*-substituted products were isolated with ratios from 1.4 to 5.4 (**3w**-**3y**).

Direct replacement of ether by amine, for example, triethylamine, did not yield the coupling product. The easy oxidation of the corresponding α-C radical of triethylamine might account for the failed attempt. However, the dimethylacetamide (DMA) was tolerated to produce **3ad**-**3ag** efficiently. Furthermore, the non-activated, cyclic alkanes worked as appropriate feedstocks, giving the resultant **3z**-**3ac** in good yields. The ring size had little effect on the reaction efficiency. In addition to nucleophilic C(sp^3^)-H coupling partners, substrates containing carbonyl C(sp^2^)-H also underwent the cross-coupling reaction. Treatment of dimethylformamide (DMF) or benzaldehyde with 1-methoxylepidinium methyl sulfate under standard reaction conditions afforded **3ad** and **3ae**, respectively. No target product was observed when **1a** was used as the substrate. Noteworthily, Zr-PZDB effectively catalyzed the late-stage functionalization of complicated drug or bioactive molecules containing Nikethamide (**3aj**), Admiral (**3ak**), and Myristyl Nicotinate (**3al**).

**Fig. 4:**
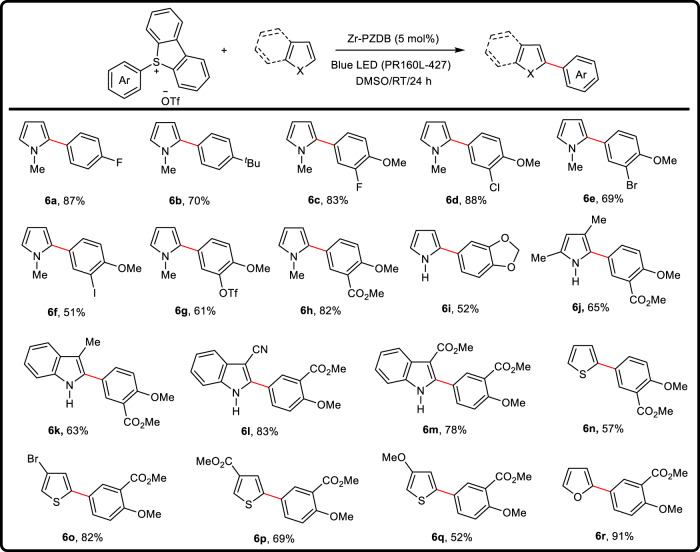
Zr-PZDB catalyzed cross-coupling of aryl sulfonium salts and heterocycles. Reactions were conducted at 0.2 mmol scale; yields of isolated products; red: the newly formed bond.

To further evaluate the ability of Zr-PZDB donor catalyst, aryl sulfonium salt was examined as the acceptor and substrate (Fig. 4). Upon getting one electron, aryl sulfonium salt would undergo decomposition to generate aryl radical, which is more reactive and sometimes incompatible with homogenous catalytic systems^13,73^. To our delight, Zr-PZDB effectively catalyzed the coupling reaction between aryl sulfonium salt **4a** and 1-methylpyrrole upon light (PR160L-427) irradiation, giving **6a** in 87% isolated yield (entry 1 in Supplementary Table S4). Replacement of Zr-PZDB by PZDB-H or PZDB-Me led to decreased yields of 39% and 12% for **6a**, respectively (entries 2 and 3 in Supplementary Table S4). PZDB-H@C as the catalyst provided a reduced yield of 36% (entry 7 in Supplementary Table S4 and Supplementary Fig. S37). The combination of ZrCl_4_ and PZDB-H instead of Zr-PZDB afforded **6a** in 45% yield (entry 4 in Supplementary Table S4). The absence of Zr-PZDB or light resulted in almost no target product (entries 5 and 6 in Supplementary Table S4), suggesting their importance in the coupling reaction.

A series of aryl sulfonium salts and heterocycles were then evaluated (Fig. 4). Functional groups, including alkyls, ethers, esters, halides, and triflate, were well tolerated, among which esters, halides, and triflate could be conveniently transformed to other substituents (**6a**-**6h**). Pyrrole, indole, thiophene, and furan underwent the coupling reaction smoothly to deliver **6i**-**6r** in 52-91% isolated yields. No obvious electronic effects were observed. In view of the prevalence of heterocycle derivatives in drug or bioactive molecules, this methodology might find potential applications in biochemistry.

**Fig. 5:**
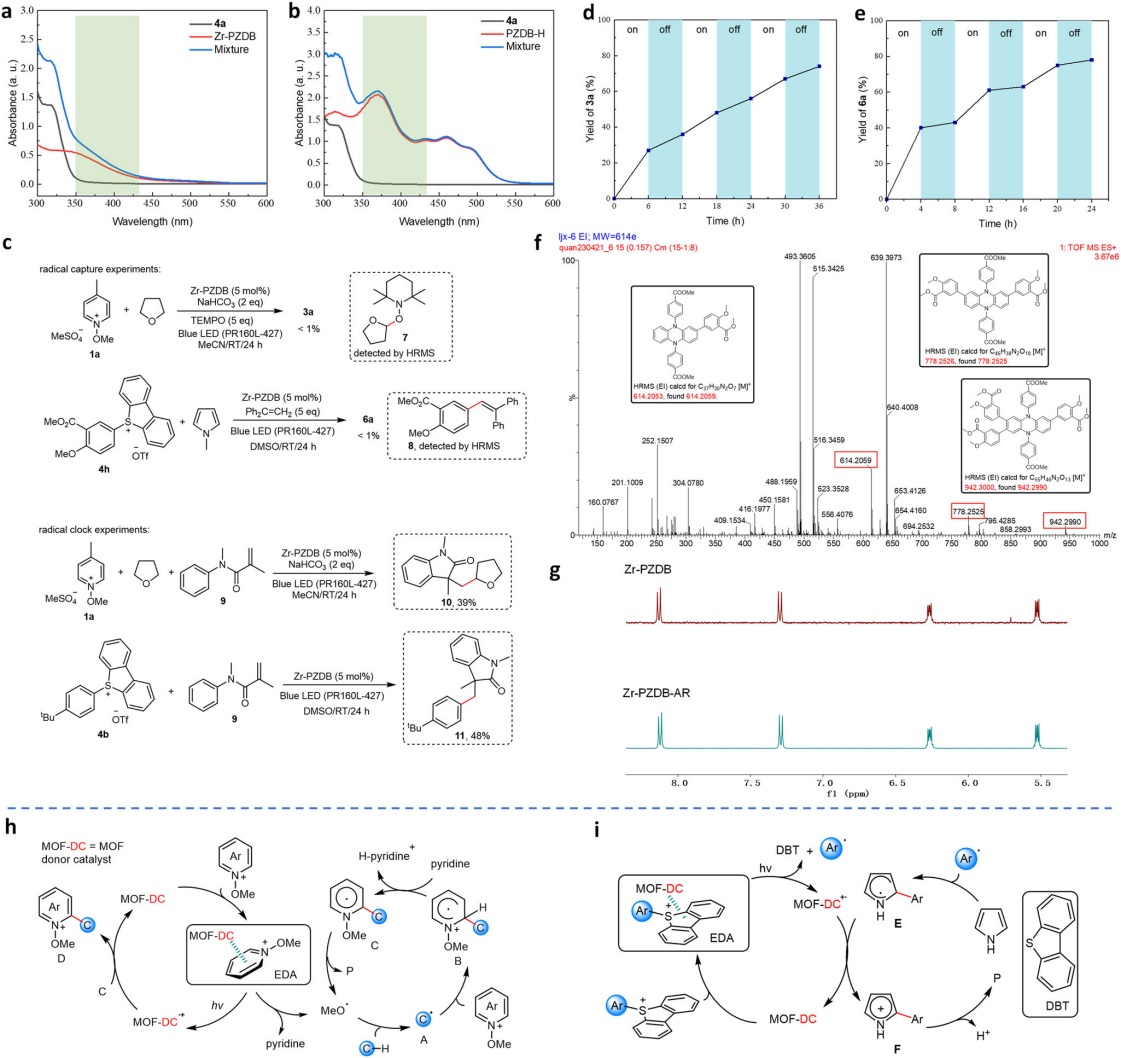
Mechanistic investigation. **a** UV-vis spectra of **4a**, Zr-PZDB, and their mixture in DMSO (5 × 10^−4 ^M); a. u. = absorbance units. **b** UV-vis spectra of **4a**, PZDB-H, and their mixture in DMSO (5 × 10^−4 ^M); a. u. = absorbance units. **c** Radical capture and clock experiments. **d**, **e** Light on/off experiments for coupling reactions of **1a**/THF (**d**) and **4a**/pyrrole (**e**). **f** HRMS spectrum of crude reaction mixture catalyzed by PZDB-Me. **g** Zoom-in ^1^H NMR of digested Zr-PZDB as prepared (up) and after catalytic reaction (down). **h**, **i** Proposed reaction mechanisms for Minisci-type reaction (**h**) and pyrrole arylation (**i**).

Investigation on the EDA interaction between Zr-PZDB and **4a** was also performed. UV-vis spectrum of the corresponding mixture showed the absorption enhancement from 350 nm to 430 nm compared with the absorption profiles of the individual **4a** and Zr-PZDB (Fig. 5a). In contrast, no obvious absorption enhancement was observed from the UV-vis spectrum of the mixture of PZDB-H and **4a** (Fig. 5b). Moreover, at a high concentration (2.5 × 10^−3 ^M), absorption enhancement was more obvious for the mixture of Zr-PZDB/**4a** with little changes for the mixture of PZDB-H/**4a** (Supplementary Fig. S12). These results further evidenced the enhancement effect of the MOF platform on the EDA interaction. To verify the involvement of radical species, radical capture and clock experiments were carried out (Fig. 5c). The addition of a radical scavenger, TEMPO, to the reaction mixture of **1a**/THF completely inhibited the formation of product **3a**, with the detection of radical capture species **7** by high-resolution mass spectrometry (HRMS). On the other hand, radical scavenger 1,1-diphenylethene successfully captured the more reactive aryl radical as proved by the formation of **8**. In addition, the results of radical clock experiments further verified the involvement of alkyl and aryl radicals in the coupling reactions, respectively.

Light on/off experiments for the coupling between **1a** and THF indicated the continuation of the reaction in the dark (Fig. 5d), suggesting a radical chain pathway. In sharp contrast, almost no reaction was detected for coupling between **4a** and pyrrole in the dark (Fig. 5e). In addition, its corresponding quantum yield was measured as 0.003 (Supplementary Information “Determination of quantum yield” section), negating the radical progression pathway^74^. A similar phenomenon was observed in hot-filtration control experiments. The removal of the Zr-PZDB catalyst from the reaction mixture of **1a** and THF after 12-hour irradiation did not totally shut down the reaction. The yield for **3a** was increased from 36% to 52% without MOF catalyst under light irradiation (Supplementary Fig. S26), albeit a higher yield of 80% in the presence of MOF catalyst. However, little yield increase for **6a** was observed after removing the Zr-PZDB catalyst from the reaction mixture of **4a** and pyrrole (Supplementary Fig. S27). In addition, the leaching of Zr was detected as <0.3% for both coupling reactions by ICP-MS, demonstrating the stability of Zr-PZDB under the coupling reaction conditions. These results combined with the light on/off experiments indicated: (1) the coupling reaction between **1a** and THF would involve the radical chain pathway, whereas the reaction of **4a** and pyrrole did not undergo radical progression; and (2) the heterogeneous nature of Zr-PZDB catalysis in both coupling reactions.

The Zr-PZDB catalyst was recovered and used in three runs of cross-coupling reactions of **1a**/THF and **4a**/pyrrole with slightly decreased catalytic performance (Supplementary Figs. S16 and S17). Zr-PZDB retained the crystalline structure, as evidenced by the matching PXRD pattern after the reaction (Fig. 2b). To compare the stability of PZDB active center in Zr-PZDB and homogeneous counterpart, attempts to recover and characterize the catalysts after the reaction were carried out. ^1^H NMR spectrum of the digested Zr-PZDB-AR (AR = after reaction) was almost identical to that of the as-prepared Zr-PZDB (Fig. 5g and Supplementary Fig. S18). In contrast, thin layer chromatography (TLC) and ^1^H NMR analyses of the homogeneous reaction mixture indicated the disappearance of the PZDB-Me catalyst (Supplementary Figs. S19 and S20). HRMS analysis further suggested the formation of mono-, di-, and tri-substituted derivatives of PZDB-Me probably originating from the attack of the corresponding aryl radicals (Fig. 5f and Supplementary Fig. S21). These results verified the reported susceptibility of dihydrophenazine molecular catalyst against active radical species and highlighted the protection ability of the MOF platform.

On the basis of the above experimental results and seminal literature precedents^75–80^, plausible reaction mechanisms are proposed (Fig. 5h, i). For Minisci-type reaction, integration of Zr-PZDB with pyridinium salt gives an EDA complex. Upon visible light irradiation, the intra-complex SET from Zr-PZDB to the pyridinium salt occurs to afford neutral pyridine and methoxy radical. The latter competently abstracts hydrogen from C-H bonds to generate carbon-centered radicals, which then attack the pyridinium salt to give the radical adduct intermediate **B**. Deprotonation of **B** by neutral pyridine or NaHCO_3_ delivers the intermediate **C**. The intermediate **C** then undergoes decomposition to form the final product and methoxy radical. The oxygen radical can further go through HAT with C-H coupling partners to start another cycle. Alternatively, intermediate **C** may reduce the MOF radical cation to regenerate the MOF catalyst and afford the substituted pyridinium salt **D**, which then forms an EDA adduct with Zr-PZDB followed by light excitation and decomposition to give the final product. For coupling reaction between aryl sulfonium salt and pyrrole, Zr-PZDB interacts with sulfonium salt to form the EDA adduct, which undergoes SET upon light irradiation to give MOF radical cation and aryl radical. The latter attacked pyrrole to deliver intermediate **E**, which is then oxidized by MOF radical cation to regenerate the Zr-PZDB catalyst and give intermediate **F**. Deprotonation of **F** finally delivers the coupling products.

In summary, we have integrated dihydrophenazine into MOF to realize a series of cross-coupling reactions. The Zr-PZDB MOF served as the heterogeneous donor catalyst to interact with the pyridinium and sulfonium acceptors for generating the photoactive EDA complexes, respectively. Photoactivation of the EDA adducts triggered the intra-complex SET and the coupling reactions. The high local concentration of PZDB active centers in Zr-PZDB is believed to promote the EDA interaction kinetically, accounting for the superior catalytic performance of Zr-PZDB over homogeneous counterparts. Zr-PZDB competently catalyzed the dehydrocoupling between pyridinium salts and ethers, alcohols, non-activated alkanes, amides, and aldehydes, leading to a wide variety of *N*-heteroarene derivatives. In addition, Zr-PZDB enabled the synthesis of various aryl heteroarenes from aryl sulfonium salts and heterocycles. Some of the resultant products may have potential bioactivity. Moreover, the developed Zr-PZDB catalysis can be used for attaining the late-stage functionalization of drug and/or bioactive molecules, including Nikethamide, Admiral, and Myristyl Nicotinate. The MOF scaffold effectively protected the dihydrophenazine active center, resulting in relatively low catalyst loading and good durability. This research not only highlights the potential of MOF engineering to address the limitations of organic catalysis but also paves an innovative avenue to green and sustainable MOF-based EDA photoactivation.

## Methods

Preparation of Zr-PZDB: PZDB-H (4.2 mg, 0.01 mmol), ZrCl_4_ (8.4 mg, 0.036 mmol), and CF_3_COOH (28.5 mg, 0.25 mmol) were mixed in DMF (0.4 mL) in a closable flash. The mixture was then heated at 120 °C for 48 h. After cooling to room temperature naturally, the yellow crystalline solid was obtained by centrifugation and then sequentially washed with DMF three times. Solvent exchange with benzene (3 × 5 mL, replaced by fresh benzene every 8 h) was then conducted. Then, the resultant crystalline solid was dried by vacuum to afford Zr-PZDB (4.4 mg, 82% yield).

General procedure for Minisci-type reaction: *N*-Methoxy pyridinium methylsulfate (0.05 mmol), CH coupling partners (3.8 mmol), NaHCO_3_ (8.4 mg, 0.10 mmol), and Zr-PZDB (1.3 mg, 2.5 μmol, 5 mol% based on linker) were mixed in acetonitrile (0.5 mL) in a sealed test tube. The resulting mixture was stirred under blue LED irradiation (PR160L-427, 390-470 nm) at room temperature in a N_2_ atmosphere for 24 h. After that, the solvent was removed under vacuum, and the residue was subjected to column chromatography on silica gel to give products **3**.

General procedure for heterocycle arylation: Dibenzothiophenium salt (0.2 mmol), heterocycle (8.0 mmol), and Zr-PZDB (5.3 mg, 10.0 μmol, 5.0 mol% based on the linker) were mixed in DMSO (1.0 mL) in a sealed test tube. The resulting mixture was stirred under blue LED irradiation (PR160L-427, 390–470 nm) at room temperature in a N_2_ atmosphere for 24 h. After that, the reaction was quenched with aqueous saturated NaHCO_3_ and diluted with EtOAc. The organic layer was washed with brine, dried with Na_2_SO_4_, filtered, and concentrated in vacuo. The residue was subjected to column chromatography on silica gel to give products **6**.

### Supplementary information

Supplementary Information

Peer Review File

Source_data

## Data Availability

The data supporting the findings of this study are available within the article and its Supplementary Information.

## References

[1] Miranda MA, Garcia H (1994). 2,4,6-triphenylpyrylium tetrafluoroborate as an electron-transfer photosensitizer. Chem. Rev..

[2] Hari DP, König B (2014). Synthetic applications of eosin Y in photoredox catalysis. Chem. Commun..

[3] Romero NA, Nicewicz DA (2016). Organic photoredox catalysis. Chem. Rev..

[4] Concepcion JJ, House RL, Papanikolas JM, Meyer TJ (2012). Chemical approaches to artificial photosynthesis. Proc. Natl Acad. Sci. USA.

[5] Theriot JC (2016). Organocatalyzed atom transfer radical polymerization driven by visible light. Science.

[6] McCarthy B, Sartor S, Cole J, Damrauer N, Miyake GM (2020). Solvent effects and side reactions in organocatalyzed atom transfer radical polymerization for enabling the controlled polymerization of acrylates catalyzed by diaryl dihydrophenazines. Macromolecules.

[7] Zhang Y (2021). Photomediated core modification of organic photoredox catalysts in radical addition: mechanism and applications. Chem. Sci..

[8] Lima CGS, de Lima TM, Duarte M, Jurberg ID, Paixão MW (2016). Organic synthesis enabled by light-irradiation of EDA complexes: theoretical background and synthetic applications. ACS Catal..

[9] Crisenza GEM, Mazzarella D, Melchiorre P (2020). Synthetic methods driven by the photoactivity of electron donor–acceptor complexes. J. Am. Chem. Soc..

[10] Tobisu M, Furukawa T, Chatani N (2013). Visible light-mediated direct arylation of arenes and heteroarenes using diaryliodonium salts in the presence and absence of a photocatalyst. Chem. Lett..

[11] Davies J, Booth SG, Essafi S, Dryfe RAW, Leonori D (2015). Visible-light-mediated generation of nitrogen-centered radicals: metal-free hydroimination and iminohydroxylation cyclization reactions. Angew. Chem. Int. Ed. Engl..

[12] Wu J, Grant PS, Li X, Noble A, Aggarwal VK (2019). Catalyst-free deaminative functionalizations of primary amines by photoinduced single-electron transfer. Angew. Chem. Int. Ed. Engl..

[13] Dewanji A (2023). A general arene C–H functionalization strategy via electron donor–acceptor complex photoactivation. Nat. Chem..

[14] Furukawa H, Cordova KE, O’Keeffe M, Yaghi OM (2013). The chemistry and applications of metal-organic frameworks. Science.

[15] Long JR, Yaghi OM (2009). The pervasive chemistry of metal–organic frameworks. Chem. Soc. Rev..

[16] Spokoyny AM, Kim D, Sumrein A, Mirkin CA (2009). Infinite coordination polymer nano- and microparticle structures. Chem. Soc. Rev..

[17] Zhao D, Timmons DJ, Yuan D, Zhou H-C (2011). Tuning the topology and functionality of metal–organic frameworks by ligand design. Acc. Chem. Res..

[18] Kim Y (2015). Hydrolytic transformation of microporous metal–organic frameworks to hierarchical micro- and mesoporous MOFs. Angew. Chem. Int. Ed. Engl..

[19] Nath I, Chakraborty J, Verpoort F (2016). Metal organic frameworks mimicking natural enzymes: a structural and functional analogy. Chem. Soc. Rev..

[20] Cohen SM (2017). The postsynthetic renaissance in porous solids. J. Am. Chem. Soc..

[21] Lu K, Aung T, Guo N, Weichselbaum R, Lin W (2018). Nanoscale metal–organic frameworks for therapeutic, imaging, and sensing applications. Adv. Mater..

[22] Islamoglu T (2020). Metal–organic frameworks against toxic chemicals. Chem. Rev..

[23] Chen Z, Jiang H, Li M, O’Keeffe M, Eddaoudi M (2020). Reticular chemistry 3.2: typical minimal edge-transitive derived and related nets for the design and synthesis of metal–organic frameworks. Chem. Rev..

[24] Hu Z, Wang Y, Zhao D (2021). The chemistry and applications of hafnium and cerium(IV) metal–organic frameworks. Chem. Soc. Rev..

[25] Wang M, Dong R, Feng X (2021). Two-dimensional conjugated metal–organic frameworks (2D c-MOFs): chemistry and function for MOFtronics. Chem. Soc. Rev..

[26] Liu J, Mukherjee S, Wang F, Fischer RA, Zhang J (2021). Homochiral metal–organic frameworks for enantioseparation. Chem. Soc. Rev..

[27] Zhao M, Ou S, Wu C-D (2014). Porous metal–organic frameworks for heterogeneous biomimetic catalysis. Acc. Chem. Res..

[28] Zhu L, Liu X-Q, Jiang H-L, Sun L-B (2017). Metal–organic frameworks for heterogeneous basic catalysis. Chem. Rev..

[29] Huang Y-B, Liang J, Wang X-S, Cao R (2017). Multifunctional metal–organic framework catalysts: synergistic catalysis and tandem reactions. Chem. Soc. Rev..

[30] Pascanu V, González Miera G, Inge AK, Martín-Matute B (2019). Metal–organic frameworks as catalysts for organic synthesis: a critical perspective. J. Am. Chem. Soc..

[31] Wei Y-S, Zhang M, Zou R, Xu Q (2020). Metal–organic framework-based catalysts with single metal sites. Chem. Rev..

[32] Bavykina A (2020). Metal–organic frameworks in heterogeneous catalysis: recent progress, new trends, and future perspectives. Chem. Rev..

[33] Guo J (2021). Metal–organic frameworks as catalytic selectivity regulators for organic transformations. Chem. Soc. Rev..

[34] Wang K (2021). Porous 2D and 3D covalent organic frameworks with dimensionality-dependent photocatalytic activity in promoting radical ring-opening polymerization. Angew. Chem. Int. Ed. Engl..

[35] Yee K-K (2013). Effective mercury sorption by thiol-laced metal–organic frameworks: in strong acid and the vapor phase. J. Am. Chem. Soc..

[36] Bloch WM (2014). Capturing snapshots of post-synthetic metallation chemistry in metal–organic frameworks. Nat. Chem..

[37] Burgess SA (2016). Improved catalytic activity and stability of a palladium pincer complex by incorporation into a metal–organic framework. J. Am. Chem. Soc..

[38] Korzyński MD, Consoli DF, Zhang S, Román-Leshkov Y, Dincă M (2018). Activation of methyltrioxorhenium for olefin metathesis in a zirconium-based metal–organic framework. J. Am. Chem. Soc..

[39] Grigoropoulos A (2018). Encapsulation of Crabtree’s catalyst in sulfonated MIL-101(Cr): enhancement of stability and selectivity between competing reaction pathways by the MOF chemical microenvironment. Angew. Chem. Int. Ed. Engl..

[40] Cao C-C (2019). Catalysis through dynamic spacer installation of multivariate functionalities in metal–organic frameworks. J. Am. Chem. Soc..

[41] Gao J, Huang Q, Wu Y, Lan Y-Q, Chen B (2021). Metal–organic frameworks for photo/electrocatalysis. Adv. Energy Sustain. Res..

[42] Newar R (2021). Amino acid-functionalized metal-organic frameworks for asymmetric base–metal catalysis. Angew. Chem. Int. Ed. Engl..

[43] Zhang Y (2022). Chiral frustrated Lewis pair@metal-organic framework as a new platform for heterogeneous asymmetric hydrogenation. Angew. Chem. Int. Ed. Engl..

[44] Babucci M, Guntida A, Gates BC (2020). Atomically dispersed metals on well-defined supports including zeolites and metal–organic frameworks: structure, bonding, reactivity, and catalysis. Chem. Rev..

[45] Jin Y, Zhang Q, Zhang Y, Duan C (2020). Electron transfer in the confined environments of metal–organic coordination supramolecular systems. Chem. Soc. Rev..

[46] Jiang Z (2020). Filling metal–organic framework mesopores with TiO_2_ for CO_2_ photoreduction. Nature.

[47] Nyakuchena J (2020). Direct evidence of photoinduced charge transport mechanism in 2D conductive metal organic frameworks. J. Am. Chem. Soc..

[48] Li G, Huang G, Sun R, Curran DP, Dai W (2021). Regioselective radical borylation of α,β-unsaturated esters and related compounds by visible light irradiation with an organic photocatalyst. Org. Lett..

[49] Fiankor C (2021). Symmetry-guided synthesis of N,N′-bicarbazole and porphyrin-based mixed-ligand metal–organic frameworks: light harvesting and energy transfer. J. Am. Chem. Soc..

[50] Fu S (2021). Feeding carbonylation with CO_2_ via the synergy of single-site/nanocluster catalysts in a photosensitizing MOF. J. Am. Chem. Soc..

[51] Liu J (2022). MOF-enabled confinement and related effects for chemical catalyst presentation and utilization. Chem. Soc. Rev..

[52] Shultz AM, Farha OK, Hupp JT, Nguyen ST (2009). A catalytically active, permanently microporous MOF with metalloporphyrin struts. J. Am. Chem. Soc..

[53] Roy S, George CB, Ratner MA (2012). Catalysis by a zinc-porphyrin-based metal–organic framework: from theory to computational design. J. Phys. Chem. C.

[54] Chen W-H, Vázquez-González M, Zoabi A, Abu-Reziq R, Willner I (2018). Biocatalytic cascades driven by enzymes encapsulated in metal–organic framework nanoparticles. Nat. Catal..

[55] Ahn S (2018). Pushing the limits on metal–organic frameworks as a catalyst support: NU-1000 supported tungsten catalysts for o-xylene isomerization and disproportionation. J. Am. Chem. Soc..

[56] Shi Y (2020). Synergistic photoredox and copper catalysis by diode-like coordination polymer with twisted and polar copper–dye conjugation. Nat. Commun..

[57] Li J, He L, Liu Q, Ren Y, Jiang H (2022). Visible light-driven efficient palladium catalyst turnover in oxidative transformations within confined frameworks. Nat. Commun..

[58] Cheng, S et al. Charge separation in metal-organic framework enables heterogeneous thiol catalysis. *Angew. Chem. Int. Ed. Engl.* **62**, e202300993 (2023). 10.1002/anie.202300993 37074229

[59] Zhang W-Q (2016). Robust metal–organic framework containing benzoselenadiazole for highly efficient aerobic cross-dehydrogenative coupling reactions under visible light. Inorg. Chem..

[60] Kinik FP, Ortega-Guerrero A, Ongari D, Ireland CP, Smit B (2021). Pyrene-based metal organic frameworks: from synthesis to applications. Chem. Soc. Rev..

[61] Jin J-K (2021). Building a pyrazole–benzothiadiazole–pyrazole photosensitizer into metal–organic frameworks for photocatalytic aerobic oxidation. J. Am. Chem. Soc..

[62] Cheng S, Tang J, Quan Y (2022). Metal-organic frameworks with organic photosensitizers in organic synthesis. Eur. J. Inorg. Chem..

[63] Martín N, Cirujano FG (2020). Organic synthesis of high added value molecules with MOF catalysts. Org. Biomol. Chem..

[64] Dai G (2019). A dual-ion organic symmetric battery constructed from phenazine-based artificial bipolar molecules. Angew. Chem. Int. Ed. Engl..

[65] Kochetygov I (2023). A simple, transition metal catalyst-free method for the design of complex organic building blocks used to construct porous metal–organic frameworks. Angew. Chem. Int. Ed. Engl..

[66] Cavka JH (2008). A new zirconium inorganic building brick forming metal organic frameworks with exceptional stability. J. Am. Chem. Soc..

[67] Thiel O (2013). Heterocyclic chemistry in drug discovery. Edited by Jie Jack Li. Angew. Chem. Int. Ed. Engl..

[68] Proctor RSJ, Phipps RJ (2019). Recent advances in Minisci-type reactions. Angew. Chem. Int. Ed. Engl..

[69] He F-S, Ye S, Wu J (2019). Recent advances in pyridinium salts as radical reservoirs in organic synthesis. ACS Catal..

[70] Rössler SL (2020). Pyridinium salts as redox-active functional group transfer reagents. Angew. Chem. Int. Ed. Engl..

[71] Quint V (2016). Metal-free, visible light-photocatalyzed synthesis of benzo[b]phosphole oxides: synthetic and mechanistic investigations. J. Am. Chem. Soc..

[72] Bahamonde A, Melchiorre P (2016). Mechanism of the stereoselective α-alkylation of aldehydes driven by the photochemical activity of enamines. J. Am. Chem. Soc..

[73] Li J (2020). Photoredox catalysis with aryl sulfonium salts enables site-selective late-stage fluorination. Nat. Chem..

[74] Cismesia MA, Yoon TP (2015). Characterizing chain processes in visible light photoredox catalysis. Chem. Sci..

[75] Buquoi JQ, Lear JM, Gu X, Nagib DA (2019). Heteroarene phosphinylalkylation via a catalytic, polarity-reversing radical cascade. ACS Catal..

[76] Jung S, Shin S, Park S, Hong S (2020). Visible-light-driven C4-selective alkylation of pyridinium derivatives with alkyl bromides. J. Am. Chem. Soc..

[77] Rammal F (2020). Photochemical C–H silylation and hydroxymethylation of pyridines and related structures: synthetic scope and mechanisms. ACS Catal..

[78] Rammal F (2020). Visible-light-mediated C–H alkylation of pyridine derivatives. Org. Lett..

[79] Shen L (2020). External oxidant-free alkylation of quinoline and pyridine derivatives. Org. Biomol. Chem..

[80] Kim M, Koo Y, Hong S (2022). N-functionalized pyridinium salts: a new chapter for site-selective pyridine C–H functionalization via radical-based processes under visible light irradiation. Acc. Chem. Res..

