# Supplementary material for: Metal-organic framework boosts heterogeneous electron donor–acceptor catalysis
Source: Nat Commun. 2023 Nov 27;14:7757. doi: 10.1038/s41467-023-43577-5 (PMC10682007; doi:10.1038/s41467-023-43577-5)
Supplement: Supplementary file 1 — Supplementary Information [file 41467_2023_43577_MOESM1_ESM.pdf]

Supporting Information for

Metal-Organic Framework Boosts Heterogeneous Electron Donor–Acceptor Catalysis

Jiaxin Lin,† Jing Ouyang,† Tianyu Liu, Fengxing Li, Herman Ho-Yung SUNG, Ian Williams, Yangjian Quan*

Department of Chemistry and the Hong Kong Branch of Chinese National Engineering Research Centre for Tissue Restoration & Reconstruction, The Hong Kong University of Science and Technology (HKUST), Kowloon, Hong Kong SAR, China

†These authors contributed equally to this work

E-mail: chyjquan@ust.hk

1. Materials and Methods	S2
2. Synthesis and Characterization of **Zr-PZDB**	S3
3. EDA Catalytic Reactions	S17
4. Mechanistic Study	S41
5. NMR Spectra	S53
6. Reference	S98

1. Materials and Methods

All the reactions and manipulations were carried out under N_2 with the use of a glovebox or Schlenk technique, unless otherwise indicated. All the solvents were dried by standard procedures. Pyridine *N*-oxides were prepared according to literature methods.[1] All other chemicals were purchased from Aldrich, Energy, or Macklin, and used directly unless otherwise indicated.

Powder X-ray diffraction (PXRD) data was collected on a PANalytical X'Pert Pro powder-X-ray diffractometer with a Cu source (λ = 1.54056 Å). N_2 sorption experiments were performed on a Quantachrome Autosorb iQ gas sorption analyzer. Samples were outgassed at 0.03 torr with a 5 °C/min ramp to 40 °C and held at 40 °C for 2 hours. The samples were then held in vacuum until the analysis was run. Pore analysis was performed using N_2 at 77 K (P/P_0 range of 2×10^{-7} to 0.995). Thermogravimetric analysis (TGA) was performed in a UNIX/TGA7 (Perkin Elmer) thermal analyzer in air ranging from 40 to 800 °C with a heating rate of 15 °C /min. Transmission electron microscopy (TEM) images were taken on Tecnai G2 F20 U-TWIN Transmission Electron Microscope (TEM). Scanning electron microscopy (SEM) images and energy dispersive X-ray (EDX) spectrum were taken on a TM3030 Tabletop Microscope operating at 5 kV and 15 kV. Fluorescence measurement was performed using an EI-FS5 Fluoresce Spectrometer. IR spectra were recorded as KBr pellets on a on a Bruker Tensor 27 Fourier transform infrared (FT-IR) spectrum radiometer from 4000 to 400 cm^{-1}. UV-vis absorption spectroscopic measurements were performed using a UH5700 spectrophotometer equipped with a 60 mm diameter integrating sphere. ICP-OES analysis was performed on AVIO-200 Perkin Elmer. Mass spectra were collected on an Agilent GC/MS 5975C system, a MALDI Micro MX mass spectrometer, or an API QSTAR XL System. ^{1}H NMR and ^{13}C NMR spectra were recorded on a Bruker AV NMR 400 or JEOL 600 spectrometer at 400/600 MHz and 100/150 MHz, respectively. The following abbreviations are used herein: s: singlet, d: doublet, t: triplet, q: quartet, m: multiplet. Kessil PR160L-427 or -390 LED lamps were used for reactions.

2. Synthesis and Characterization of Zr-PZDB

2.1 Synthesis and Characterization of PZDB Linker

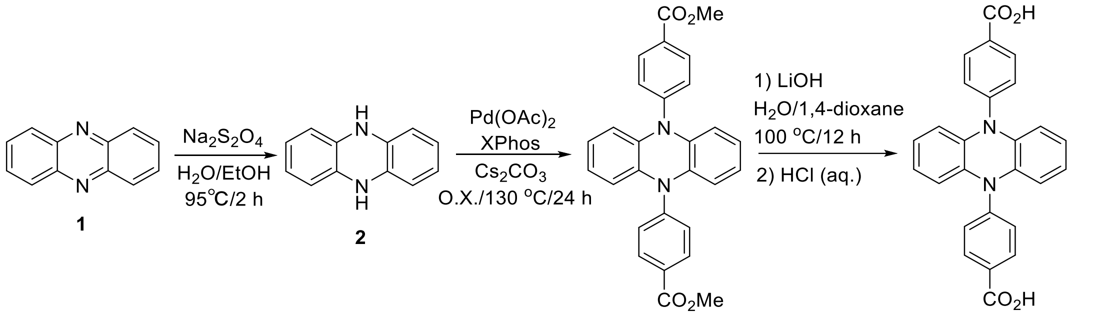

Step 1. A solution of sodium dithionite (14.63 g, 84.0 mmol) in water (130 mL) was slowly added to a boiling solution of phenazine **1** (1.08 g, 6.0 mmol) in EtOH (30 mL). The mixture was refluxed at 95 °C for 2 h. The resulting greenish gray precipitate was filtered off, washed by water, and dried under vacuum to give the compound **2** (0.98g) in 90% yield.

Step 2. A mixture of 5,10-dihydrophenazine **2** (0.728 g, 4.0 mmol), methyl 4-bromobenzoate (1.89 g, 8.8 mmol), $Pd(OAc)_2$ (44.9 mg, 0.2 mmol, 5 mol%), Xphos (191 mg, 0.4 mmol, 10 mol%) and Cs_2CO_3 (2.61 g, 8 mmol) was added to a Schlenk flask under N_2 atmosphere. Then anhydrous *o*-xylene (50 mL) was added. The resultant mixture was stirred at 130 °C for 24 h. After cooling to room temperature, water (60 mL) was added, and the organic phase was extracted with dichloromethane (DCM) (90 mL ×2). After the removal of organic solvent under vacuum, the residue was subjected to column chromatography to afford **PZDB-Me** (1.49 g) in 85% yield. ^{1}H NMR (400 MHz, DMSO-d_6) δ 8.22 (d, J = 8.4 Hz, 4H), 7.58 (d, J = 8.4 Hz, 4H), 6.41 (dd, J = 6.0, 3.4 Hz, 4H), 5.72 (dd, J = 5.9, 3.4 Hz, 4H), 3.90 (s, 6H). HRMS (ESI) calcd for $C_{28}H_{23}N_2O_4$ $[M+H]^+$ 451.1652, found 451.1613. Characterization data matched that reported in the literature.[2]

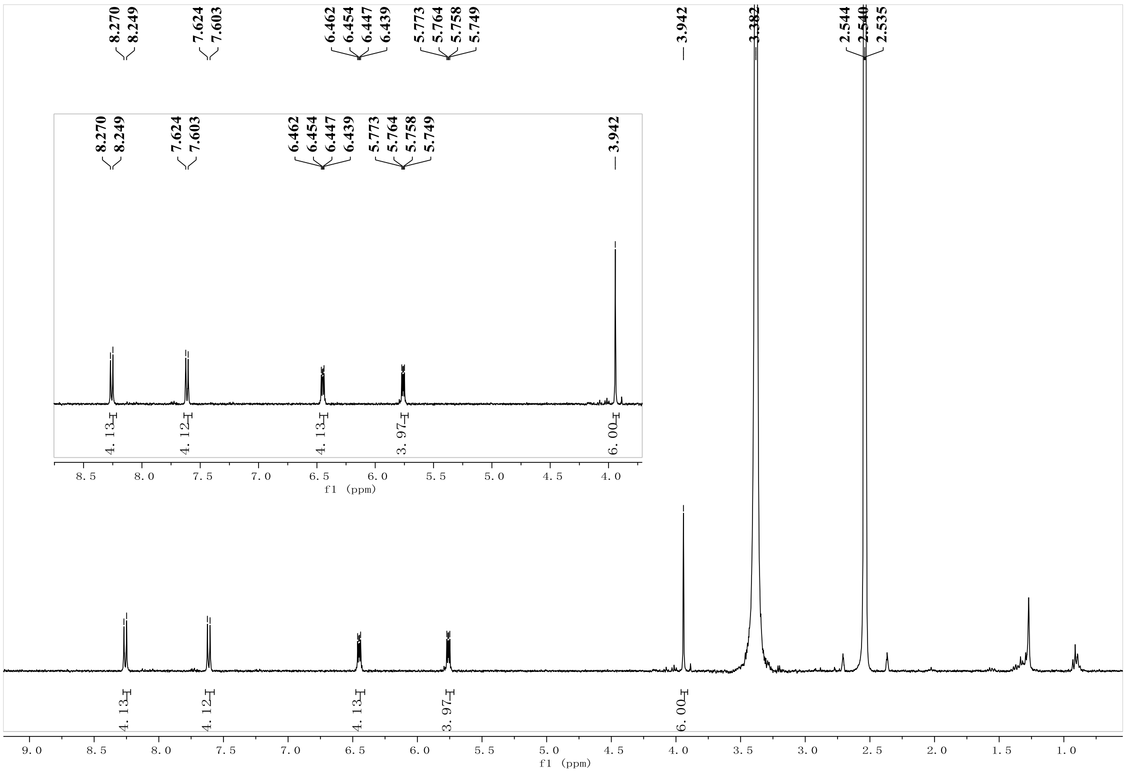

Figure S1. ^{1}H NMR spectrum of **PZDB-Me** in DMSO-d_6

Step 3. A suspension of **PZDB-Me** (1.49 g, 3.0 mmol) and LiOH (0.72 g, 30.0 mmol) in dioxane (30 mL) and water (15 mL) was heated at 100°C for 12 h under dry N_2. After cooling down to room temperature, HCl aqueous solution was added to the reaction mixture until pH = 2. The yellow precipitate was filtered and washed by water, hexane, and dichloromethane to give **PZDB-H** (1.13 g) in 92 % yield. NMR analysis was carried out by dissolving **PZDB-H** (2 mg) in the mixture of NaOH/D_2O solution (50 mg NaOH in 200 μL D_2O) and DMSO-d_6 (1 mL) with sonication for 3 minutes. After centrifugation, the supernatant solution was placed in a NMR tube for analysis. ^{1}H NMR (400 MHz, DMSO-d_6) δ 8.09 (d, J = 8.3 Hz, 4H), 7.27 (d, J = 8.6 Hz, 4H), 6.24 (dd, J = 5.9, 3.3 Hz, 4H), 5.50 (dd, J = 6.2, 3.1 Hz, 4H). ^{13}C NMR spectrum cannot be obtained due to the low solubility. HRMS (ESI) calcd for $C_{26}H_{19}N_2O_4$ $[M+H]^+$ 423.1267, found 423.1295.

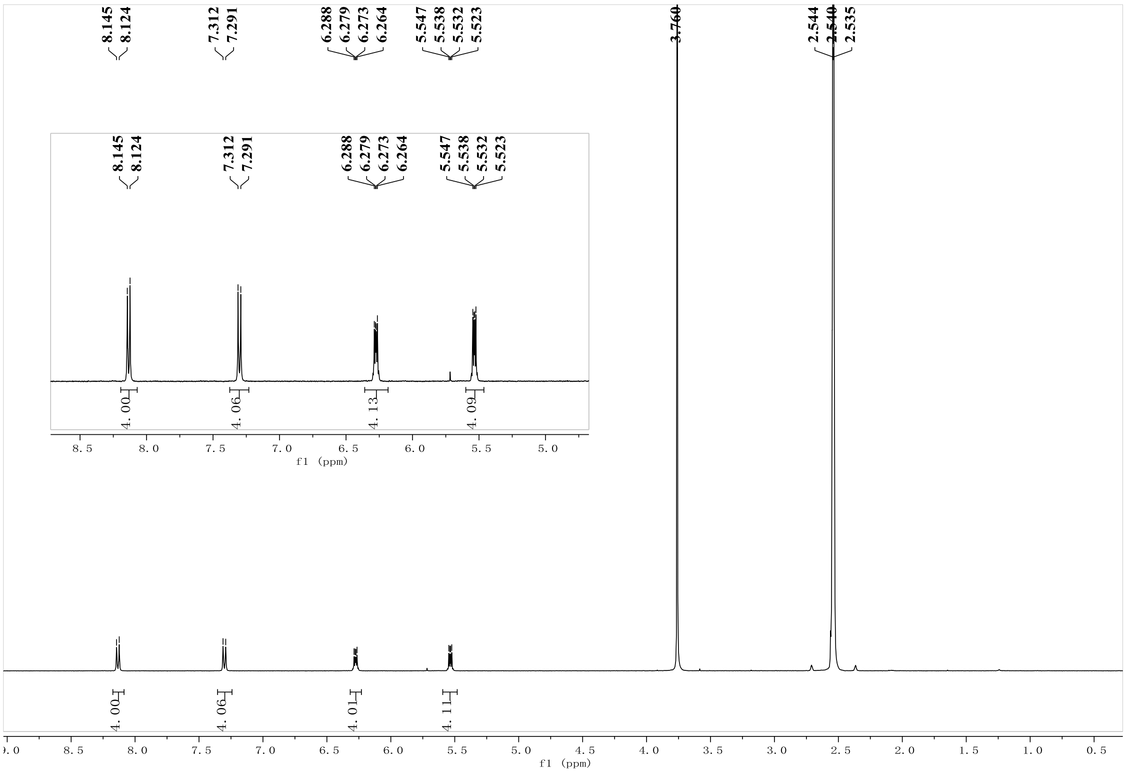

Figure S2. ^{1}H NMR spectrum of **PZDB-Na** in $NaOH/D_2O/DMSO\text{-}d_6$

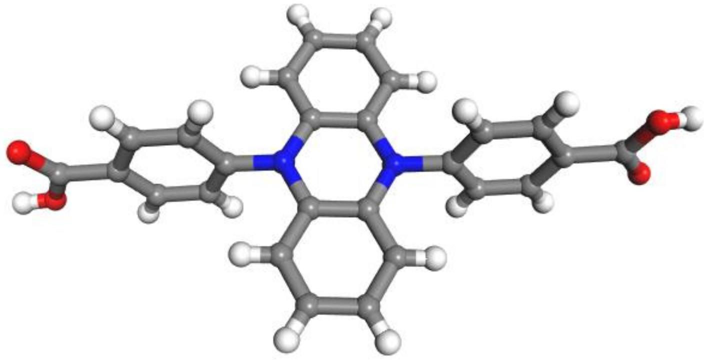

Figure S3. Single crystal structure of **PZDB-H**

Table S1. Crystal data and structure refinement for **PZDB-H**

Identification code	PZDB
Empirical formula	$C_{32}H_{32}N_4O_6$ (**PZDB-H** + 2 DMF)
Formula weight	568.61
Temperature	296(2) K
Wavelength	0.71073 Å
Crystal system	Triclinic
Space group	P-1
Unit cell dimensions	a = 7.340(4) Å; α = 72.365(16)°.
	b = 8.946(5) Å; β = 77.706(17)°.
	c = 12.009(7) Å; γ = 81.338(17)°.

Volume	731.1(7) Å^3
Z	1
Density (calculated)	1.291 Mg/m^3
Absorption coefficient	0.090 mm^{-1}
F(000)	300
Crystal size	0.400 x 0.300 x 0.200 mm^3
Theta range for data collection	2.852 to 25.249°.
Index ranges	-8<=h<=8, -10<=k<=10, -14<=l<=14
Reflections collected	18375
Independent reflections	2623 [R(int) = 0.0275]
Completeness to theta = 25.242°	99.2 %
Absorption correction	multi-scan
Max. and min. transmission	0.7456 and 0.6932
Refinement method	Full-matrix least-squares on F^2
Data / restraints / parameters	2623 / 1 / 204
Goodness-of-fit on F^2	1.084
Final R indices [I>2sigma(I)]	R1 = 0.0736, wR2 = 0.1816
R indices (all data)	R1 = 0.0798, wR2 = 0.1908
Extinction coefficient	0.91(8)
Largest diff. peak and hole	0.601 and -0.506 e.Å^{-3}
CCDC	2233893

https://www.ccdc.cam.ac.uk/structures/Search?ccdc=2233893

2.2. Synthesis and Characterization of Zr-PZDB

2.2.1 Synthesis of Zr-PZDB

PZDB-H (4.2 mg, 0.01 mmol), $ZrCl_4$ (8.4 mg, 0.036 mmol), and CF_3COOH (28.5 mg, 0.25 mmol) were mixed in DMF (0.8 mL) in a closable flash. The mixture was then heated at 120 °C for 48 h. After cooling to room temperature naturally, the yellow crystalline solid was obtained by centrifugation and then sequentially washed with DMF three times. Solvent exchange with benzene (3 × 5 mL, replaced by fresh benzene every 8 hours) was then conducted. Then, the resultant crystalline solid was dried by vacuum to afford **Zr-PZDB** (4.4 mg, 82% yield).

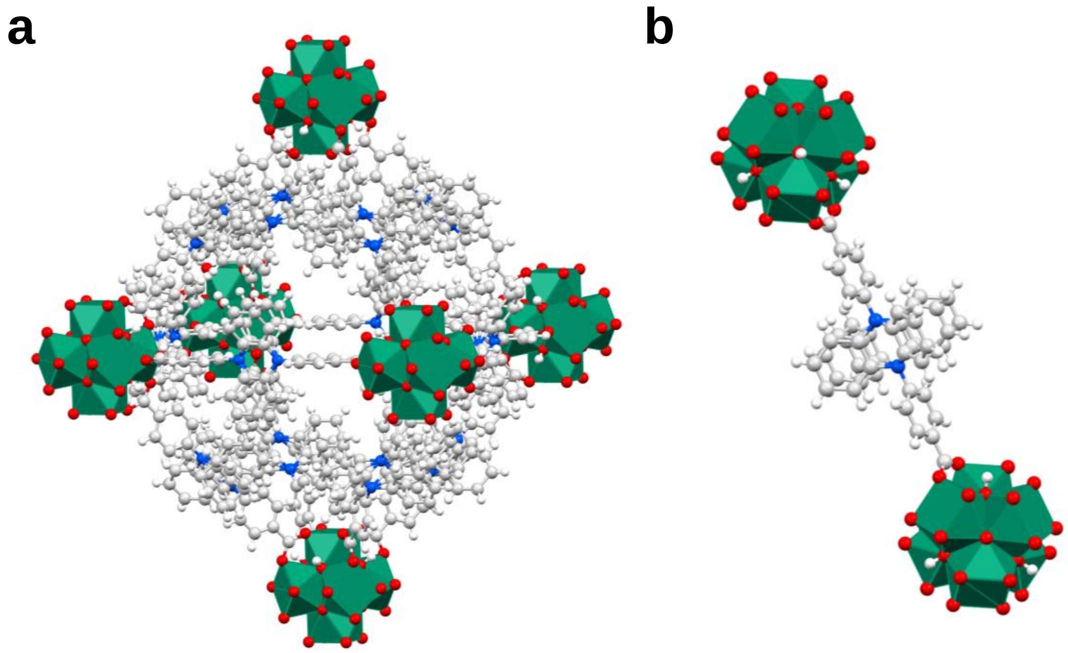

Figure S4. (a) Single crystal structure of **Zr-PZDB** represented by an octahedron-like cage; green: Zr, red: O, blue: N, grey: C, white: H. (b) Disorder of dihydrophenazine moiety in PZDB of **Zr-PZDB**

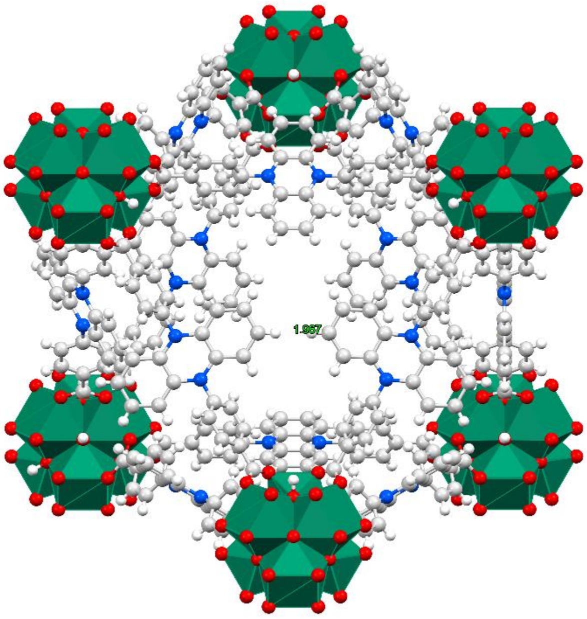

Figure S5. Single crystal structure of **Zr-PZDB** showing the shortest distance of ~2.0 Å between two adjacent PZDB linkers

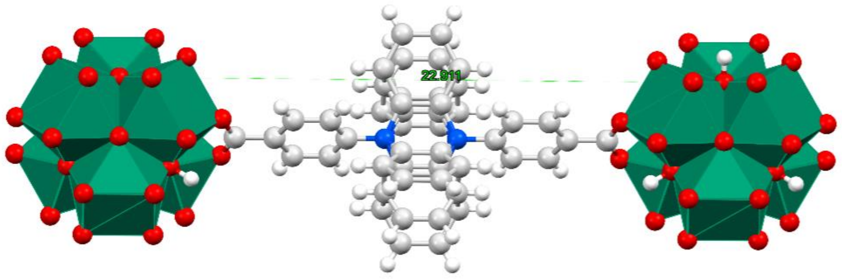

Figure S6. Single crystal structure of **Zr-PZDB** showing the distance of ~22.9 Å between two Zr_6-SBUs

Table S2. Crystal data and structure refinement for **Zr-PZDB**

Identification code	linjx10_green
Empirical formula	$C_{156}H_{100}N_{12}O_{32}Zr_6$
Formula weight	3201.79
Temperature	100.01(10)
Wavelength	0.71073 Å
Crystal system	cubic
Space group	Fm-3m
Unit cell dimensions	a = 32.4013(6) Å; α = 90°.
	b = 32.4013(6) Å; β = 90°.
	c = 32.4013(6) Å; γ = 90°.
Volume	34016.2(18) $Å^3$
Z	4
Density (calculated)	0.625 g/cm^3
Absorption coefficient	1.737 mm^{-1}
F(000)	6464.0
Crystal size	0.06 × 0.06 × 0.05 mm^3
Theta range for data collection	9.052 to 148.462°.
Index ranges	$-27 \leq h \leq 37$, $-39 \leq k \leq 24$, $-16 \leq l \leq 39$
Reflections collected	9094
Independent reflections	1712 [R_{int} = 0.0762, R_{sigma} = 0.0511]
Data / restraints / parameters	1712/183/89
Goodness-of-fit on F^2	1.018
Final R indices [I>2sigma(I)]	R_1 = 0.0561, wR_2 = 0.1567
R indices (all data)	R_1 = 0.0700, wR_2 = 0.1673
Largest diff. peak and hole	1.23/-0.73 e.$Å^{-3}$

CCDC 2258527
https://www.ccdc.cam.ac.uk/structures/Search?ccdc=2258527

2.2.2. ^{1}H NMR Analysis of Digested Zr-PZDB

Zr-PZDB (10 mg) was digested in a NaOH solution in D_2O (10 mg NaOH in 50 µL D_2O) under sonication for 3 minutes. The mixture was then filtered, and the filtrate was dissolved in DMSO-d_6 (0.5 mL) for ^{1}H NMR analysis.

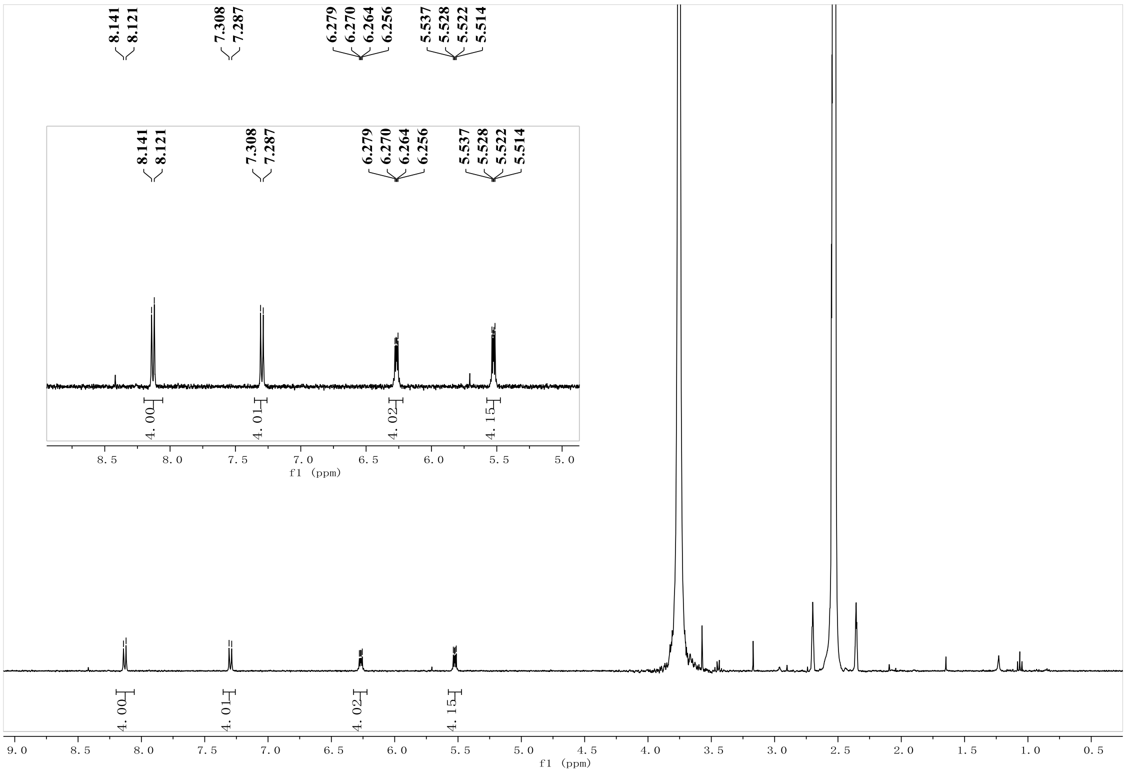

Figure S7. ^{1}H NMR spectrum of digested **Zr-PZDB** in NaOH/D_2O/DMSO-d_6

2.2.3 Thermogravimetric Analysis of Zr-PZDB MOFs

The as-made **Zr-PZDB** was characterized by thermogravimetric analysis (TGA, Figure S8). The measured ZrO_2 residue of 23.7% is close to the value of 23.1% calculated from the fitting formula.

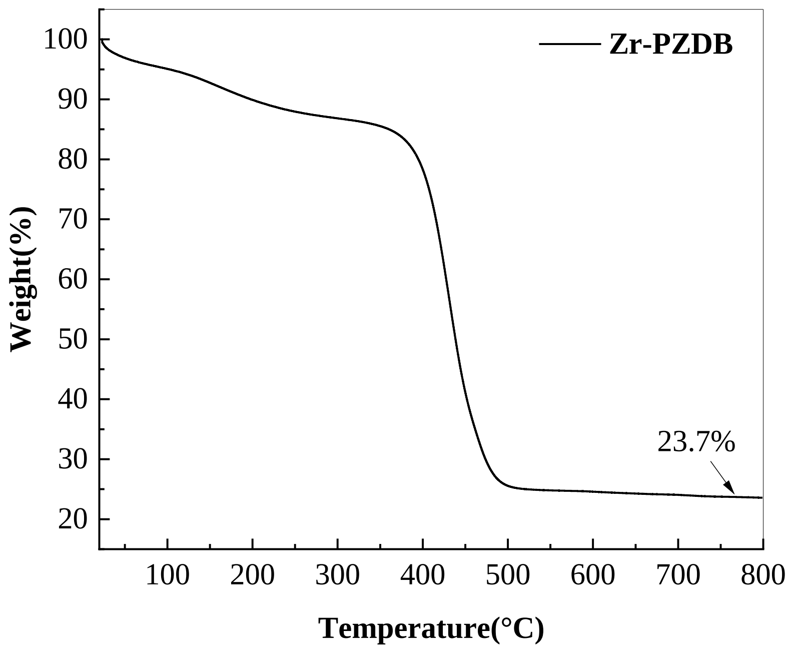

Figure S8. TGA plot of **Zr-PZDB** (in air, 5 °C/min)

2.2.4 N_2 Adsorption Analyses of Zr-PZDB

The as-synthesized **Zr-PZDB** (60 mg) was washed with fresh DMF three times (20 mL). Prior to activation, the sample was stored in DMF (20 mL). The DMF-exchanged **Zr-PZDB** was then placed in a vial and soaked in DCM (20 mL) for 20 minutes. This step was repeated three times with fresh DCM. After that, the **Zr-PZDB** was transferred to a Soxhlet extractor using a dropper. The MOF solid was extracted with DCM for 12 hours. Then the DCM-exchanged MOFs were soaked in fresh hexane (20 mL) for 20 minutes. This step was also repeated three times with fresh hexane, followed by a similar overnight hexane Soxhlet extraction. Afterwards, the **Zr-PZDB** was transferred to a vial with hexane. The remaining hexane gradually volatilized in a nitrogen-filled glovebox for 3 hours while fan blowing. Then the dried sample was loaded in an adsorption tube and activated at 40 °C for 2 hours before the adsorption measurement.

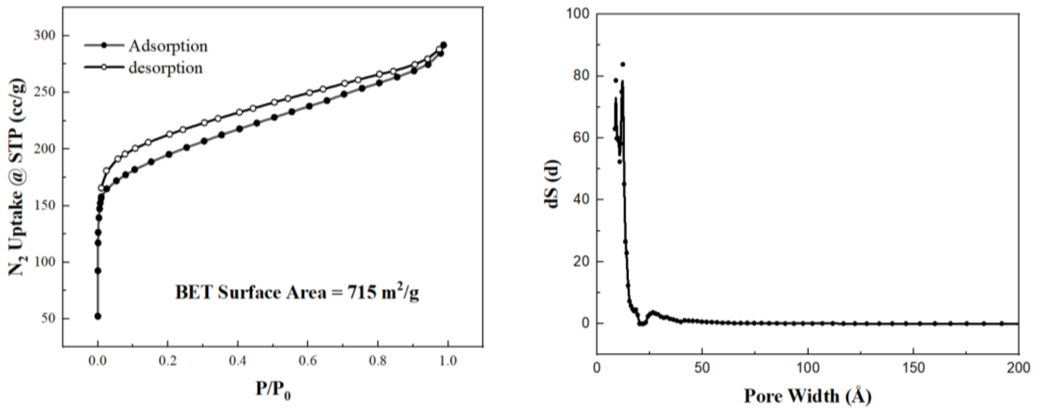

Figure S9. N_2 adsorption-desorption isotherms (left) and pore size distribution profiles (right) of **Zr-PZDB**

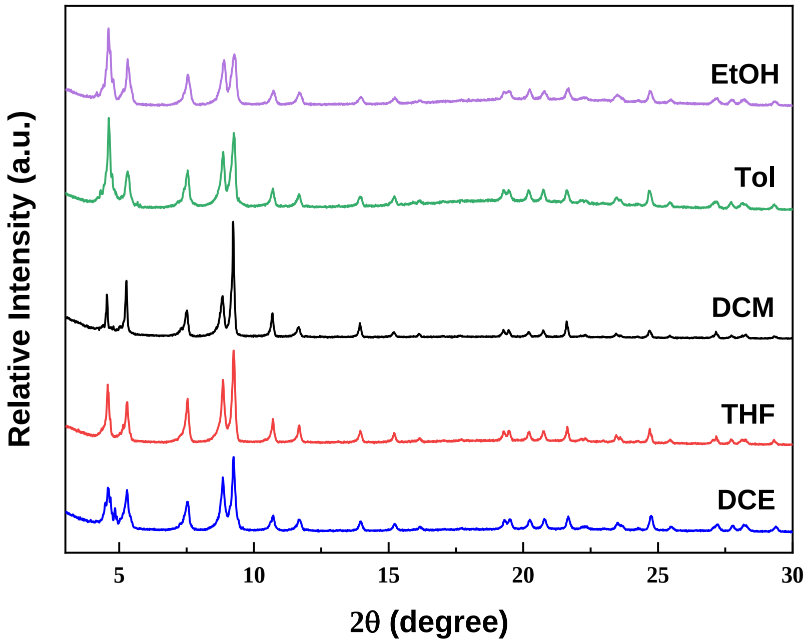

Figure S10. PXRD patterns of **Zr-PZDB** in different solvents

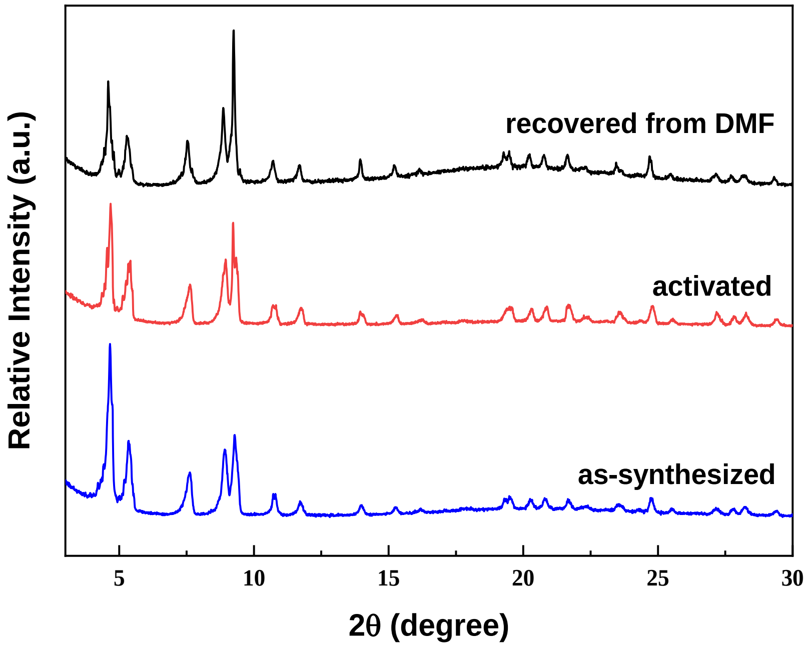

Figure S11. PXRD patterns of **Zr-PZDB** (as-synthesized, activated and recovered from DMF)

2.3 UV-Vis Determination of EDA Interactions

2.3.1 UV-Vis Determination of EDA Interaction between Zr-PZDB and 1a

UV-Vis spectrum of **1a**: 1.0 mL of **1a** (0.005 M in MeCN) was added to a cuvette (3.5 ml) along with 1.0 mL of anhydrous MeCN. The cuvette was then sealed and incubated for 5 minutes at ambient temperature with a Bech mixer. Afterwards the sample was tested for absorption from 300 nm to 600 nm. UV-Vis spectrum of **Zr-PZDB**: 1.0 mL **Zr-PZDB** (0.005 M in MeCN, based on the linker) was added to a cuvette (3.5 ml) along with 1.0 mL of anhydrous MeCN. The cuvette was then sealed and ultrasonicated for 5 minutes at ambient temperature. Afterwards the sample was tested for absorption from 300 nm to 600 nm. UV-Vis spectrum of the mixture: 1.0 mL of **1a** (0.005 M in MeCN) and 1.0 mL of **Zr-PZDB** (0.005 M in MeCN, based on the linker) were added to a cuvette (3.5 ml). The cuvette was then sealed and ultrasonicated for 5 minutes at ambient temperature. Afterwards the sample was tested for absorption from 300 nm to 600 nm.

2.3.2 UV-Vis Determination of EDA Interaction between PZDB-H and 1a

UV-Vis spectrum of **PZDB-H**: 1.0 mL of **PZDB-H** (0.005 M in MeCN) was added to a cuvette (3.5 ml) along with 1.0 mL of anhydrous MeCN. The cuvette was then sealed and incubated for 5 minutes at ambient temperature with a Bech mixer. Afterwards the sample was tested for absorption from 300 nm to 600 nm. UV-Vis spectrum of **1a**: 1.0 mL **1a** (0.005 M in MeCN) was added to a cuvette (3.5 ml) along with 1.0 mL of anhydrous MeCN. The cuvette was then sealed and incubated for 5 minutes at ambient temperature with a Bech mixer. Afterwards the sample was tested for absorption from 300 nm to 600 nm. UV-Vis spectrum of the mixture: 1.0 mL of **PZDB-H** (0.005 M in MeCN) and 1.0 mL of **1a** (0.005 M in MeCN) were added to a cuvette (3.5 ml). The cuvette was then sealed and incubated for 5 minutes at ambient temperature with a Bech mixer. Afterwards the sample was tested for absorption from 300 nm to 600 nm.

2.3.3 UV-Vis Analyses of the Mixtures of Zr-PZDB and 1a with Different Ratios

Two stock solutions (0.005 M in MeCN) of **1a** and **Zr-PZDB** were prepared, respectively. Seven samples including 2.0 mL **1a** (1), 1.75 mL **1a** and 0.25 mL **Zr-PZDB** (2), 1.5 mL **1a** and 0.5 mL **Zr-PZDB** (3), 1.0 mL **1a** and 1.0 mL **Zr-PZDB** (4), 0.5 mL **1a** and 1.5 mL **Zr-PZDB** (5), 0.25 mL **1a** and 1.75 mL **Zr-PZDB** (6), and 2.0 mL **Zr-PZDB** (7) were prepared and analyzed by UV-vis spectroscopy for absorption from 300 nm to 600 nm. The corresponding spectra were compiled in Figure 2j. The absorption intensity at 375 nm, the significantly enhanced peak probably originating from the EDA interaction, was measured for each sample, respectively, as shown in Table S3.

2.3.4 Job Plot

Job plot was based on the UV-vis absorption data shown in Figure 2j.[3] The absorption intensity at 375 nm of **1a** was measured as 0.00669 in the absence of **Zr-PZDB**. The corresponding absorption intensity and calculations were compiled in Table S3.

Table S3. UV-vis results for Job plot

entry	V (**1a**, mL)	V (**Zr-PZDB**, mL)	X (**1a**)	A (370 nm)	ΔA (370 nm)	X (**1a**) * ΔA
1	2.0	0	1	0.00669	0	0
2	1.75	0.25	0.875	0.38615	0.37946	0.304
3	1.5	0.5	0.75	0.54902	0.54233	0.363
4	1.0	1.0	0.5	1.04870	1.04201	0.521
5	0.5	1.5	0.25	1.12757	1.12088	0.381
6	0.25	1.75	0.125	1.23065	1.22396	0.245
7	0	2.0	0	1.29781	1.29112	0

2.3.5 UV-Vis Determination of EDA Interaction between Zr-PZDB and 4a

UV-Vis spectrum of **4a**: 1.0 mL of **4a** (0.001 M, low concentration or 0.005 M, high concentration in DMSO) was added to a cuvette (3.5 ml) along with 1.0 mL of anhydrous DMSO. The cuvette was then sealed and incubated for 5 minutes at ambient temperature with a Bech mixer. Afterwards the sample was tested for absorption from 300 nm to 600 nm. UV-Vis spectrum of **Zr-PZDB**: 1.0 mL **Zr-PZDB** (0.001 M, low concentration or 0.005 M, high concentration in DMSO, based on the linker) was added to a cuvette (3.5 ml) along with 1.0 mL of anhydrous DMSO. The cuvette was then sealed and ultrasonicated for 5 minutes at ambient temperature. Afterwards the sample was tested for absorption from 300 nm to 600 nm. UV-Vis spectrum of the mixture: 1.0 mL of **4a** (0.001 M, low concentration or 0.005 M, high concentration in DMSO) and 1.0 mL of **Zr-PZDB** (0.001 M, low concentration or 0.005 M, high concentration in DMSO, based on the linker) were added to a cuvette (3.5 ml). The cuvette was then sealed and ultrasonicated for 5 minutes at ambient temperature. Afterwards the sample was tested for absorption from 300 nm to 600 nm.

2.3.6 UV-Vis Determination of EDA Interaction between PZDb-H and 4a

UV-Vis spectrum of **PZDB-H**: 1.0 mL of **PZDB-H** (0.001 M, low concentration or 0.005 M, high concentration in DMSO) was added to a cuvette (3.5 ml) along with 1.0

mL of anhydrous DMSO. The cuvette was then sealed and incubated for 5 minutes at ambient temperature with a Bech mixer. Afterwards the sample was tested for absorption from 300 nm to 600 nm. UV-Vis spectrum of **4a**: 1.0 mL **1a** (0.001 M, low concentration or 0.005 M, high concentration in DMSO) was added to a cuvette (3.5 ml) along with 1.0 mL of anhydrous DMSO. The cuvette was then sealed and incubated for 5 minutes at ambient temperature with a Bech mixer. Afterwards the sample was tested for absorption from 300 nm to 600 nm. UV-Vis spectrum of the mixture: 1.0 mL of **PZDB-H** (0.001 M, low concentration or 0.005 M, high concentration in DMSO) and 1.0 mL of **4a** (0.001 M, low concentration or 0.005 M, high concentration in DMSO) were added to a cuvette (3.5 ml). The cuvette was then sealed and incubated for 5 minutes at ambient temperature with a Bech mixer. Afterwards the sample was tested for absorption from 300 nm to 600 nm.

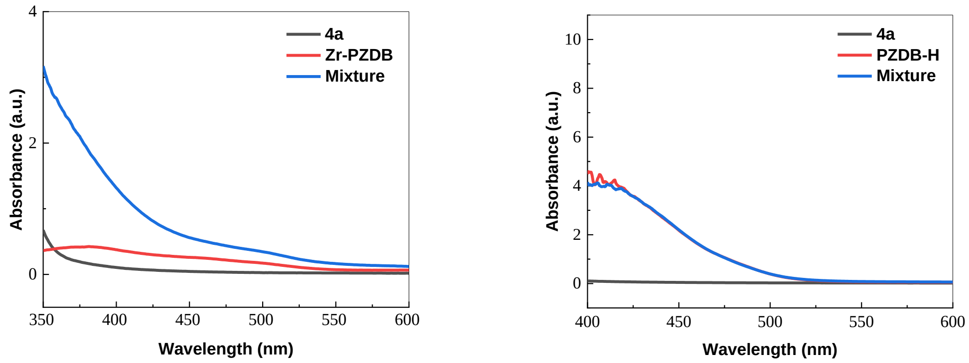

Figure S12. UV-vis spectra of **4a**, **Zr-PZDB**, and their mixture in DMSO (2.5×10^{-3} M), left and of **4a**, PZDB-H, and their mixture in DMSO (2.5×10^{-3} M), right

2.3.7 UV-Vis Determination of EDA Interaction between $NaHCO_3$ and 1a

UV-Vis spectrum of **$NaHCO_3$**: 1.0 mL of **$NaHCO_3$** (0.005 M in MeCN) was added to a cuvette (3.5 ml) along with 1.0 mL of anhydrous MeCN. The cuvette was then sealed and ultrasonicated for 5 minutes at ambient temperature. Afterwards the sample was tested for absorption from 300 nm to 600 nm. UV-Vis spectrum of **1a**: 1.0 mL **1a** (0.005 M in MeCN) was added to a cuvette (3.5 ml) along with 1.0 mL of anhydrous MeCN. The cuvette was then sealed and incubated for 5 minutes at ambient temperature with a

Bech mixer. Afterwards the sample was tested for absorption from 300 nm to 600 nm. UV-Vis spectrum of the mixture: 1.0 mL of **$NaHCO_3$** (0.005 M in MeCN) and 1.0 mL of **1a** (0.005 M in MeCN) were added to a cuvette (3.5 ml). The cuvette was then sealed and ultrasonicated for 5 minutes at ambient temperature. Afterwards the sample was tested for absorption from 300 nm to 600 nm.

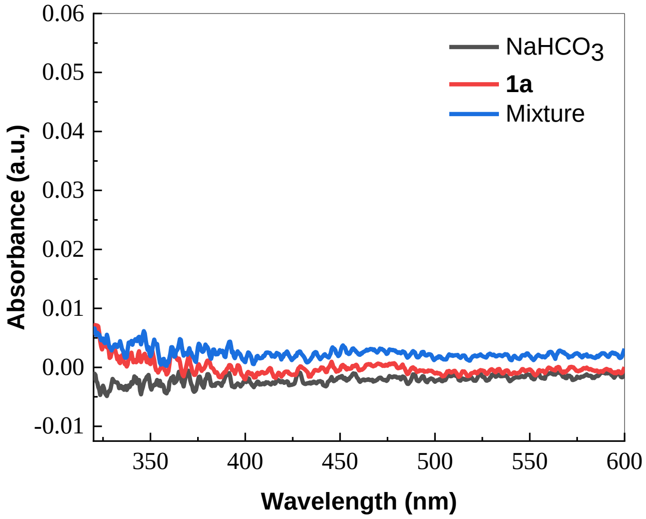

Figure S13. UV-vis spectra of **1a**, $NaHCO_3$, and their mixture in MeCN (2.5×10^{-3} M)

3. Catalytic reactions

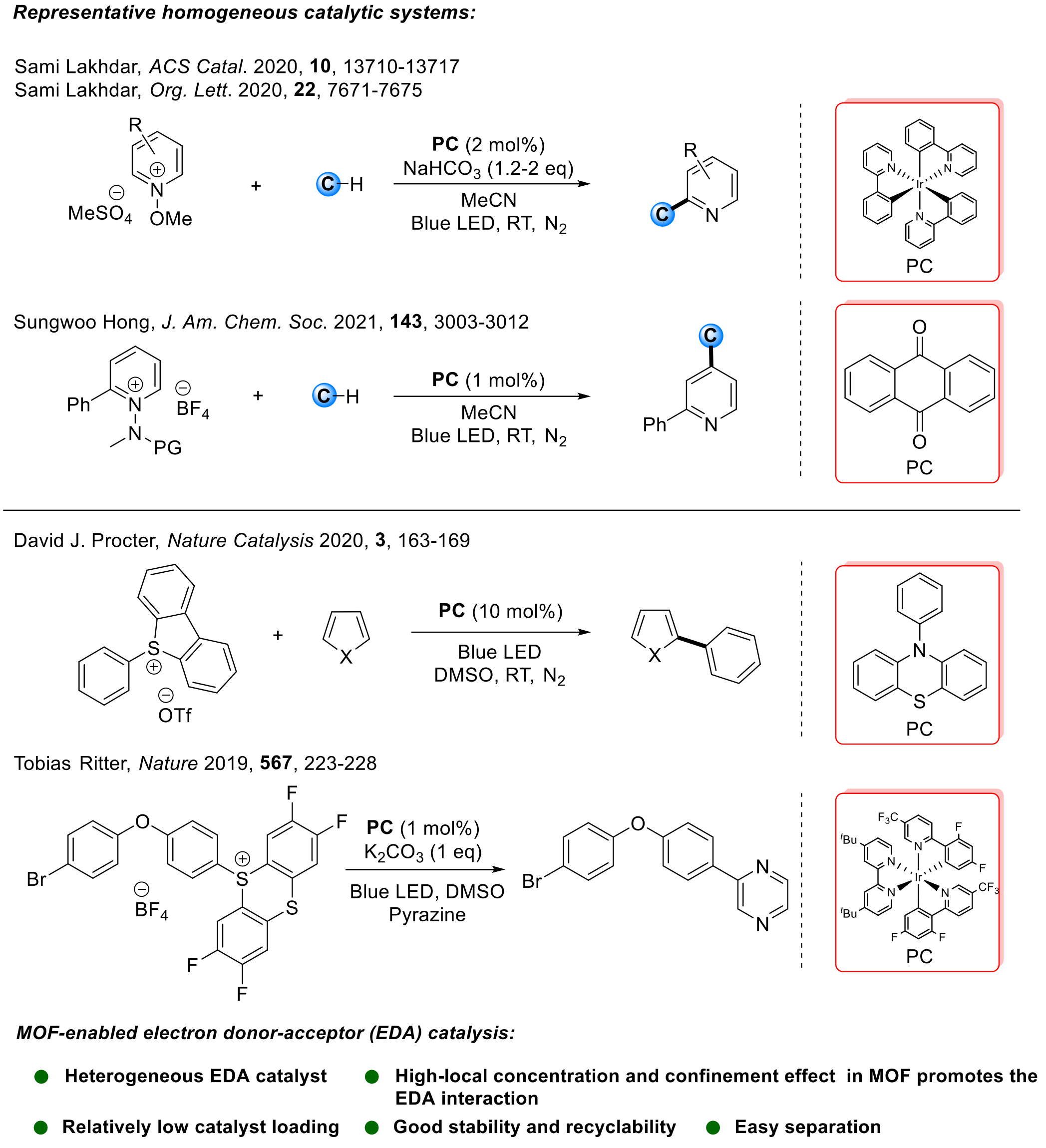

Figure S14. Comparison with homogeneous catalysis

3.1 Zr-PZDB Catalyzed Minisci-Type Cross-Coupling

3.1.1 Synthesis of Pyridinium Salts

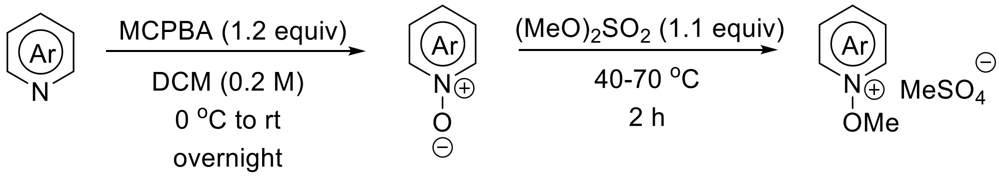

General procedures. Step 1: To a 10 ml round bottom flask with a stirrer bar was added pyridine (1.0 mmol) and DCM (5 ml, 0.2 M). The mixture was then stirred at 0 °C for

15min. MCPBA (243.6 mg, 1.2 mmol, 85%) was added in proportions. Later on, the resultant mixture was allowed to ambient temperature and stirred overnight. After removing the solvent via vacuum, the residue was subjected to flash column chromatography on silica gel to give the pyridine *N*-oxide. Step 2: To a 10 ml vial with a stirrer bar was added pyridine *N*-oxide (0.45 mmol) and dimethyl sulfate (46.9 μL, 0.495 mmol). The mixture was then heated at 40-50 °C to get a homogeneous solution, which was then heated at 70°C and stirred for additional 2 hours. The excess dimethyl sulfate was removed under vacuum for at least 12 hours, and the resultant pyridinium salt was used without further purification.

3.1.2 Coupling Reactions

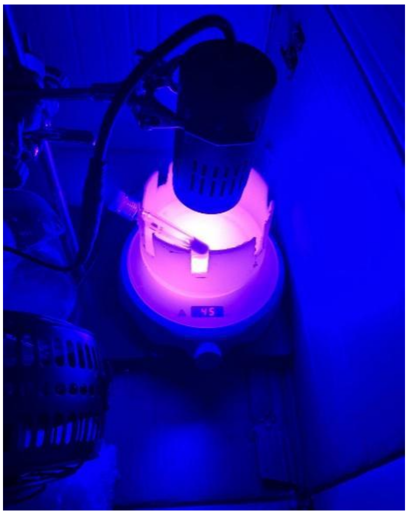

Figure S15. General reaction setup

General procedure for cross-coupling with ethers. *N*-Methoxy pyridinium methylsulfate (0.05 mmol), ether (3.8 mmol), $NaHCO_3$ (8.4 mg, 0.10 mmol), and Zr-PZDB (1.3 mg, 2.5 μmol, 5 mol% based on linker) were mixed in acetonitrile (0.5 mL) in a sealed test tube. The resulting mixture was stirred under blue LED irradiation (PR160L-427, 390-470 nm) at room temperature in a N_2 atmosphere for 24 hours. After that, the solvent was removed under vacuum, and the residue was subjected to column chromatography on silica gel to give products **3a-3s**, **3aj-3al**.

General procedure for cross-coupling with alcohols. *N*-Methoxy pyridinium methylsulfate (0.05 mmol), alcohol (3.8 mmol), $NaHCO_3$ (8.4 mg, 0.10 mmol), and **Zr-PZDB** (1.3 mg, 2.5 μmol, 5 mol% based on linker) were mixed in acetonitrile (0.5 mL)

in a sealed test tube. The resulting mixture was stirred under blue LED irradiation (PR160L-427, 390-470 nm) at room temperature in a N_2 atmosphere for 24 hours. After that, the solvent was removed under vacuum and the residue was subjected to column chromatography on silica gel to give products **3t-3y**.

General procedure for cross-coupling with inactivated alkanes. *N*-Methoxy pyridinium methylsulfate (0.05 mmol), alkane (3.8 mmol), $NaHCO_3$ (8.4 mg, 0.1 mmol), and **Zr-PZDB** (1.3 mg, 2.5 μmol, 5 mol% based on linker) were mixed in acetonitrile (0.5 mL) in a sealed test tube. The resulting mixture was stirred under blue LED irradiation (PR160L-427, 390-470 nm) at room temperature in a N_2 atmosphere for 24 hours. After that, the solvent was removed under vacuum, and the residue was subjected to column chromatography on silica gel to give products **3z-3ac**.

General procedure for cross-coupling with amides. *N*-Methoxy pyridinium methylsulfate (0.05 mmol), amide (3.8 mmol), $NaHCO_3$ (8.4 mg, 0.1 mmol), and **Zr-PZDB** (1.3 mg, 2.5 μmol, 5 mol% based on linker) were mixed in acetonitrile (0.5 mL) in a sealed test tube. The resulting mixture was stirred under blue LED irradiation (PR160L-427, 390-470 nm) at room temperature in a N_2 atmosphere for 24 hours. After that, the solvent was removed under vacuum, and the residue was subjected to column chromatography on silica gel to give products **3ad-3ag**.

General procedure for C-H alkylation of heteroarenes with aldehydes. *N*-Methoxy pyridinium methylsulfate (0.05 mmol), aldehyde (3.8 mmol), $NaHCO_3$ (8.4 mg, 0.1 mmol), and **Zr-PZDB** (1.3 mg, 2.5 μmol, 5 mol% based on linker) were mixed in acetonitrile (0.5 mL) in a sealed test tube. The resulting mixture was stirred under blue LED irradiation (PR160L-427, 390-470 nm) at room temperature in a N_2 atmosphere for 24 hours. After that, the solvent was removed under vacuum, and the residue was subjected to column chromatography on silica gel to give products **3ah** and **3ai**.

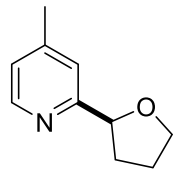

3a: Yield 80%. Colorless oil. ^{1}H NMR (400 MHz, Chloroform-*d*) δ 8.39 (d, *J* = 5.0 Hz, 1H), 7.27 (s, 1H), 6.99 (d, *J* = 4.7 Hz, 1H), 4.99 (t, *J* = 6.6 Hz, 1H), 4.17 – 4.06 (m, 1H), 4.03 – 3.92 (m, 1H), 2.42 (m, *J* = 10.8, 7.5, 3.9 Hz, 1H), 2.35 (s, 3H), 1.98 (m, *J*=10.7, 5.3 Hz, 3H). Characterization data matched that reported in the literature.[4]

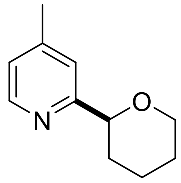

3b: Yield 88%. Colorless oil. ^{1}H NMR (400 MHz, Chloroform-*d*) δ 8.39 (d, *J* = 4.1 Hz, 1H), 7.30 (s, 1H), 7.01 (d, *J* = 4.7 Hz, 1H), 4.46 (dd, *J* = 11.2, 2.1 Hz, 1H), 4.23 – 4.11 (m, 1H), 3.65 (td, *J* = 11.5, 2.5 Hz, 1H), 2.36 (s, 3H), 2.07 (d, *J* = 13.0 Hz, 1H), 1.98 – 1.91 (m, 1H), 1.75 – 1.51 (m, 4H). Characterization data matched that reported in the literature.[4]

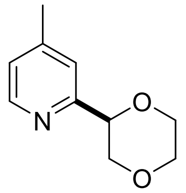

3c: Yield 74%. Colorless oil.^{1}H NMR (400 MHz, Chloroform-*d*) δ 8.40 (d, *J* = 4.8 Hz, 1H), 7.31 (s, 1H), 7.05 (d, *J* = 4.7 Hz, 1H), 4.75 (dd, *J* = 10.1, 2.8 Hz, 1H), 4.14 (dd, *J* = 11.6, 2.8 Hz, 1H), 4.02 – 3.88 (m, 2H), 3.84 – 3.66 (m, 2H), 3.51 (dd, *J* = 11.5, 10.2 Hz, 1H), 2.37 (s, 3H). Characterization data matched that reported in the literature.[4]

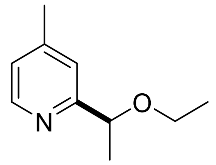

3d: Yield 60%. Colorless oil.^{1}H NMR (400 MHz, Chloroform-*d*) δ 8.38 (d, *J* = 5.0 Hz, 1H), 7.25 (s, 1H), 6.99 (d, *J* = 4.2 Hz, 1H), 4.50 (q, *J* = 6.6 Hz, 1H), 3.51 – 3.39 (m, 2H), 2.37 (s, 3H), 1.45 (d, *J* = 6.6 Hz, 3H), 1.22 (t, *J* = 7.0 Hz, 3H). Characterization data matched that reported in the literature.[4]

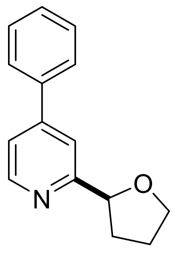

3e: Yield 76%. Colorless oil.^{1}H NMR (400 MHz, Chloroform-*d)* δ 8.59 (d, *J* = 5.1 Hz, 1H), 7.70 – 7.63 (m, 3H), 7.52 – 7.41 (m, 3H), 7.38 (dd, *J* = 5.1, 1.7 Hz, 1H), 5.09 (t, *J*=6.4 Hz, 1H), 4.19 – 4.10 (m, 1H) , 4.09 – 3.96 (m, 1H), 2.47 (m, 1H), 2.02 (m, 3H). Characterization data matched that reported in the literature.[5]

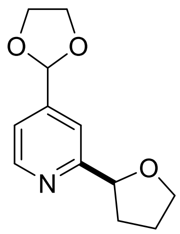

3f: Yield 70%. Colorless oil. ^{1}H NMR (400 MHz, Chloroform-*d*) δ 8.56 (dd, *J* = 5.0, 0.9 Hz, 1H), 7.54 (dd, *J* = 1.6, 0.8 Hz, 1H), 7.27 – 7.25 (m, 1H), 5.82 (s, 1H), 5.07 – 5.00 (t, *J* = 6.4 Hz, 1H), 4.16 – 4.02 (m, 5H), 4.02 – 3.95 (m, 1H), 2.50 – 2.36 (m, 1H), 2.06 – 1.93 (m, 3H). ^{13}C NMR (100 MHz, $CDCl_3$) δ 163.5, 149.3, 147.3, 119.4, 117.2, 102.1, 81.2, 69.1, 65.4, 65.3, 33.1, 25.8. HRMS (ESI) calcd for $C_{12}H_{15}NO_3$ $[M+H]^+$ 222.1052, found 222.1131.

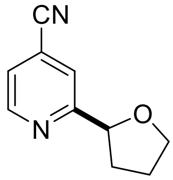

3g: Yield 42%. Colorless oil. ^{1}H NMR (400 MHz, Chloroform-*d*) δ 8.71 (d, *J* = 5.0 Hz, 1H), 7.71 (s, 1H), 7.38 (dd, *J* = 5.0, 1.6 Hz, 1H), 5.05 (t, *J*=7.2 Hz, 1H), 4.13 – 4.08 (m, 1H), 4.02 – 3.95 (m, 1H), 2.52 – 2.38 (m, 1H), 2.04 – 1.90 (m, 3H). Characterization data matched that reported in the literature.[5]

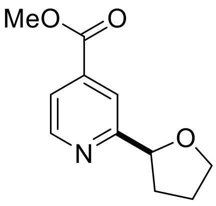

3h: Yield 50%. Colorless oil. ^{1}H NMR (400 MHz, Chloroform-*d*) δ 8.70 (s, 1H), 8.00 (s, 1H), 7.72 (d, *J* = 4.3 Hz, 1H), 5.08 (t, *J* = 6.4 Hz, 1H), 4.18 – 4.09 (m, 1H), 4.03 – 3.98 (m, 1H), 3.95 (s, 3H), 2.51 – 2.40 (m, 1H), 2.04 – 1.93 (m, 3H). ^{13}C NMR (100 MHz, $CDCl_3$) δ 165.7, 164.4, 149.7, 138.1, 121.3, 119.2, 81.0, 69.2, 52.7, 33.2, 25.8. HRMS (ESI) calcd for $C_{11}H_{14}NO_3$ $[M+H]^+$ 208.0968, found 208.0978.

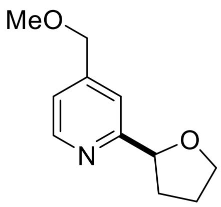

3i: Yield 56%. Colorless oil. ^{1}H NMR (400 MHz, Chloroform-*d*) δ 8.51 (d, *J* = 5.0 Hz, 1H), 7.39 (d, *J* = 1.6 Hz, 1H), 7.14 (dd, *J* = 5.1, 1.6 Hz, 1H), 5.02 (t, *J*=6.8 Hz, 1H), 4.47 (s, 2H), 4.16 – 4.07 (m, 1H), 4.01 – 3.96 (m, 1H), 3.43 (s, 3H), 2.48 – 2.39 (m, 1H), 2.04 – 1.93 (m, 3H). ^{13}C NMR (100 MHz, $CDCl_3$) δ 163.1, 149.1, 148.1, 120.2, 117.8, 81.3, 73.2, 69.1, 58.7, 33.1, 25.8. HRMS (ESI) calcd for $C_{11}H_{16}NO_2^+$ $[M+H]^+$ 194.1176, found 194.1183.

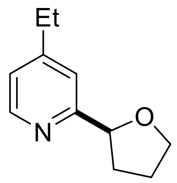

3j: Yield 56%. Colorless oil. ^{1}H NMR (400 MHz, Chloroform-*d*) δ 8.44 (d, *J* = 4.9 Hz, 1H), 7.36 (s, 1H), 7.08 (d, *J* = 4.7 Hz, 1H), 5.07 (t, *J* = 6.7 Hz, 1H), 4.17 – 4.09 (m, 1H), 4.03 – 3.95 (m, 1H), 2.69 (q, *J* = 7.6 Hz, 2H), 2.54 – 2.44 (m, 1H), 2.04 – 1.94(m, 3H), 1.26 (t, *J* = 7.6 Hz, 3H). Characterization data matched that reported in the literature.[6]

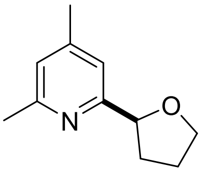

3k: Yield 32%. Colorless oil. ^{1}H NMR (400 MHz, Chloroform-*d*) δ 7.09 (s, 1H), 6.85 (s, 1H), 4.99 (t, *J* = 6.7 Hz, 1H), 4.17 – 4.04 (m, 1H), 3.98 – 3.93 (m, 1H), 2.50 (s, 3H), 2.46 – 2.40 (m, 1H), 2.30 (s, 3H), 2.00 – 1.88 (m, 3H). ^{13}C NMR (101 MHz, $CDCl_3$) δ 162.0, 157.0, 148.5, 122.7, 117.5, 81.1, 69.0, 33.3, 25.7, 23.9, 21.1. HRMS (ESI) calcd for $C_{11}H_{16}NO$ $[M+H]^+$ 178.1226, found 178.1239.

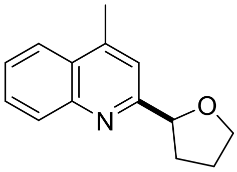

3l: Yield 70%. Colorless oil. ^{1}H NMR (400 MHz, Chloroform-*d*) δ 8.09 – 8.02 (m, 1H), 8.01 – 7.94 (m, 1H), 7.70 – 7.66 (m, 1H), 7.55 – 7.51 (m, 1H), 7.44 (s, 1H), 5.14 (t, *J* = 6.9 Hz, 1H), 4.25 – 4.12 (m, 1H), 4.08 – 3.99 (m, 1H), 2.71 (s, 3H), 2.58 – 2.41 (m, 1H), 2.12 – 2.00 (m, 3H). Characterization data matched that reported in the literature.[4]

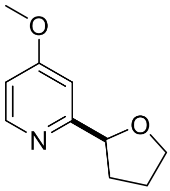

3m: quant. Colorless oil. ^{1}H NMR (400 MHz, Chloroform-*d*) δ 8.35 (d, *J* = 5.7 Hz, 1H), 6.99 (d, *J* = 2.6 Hz, 1H), 6.67 (dd, *J* = 5.7, 2.6 Hz, 1H), 5.02 – 4.92 (m, 1H), 4.19 – 4.05 (m, 1H), 4.01 – 3.91 (m, 1H), 3.85 (s, 3H), 2.48 – 2.37 (m, 1H), 2.06 – 1.90 (m, 3H). ^{13}C NMR (101 MHz, $CDCl_3$) δ 166.4, 165.0, 150.3, 108.4, 105.2, 81.1, 69.0, 55.1, 33.0, 25.7. HRMS (ESI) calcd for $C_{11}H_{14}NO_2^+$ $[M+H]^+$ 180.1019, found 180.1021.

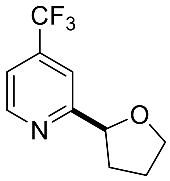

3n: Yield 52%. Colorless oil. ^{1}H NMR (400 MHz, Chloroform-*d*) δ 8.72 (d, *J* = 5.1 Hz, 1H), 7.70 (s, 1H), 7.38 (d, *J* = 4.7 Hz, 1H), 5.08 (t, *J*=6.8 Hz, 1H), 4.19 – 4.10 (m, 1H), 4.05 – 3.96 (m, 1H), 2.51 – 2.45

(m, 1H), 2.09 – 1.95 (m, 3H). Characterization data matched that reported in the literature.[7]

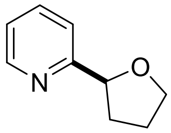

3o-C2: Yield 76% (C2:C4=1.8:1). Colorless oil. ^{1}H NMR (600 MHz, Chloroform-*d*) δ 8.55 (d, *J* = 4.9 Hz, 1H), 7.68 – 7.65 (m, 1H), 7.44 (d, *J* = 7.8 Hz, 1H), 7.15 (dd, *J* = 7.6, 5.0 Hz, 1H), 5.02 (t, *J* = 6.4 Hz, 1H), 4.13 – 4.08 (m, 1H), 4.00 – 3.96 (m, 1H), 2.45 – 2.37 (m, 1H), 2.03 – 1.96 (m, 3H). Characterization data matched that reported in the literature.[7]

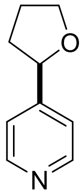

3o-C4: Yield 76% (C2:C4=1.8:1). Colorless oil. ^{1}H NMR (600 MHz, Chloroform-*d*) δ 8.72 – 8.45 (m, 2H), 7.25 (d, *J* = 5.3 Hz, 2H), 4.90 (t, *J* = 7.1 Hz, 1H), 4.10 – 4.06 (m, 1H), 3.98 – 3.94 (m, 1H), 2.40 – 2.35 (m, 1H), 2.03 – 1.94 (m, 2H), 1.79 – 1.73 (m, 1H). Characterization data matched that reported in the literature.[8]

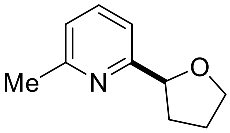

3p-C2: Yield 69% (C2:C4=1.5:1). Colorless oil. ^{1}H NMR (600 MHz, Chloroform-*d*) δ 7.55 (t, *J* = 7.7 Hz, 1H), 7.24 (d, *J* = 7.7 Hz, 1H), 7.01 (d, *J* = 7.6 Hz, 1H), 5.00 (t, *J* = 6.6 Hz, 1H), 4.24 – 4.05 (m, 1H), 4.03 – 3.90 (m, 1H), 2.54 (s, 3H), 2.47 – 2.38 (m, 1H), 2.00 – 1.91 (m, 3H). Characterization data matched that reported in the literature.[9]

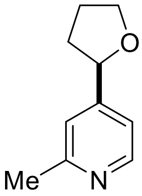

3p-C4: Yield 69% (C2:C4=1.5:1). Colorless oil. ^{1}H NMR (600 MHz, Chloroform-*d*) δ 8.43 – 8.42 (m, 1H), 7.13 (s, 1H), 7.04 (d, *J* = 4.7 Hz, 1H), 4.86 (t, *J* = 7.2 Hz, 1H), 4.11 – 4.05 (m, 1H), 3.97 – 3.94 (m, 1H), 2.55 (s, 3H), 2.39 – 2.33 (m, 1H), 2.03 – 1.96 (m, 2H), 1.79 – 1.74 (m, 1H). ^{13}C NMR (151 MHz, $CDCl_3$) δ 158.5, 153.2, 149.1, 120.1, 117.9, 79.3, 69.1, 34.4, 26.0, 24.6. HRMS (ESI) calcd for $C_{10}H_{14}NO_3^+$ [M+H]$^+$ 164.1070, found 164.1076.

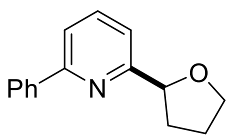

3q-C2: Yield 93% (C_2:C_4=2.9:1). Colorless oil. ^1H NMR (600 MHz, Chloroform-*d*) δ 8.00 (d, *J* = 7.6 Hz, 2H), 7.73 (t, *J* = 7.8 Hz, 1H), 7.59 (d, *J* = 5.8 Hz, 1H), 7.46 (t, *J* = 6.6 Hz, 2H), 7.40 (t, *J* = 7.2 Hz, 2H), 5.11 (t, *J* = 6.0 Hz, 1H), 4.15 – 4.11 (m, 1H), 4.03 – 3.99 (m, 1H), 2.53 – 2.39 (m, 1H), 2.22 – 2.09 (m, 1H), 2.00 (p, *J* = 7.0 Hz, 2H). Characterization data matched that reported in the literature.[10]

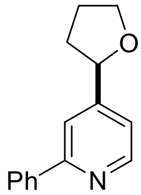

3q-C4: Yield 93% (C_2:C_4=2.9:1). Colorless oil. ^1H NMR (600 MHz, Chloroform-*d*) δ 8.63 (d, *J* = 5.1 Hz, 1H), 8.00 (d, *J* = 7.1 Hz, 2H), 7.70 (s, 1H), 7.47 (t, *J* = 7.6 Hz, 2H), 7.44 – 7.36 (m, 1H), 7.19 (d, *J* = 4.7 Hz, 1H), 4.97 (t, *J* = 7.2 Hz, 1H), 4.14 – 4.11 (m, 1H), 4.01 – 3.98 (m, 1H), 2.45 – 2.40 (m, 1H), 2.04 – 2.01 (m, 2H), 1.85 – 1.81 (m, 1H). Characterization data matched that reported in the literature.[11]

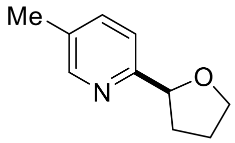

3r-C2: Yield 79% (C_2:C_4=1.5:1). Colorless oil. ^1H NMR (600 MHz, Chloroform-*d*) δ 8.37 (s, 1H), 7.48 (dd, *J* = 7.9, 2.3 Hz, 1H), 7.33 (d, *J* = 7.9 Hz, 1H), 4.99 (t, *J* = 6.2 Hz, 1H),4.12 – 4.08 (m, 1H), 4.00 – 3.90 (m, 1H), 2.37 – 2.42 (m, 1H), 2.32 (s, 3H), 2.01 – 1.94 (m, 3H). ^{13}C NMR (151 MHz, $CDCl_3$) δ 160.0, 149.4, 137.4, 131.6, 119.6, 81.3, 69.1, 33.2, 25.9, 18.3. HRMS (ESI) calcd for $C_{10}H_{14}NO_3^+$ $[M+H]^+$ 164.1070, found 164.1077.

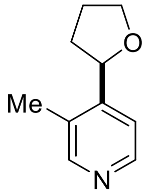

3r-C4: Yield 79% (C_2:C_4=1.5:1). Colorless oil. ^1H NMR (600 MHz, Chloroform-*d*) δ 8.42 (s, 1H), 8.33 (s, 1H), 7.36 (d, *J* = 5.0 Hz, 1H), 5.00 (t, *J* = 7.3 Hz, 1H), 4.16 – 4.12 (m, 1H), 3.97 – 3.93 (m, 1H), 2.45 – 2.36 (m, 1H), 2.26 (s, 3H), 2.05 – 1.95 (m, 2H), 1.65 – 1.59 (m, 1H). ^{13}C NMR (151 MHz, $CDCl_3$) δ 151.3, 150.6, 147.9, 129.5, 119.2, 77.0, 69.1, 32.9, 26.1, 16.2. HRMS (ESI) calcd for $C_{10}H_{14}NO_3^+$ $[M+H]^+$ 164.1070, found 164.1079.

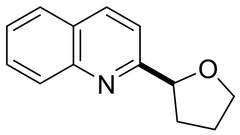

3s-C$_2$: Yield 89% (C$_2$:C$_4$=1.2:1). Colorless oil. ^{1}H NMR (600 MHz, Chloroform-*d*) δ 8.16 (d, *J* = 8.5 Hz, 1H), 8.06 (d, *J* = 8.5 Hz, 1H), 7.80 (d, *J* = 8.0 Hz, 1H), 7.71 – 7.68 (m, 1H), 7.61 (d, *J* = 8.5 Hz, 1H), 7.52 – 7.50 (m, 1H), 5.19 (t, *J* = 7.0 Hz, 1H), 4.19 – 4.16 (m, 1H), 4.07 – 4.03 (m, 1H), 2.56 – 2.50 (m, 1H), 2.11 – 2.02 (m, 3H). Characterization data matched that reported in the literature.[12]

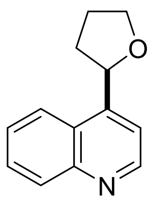

3s-C$_4$: Yield 89% (C$_2$:C$_4$=1.2:1). Colorless oil. ^{1}H NMR (600 MHz, Chloroform-*d*) δ 8.88 (d, *J* = 4.5 Hz, 1H), 8.13 (d, *J* = 8.3 Hz, 1H), 7.91 (d, *J* = 8.4 Hz, 1H), 7.72 – 7.69 (m, 1H), 7.58 – 7.53 (m, 2H), 5.62 (t, *J* = 7.1 Hz, 1H), 4.25 – 4.21 (m, 1H), 4.07 – 4.03 (m, 1H), 2.65 – 2.59 (m, 1H), 2.12 – 2.05 (m, 1H), 2.05 – 1.97 (m, 1H), 1.90 – 1.85 (m, 1H). Characterization data matched that reported in the literature.[13]

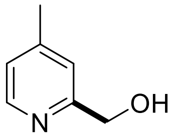

3t: Yield 70%. Colorless oil.^{1}H NMR (400 MHz, Chloroform-*d*) δ 8.42 (d, *J* = 5.2 Hz, 1H), 7.13 (s, 1H), 7.08 (d, *J* = 4.9 Hz, 1H), 4.75 (s, 2H), 2.39 (s, 3H). Characterization data matched that reported in the literature.[14]

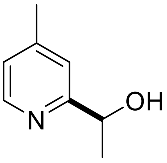

3u: Yield 44%. Colorless oil. ^{1}H NMR (400 MHz, Chloroform-*d*) δ 8.41 (d, *J* = 5.1 Hz, 1H), 7.16 (s, 1H), 7.09 (d, *J* = 6.0 Hz, 1H), 4.92 (q, *J* = 6.6 Hz, 1H), 2.41 (s, 3H), 1.52 (d, *J* = 6.6 Hz, 3H). Characterization data matched that reported in the literature.[15]

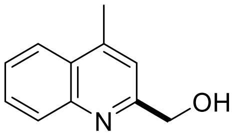

3v: Yield 40%. Colorless oil. ^{1}H NMR (400 MHz, Chloroform-*d*) δ 8.07 (d, *J* = 8.4 Hz, 1H), 7.99 (d, *J* = 8.3 Hz, 1H), 7.77 – 7.68 (m, 1H), 7.60 – 7.52 (m, 1H), 7.12 (s, 1H), 4.87 (s, 2H), 2.70 (s, 3H). Characterization data matched that reported in the literature.[16]

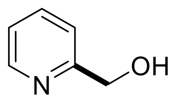

3w-C_2: Yield 76% (C_2:C_4=5.4:1). Colorless oil. ^{1}H NMR (600 MHz, Chloroform-*d*) δ 8.52 (d, *J* = 5.1 Hz, 1H), 7.71 – 7.56 (m, 1H), 7.25 (d, *J* = 6.8 Hz, 1H), 7.18 (dd, *J* = 7.9, 4.6 Hz, 1H), 4.74 (s, 2H), 3.35 (br, 1H). Characterization data matched that reported in the literature.[14]

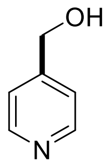

3w-C_4: Yield 76% (C_2:C_4=5.4:1). Colorless oil. ^{1}H NMR (600 MHz, Chloroform-*d*) δ 8.52 (d, *J* = 5.1 Hz, 2H), 7.30 (d, *J* = 5.0 Hz, 2H), 4.75 (s, 2H), 2.56 (s, br). Characterization data matched that reported in the literature.[14]

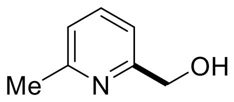

3x-C_2: Yield 67% (C_2:C_4=1.2:1). Colorless oil. ^{1}H NMR (400 MHz, Chloroform-*d*) δ 7.56 (t, *J* = 7.7 Hz, 1H), 7.04 (dd, *J* = 10.6, 7.7 Hz, 2H), 4.72 (s, 2H), 2.56 (s, 3H). Characterization data matched that reported in the literature.[14]

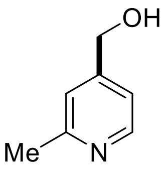

3x-C_4: Yield 67% (C_2:C_4=1.2:1). Colorless oil. ^{1}H NMR (400 MHz, Chloroform-*d*) δ 8.41 (d, *J* = 5.2 Hz, 1H), 7.17 (s, 1H), 7.09 (d, *J* = 5.1 Hz, 1H), 4.71 (s, 2H), 2.54 (s, 3H). Characterization data matched that reported in the literature.[14]

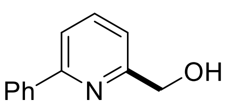

3y-C_2: Yield 58% (C_2:C_4=1.4:1). Colorless oil. ^{1}H NMR (400 MHz, Chloroform-*d*) δ 8.02 (d, *J* = 6.9 Hz, 2H), 7.76 (t, *J* = 7.7 Hz, 1H), 7.65 (d, *J* = 7.8 Hz, 1H), 7.55 – 7.46 (m, 2H), 7.46 – 7.38 (m, 1H), 7.16 (d, *J* = 7.6 Hz, 1H), 4.82 (s, 2H), 4.18 (s, 1H). Characterization data matched that reported in the literature.[14]

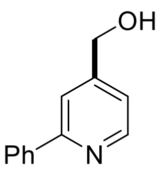

3y-C_4: Yield 58% (C_2:C_4=1.4:1). Colorless oil. ^{1}H NMR (600 MHz, Chloroform-*d*) δ 8.60 (d, *J* = 5.0 Hz, 1H), 7.95 (d, *J* = 7.3 Hz, 2H), 7.69 (s, 1H), 7.46 (t, *J* = 7.3 Hz, 2H), 7.43 – 7.38 (m, 1H), 7.19 (d, *J* = 4.8 Hz, 1H), 4.76 (s, 2H). Characterization data matched that reported in the literature.[14]

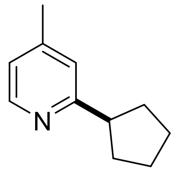

3z: Yield 56%. Colorless oil. ^{1}H NMR (400 MHz, Chloroform-*d*) δ 8.40 (d, *J* = 5.1 Hz, 1H), 7.01 (s, 1H), 6.96 – 6.89 (m, 1H), 3.19 – 3.11 (m, 1H), 2.32 (s, 3H), 2.10 – 1.76 (m, 8H). Characterization data matched that reported in the literature.[17]

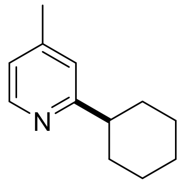

3aa: Yield 62%. Colorless oil. ^{1}H NMR (400 MHz, Chloroform-*d)* δ 8.44 (d, *J* = 5.3 Hz, 1H), 7.14 (s, 1H), 7.12 (d, *J* = 4.8 Hz, 1H), 2.96 – 2.84 (m, 1H), 2.43 (s, 3H), 1.99 (d, *J* = 13.8 Hz, 2H), 1.87 (d, *J* = 12.8 Hz, 2H), 1.77 (d, *J* = 15.5 Hz, 1H), 1.61 – 1.39 (m, 5H). Characterization data matched that reported in the literature.[17]

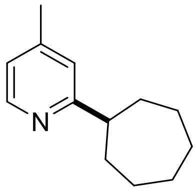

3ab: Yield 62%. Colorless oil. ^{1}H NMR (400 MHz, Chloroform-*d*) δ 8.46 (d, *J* = 5.2 Hz, 1H), 7.02 (s, 1H), 6.98 (d, *J* = 5.3 Hz, 1H), 2.96 – 2.86 (m, 1H), 2.36 (s, 3H), 2.00 – 1.80 (m, 12H). Characterization data matched that reported in the literature.[17]

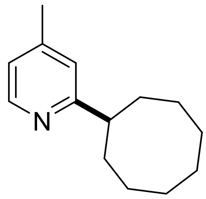

3ac: Yield 64%. Colorless oil. ^{1}H NMR (400 MHz, Chloroform-*d*) δ 8.35 (d, *J* = 5.0 Hz, 1H), 6.94 (s, 1H), 6.89 (d, *J* = 5.0 Hz, 1H), 2.94 – 2.87 (m, 1H), 2.31 (s, 3H), 1.91 – 1.73 (m, 8H), 1.72 – 1.60 (m, 6H). Characterization data matched that reported in the literature.[18]

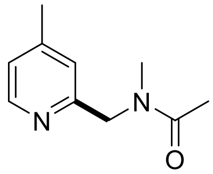

3ad: Yield 70%. Colorless oil. ^{1}H NMR (400 MHz, Chloroform-*d*) δ 8.38 (dd, *J* = 21.7, 5.0 Hz, 1H), 7.09 – 6.93 (m, 2H), 4.61 (d, *J* = 30.1 Hz, 2H), 3.00 (s, 3H), 2.33 (d, *J* = 13.4 Hz, 3H), 2.14 (d, *J* = 11.8 Hz, 3H). ^{13}C NMR (100 MHz, $CDCl_3$) δ 170.9, 170.7, 157.1, 156.5, 149.6, 148.8, 148.5, 148.1, 123.5, 123.4, 123.0, 121.0, 56.1, 52.6, 38.0, 35.2, 21.8, 21.5, 21.5, 21.0. HRMS (ESI) calcd for $C_{10}H_{15}N_2O^+$ $[M+H]^+$ 179.1179, found 179.1179.

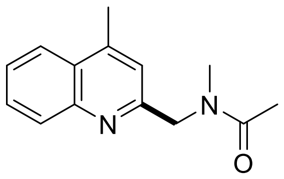

3ae: quant. Colorless oil. ^{1}H NMR (400 MHz, Chloroform-*d*) δ 8.10 – 7.93 (m, 2H), 7.75 – 7.68 (m, 1H), 7.60 – 7.53 (m, 1H), 7.25 – 7.09 (m, 1H), 4.80 (d, *J* = 36.9 Hz, 2H), 3.05 (d, *J* = 2.3 Hz, 3H), 2.70 (dd, *J* = 16.5, 1.0 Hz, 3H), 2.20 (d, *J* = 7.0 Hz, 3H). Characterization data matched that reported in the literature.[7]

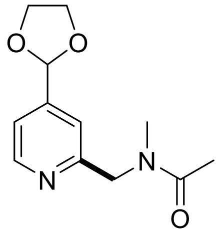

3af: Yield 61%. Colorless oil.^{1}H NMR (400 MHz, Chloroform-*d*) δ 8.55 (dd, *J* = 21.4, 4.9 Hz, 1H), 7.30 (d, *J* = 9.0 Hz, 1H), 7.26 (d, *J* = 3.8 Hz, 1H), 5.75 (s, 1H), 4.66 (d, *J* = 36.0 Hz, 2H), 4.16 – 3.97 (m, 4H), 3.04 – 2.96 (m, 3H), 2.14 (d, *J* = 8.3 Hz, 3H).^{13}C NMR (100 MHz, $CDCl_3$) δ 171.3, 170.8, 157.7, 157.1, 150.1, 149.3, 147.9, 147.5, 120.0, 119.7, 119.5, 117.6, 101.8, 101.7, 65.3, 56.1, 52.6, 36.3, 34.1, 21.7, 21.5. HRMS (ESI) calcd for $C_{12}H_{17}N_2O_3^+$ $[M+H]^+$ 237.1234, found 237.1235.

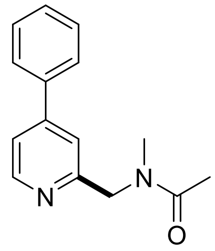

3ag: Yield 80%. Colorless oil.^{1}H NMR (400 MHz, Chloroform-*d*) δ 8.59 (dd, *J* = 22.7, 5.1 Hz, 1H), 7.63 – 7.59 (m, 2H), 7.52 – 7.32 (m, 5H), 4.72 (d, *J* = 33.3 Hz, 2H), 3.06 (d, *J* = 13.2 Hz, 3H), 2.18 (d, *J* = 3.5 Hz, 3H). ^{13}C NMR (101 MHz, $CDCl_3$) δ 171.4, 170.9, 157.9, 157.3, 150.3, 149.7, 149.5, 149.3, 138.0, 137.7, 129.3, 129.2, 129.0, 129.0, 127.1, 127.0, 120.6, 120.4, 120.2, 118.0, 56.3, 52.8, 36.4, 34.2, 21.7, 21.5. HRMS (ESI) calcd for $C_{15}H_{17}N_2O^+$ $[M+H]^+$ 241.1335, found 241.1339.

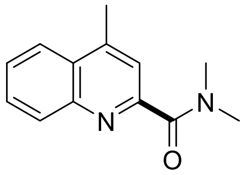

3ah: quant. Colorless oil. ^{1}H NMR (400 MHz, Chloroform-*d*) δ 8.09 (d, *J* = 8.4 Hz, 1H), 8.00 (d, *J* = 8.3 Hz, 1H), 7.78 – 7.68 (m, 1H), 7.66 – 7.56 (m, 1H), 7.52 (s, 1H), 3.17 (s, 3H), 3.14 (s, 3H), 2.72 (s, 3H). Characterization data matched that reported in the literature.[19]

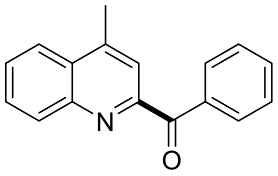

3ai: Yield 60%. Colorless oil. ^{1}H NMR (400 MHz, Chloroform-*d*) δ 8.31 – 8.16 (m, 3H), 8.12 – 8.05 (m, 1H), 7.94 (s, 1H), 7.80 – 7.75 (m, 1H), 7.70 – 7.66 (m, 1H), 7.65 – 7.59 (m, 1H), 7.51 (t, *J* = 7.6 Hz, 2H), 2.87 – 2.79 (m, 3H). Characterization data matched that reported in the literature.[20]

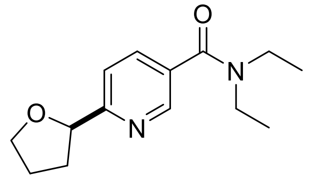

3aj: Yield 48%. Colorless oil. ^{1}H NMR (400 MHz, Chloroform-*d*) δ 8.57 (d, *J* = 1.5 Hz, 1H), 7.71 (dd, *J* = 8.0, 2.2 Hz, 1H), 7.49 (d, *J* = 8.0 Hz, 1H), 5.04 (t, *J* = 6.8, 1H), 4.14 – 4.08 (m, 1H), 4.03 – 3.96 (m, 1H), 3.59 – 3.25 (m, 4H), 2.49 – 2.40 (m, 1H), 2.05 – 1.93 (m, 3H), 1.35 – 1.17 (m, 6H). ^{13}C NMR (101 MHz, $CDCl_3$) δ 168.8, 164.0, 146.6, 135.0, 131.3, 119.5, 81.1, 69.2, 33.1, 25.7. HRMS (ESI) calcd for $C_{14}H_{21}N_2O_2^+$ $[M+H]^+$ 249.1598, found 249.1605.

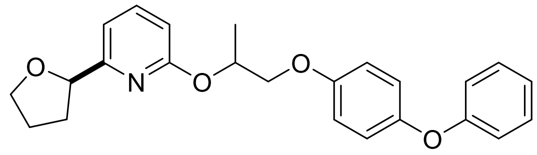

3ak-C2: Yield 78% (C_2:C_4=1.8:1). Colorless oil.^{1}H NMR (400 MHz, Chloroform-*d*) δ 7.54 (t, *J* = 7.8 Hz, 1H), 7.32 – 7.26 (m, 2H), 7.04 (t, *J* = 7.4 Hz, 1H), 7.00 – 6.93 (m, 7H), 6.60 (d, *J* = 8.2 Hz, 1H), 5.61 – 5.53 (m, 1H), 4.92 (t, *J* = 6.6 Hz, 1H), 4.21 (dd, *J* = 9.9, 5.2 Hz, 1H), 4.11 – 4.01 (m, 2H), 3.99 – 3.90 (m, 1H), 2.37 – 2.82 (m, 1H), 2.09 – 1.92 (m, 3H), 1.48 – 1.46 (m, 3H).^{13}C NMR (100 MHz, $CDCl_3$) δ 162.5, 160.5, 158.4, 155.2, 150.2, 139.1, 129.6, 122.4, 120.7, 117.6, 115.8, 112.6, 109.6, 81.0, 71.0, 71.0, 69.0, 32.5, 25.7, 17.1. HRMS (ESI) calcd for $C_{24}H_{26}NO_4^+$ $[M+H]^+$ 392.1856, found 392.1859.

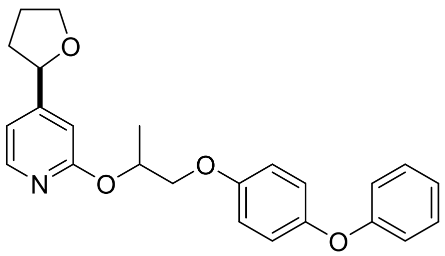

3ak-C4: Yield 78% (C_2:C_4=1.8:1). Colorless oil. ^{1}H NMR (400 MHz, Chloroform-*d*) δ 8.08 (d, *J* = 5.2 Hz, 1H), 7.29 (t, *J* = 7.9 Hz, 2H), 7.03 (t, *J* = 7.3 Hz, 1H), 6.94 (q, *J* = 9.1 Hz, 6H), 6.82 (d, *J* = 5.1 Hz, 1H), 6.72 (s, 1H), 5.61 – 5,53 (m, 1H), 4.85 (t,

J = 7.0 Hz, 1H), 4.20 – 4.16 (m, 1H), 4.11 – 4.00 (m, 2H), 3.94 (q, *J* = 7.1 Hz, 1H), 2.38 – 2.28 (m, 1H), 2.01 – 1.94 (m, 2H), 1.81 – 1.73 (m, 1H), 1.47 (dd, *J* = 6.4, 2.7 Hz, 3H). ^{13}C NMR (100 MHz, $CDCl_3$) δ 163.5, 158.5, 156.0, 155.2, 150.2, 146.7, 129.6, 122.4, 120.7, 117.6, 115.8, 114.1, 107.9, 79.1, 71.1, 69.3, 68.9, 34.1, 25.8, 17.0. HRMS (ESI) calcd for $C_{24}H_{26}NO_4^+$ [M+H]$^+$ 392.1856, found 392.1861.

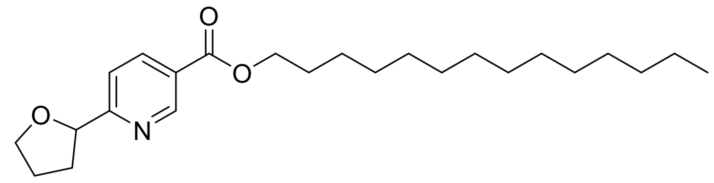

3al: Yield 48%. Colorless oil. ^{1}H NMR (400 MHz, Chloroform-*d*) δ 9.13 (s, 1H), 8.26 (dd, *J* = 8.2, 1.9 Hz, 1H), 7.54 (d, *J* = 8.2 Hz, 1H), 5.13 – 5.02 (m, 1H), 4.33 (t, *J* = 6.7 Hz, 2H), 4.11 (q, *J* = 6.5 Hz, 1H), 4.03 – 3.96 (m, 1H), 2.50 – 2.42 (m, 1H), 2.04 – 1.89 (m, 3H), 1.78 – 1.72 (m, 3H), 1.44 – 1.40 (m, 2H), 1.37 – 1.24 (m, 19H), 0.87 (t, *J* = 7.0 Hz, 3H). ^{13}C NMR (100 MHz, $CDCl_3$) δ 167.6, 165.4, 150.3, 137.7, 124.7, 119.2, 81.1, 69.2, 65.4, 33.1, 31.9, 29.7, 29.6, 29.6, 29.5, 29.5, 29.3, 29.2, 28.6, 26.0, 25.7, 22.7, 14.1. HRMS (ESI) calcd for $C_{24}H_{40}NO_3^+$ [M+H]$^+$ 390.3003, found 390.3008.

3.2 Zr-PZDB Catalyzed Cross-Coupling of Aryl Sulfonium Salts with Heterocycles

3.2.1 Synthesis of Aryl Sulfonium Salts

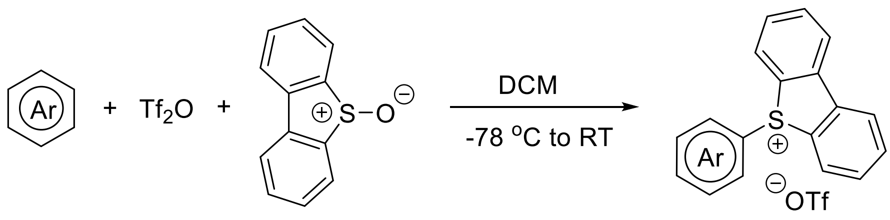

General procedures. Tf_2O (403 μL, 2.4 mmol) was added dropwise to a solution of the corresponding arene (2.0 mmol) and dibenzothiophene S-oxide (0.44 g, 2.2 mmol) in CH_2Cl_2 (0.1 M) at -78 °C under N_2. The resultant solution was stirred at this temperature for 15 minutes before warming to room temperature. After stirring for 1 h, TLC analysis showed the arene starting material was consumed completely, at which

point the solvent was removed in vacuo. The sulfonium salt was then precipitated after the addition of Et_2O. The crude product was washed with Et_2O for three times. The product was purified by recrystallization from DCM/Et_2O.

3.2.2. Optimization of Reaction Conditions

Table S4. Optimization of reaction conditions for pyrrole arylation

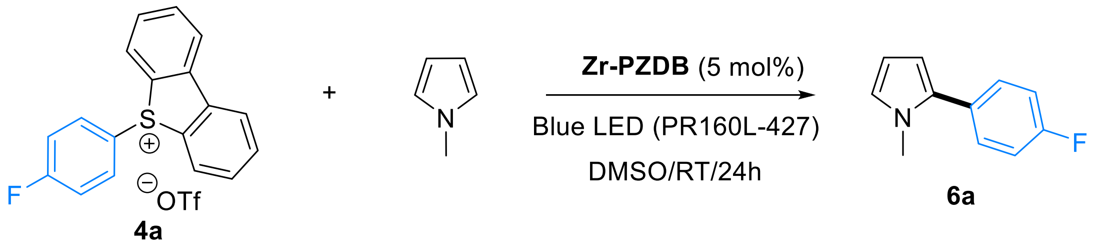

Entry	Variation from standard conditions[a]	Yield (%)[b]
1	no	87
2	PZDB-Me instead of **Zr-PZDB**	12
3	PZDB-H instead of **Zr-PZDB**	39
4	$ZrCl_4$ and PZDB-H instead of **Zr-PZDB**	45
5	no light	NR
6	No Zr-PZDB	Trace
7	PZDB-H@C instead of **Zr-PZDB**	36%

[a]Standard conditions: sulfonium salt (0.2 mmol), **Zr-PZDB** (10.0 μmol, 5 mol% based on the linker), *N*-methyl pyrrole (8.0 mmol), DMSO (1.0 mL), N_2, r.t., PR160L-427 (390-470 nm), 24 h; @C = loaded into activated carbons. [b]Yields of isolated **6a**.

3.2.3. Coupling Reactions

General procedure for cross-coupling of aryl sulfonium salts and heterocycles. Dibenzothiophenium salt (0.2 mmol), heterocycle (8.0 mmol), and **Zr-PZDB** (5.3 mg, 10.0 μmol, 5.0 mol% based on the linker) were mixed in DMSO (1.0 mL) in a sealed test tube. The resulting mixture was stirred under blue LED irradiation (PR160L-427, 390-470 nm) at room temperature in a N_2 atmosphere for 24 hours. After that, the reaction was quenched with aqueous saturated $NaHCO_3$ and diluted with EtOAc. The organic layer was washed with brine, dried with Na_2SO_4, filtered, and concentrated in

vacuo. The residue was subjected to column chromatography on silica gel to give products **6a-6l**.

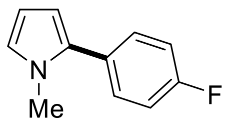

6a: Yield 87%. White solid. ^{1}H NMR (600 MHz, Chloroform-d) δ 7.36 (dd, *J* = 8.5, 5.6 Hz, 2H), 7.09 (t, *J* = 8.7 Hz, 2H), 6.72 – 6.71 (m, 1H), 6.20 – 6.19 (m, 2H), 3.64 (s, 3H). Characterization data matched that reported in the literature.[21]

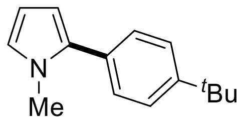

6b: Yield 70%. Colorless liquid. ^{1}H NMR (600 MHz, Chloroform-d) δ 7.44 (d, *J* = 8.4 Hz, 2H), 7.36 (d, *J* = 8.4 Hz, 2H), 6.73 – 6.72 (t, *J* = 2.3 Hz, 1H), 6.23 – 6.21 (m, 2H), 3.68 (s, 3H), 1.38 (s, 9H). Characterization data matched that reported in the literature.[22]

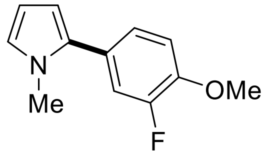

6c: Yield 83%. White solid. ^{1}H NMR (600 MHz, Chloroform-d) δ 7.17 – 7.07 (m, 2H), 6.99 (t, *J* = 8.6 Hz, 1H), 6.70 (t, *J* = 2.2 Hz, 1H), 6.20 – 6.14 (m, 2H), 3.92 (s, 3H), 3.64 (s, 3H). Characterization data matched that reported in the literature.[23]

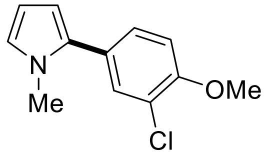

6d: Yield 88%. White solid. ^{1}H NMR (600 MHz, Chloroform-d) δ 7.41 (d, *J* = 2.2 Hz, 1H), 7.27 – 7.24 (m, 1H), 6.96 (d, *J* = 8.5 Hz, 1H), 6.70 (t, *J* = 2.3 Hz, 1H), 6.32 – 6.06 (m, 2H), 3.94 (s, 3H), 3.64 (s, 3H). Characterization data matched that reported in the literature.[23]

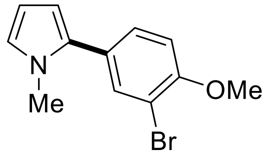

6e: Yield 69%. White solid. ^{1}H NMR (600 MHz, Chloroform-d) δ 7.59 (d, *J* = 2.2 Hz, 1H), 7.30 (dd, *J* = 8.4, 2.2 Hz, 1H), 6.94 (d, *J* = 8.5 Hz, 1H), 6.70 (t, *J* = 2.3 Hz, 1H), 6.18 (m, 2H), 3.93 (s, 3H), 3.63 (s, 3H). Characterization data matched that reported in the literature.[23]

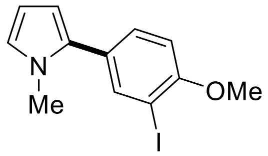

6f: Yield 51%. Colorless oil. ^{1}H NMR (600 MHz, Chloroform-d) δ 7.83 (d, *J* = 2.2 Hz, 1H), 7.34 (dd, *J* = 8.4, 2.2 Hz, 1H), 6.86 (d, *J* = 8.4 Hz, 1H), 6.69 (dd, *J* = 2.6, 1.9 Hz, 1H), 6.24 – 6.09 (m, 2H), 3.91 (s, 3H), 3.63 (s, 3H). Characterization data matched that reported in the literature.[23]

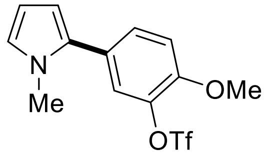

6g: Yield 61%. Colorless oil. ^{1}H NMR (400 MHz, Chloroform-d) δ 7.36 (dd, *J* = 8.5, 2.1 Hz, 1H), 7.26 (d, *J* = 2.1 Hz, 1H), 7.08 (d, *J* = 8.6 Hz, 1H), 6.73 (t, *J* = 2.3 Hz, 1H), 6.20 (d, *J* = 2.3 Hz, 2H), 3.96 (s, 3H), 3.64 (s, 3H). Characterization data matched that reported in the literature.[23]

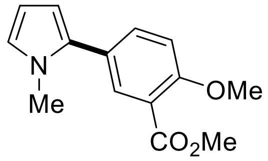

6h: Yield 82%. Yellow solid. ^{1}H NMR (600 MHz, Chloroform-d) δ 7.82 (d, *J* = 2.4 Hz, 1H), 7.48 (dd, *J* = 8.6, 2.4 Hz, 1H), 7.01 (d, *J* = 8.6 Hz, 1H), 6.69 (t, *J* = 2.2 Hz, 1H), 6.18 (d, *J* = 2.2 Hz, 2H), 3.93 (s, 3H), 3.89 (s, 3H), 3.62 (s, 3H). Characterization data matched that reported in the literature.[23]

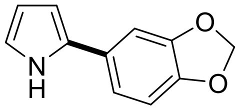

6i: Yield 52%. Red solid. ^{1}H NMR (600 MHz, Chloroform-d) δ 8.31 (s, 1H), 6.97 (d, *J* = 1.8 Hz, 1H), 6.93 (dd, *J* = 8.1, 1.8 Hz, 1H), 6.86 – 6.79 (m, 2H), 6.40 – 6.39 (m, 1H), 6.28 – 6.27 (m, 1H), 5.97 (s, 2H). ^{13}C NMR (151 MHz, $CDCl_3$) δ 148.3, 146.3, 132.3, 127.6, 118.5, 117.4, 110.1, 108.8, 105.5, 105.3, 101.2. HRMS (ESI) calcd for $C_{11}H_{10}NO_2^+$ $[M+H]^+$ 188.0706, found 188.0672.

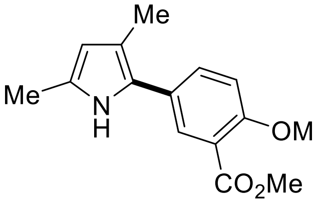

6j: Yield 65%. Red solid. ^{1}H NMR (600 MHz, Chloroform-d) δ 7.93 (s, 1H), 7.80 (t, *J* = 2.1 Hz, 1H), 7.50 (dt, *J* = 8.6, 2.1 Hz, 1H), 6.99 (dd, *J* = 8.7, 1.7 Hz, 1H), 5.81 (s, 1H), 3.91 – 3.90 (m, 6H), 2.28 (s, 3H), 2.20 (s, 3H). ^{13}C NMR (151 MHz, $CDCl_3$) δ 167.0, 157.1, 131.3, 129.2, 127.5, 126.6, 125.6, 120.3,

116.2, 112.6, 110.2, 56.3, 52.2, 13.1, 12.4. HRMS (ESI) calcd for $C_{15}H_{17}NNaO_3^+$ $[M+Na]^+$ 282.1101, found 282.1111.

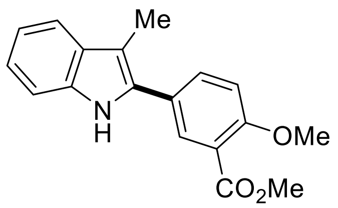

6k: Yield 63%. White solid. ^{1}H NMR (600 MHz, Chloroform-d) δ 8.19 – 8.05 (m, 1H), 8.00 (d, *J* = 2.4 Hz, 1H), 7.68 (dd, *J* = 8.6, 2.4 Hz, 1H), 7.59 (dd, *J* = 7.9, 1.1 Hz, 1H), 7.36 (dd, *J* = 8.0, 0.9 Hz, 1H), 7.22 – 7.19 (m, 1H), 7.16 – 7.13 (m, 1H), 7.08 (d, *J* = 8.6 Hz, 1H), 3.95 (s, 3H), 3.93 (s, 3H), 2.44 (s, 3H). Characterization data matched that reported in the literature.[23]

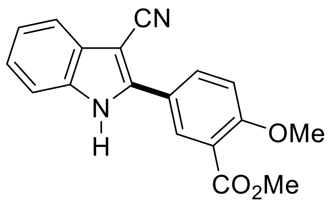

6l: Yield 83%. White solid. ^{1}H NMR (600 MHz, Chloroform-*d*) δ 9.40 (s, 1H), 8.18 (d, *J* = 2.6 Hz, 1H), 8.11 (dd, *J* = 8.7, 2.5 Hz, 1H), 7.74 (d, *J* = 7.6 Hz, 1H), 7.54 – 7.40 (m, 1H), 7.34 – 7.27 (m, 2H), 7.04 (d, *J* = 8.7 Hz, 1H), 3.92 (s, 3H), 3.84 (s, 3H). ^{13}C NMR (151 MHz, $CDCl_3$) δ 166.4, 160.2, 143.8, 135.2, 132.6, 130.0, 128.9, 124.5, 122.6, 121.8, 120.6, 119.6, 117.0, 113.1, 111.9, 83.6, 56.3, 52.6. HRMS (ESI) calcd for $C_{18}H_{14}N_2NaO_3^+$ $[M+Na]^+$ 329.0897, found 329.0905.

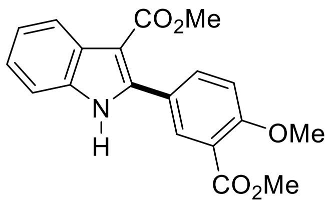

6m: Yield 78%. White solid. ^{1}H NMR (600 MHz, Chloroform-*d*) δ 9.26 (s, 1H), 8.21 – 8.11 (m, 1H), 8.00 (d, *J* = 2.4 Hz, 1H), 7.81 (dd, *J* = 8.7, 2.4 Hz, 1H), 7.47 – 7.39 (m, 1H), 7.27 (dd, *J* = 6.1, 3.2 Hz, 2H), 6.91 (d, *J* = 8.7 Hz, 1H), 3.88 (s, 3H), 3.84 (s, 3H), 3.77 (s, 3H). ^{13}C NMR (151 MHz, $CDCl_3$) δ 166.4, 166.0, 159.8, 143.5, 135.7, 135.4, 132.6, 127.6, 124.0, 123.4, 122.2, 119.2, 111.7, 111.3, 104.3, 56.0, 52.4, 51.0. HRMS (ESI) calcd for $C_{19}H_{17}NNaO_5^+$ $[M+Na]^+$ 362.0999, found 362.1008.

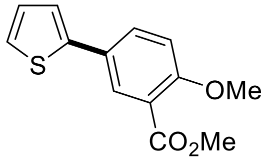

6n: Yield 57%. White solid. ^{1}H NMR (600 MHz, Chloroform-d) δ 8.03 (d, *J* = 2.5 Hz, 1H), 7.69 (dd, *J* = 8.7, 2.5 Hz, 1H), 7.26 – 7.22 (m, 2H), 7.06 (dd, *J* = 4.9, 3.7 Hz, 1H), 6.99 (d, *J* = 8.8

Hz, 1H), 3.93 (s, 3H), 3.92 (s, 3H). Characterization data matched that reported in the literature.[23]

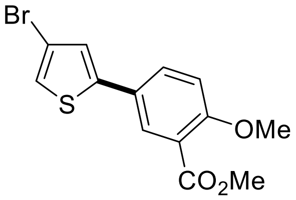

6o: Yield 82%. White solid. ^{1}H NMR (600 MHz, Chloroform-*d*) δ 8.05 (d, *J* = 2.5 Hz, 1H), 7.76 (dd, *J* = 8.7, 2.4 Hz, 1H), 7.26 (d, *J* = 5.3 Hz, 1H), 7.05 (d, *J* = 5.1 Hz, 1H), 7.03 (d, *J* = 1.7 Hz, 1H), 3.95 (s, 3H), 3.91 (s, 3H). ^{13}C NMR (151 MHz, $CDCl_3$) δ 166.3, 159.1, 137.0, 134.2, 132.6, 131.7, 125.1, 125.0, 120.2, 112.2, 107.8, 56.3, 52.3. HRMS (ESI) calcd for $C_{13}H_{11}BrNaO_3S^+$ $[M+Na]^+$ 348.9504, found 348.9514.

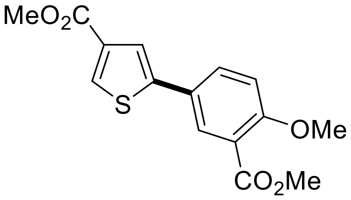

6p: Yield 69%. White solid. ^{1}H NMR (600 MHz, Chloroform-*d*) δ 7.94 (d, *J* = 2.4 Hz, 1H), 7.63 (dd, *J* = 8.6, 2.4 Hz, 1H), 7.49 (d, *J* = 5.4 Hz, 1H), 7.22 (d, *J* = 5.5 Hz, 1H), 7.01 (d, *J* = 8.7 Hz, 1H), 3.94 (s, 3H), 3.88 (s, 3H), 3.74 (s, 3H). ^{13}C NMR (151 MHz, $CDCl_3$) δ 166.3, 163.7, 159.5, 150.0, 135.1, 133.3, 130.1, 127.8, 125.3, 124.1, 119.5, 111.6, 56.3, 52.2, 51.7. HRMS (ESI) calcd for $C_{15}H_{14}NaO_5S^+$ $[M+Na]^+$ 329.0454, found 329.0464.

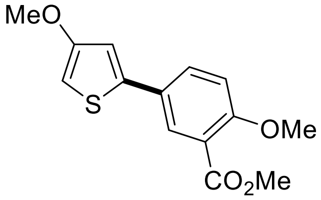

6q: Yield 52%. White solid. ^{1}H NMR (600 MHz, Chloroform-*d*) δ 8.11 (d, *J* = 2.5 Hz, 1H), 7.85 (dd, *J* = 8.8, 2.4 Hz, 1H), 7.13 (d, *J* = 5.5 Hz, 1H), 6.98 (d, *J* = 8.8 Hz, 1H), 6.92 (d, *J* = 5.5 Hz, 1H), 3.92 (s, 3H), 3.91 (s, 3H), 3.91 (s, 3H). ^{13}C NMR (151 MHz, $CDCl_3$) δ 166.8, 157.7, 153.5, 132.0, 130.2, 126.0, 121.9, 120.2, 119.0, 117.5, 112.4, 58.9, 56.3, 52.2. HRMS (ESI) calcd for $C_{14}H_{14}NaO_4S^+$ $[M+Na]^+$ 301.0505, found 301.0514.

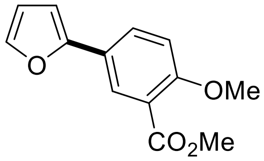

6r: Yield 91%. White solid. ^{1}H NMR (600 MHz, Chloroform-*d*) δ 8.09 (d, *J* = 1.6 Hz, 1H), 7.84 – 7.70 (m, 1H), 7.44 (d, *J* = 1.7 Hz, 1H), 6.99 (dd, *J* = 8.6, 1.4 Hz, 1H), 6.61 – 6.52 (m, 1H), 6.45

(dd, J = 3.4, 1.7 Hz, 1H), 3.92 (s, 3H), 3.91 (s, 3H). Characterization data matched that reported in the literature.[23]

3.3 Catalyst Recycle and Recovery Experiments

Recycle experiments for coupling of 1a/THF. **1a** (23.6 mg, 0.1 mmol), THF (0.62 mL, 7.6 mmol), $NaHCO_3$ (16.8 mg, 0.2 mmol), and **Zr-PZDB** (2.6 mg, 5.0 μmol, 5 mol% based on the linker) were mixed in acetonitrile (1.0 mL) in a sealed test tube. The resultant mixture was stirred under blue LED irradiation (PR160L-427, 390-470 nm) at room temperature in a N_2 atmosphere for 24 hours. After the reaction, the **Zr-PZDB** catalyst was recovered via centrifugation in a glovebox, washed with MeCN (1.0 mL × 3), and used direct in subsequent cycles of reactions. The reaction supernatant was then subjected to ^{1}H NMR analysis. This procedure was repeated three times.

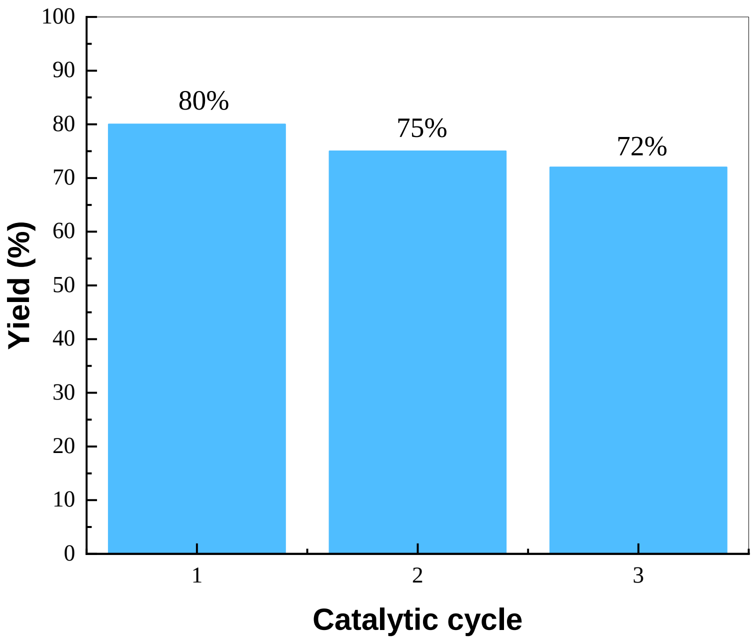

Figure S16. Yields of **3a** with recovered **Zr-PZDB** in three consecutive runs

Recycle experiments for coupling of 4a/pyrrole. **4a** (85.6 mg，0.2 mmol), *N*-methyl pyrrole (0.71 mL, 8.0 mmol), and **Zr-PZDB** (5.3 mg, 10.0 µmol, 5.0 mol% based on the linker) were mixed in DMSO (1.0 mL) in a sealed test tube. The resultant mixture was stirred under blue LED irradiation (PR160L-427, 390-470 nm) at room temperature in a N_2 atmosphere for 24 hours. After the reaction, the **Zr-PZDB** catalyst was recovered via centrifugation in a glovebox, washed with DMSO (1.0 mL × 3), and used direct in subsequent cycles of reactions. The supernatant was then subjected to ^{1}H NMR analysis. This procedure was repeated three times.

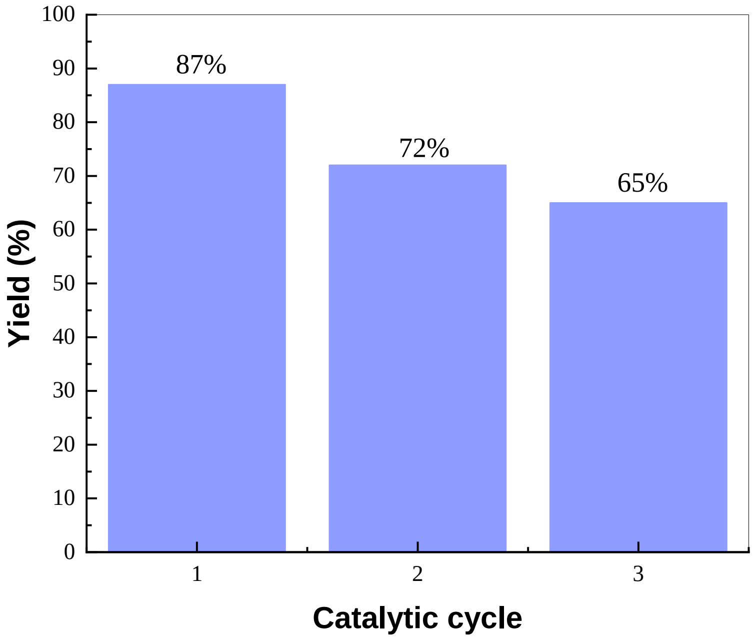

Figure S17. Yields of **6a** with recovered **Zr-PZDB** in three consecutive runs

Recovery of Zr-PZDB catalyst. Aryl sulfonium salt **4h** (99.6 mg, 0.2 mmol), *N*-methyl pyrrole (0.71 mL, 8.0 mmol), and **Zr-PZDB** (5.3 mg, 10.0 µmol, 5.0 mol% based on the linker) were mixed in DMSO (1.0 mL) in a sealed test tube. The resultant mixture was stirred under blue LED irradiation (PR160L-427, 390-470 nm) at room temperature in a N_2 atmosphere for 24 hours. After the reaction, the **Zr-PZDB** catalyst was

recovered via centrifugation, washed with DMSO (1.0 mL × 3) and acetone (1.0 mL × 3), dried in vacuum, and digested in a NaOH solution in D_2O under sonication for 3 minutes. The resultant mixture was then filtered, and the filtrate was dissolved in DMSO-d_6 (0.5 mL) for ^{1}H NMR analysis.

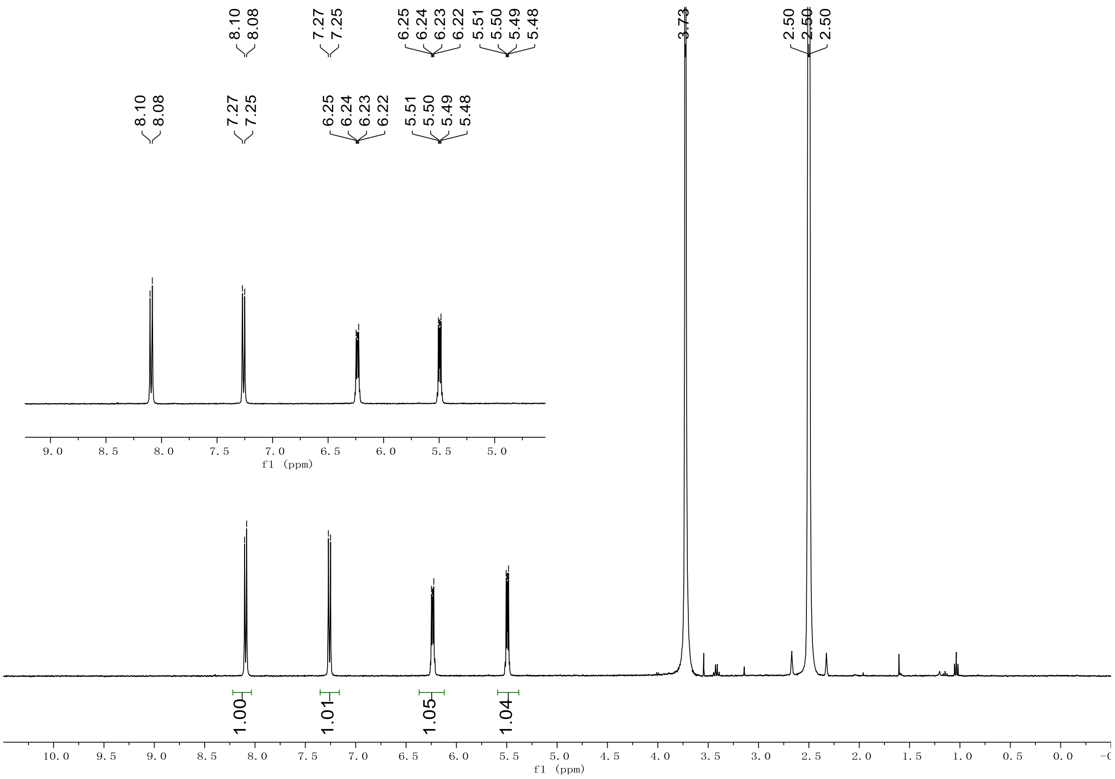

Figure S18. ^{1}H NMR spectrum of digested **Zr-PZDB-AR** in NaOH/D_2O/DMSO-d_6

Recovery of PZDB-Me catalyst. Aryl sulfonium salt **4h** (99.6 mg, 0.2 mmol), *N*-methyl pyrrole (0.71 mL, 8.0 mmol), and **PZDB-Me** (7.2 mg, 16 μmol, 8 mol%) were mixed in DMSO (1.0 mL) in a sealed test tube. The resultant mixture was stirred under blue LED irradiation (PR160L-427, 390-470 nm) at room temperature in a N_2 atmosphere for 24 hours. Afterward, the reaction mixture was subjected to thin layer chromatography (TLC) analysis, whose result indicated the disappearance of **PZDB-Me** catalyst (Figure S19). ^{1}H NMR spectrum of crude reaction mixture did not present the signals assignable to **PZDB-Me**, further verifying its susceptibility (Figure S20). To shed light on the possible transformations of **PZDB-Me**, HRMS analysis of crude reaction mixture was carried out, whose result suggested the formation of mono-, di-, and tri-substituted derivatives of **PZDB-Me** probably originating from the attack of the

corresponding aryl radicals (Figure S21).

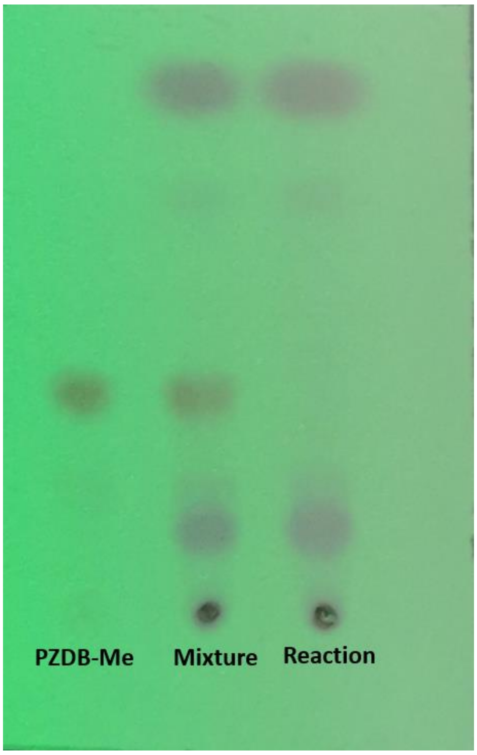

Figure S19. TLC analysis of the crude reaction mixture showing the disappearance of **PZDB-Me** catalyst after reaction

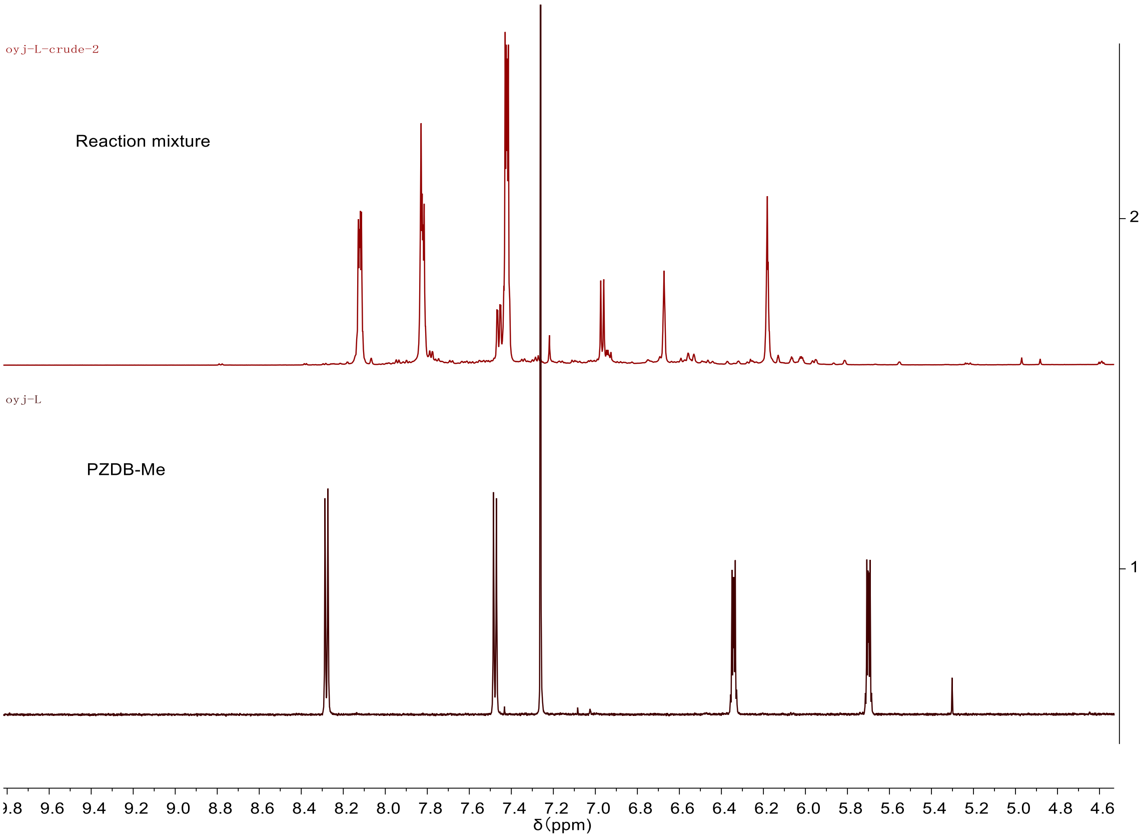

Figure S20. ^{1}H NMR spectra of crude reaction mixture (up) and PZDB-Me (down)

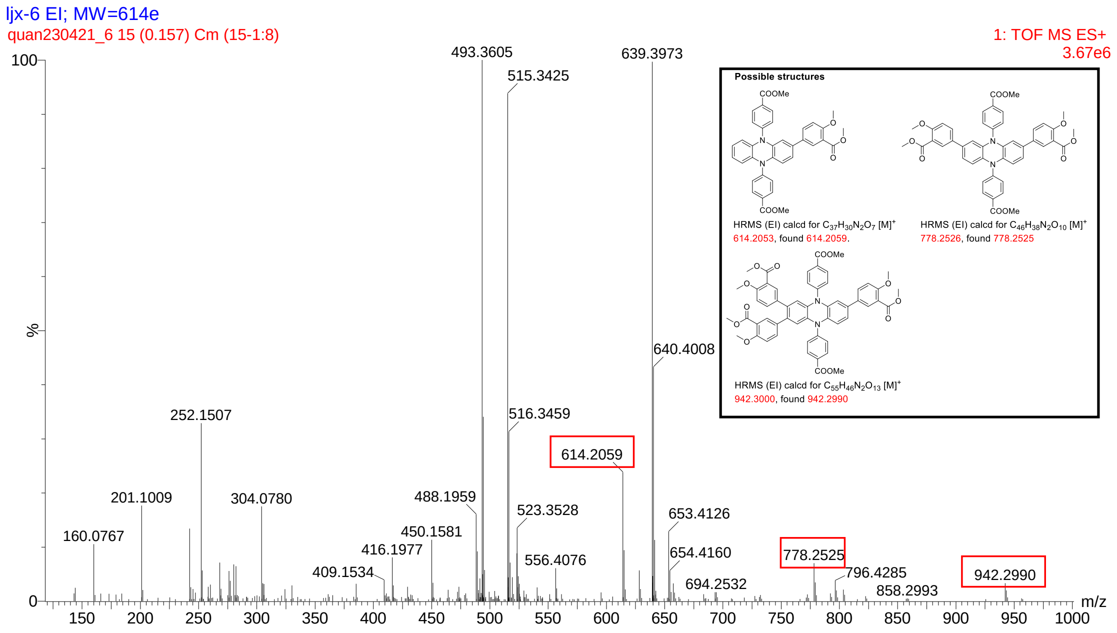

Figure S21. HRMS analysis of crude reaction mixture of homogeneous system

4. Mechanistic Study

4.1 Radical Capture Experiments

Radical capture experiment with TEMPO. 1a (11.8 mg, 0.05 mmol), THF (0.31 mL, 3.8 mmol), $NaHCO_3$ (8.4 mg, 0.1 mmol), (2,2,6,6-tetramethylpiperidin-1-yl)oxyl (TEMPO) (39.1 mg, 0.25 mmol, 5.0 equiv), and **Zr-PZDB** (1.3 mg, 2.5 μmol, 2.5 mol% based on the linker) were mixed in acetonitrile (0.5 mL) in a sealed test tube. The resulting mixture was stirred under blue LED irradiation (PR160L-427, 390-470 nm) at room temperature in a N_2 atmosphere for 24 hours. After that, the solvent was removed under vacuum, and the residue was then subjected to ^1H NMR and HRMS analysis.

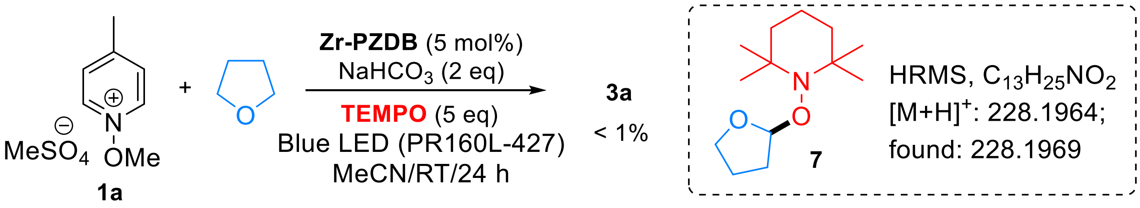

Figure S22. Radical capture experiment with TEMPO

Radical capture experiment with 1,1-diphenylethene. 4h (99.6 mg, 0.2 mmol), *N*-methyl pyrrole (0.71 mL, 8.0 mmol), 1,1-diphenylethene (0.18 mL, 1.0 mmol, 5.0 equiv), and **Zr-PZDB** (5.3 mg, 10.0 μmol, 5.0 mol% based on the linker) were mixed in DMSO (1.0 mL) in a sealed test tube. The resulting mixture was stirred under blue LED irradiation (PR160L-427, 390-470 nm) at room temperature in a N_2 atmosphere for 24 hours. After that, the solvent was removed under vacuum, and the residue was then subjected to ^1H NMR analysis and HRMS analysis.

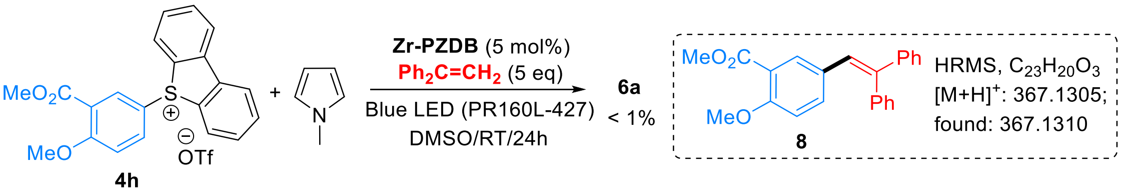

Figure S23. Radical capture experiment with 1,1-diphenylethene

4.2 Radical Clock Experiments

Radical clock experiment of alkyl radical. **1a** (11.8 mg, 0.05 mmol), THF (0.31 mL, 3.8 mmol), $NaHCO_3$ (8.4 mg, 0.1 mmol), alkene **9** (43.8 mg, 0.25 mmol, 5.0 equiv), and **Zr-PZDB** (1.3 mg, 2.5 μmol, 2.5 mol% based on the linker) were mixed in acetonitrile (0.5 mL) in a sealed test tube. The resulting mixture was stirred under blue LED irradiation (PR160L-427, 390-470 nm) at room temperature in a N_2 atmosphere for 24 hours. After that, the solvent was removed under vacuum. The crude product was purified by column chromatography (eluent: n-hexane/AcOEt 10:1 to 5:1) to provide **10** in 39 % yield.

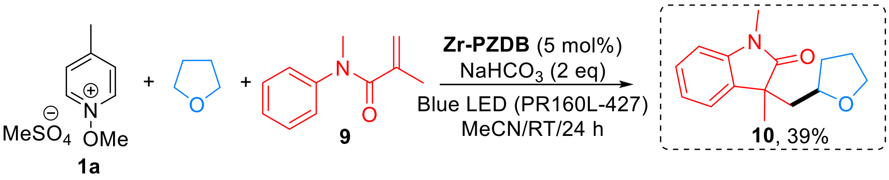

Figure S24. Radical clock experiment of alkyl radical

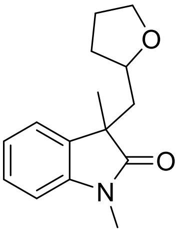

10: Yield 39%. Colorless liquid. ^{1}H NMR (600 MHz, Chloroform-d). δ 7.29 – 7.26 (m, 1H), 7.18 (dd, J = 7.3, 1.3 Hz, 1H), 7.07 – 7.01 (m, 1H), 6.84 (d, J = 7.8 Hz, 1H), 3.71 – 3.67 (m, 1H), 3.54 – 3.46 (m, 2H), 3.20 (s, 3H), 2.23 (dd, J = 14.0, 9.7 Hz, 1H), 1.87 (dd, J = 14.0, 3.9 Hz, 1H), 1.83 – 1.78 (m, 1H), 1.71 – 1.62 (m, 3H), 1.35 (s, 3H).Characterization data matched that reported in the literature.[24]

Radical clock experiment of aryl radical. **4b** (93.2 mg, 0.2 mmol), alkene **9** (175 mg, 1.0 mmol, 5.0 equiv), and **Zr-PZDB** (5.3 mg, 10.0 μmol, 5.0 mol% based on the linker) were mixed in DMSO (1.0 mL) in a sealed test tube. The resulting mixture was stirred under blue LED irradiation (PR160L-427, 390-470 nm) at room temperature in a N_2 atmosphere for 24 hours. After that, the solvent was removed under vacuum, and the residue was then subjected to ^{1}H NMR analysis and HRMS analysis. The crude product was purified by column chromatography (eluent: n-hexane/AcOEt 50:1 to 20:1) to provide **11** in 48 % yield.

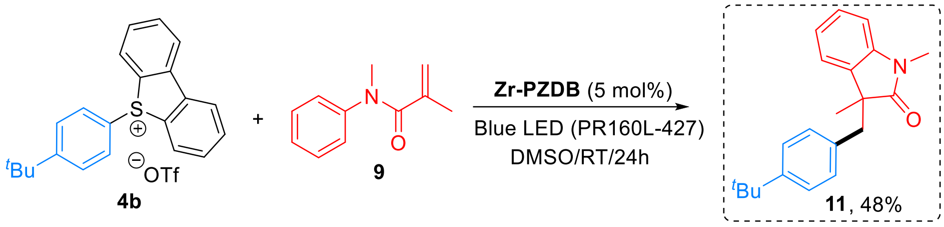

Figure S25. Radical clock experiment of aryl radical

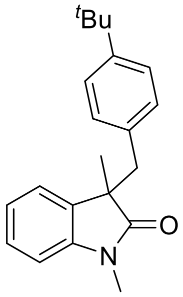

11: Yield 48%. White solid. ^{1}H NMR (600 MHz, Chloroform-d) δ 7.20 (td, *J* = 7.6, 1.3 Hz, 1H), 7.12 – 7.06 (m, 3H), 7.04 (t, *J* = 7.4 Hz, 1H), 6.79 (d, *J* = 8.1 Hz, 2H), 6.64 (d, *J* = 7.7 Hz, 1H), 3.02 (d, *J* = 5.4 Hz, 2H), 2.99 (s, 3H), 1.46 (s, 3H), 1.23 (s, 9H). Characterization data matched that reported in the literature.[25]

4.3 Light On/Off Experiments

Light on/off experiments for coupling of 1a/THF. 1a (11.8 mg, 0.05 mmol), THF (0.31 mL, 3.8 mmol), $NaHCO_3$ (0.1 mmol), and **Zr-PZDB** (1.3 mg, 2.5 μmol, 5 mol% based on the linker) were mixed in acetonitrile (0.5 mL) in a sealed test tube. Six parallel reactions were carried out simultaneously. The reaction vials were then stirred under light irradiation (PR160L-427, 390-470 nm) at room temperature for 6 hours. After that, the light was turned off, and one of the parallel reactions was quenched and subjected to ^{1}H NMR analysis. The other reactions were stirred in the dark for another 6 hours. Then, one of them was quenched and subjected to ^{1}H NMR analysis. The same light on/off procedure repeated until all the parallel reactions were quenched and analyzed.

Light on/off experiments for coupling of 4a/pyrrole. 4a (85.6 mg, 0.2 mmol), *N*-methyl pyrrole (0.71 mL, 8.0 mmol), and **Zr-PZDB** (5.3 mg, 10.0 μmol, 5.0 mol% based on the linker) were mixed in DMSO (1.0 mL) in a sealed test tube. Six parallel reactions were carried out simultaneously. The reaction vials were then stirred under light irradiation (PR160L-427, 390-470 nm) at room temperature for 4 hours. After that, the light was turned off, and one of the parallel reactions was quenched and subjected to ^{1}H NMR analysis. The other reactions were stirred in the dark for another 4 hours.

Then, one of them was quenched and subjected to ^{1}H NMR analysis. The same light on/off procedure repeated until all the parallel reactions were quenched and analyzed.

4.4 Hot-Filtration Experiments.

Hot-filtration experiments for coupling of 1a/THF. **1a** (11.8 mg, 0.05 mmol), THF (0.31 mL, 3.8 mmol), $NaHCO_3$ (0.1 mmol), and **Zr-PZDB** (1.3 mg, 2.5 μmol, 5 mol% based on the linker) were mixed in acetonitrile (0.5 mL) in a sealed test tube. The tube was then taken out from the glovebox after being tightly screw-caped, and the reaction mixture was subsequently stirred under irradiation (PR160L-427, 390-470 nm) at room temperature for 12 hours. Afterward, the MOF catalyst was separated from the reaction mixture by centrifugation in the glovebox. Half of the supernatant was subjected to ^{1}H NMR analysis to identify the yield of **3a**. Another half was stirred under irradiation (PR160L-427, 390-470 nm) at room temperature for an additional 12 hours, and subsequently subjected to ^{1}H NMR analysis to identify the yield of **3a**.

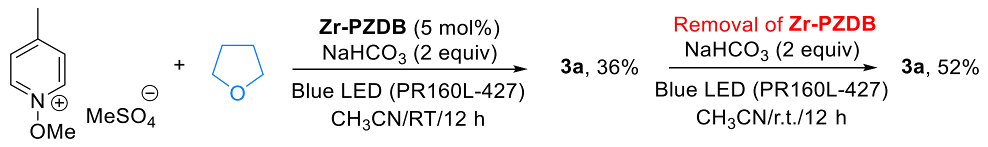

Figure S26. Hot-filtration experiments for coupling of **1a**/THF

Hot-filtration experiments for coupling of 4a/pyrrole. **4a** (85.6 mg, 0.2 mmol), *N*-methyl pyrrole (0.71 mL, 8.0 mmol), and **Zr-PZDB** (5.3 mg, 10.0 μmol, 5.0 mol% based on the linker) were mixed in DMSO (1.0 mL) in a sealed test tube. The tube was then taken out from the glovebox after being tightly screw-caped, and the reaction mixture was subsequently stirred under irradiation (PR160L-427, 390-470 nm) at room temperature for 12 hours. Afterward, the MOF catalyst was separated from the reaction mixture by centrifugation in the glovebox. Half of the supernatant was subjected to ^{1}H NMR analysis to identify the yield of **6a**. Another half was stirred under irradiation (PR160L-427, 390-470 nm) at room temperature for an additional 12 hours, and subsequently subjected to ^{1}H NMR analysis to identify the yield of **6a**.

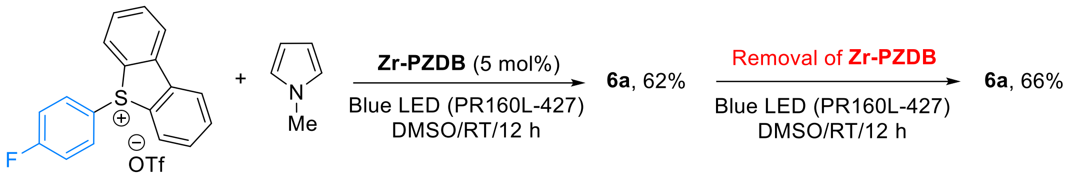

Figure S27. Hot-filtration experiments for coupling of **4a**/pyrrole

4.5 Determination of Quantum Yield

An aqueous H_2SO_4 (0.05 M, 6 mL) solution of potassium ferrioxalate (339.3 mg, 0.9 mmol) was irradiated by a Kessil lamp (PR160L-427, 10 W, 4 cm) for 1 min. A proportion (10 μL) of the solution was added to the aqueous H_2SO_4 (0.5 M, 5 mL) including sodium acetate trihydrate (1.1 g) and 1,10-phenantroline (50 mg). UV-vis analysis was then carried out to measure the concentration of generated Fe^{2+} via illumination, according to the calibration curve by plotting the concentration of $[Fe(phen)_3]^{2+}$ versus the corresponding UV-vis absorption intensity at 510 nm (Figure S28).[26] The amount of generated Fe^{2+} was calculated as 0.108 mmol $\{(3.62 * 10^{-5}\ \mathrm{M} * 6\ \mathrm{mL} * 500\ )\}$. On the other hand, **4a** (85.6 mg, 0.2 mmol), *N*-methyl pyrrole (0.71 mL, 8.0 mmol), and **Zr-PZDB** (5.3 mg, 10.0 μmol, 5.0 mol% based on the linker) were mixed in DMSO (1.0 mL) in a sealed test tube. The resulting mixture was illuminated by the same lamp (PR160L-427, 40 W, 4 cm) at room temperature in a N_2 atmosphere for 1 hour. After that, the reaction was quenched with aqueous saturated $NaHCO_3$ and diluted with EtOAc. The amount of generated product **6a** was detected as 0.074 mmol by ^1H NMR using 1,3,5-trimethoxybenzene as internal standard (Figure S29). The quantum yield (Φ) of potassium ferrioxalate photoreduction is known as 1.13. On this basis, the quantum yield of pyrrole arylation was calculated as 0.003 $\{(0.074 \times 1.13) \div (0.108 \times 4 \times 60)\}$.

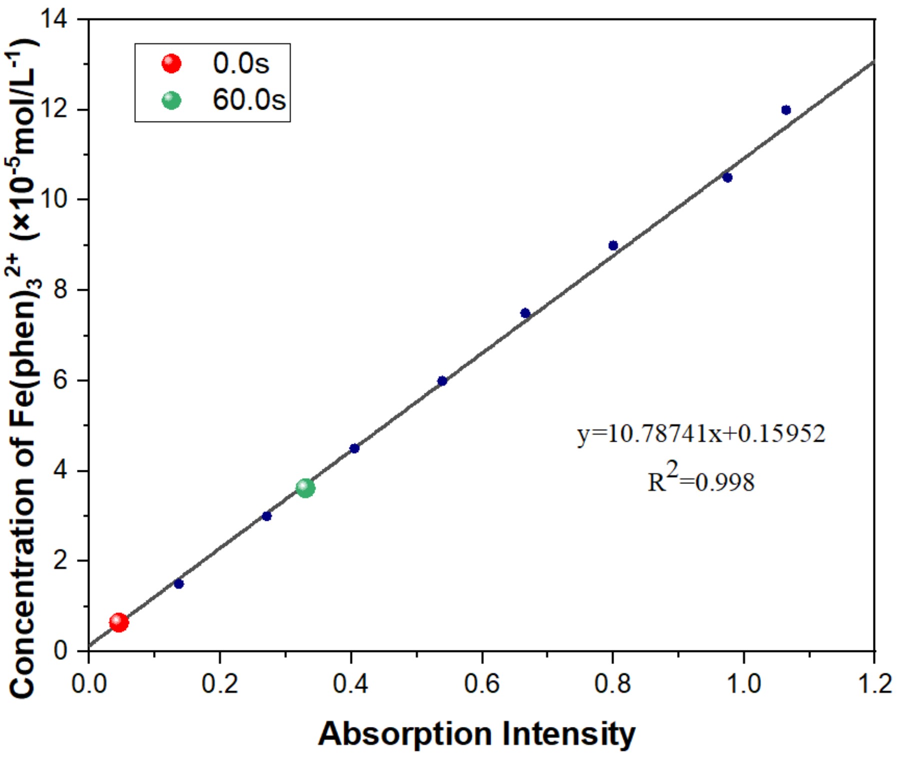

Figure S28. Calibration curve for measuring the concentration of Fe^{2+} ion.[26]

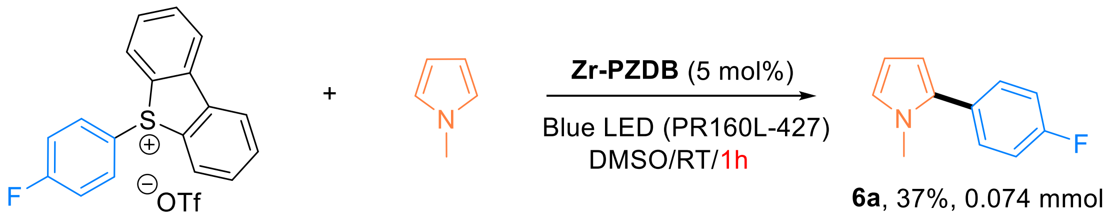

$$\text{Quantum yield (F)} = \frac{\text{moles of product formed}}{\text{einsteins of light absorbed}} = \frac{0.074 \times 1.13}{0.108 \times 4 \times 60} = \mathbf{0.003}$$

Figure S29. Measurement of quantum yield for pyrrole arylation.

4.6 Effects of Light Sources on Minisci-Type Reaction

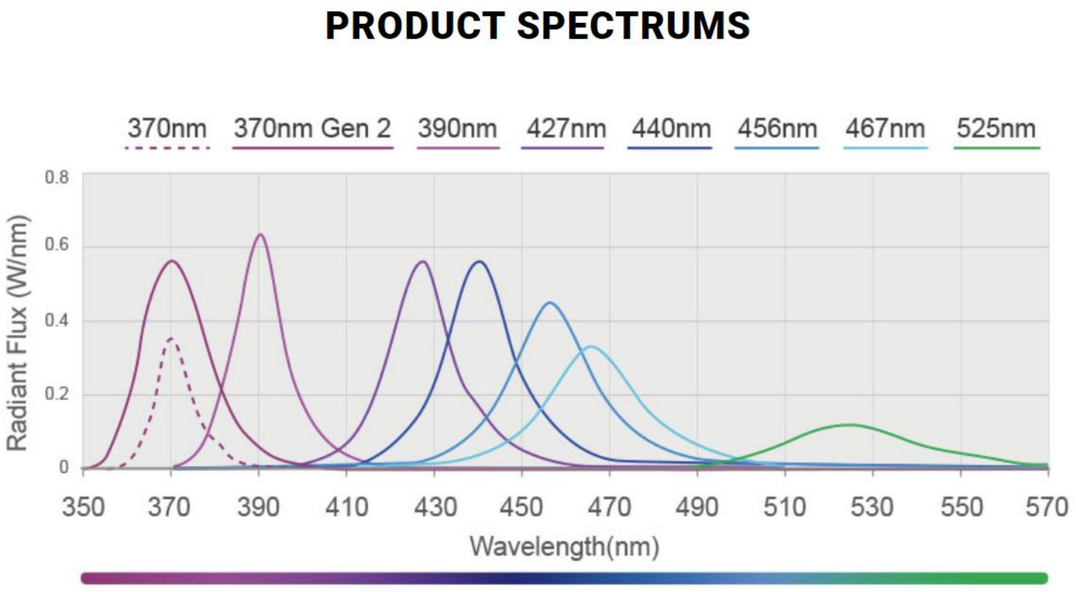

Figure S30. Spectrums of Kessil PR160L lamps.

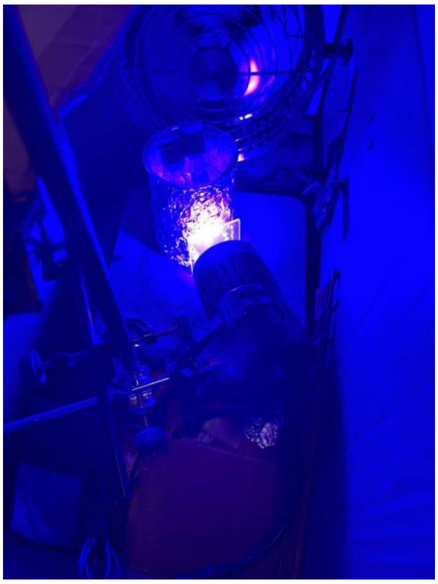

Figure S31. Experiment setup using light band filter

General procedure for experiments with a band filter. **1a** (11.8 mg, 0.05 mmol), THF (0.31 mL, 3.8 mmol), $NaHCO_3$ (8.4 mg, 0.10 mmol), and Zr-PZDB (1.3 mg, 2.5 μmol, 5 mol% based on linker) were mixed in acetonitrile (0.5 mL) in a sealed test tube. A band filter was placed in front of the tube. The resulting mixture was stirred under LED irradiation at room temperature in a N_2 atmosphere for 24 hours. After that, the solvent was removed under vacuum, and the residue was then subjected to ^1H NMR

analysis. More specifically, Kessil PR160L-427 (390-470 nm) with a 420 nm band filter and Kessil PR160L-390 (370-420 nm) with a 380 nm band filter were used respectively.

Experiment using Kessil PR160L-390. **1a** (11.8 mg, 0.05 mmol), THF (0.31 mL, 3.8 mmol), $NaHCO_3$ (8.4 mg, 0.10 mmol), and Zr-PZDB (1.3 mg, 2.5 μmol, 5 mol% based on linker) were mixed in acetonitrile (0.5 mL) in a sealed test tube. The resulting mixture was stirred under Kessil PR160L-390 irradiation at room temperature in a N_2 atmosphere for 24 hours. After that, the solvent was removed under vacuum, and the residue was then subjected to ^{1}H NMR analysis.

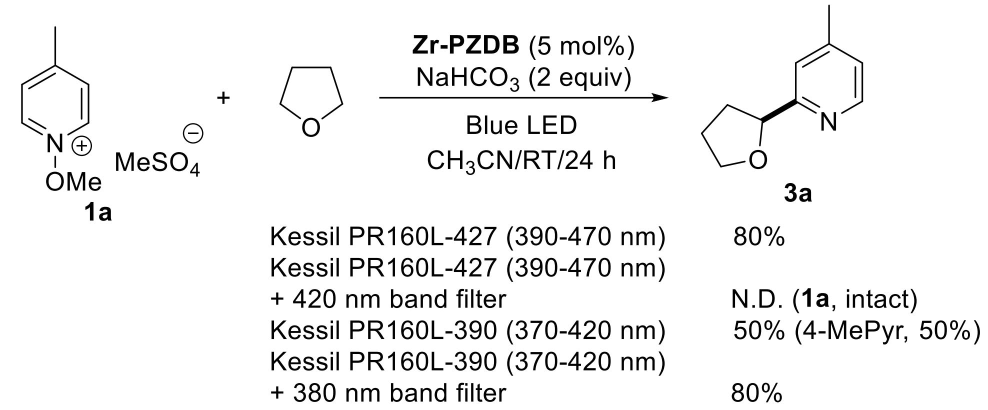

Figure S32. Effects of light sources on the reaction of pyridinium salt with THF.

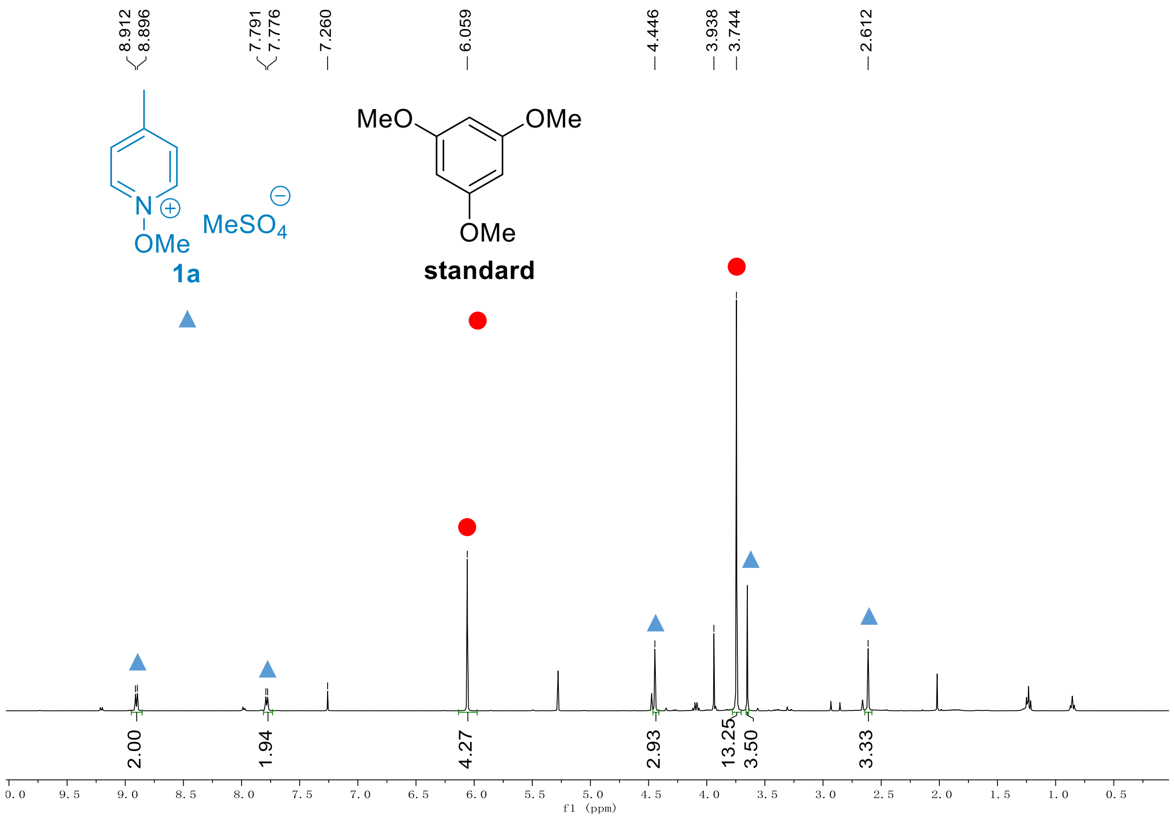

Figure S33. ^{1}H NMR spectrum of crude mixture using PR160L-427 lamp with a 420 nm filter.

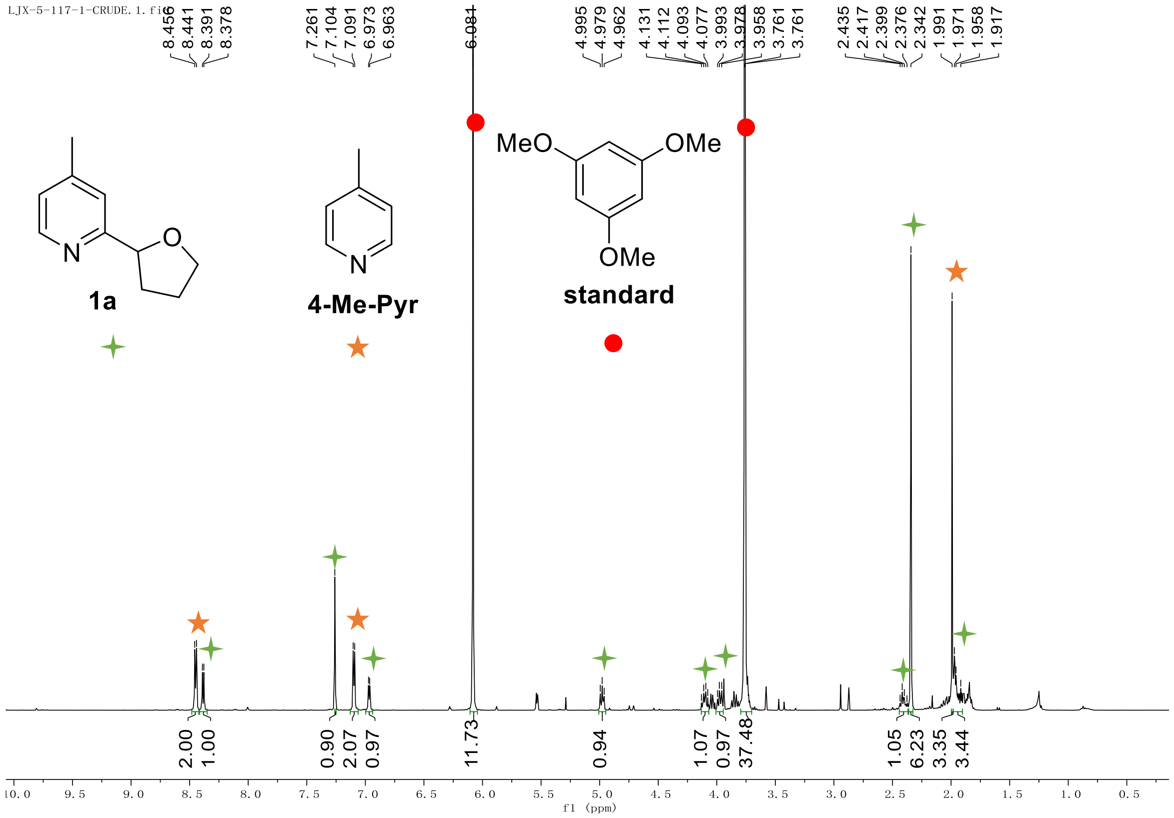

Figure S34. ^{1}H NMR spectrum of crude mixture using PR160L-390 lamp.

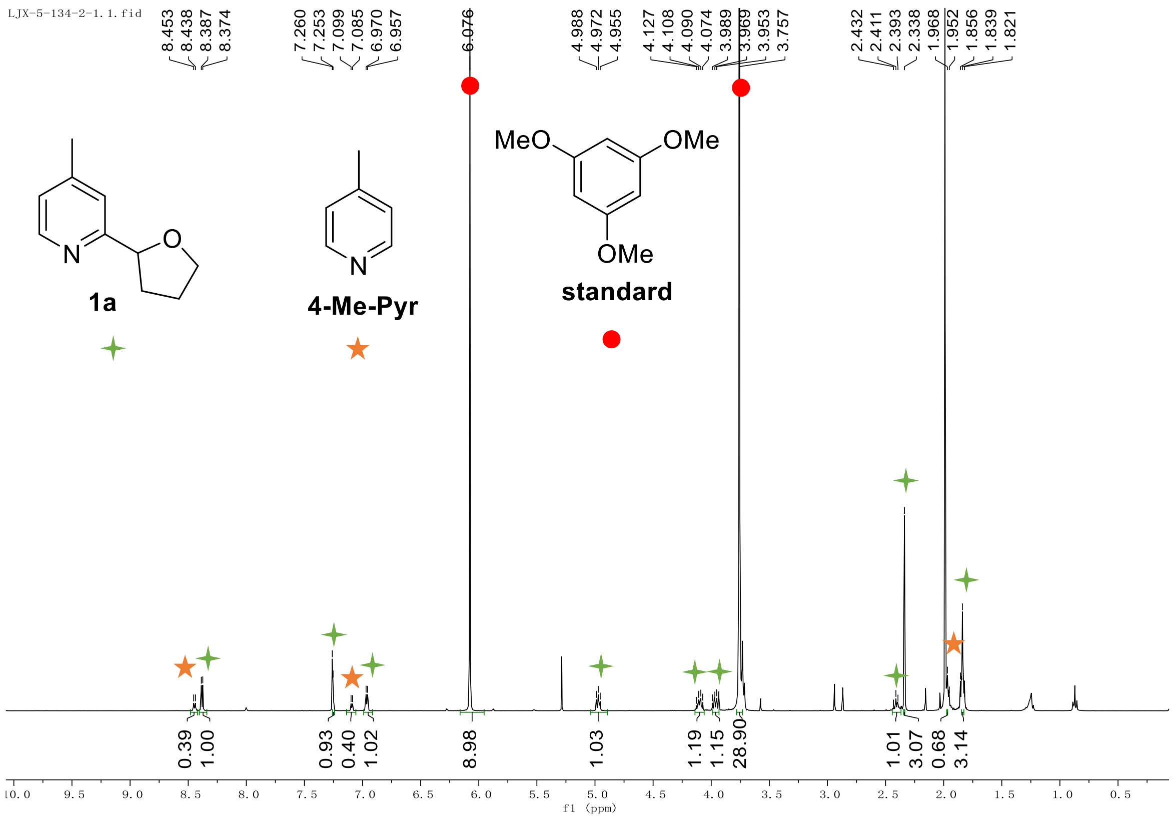

Figure S35. ^{1}H NMR spectrum of crude mixture using PR160L-390 lamp with a 380 nm filter.

4.7 Control Experiments using Activated Carbons as Support

Preparation of PZDB-H@C. PZDB-H (42.2 mg, 0.1 mmol) and activated carbon (45.0 mg) was mixed in DMF (2 mL) in a sealed tube. The reaction mixture was stirred at 60 °C overnight. The brown precipitate was collected by filtration and washed with DMF and DCM. The resulted **PZDB-H@C** was then dried via vacuum (58.6 mg). The percentage of PZDB-H in **PZDB-H@C** by weight was determined as 23.2%.

PZDB-H@C catalyzed reaction of 1a/THF. **1a** (11.8 mg, 0.05 mmol), THF (0.31 mL, 3.8 mmol), $NaHCO_3$ (8.4 mg, 0.10 mmol), and **PZDB-H@C** (4.5 mg, 2.5 μmol, 5 mol% based on PZDB-H) were mixed in acetonitrile (0.5 mL) in a sealed test tube. The resulting mixture was stirred under blue LED irradiation (PR160L-427, 390-470 nm) at room temperature in a N_2 atmosphere for 24 hours. After that, the solvent was removed under vacuum, and the residue was subjected to column chromatography on silica gel to give products **3a** in 15% yield.

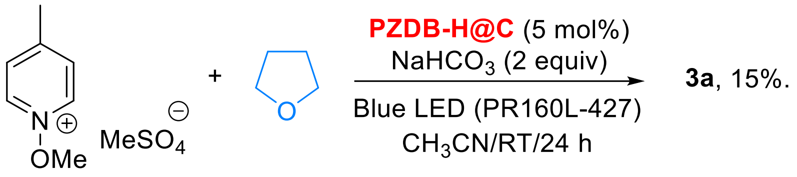

Figure S36. PZDB-H@C catalyzed coupling of **1a** with THF.

PZDB-H@C catalyzed coupling of 4a/pyrrole. **4a** (85.6 mg, 0.2 mmol), *N*-methyl pyrrole (0.71 mL, 8.0 mmol), and **PZDB-H@C** (18.2 mg, 10.0 μmol, 5.0 mol% based on PZDB-H) were mixed in DMSO (1.0 mL) in a sealed test tube. The resulting mixture was stirred under blue LED irradiation (PR160L-427, 390-470 nm) at room temperature in a N2 atmosphere for 24 hours. After that, the reaction was quenched with aqueous saturated $NaHCO_3$ and diluted with EtOAc. The organic layer was washed with brine, dried with Na_2SO_4, filtered, and concentrated in vacuo. The residue was subjected to column chromatography on silica gel to give products **6a** in 36% yield.

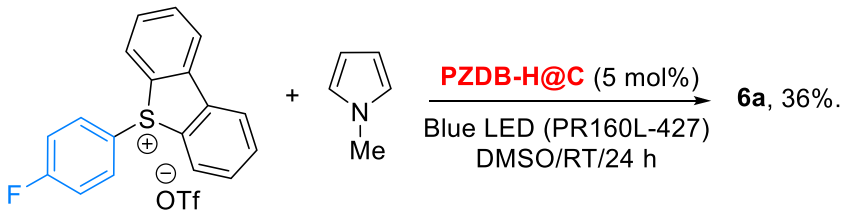

Figure S37. PZDB-H@C catalyzed coupling of **4a** with *N*-methyl pyrrole.

4.8 Determination of Adsorption Ability of Zr-PZDB to Pyridinium Salt 1a.

A mixture of **1a** (11.8 mg, 0.05 mmol), **Zr-PZDB** (26.0 mg, 0.05 mmol, 1 equiv based on the linker), and CH_3CN (1 mL) was stirred at room temperature in dark for 12 h. Then, the MOF solid was separated via centrifugation, and washed with CH_3CN (2 mL * 3). All the CH_3CN portions were combined and then dried under vacuum. The residue was subjected to ^{1}H NMR analysis to identify the amount of **1a** being remained in CH_3CN solution, which is measured as 5%.

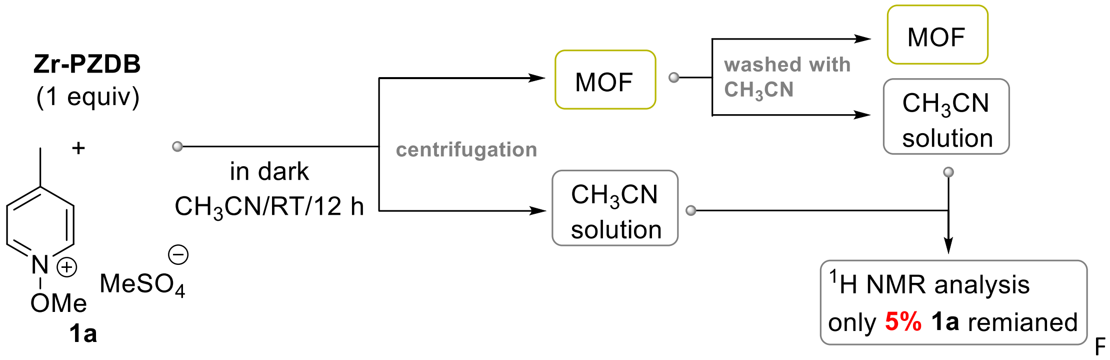

Figure S38. Adsorption of **1a** by **Zr-PZDB**.

5. NMR spectra

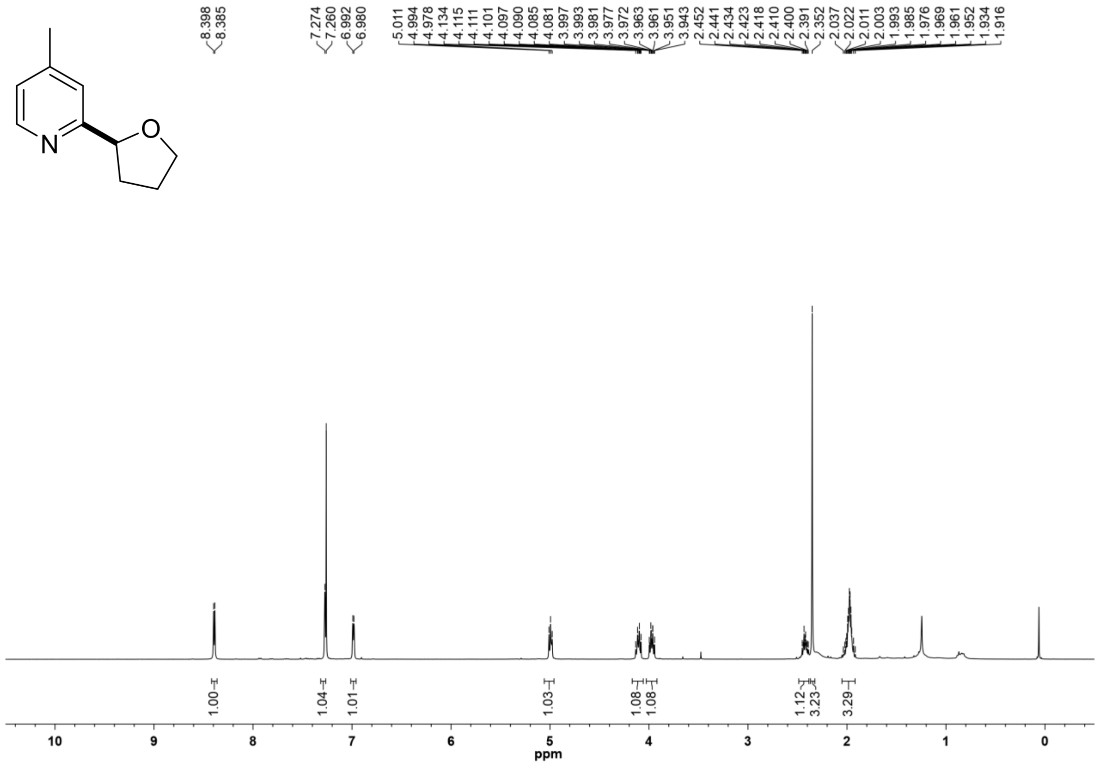

Figure S39. ^{1}H NMR spectrum of **3a** (400 MHz) in $CDCl_3$.

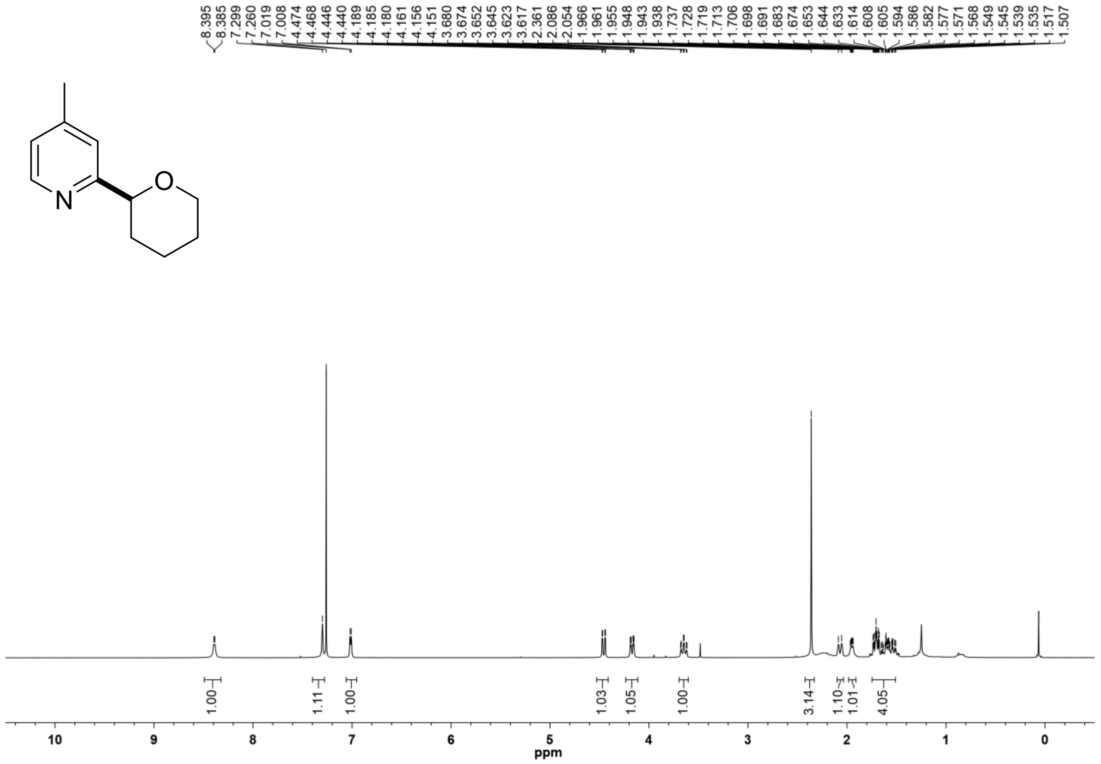

Figure S40. ^{1}H NMR spectrum of **3b** (400 MHz) in $CDCl_3$.

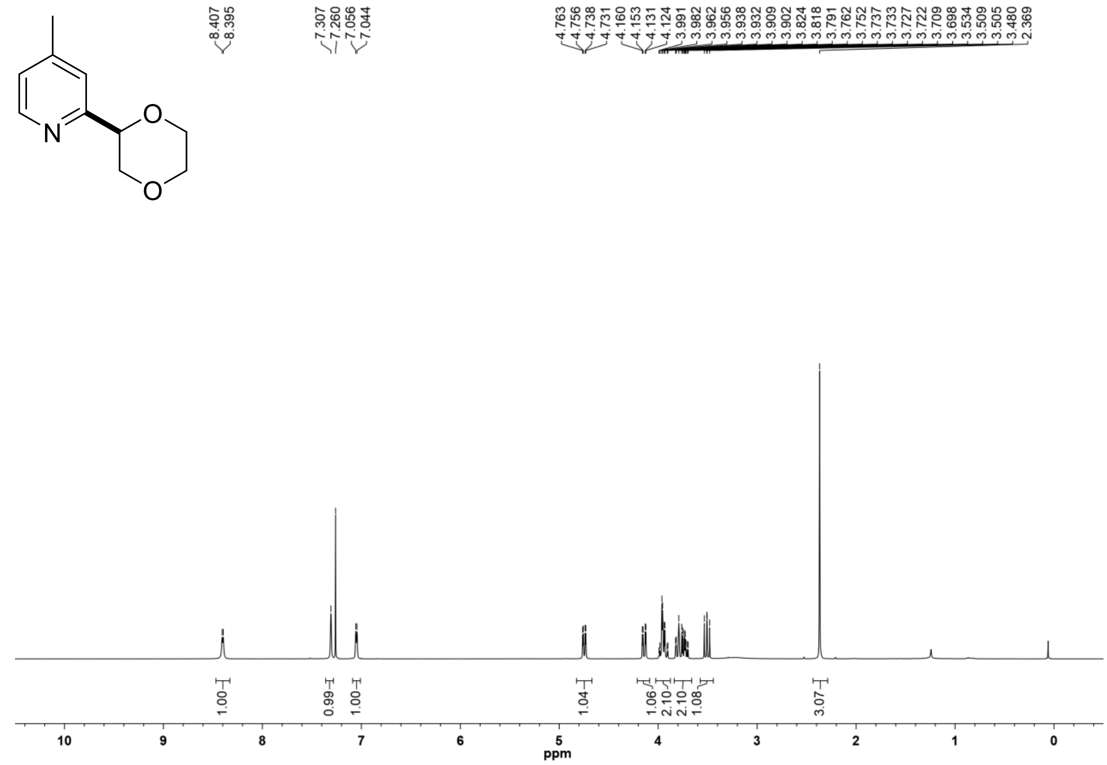

Figure S41. ^1H NMR spectrum of **3c** (400 MHz) in $CDCl_3$.

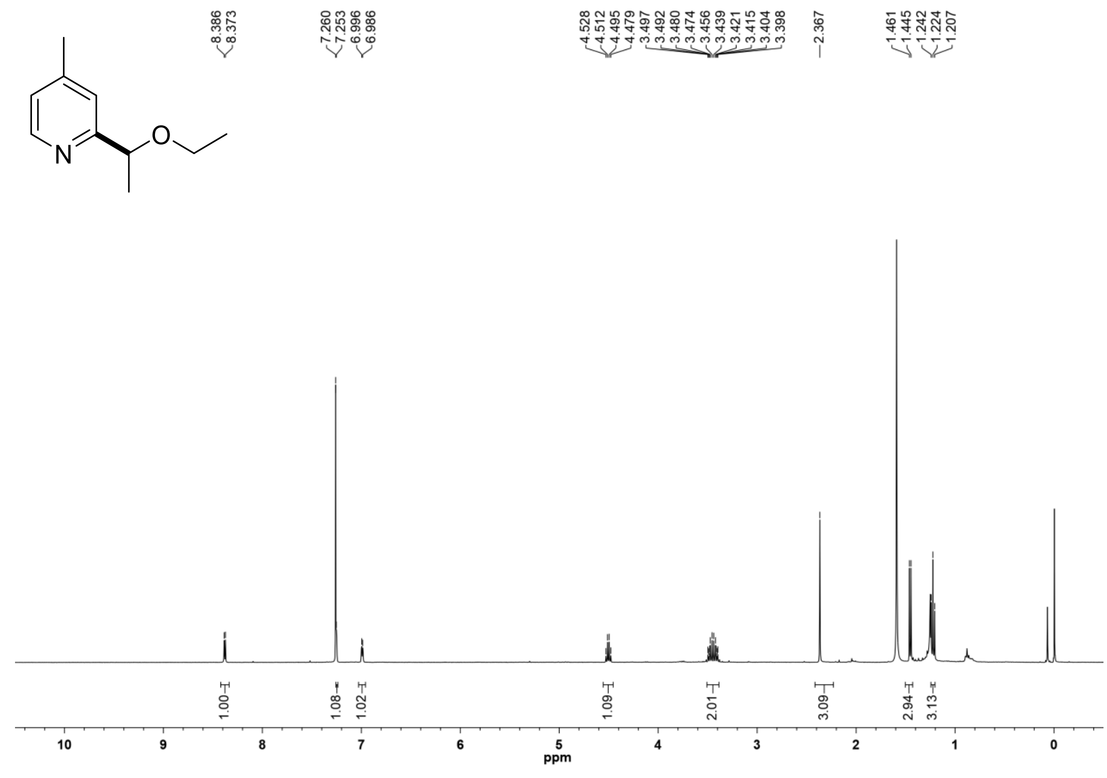

Figure S42. ^1H NMR spectrum of **3d** (400 MHz) in $CDCl_3$.

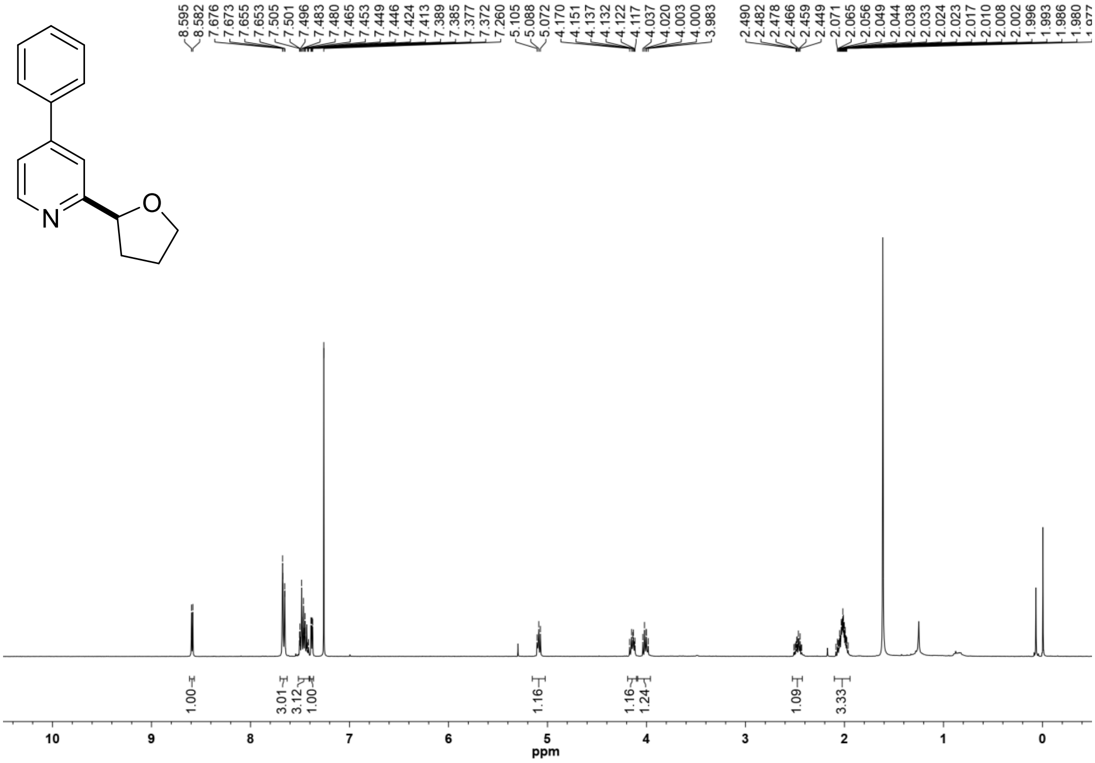

Figure S43. ^{1}H NMR spectrum of **3e** (400 MHz) in $CDCl_3$.

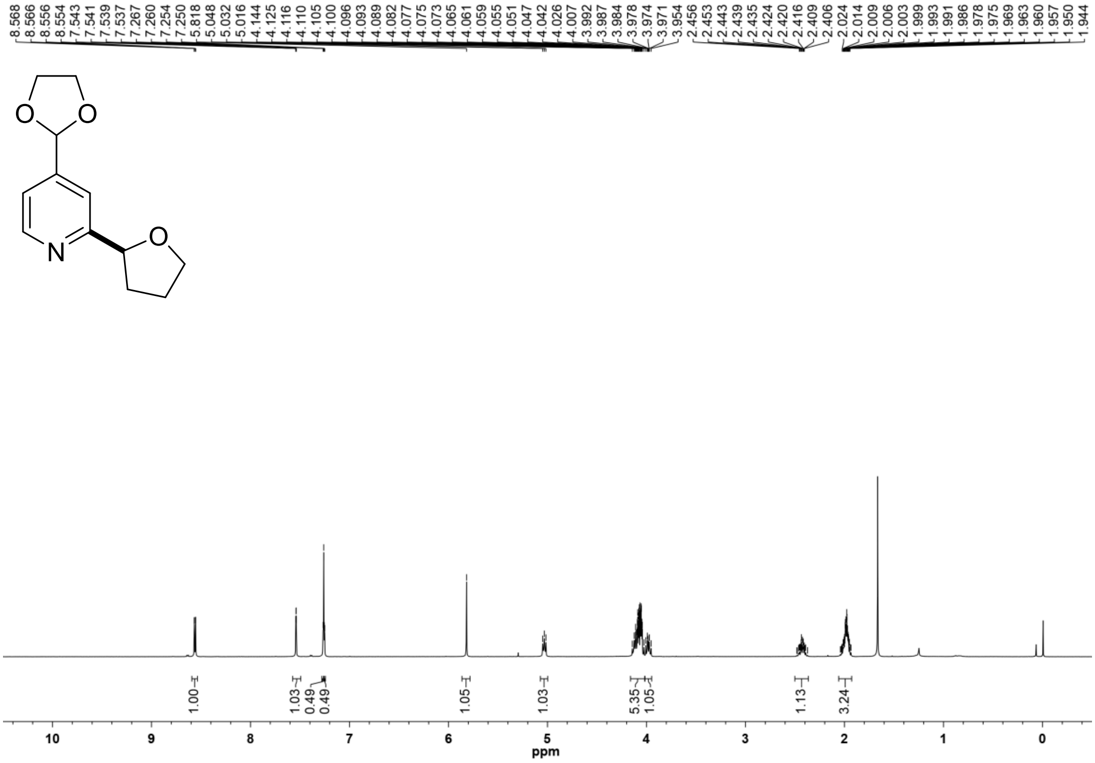

Figure S44. ^{1}H NMR spectrum of **3f** (400 MHz) in $CDCl_3$.

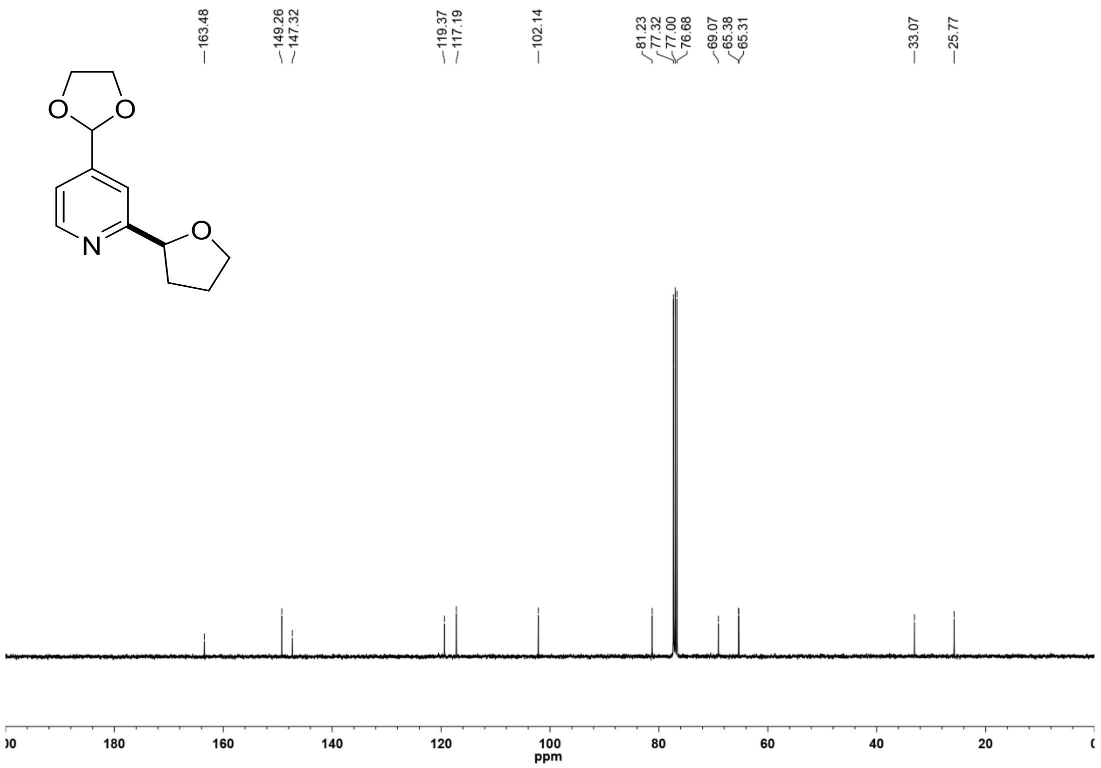

Figure S45. ^{13}C NMR spectrum of **3f** (100 MHz) in $CDCl_3$.

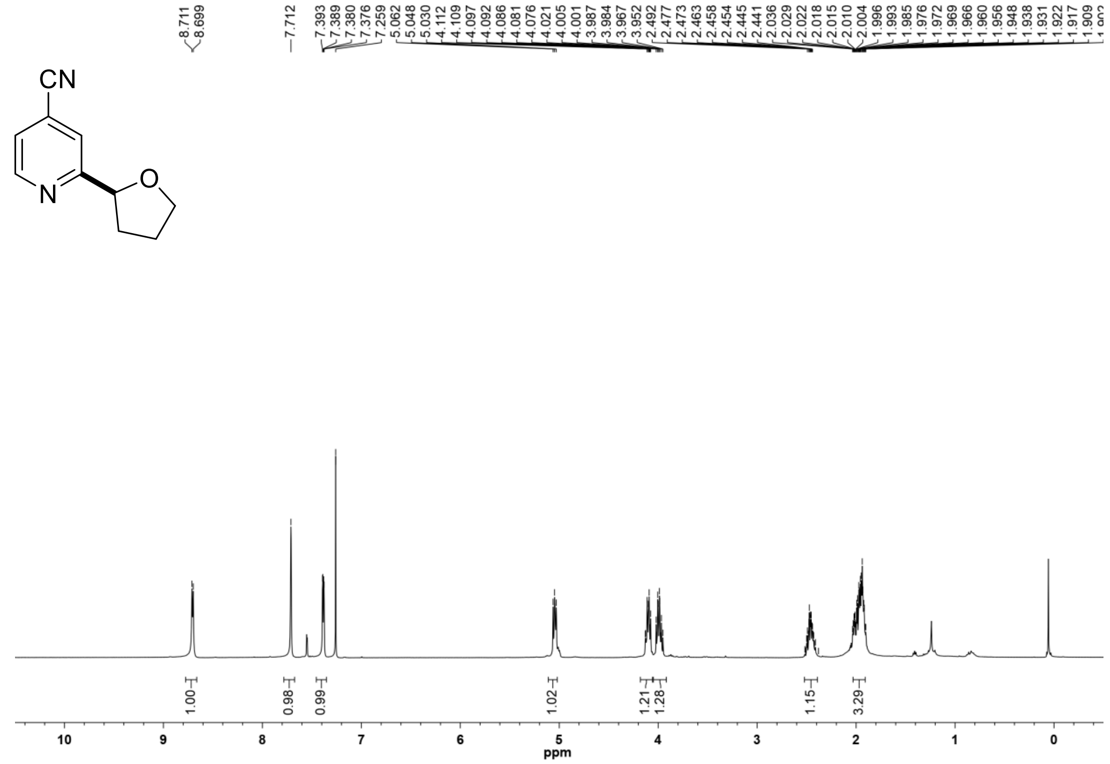

Figure S46. ^{1}H NMR spectrum of **3g** (400 MHz) in $CDCl_3$.

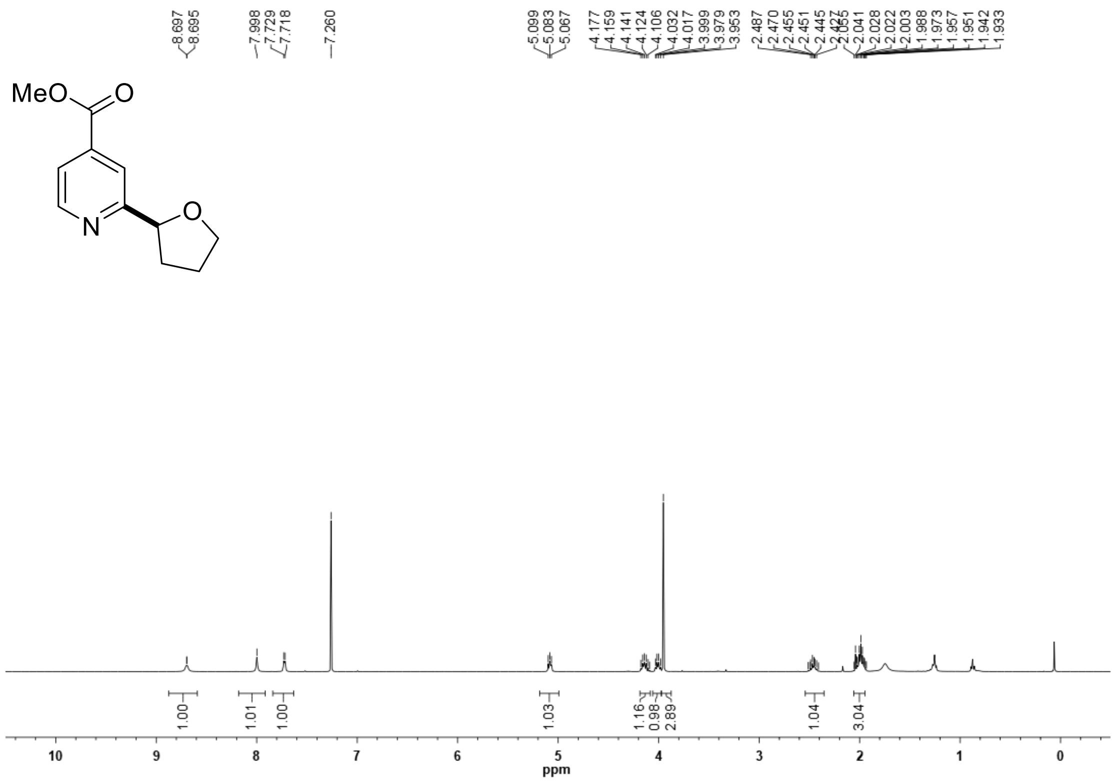

Figure S47. ^{1}H NMR spectrum of **3h** (400 MHz) in $CDCl_3$.

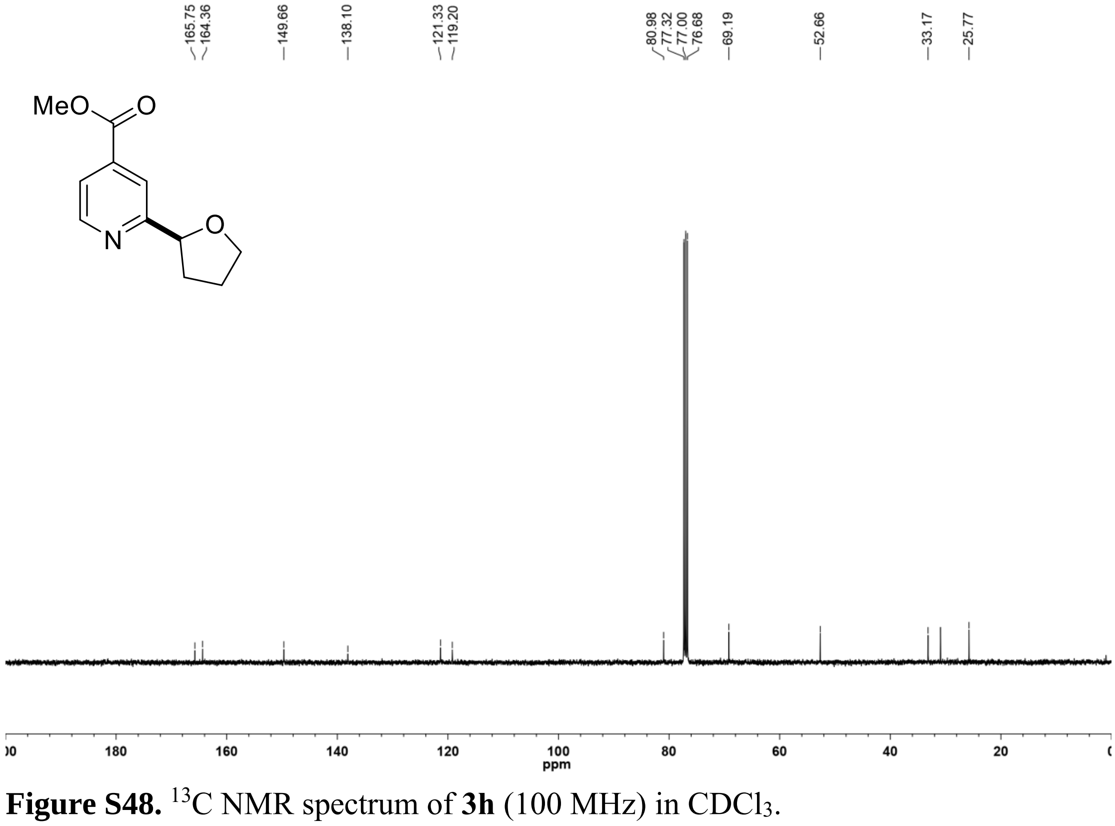

Figure S48. ^{13}C NMR spectrum of **3h** (100 MHz) in $CDCl_3$.

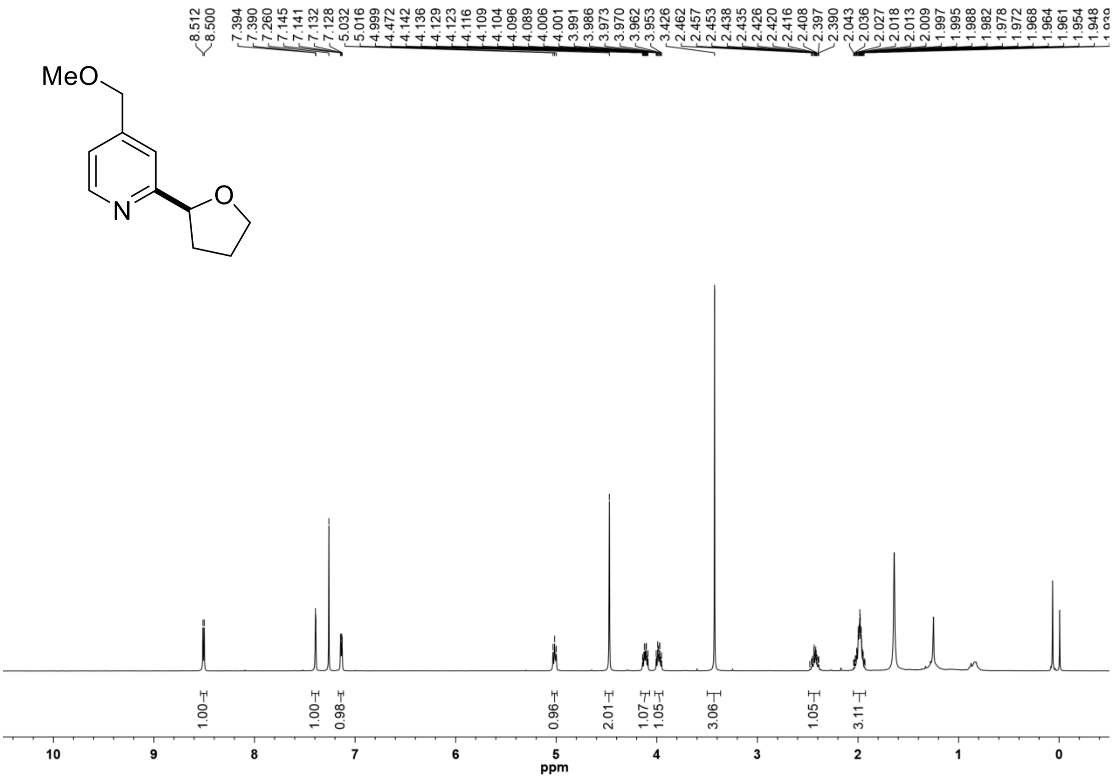

Figure S49. ^{1}H NMR spectrum of **3i** (400 MHz) in $CDCl_3$.

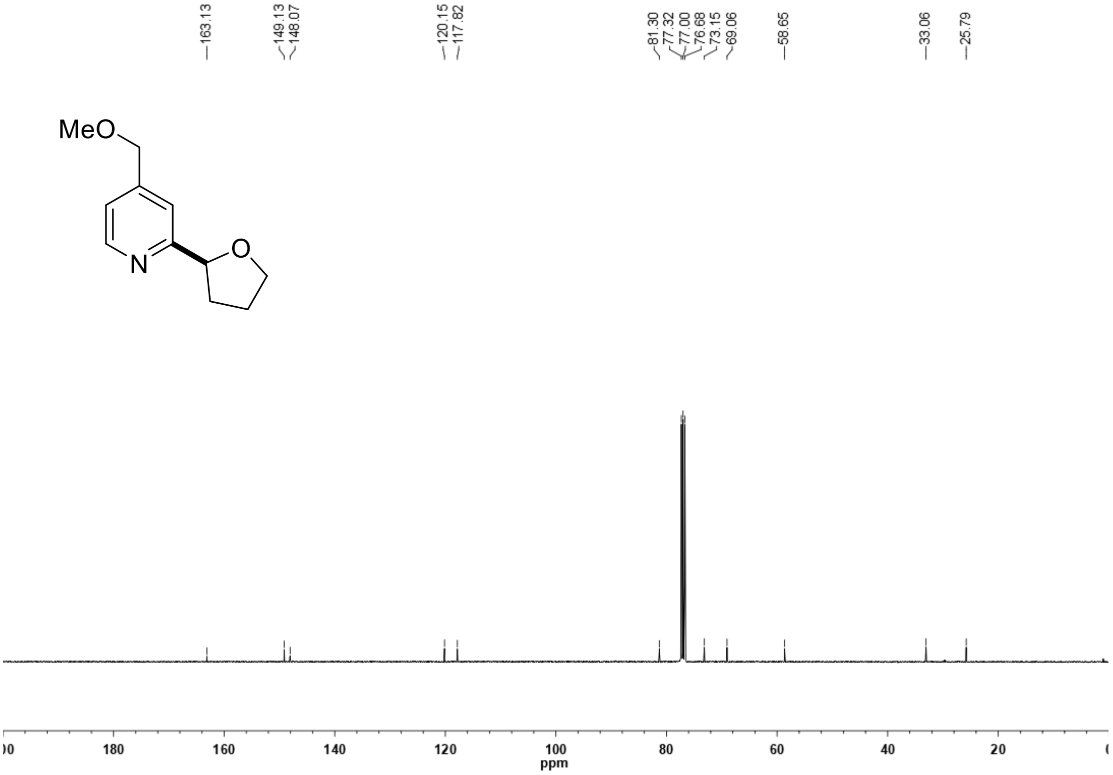

Figure S50. ^{13}C NMR spectrum of **3i** (100 MHz) in $CDCl_3$.

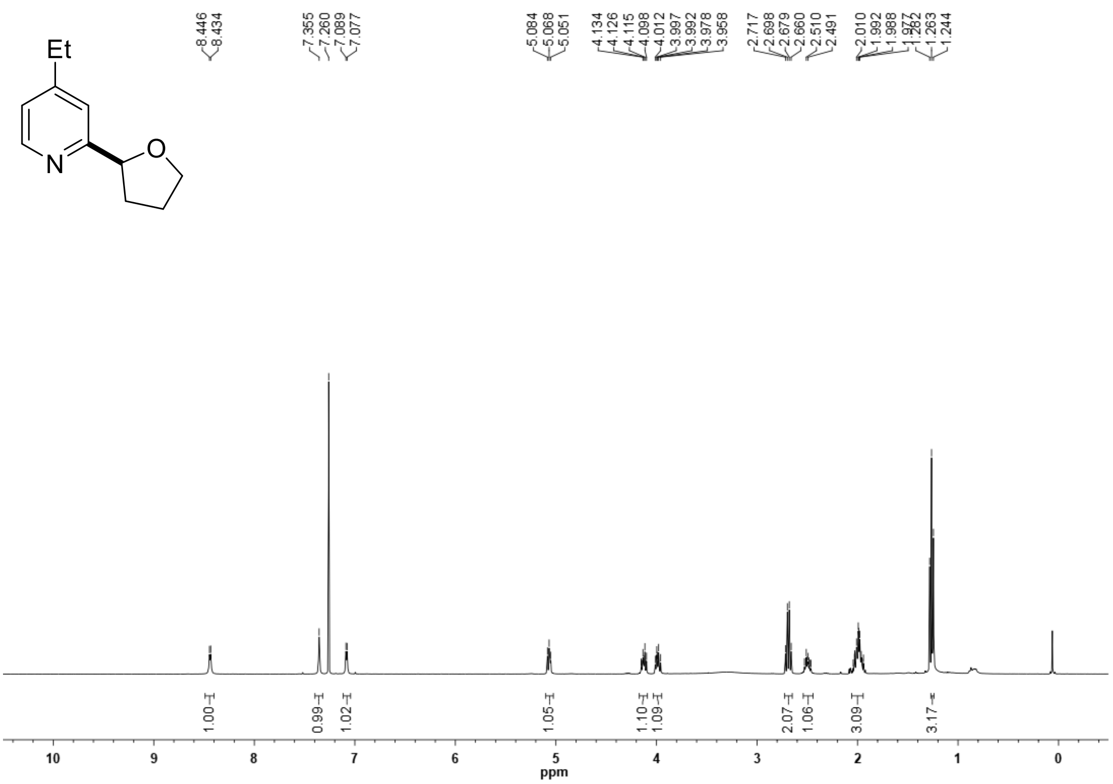

Figure S51. ^{1}H NMR spectrum of **3j** (400 MHz) in $CDCl_3$.

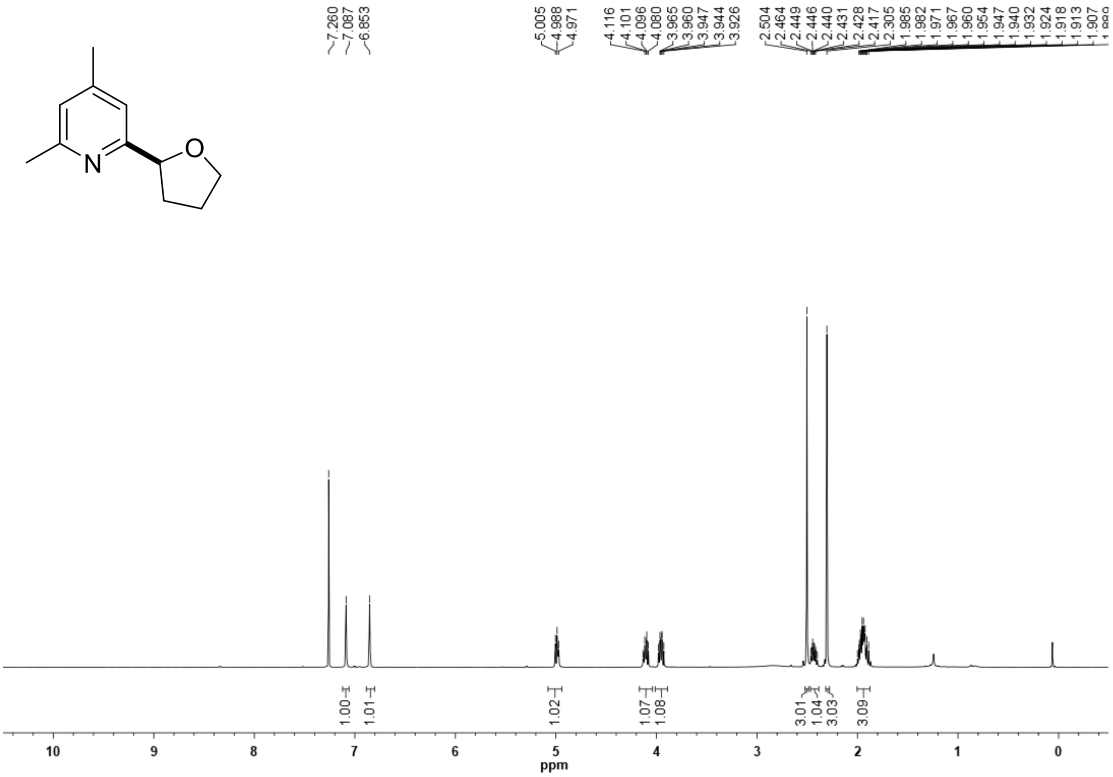

Figure S52. ^{1}H NMR spectrum of **3k** (400 MHz) in $CDCl_3$.

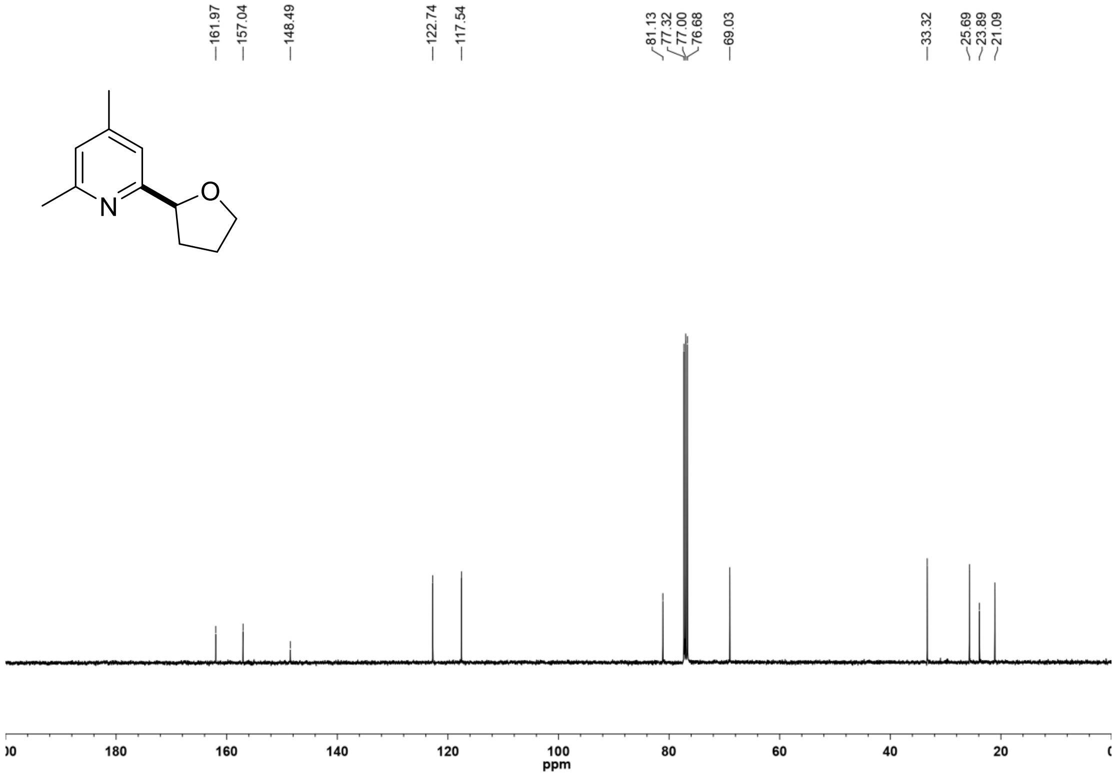

Figure S53. ^{13}C NMR spectrum of **3k** (100 MHz) in $CDCl_3$.

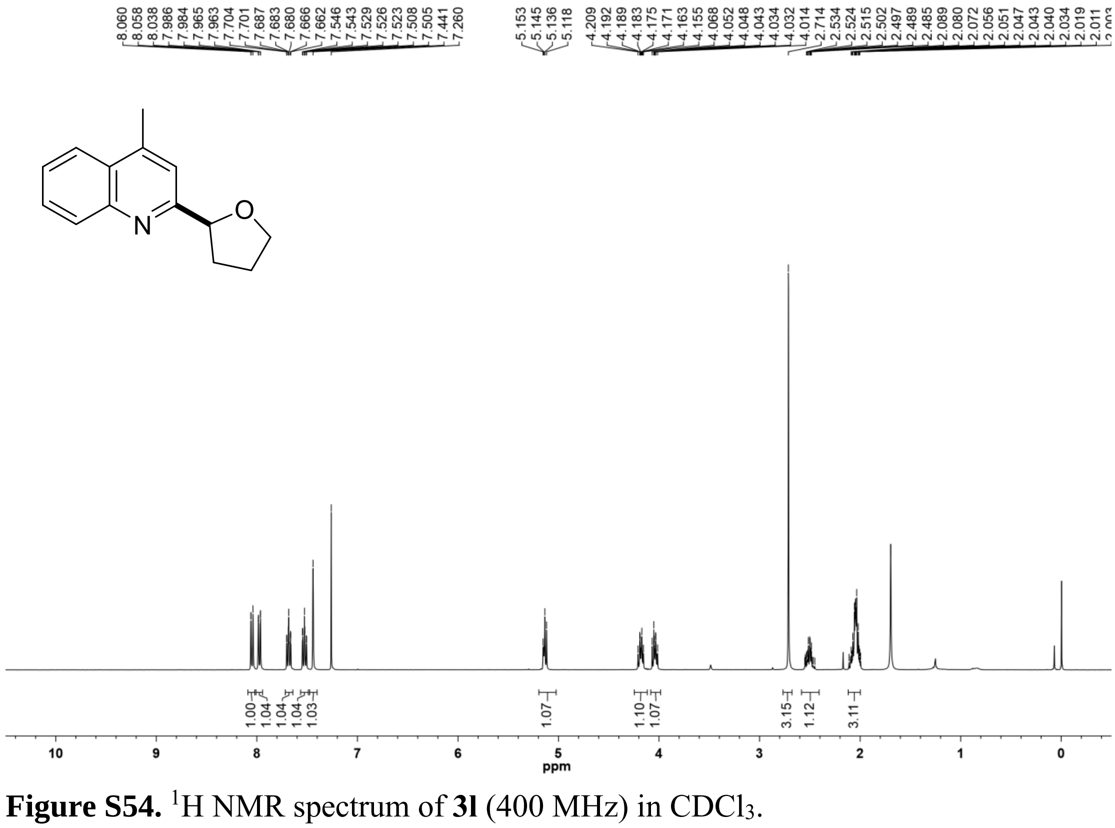

Figure S54. ^{1}H NMR spectrum of **3l** (400 MHz) in $CDCl_3$.

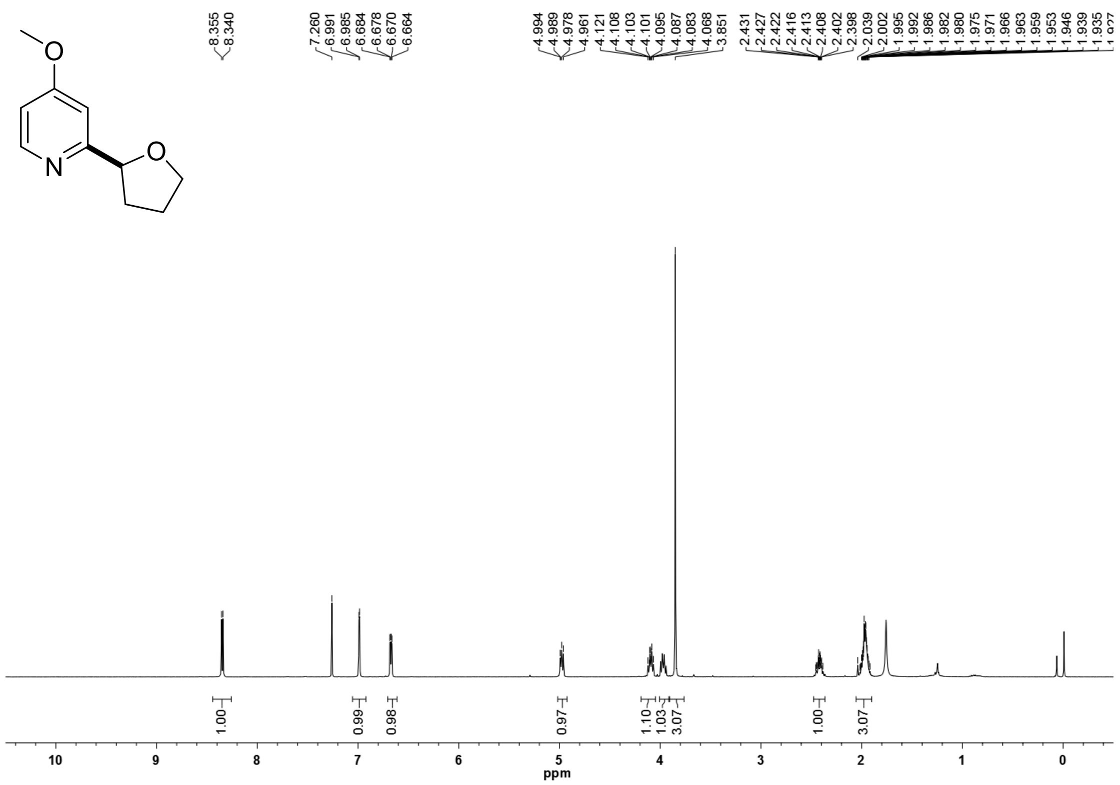

Figure S55. ^{1}H NMR spectrum of **3m** (400 MHz) in $CDCl_3$.

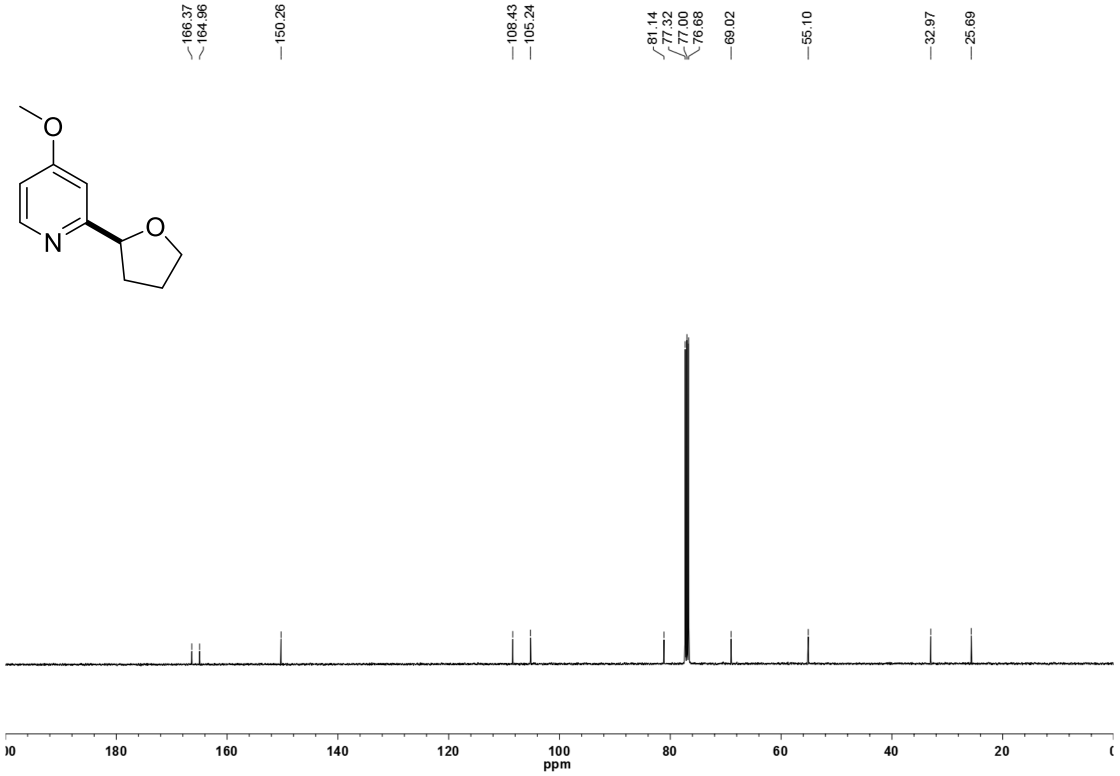

Figure S56. ^{13}C NMR spectrum of **3m** (100 MHz) in $CDCl_3$.

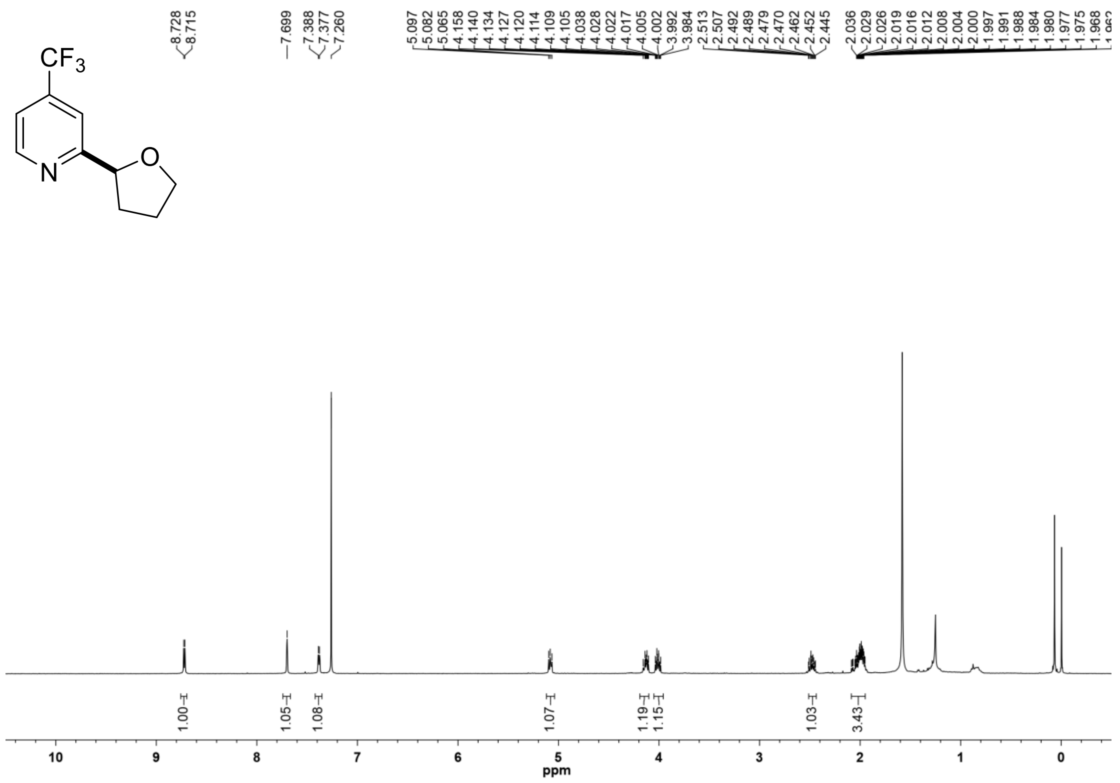

Figure S57. ^{1}H NMR spectrum of **3n** (400 MHz) in $CDCl_3$.

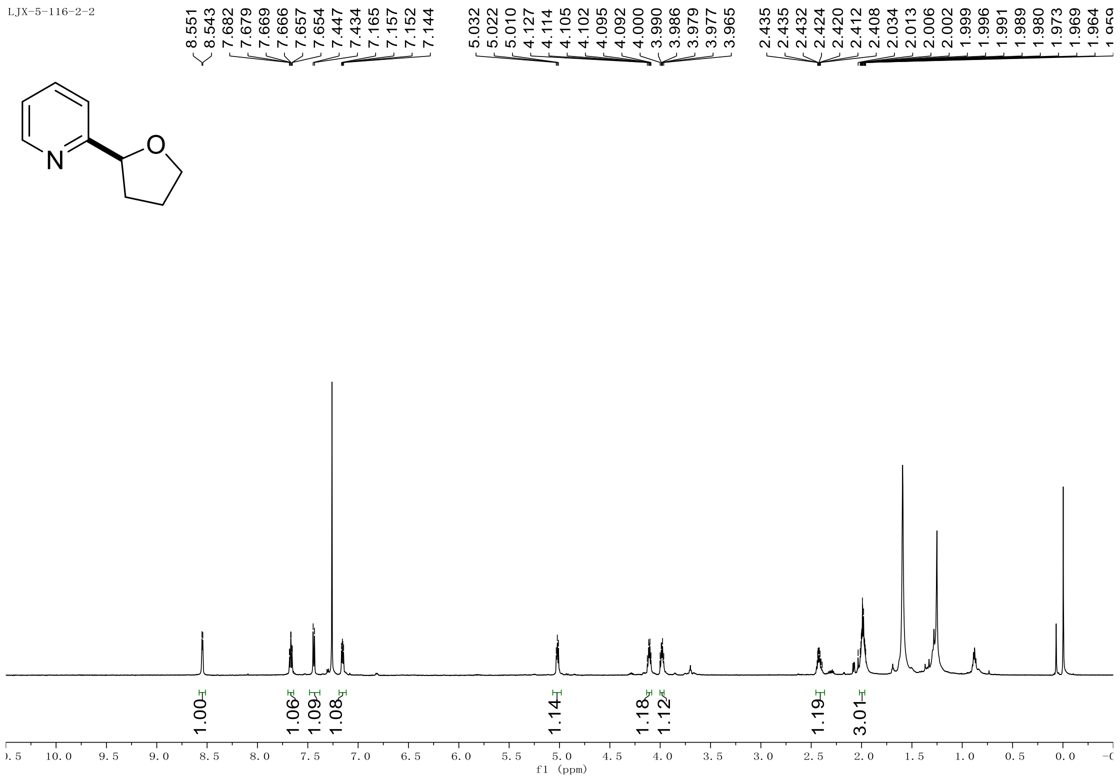

Figure S58. ^{1}H NMR spectrum of **3o-C₂** (600 MHz) in $CDCl_3$.

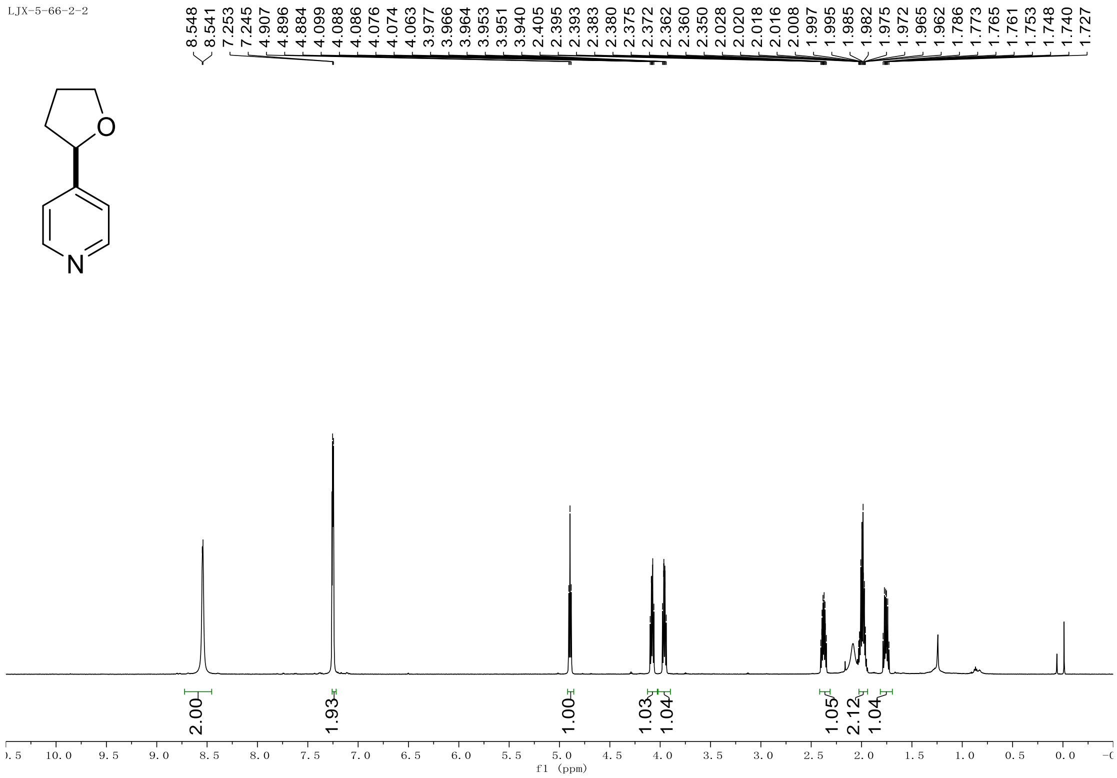

Figure S59. ^{1}H NMR spectrum of **3o-C$_4$** (600 MHz) in $CDCl_3$.

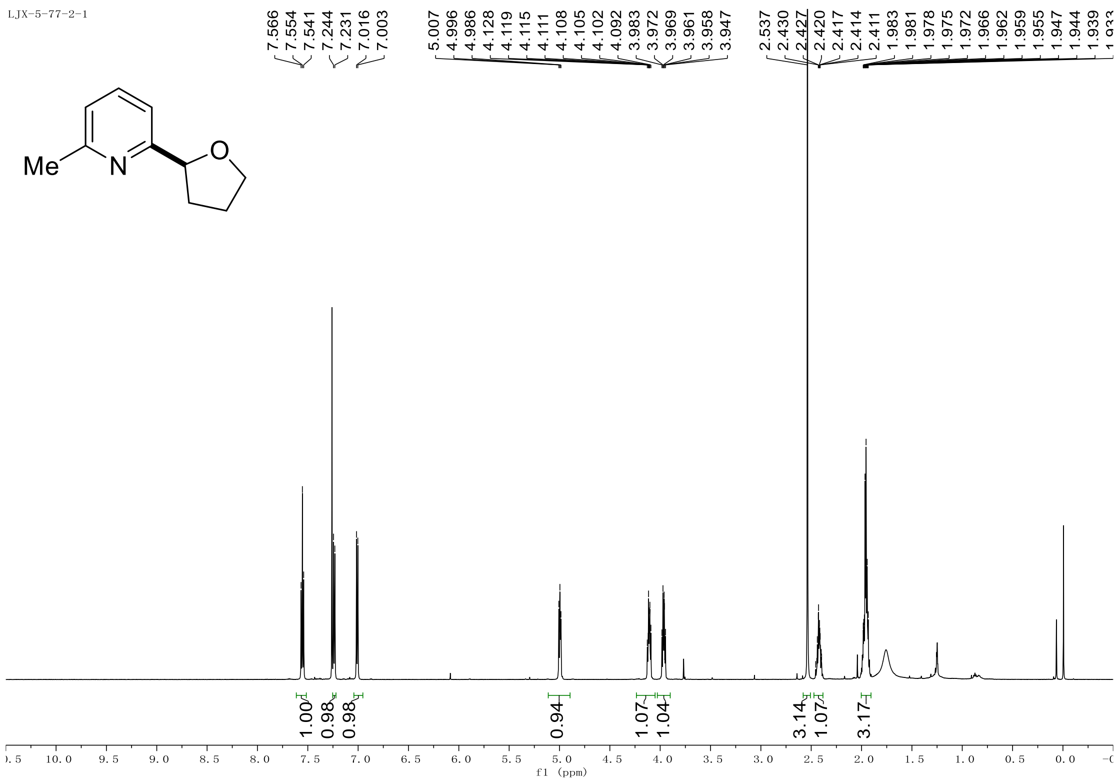

Figure S60. ^{1}H NMR spectrum of **3p-C$_2$** (600 MHz) in $CDCl_3$.

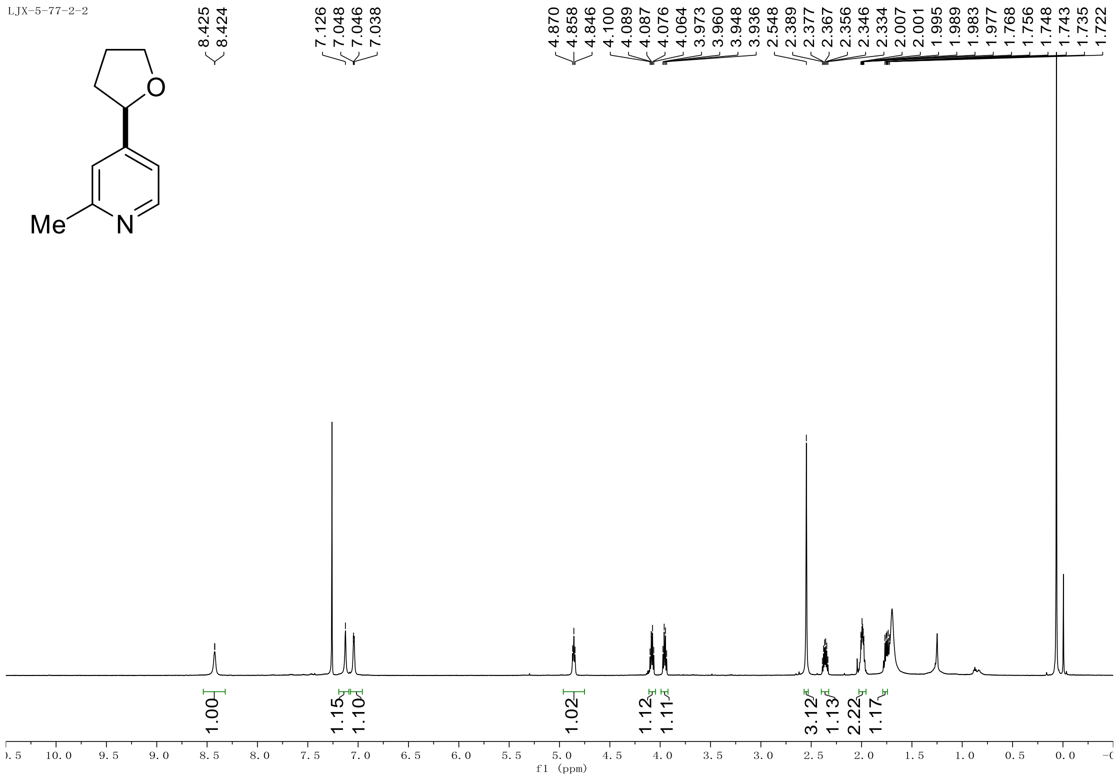

Figure S61. ^{1}H NMR spectrum of **3p-C4** (600 MHz) in $CDCl_3$.

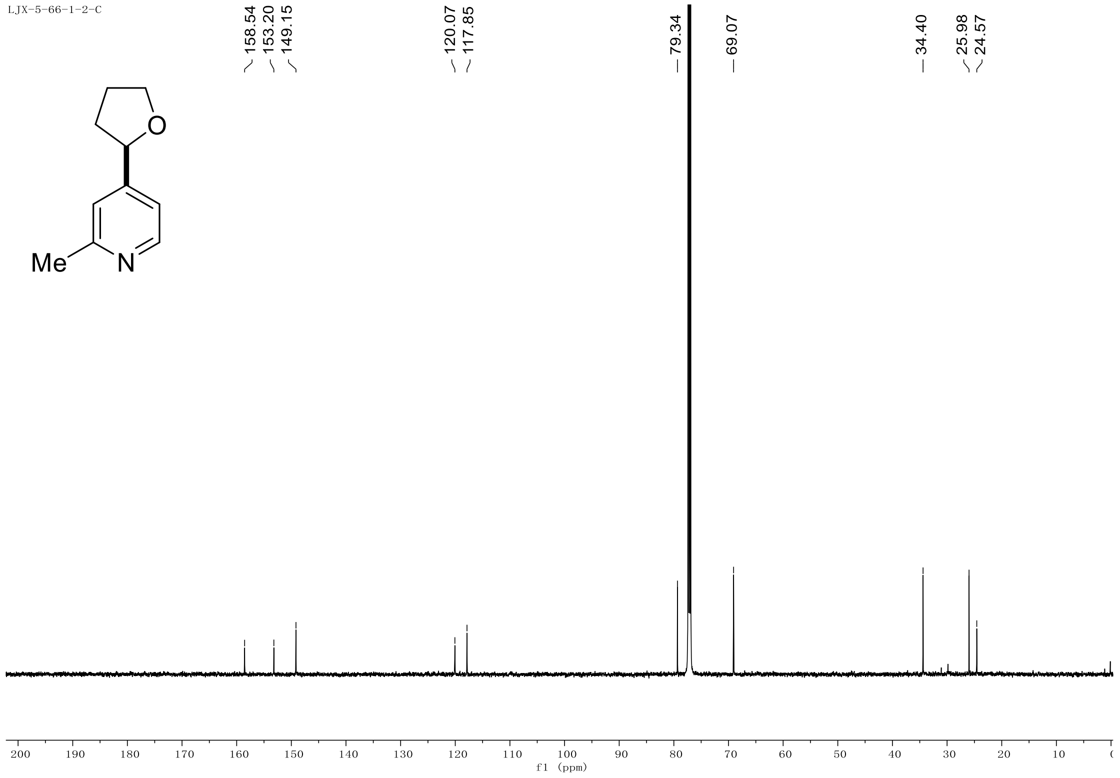

Figure S62. ^{13}C NMR spectrum of **3p-C4** (105 MHz) in $CDCl_3$.

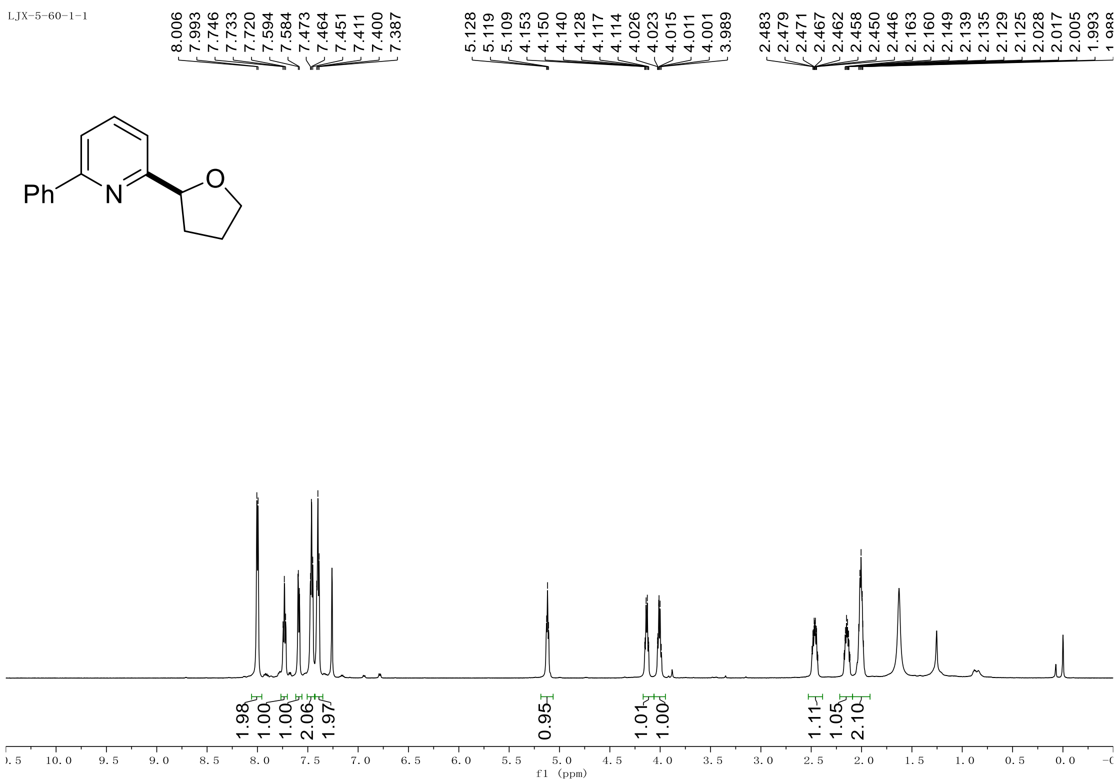

Figure S63. ^{1}H NMR spectrum of **3q-C$_2$** (600 MHz) in $CDCl_3$.

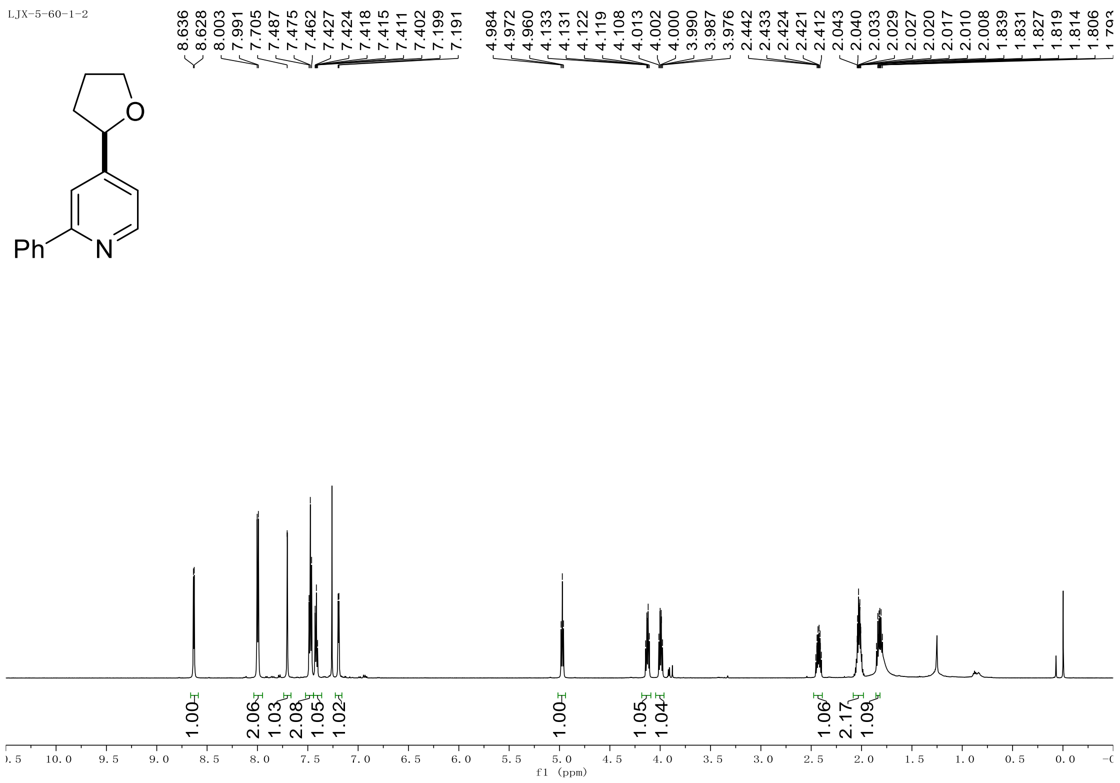

Figure S64. ^{1}H NMR spectrum of **3q-C$_4$** (600 MHz) in $CDCl_3$.

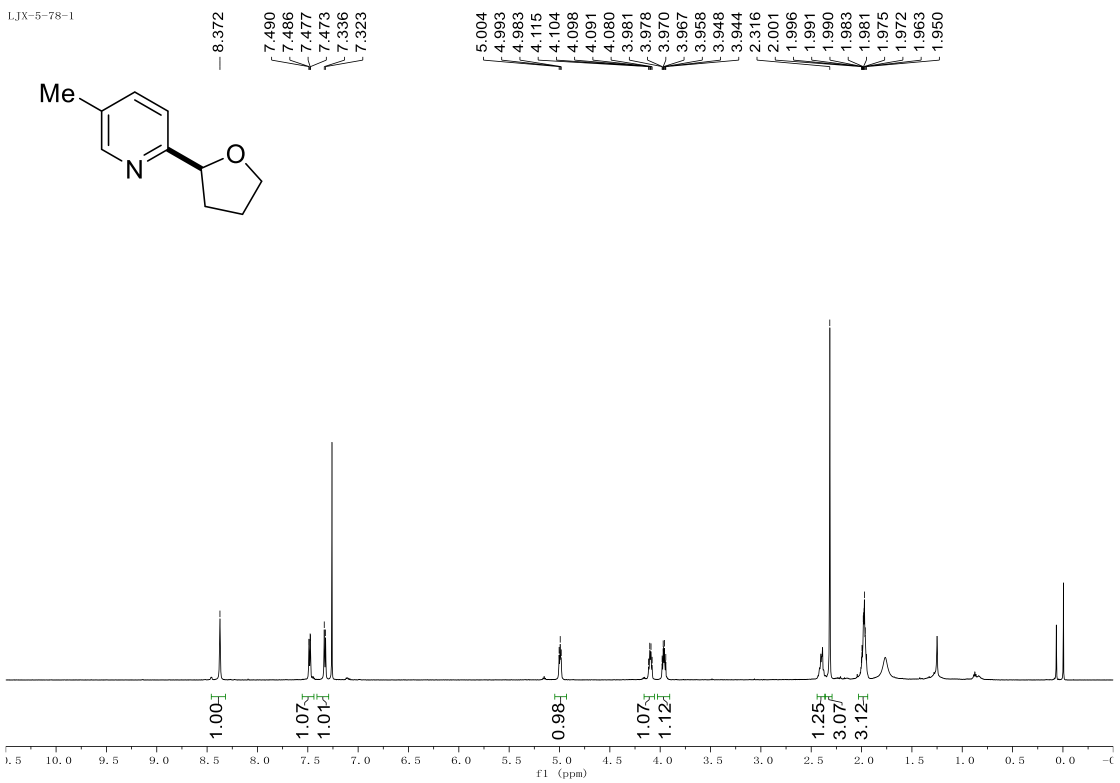

Figure S65. ^{1}H NMR spectrum of **3r-C$_2$** (600 MHz) in $CDCl_3$.

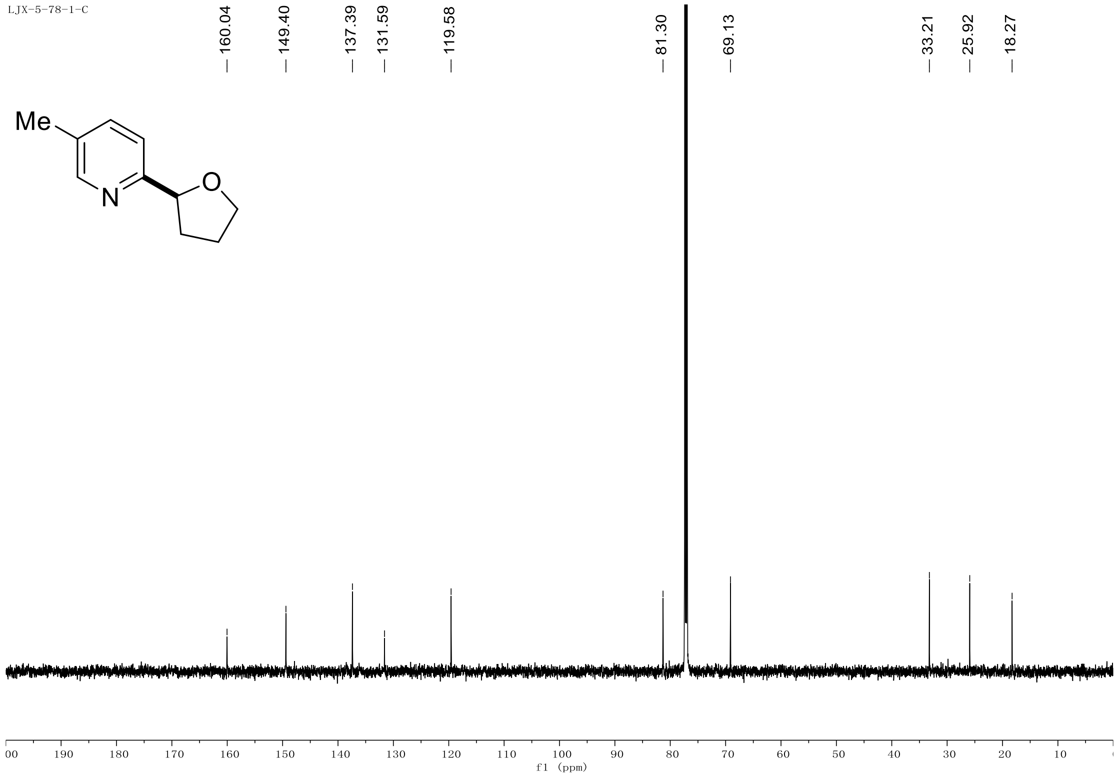

Figure S66. ^{13}C NMR spectrum of **3r-C$_2$** (105 MHz) in $CDCl_3$.

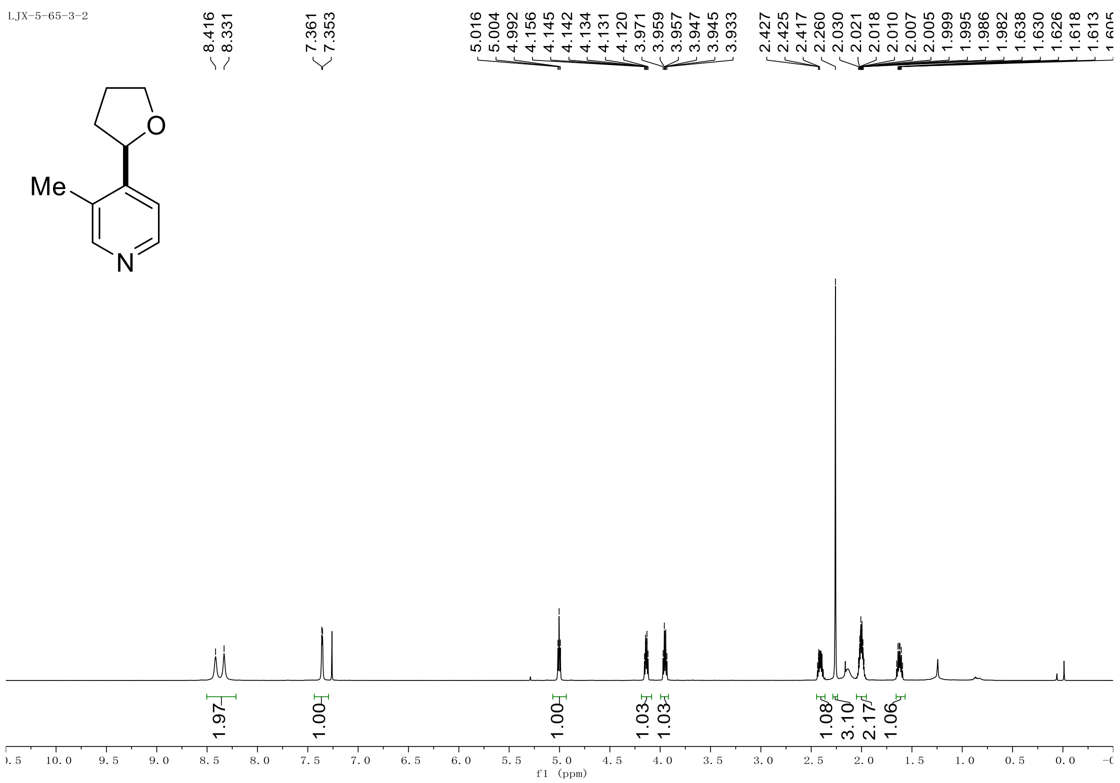

Figure S67. ^{1}H NMR spectrum of **3r-C**$_4$ (600 MHz) in $CDCl_3$.

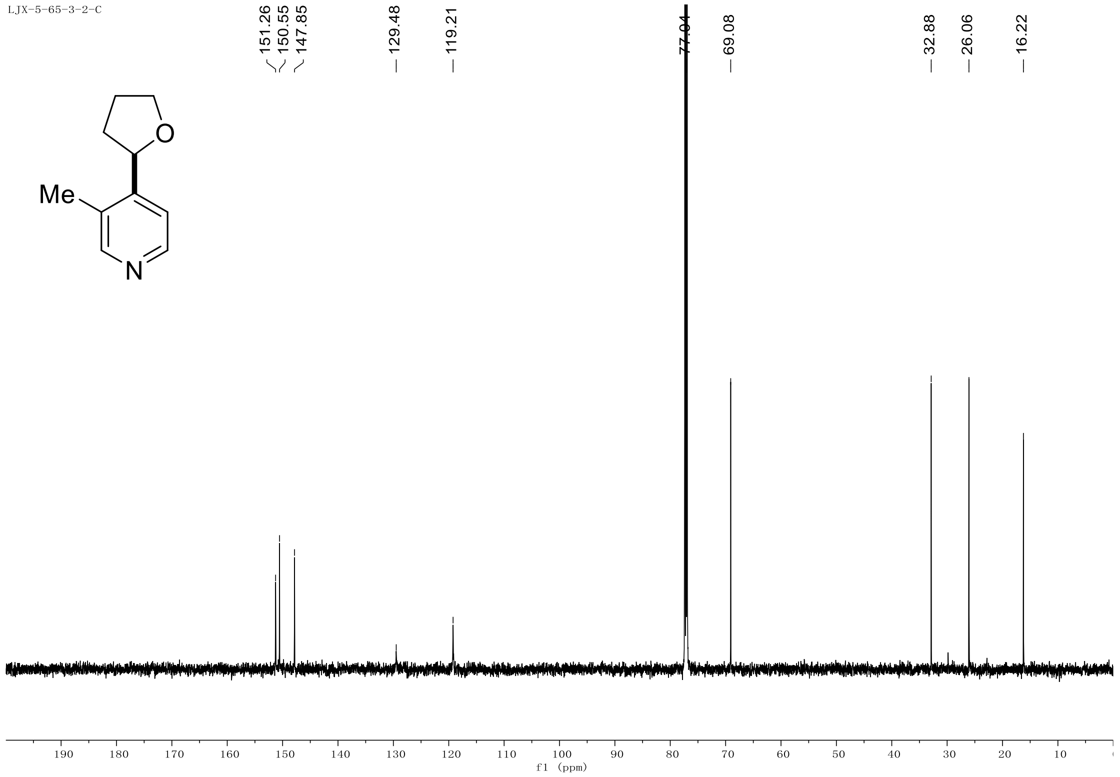

Figure S68. ^{13}C NMR spectrum of **3r-C**$_4$ (105 MHz) in $CDCl_3$.

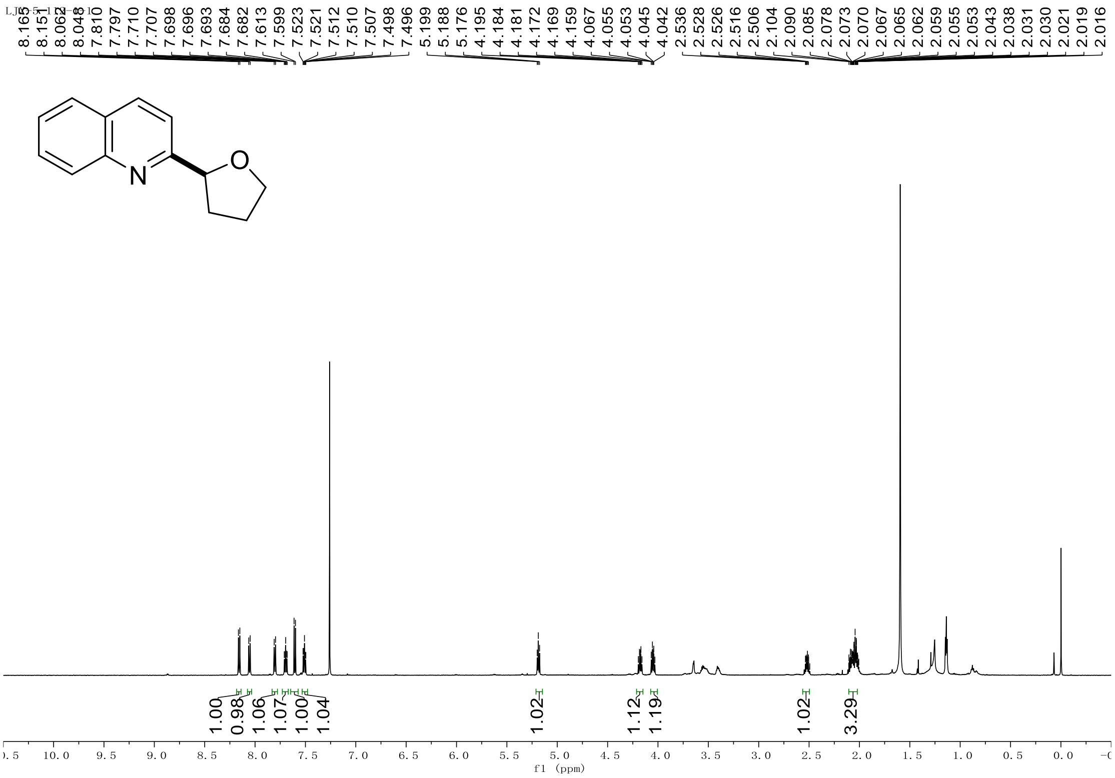

Figure S69. ^{1}H NMR spectrum of **3s-C$_2$** (600 MHz) in $CDCl_3$.

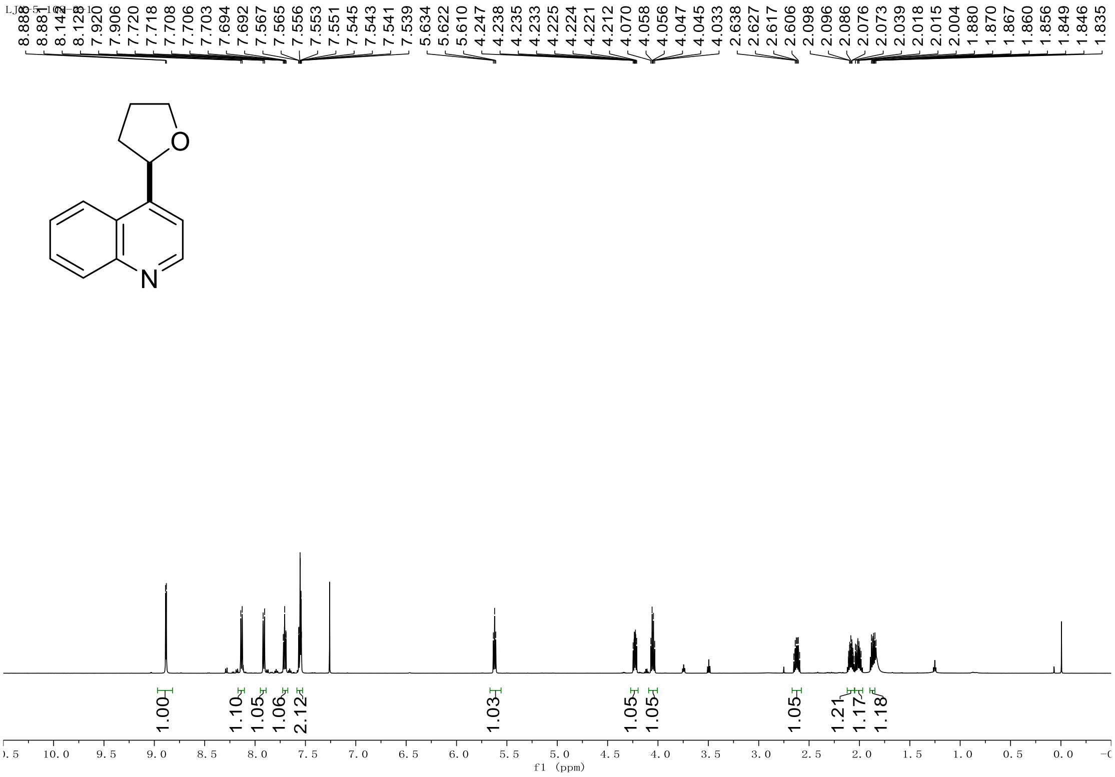

Figure S70. ^{1}H NMR spectrum of **3s-C$_4$** (600 MHz) in $CDCl_3$.

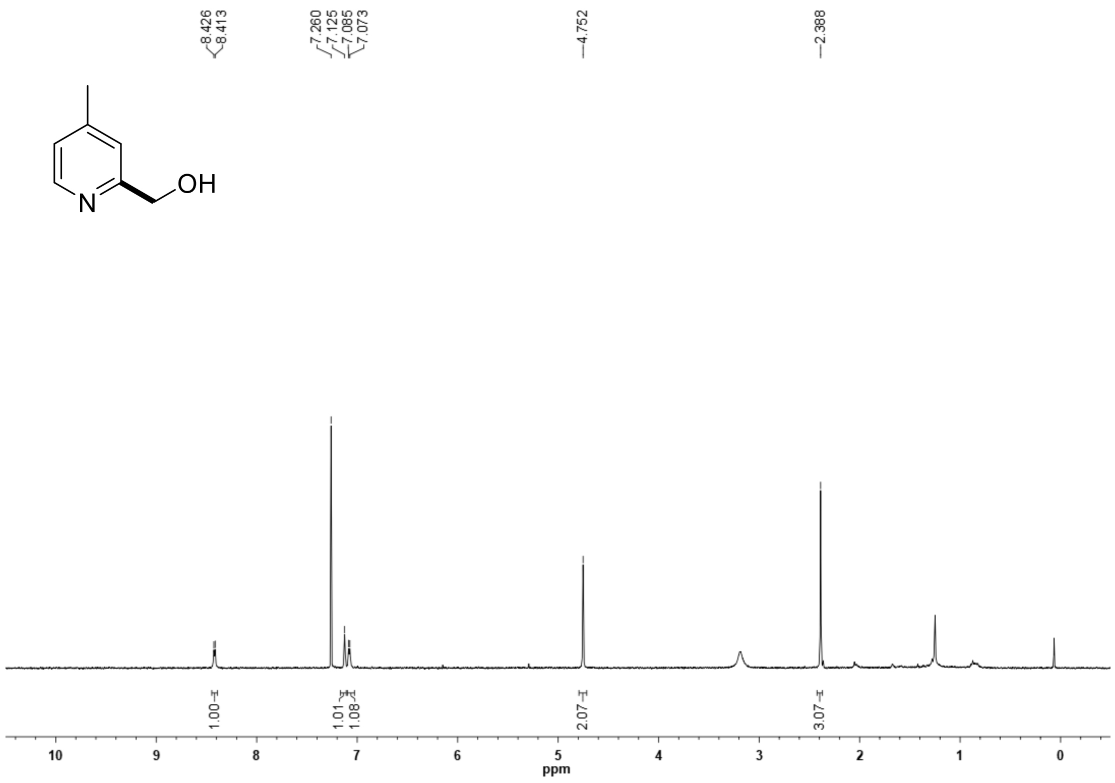

Figure S71. ^{1}H NMR spectrum of **3t** (400 MHz) in $CDCl_3$.

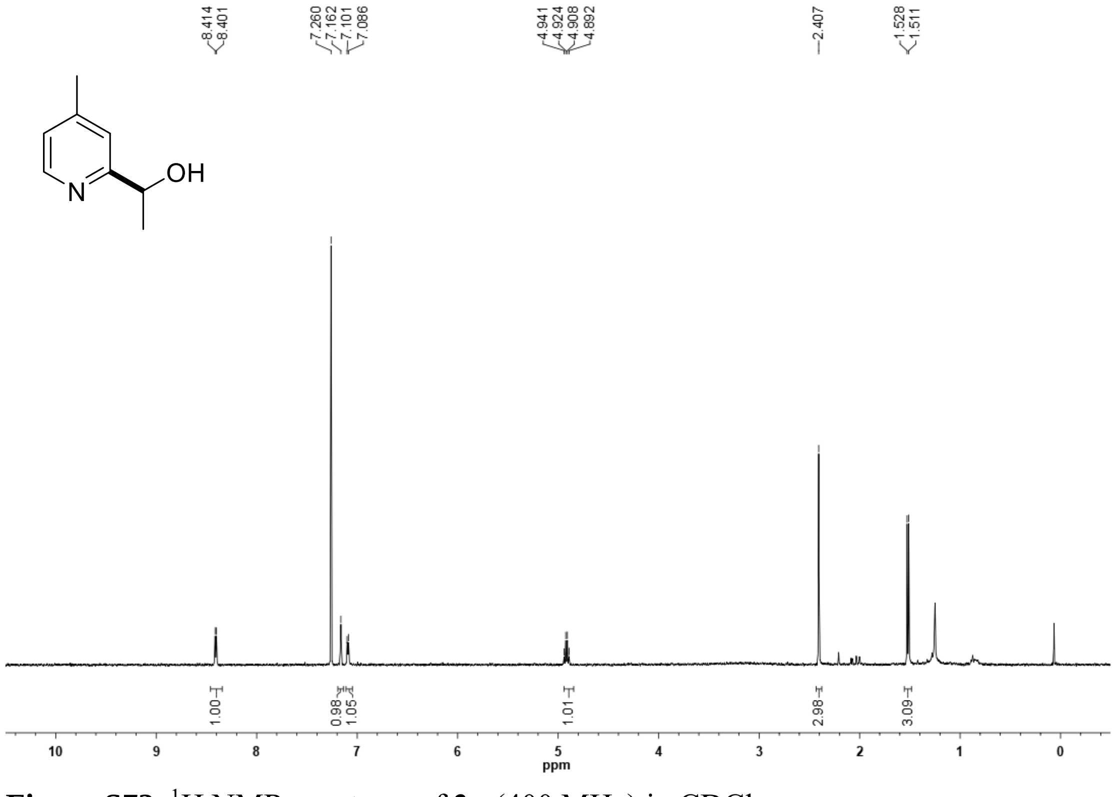

Figure S72. ^{1}H NMR spectrum of **3u** (400 MHz) in $CDCl_3$.

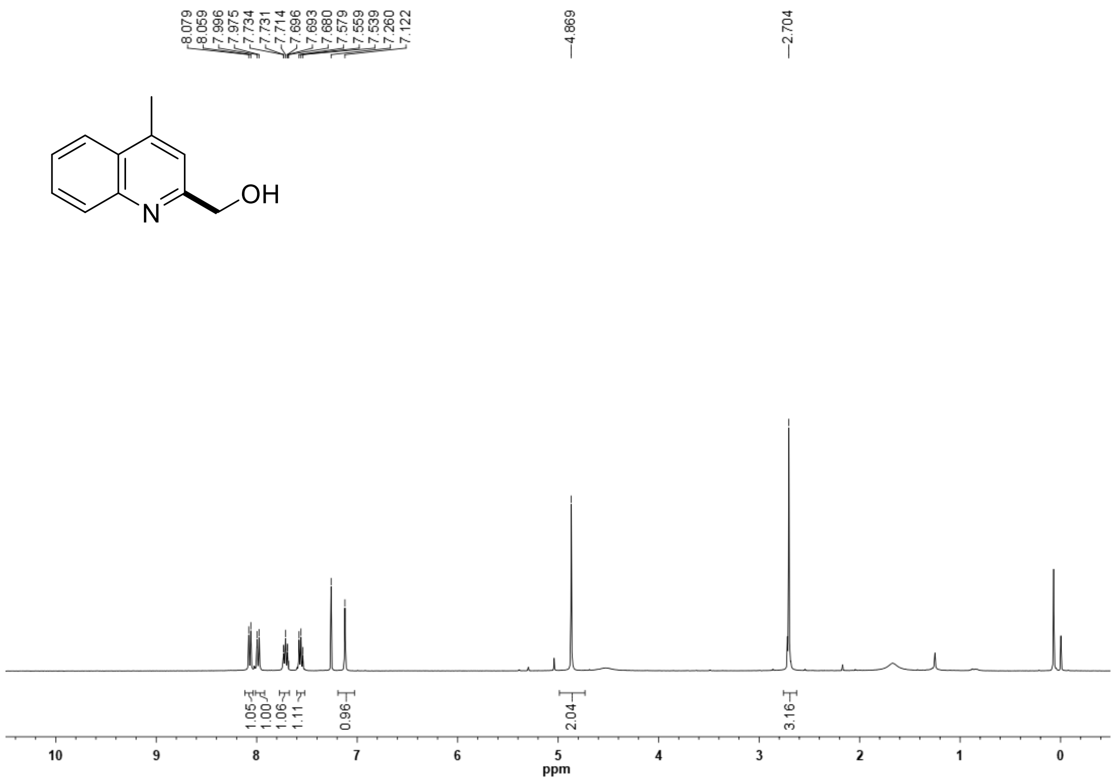

Figure S73. ^{1}H NMR spectrum of **3v** (400 MHz) in $CDCl_3$.

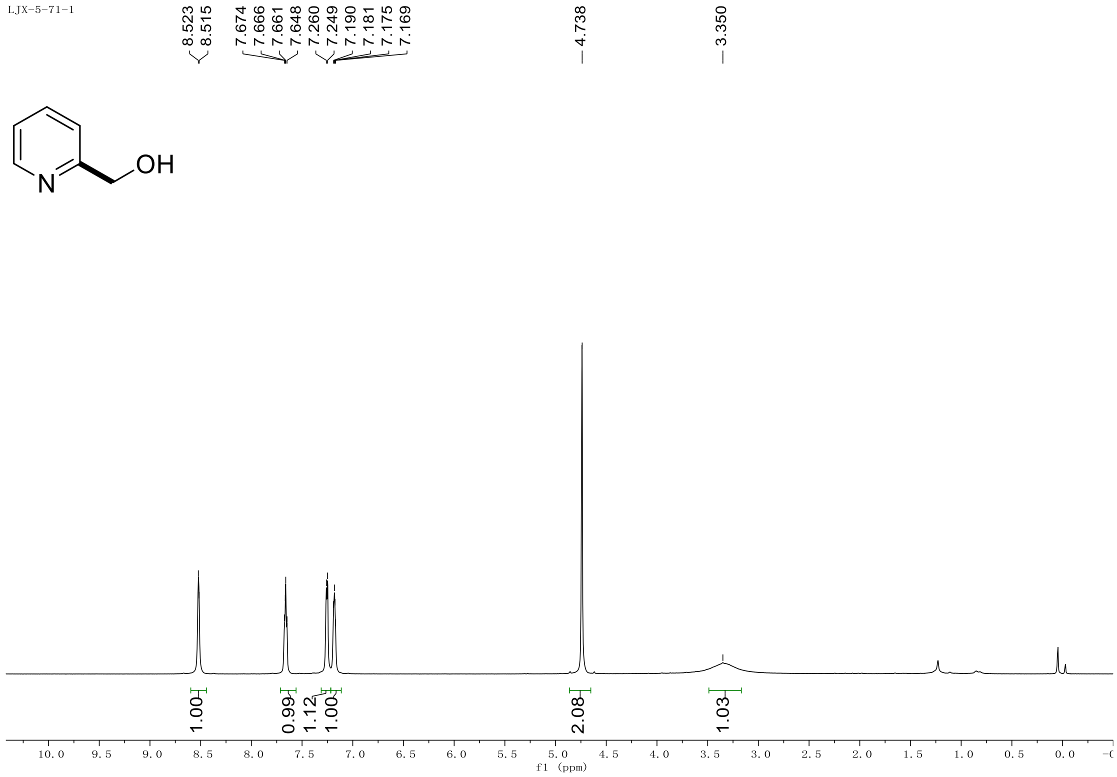

Figure S74. ^{1}H NMR spectrum of **3w-C$_2$** (600 MHz) in $CDCl_3$.

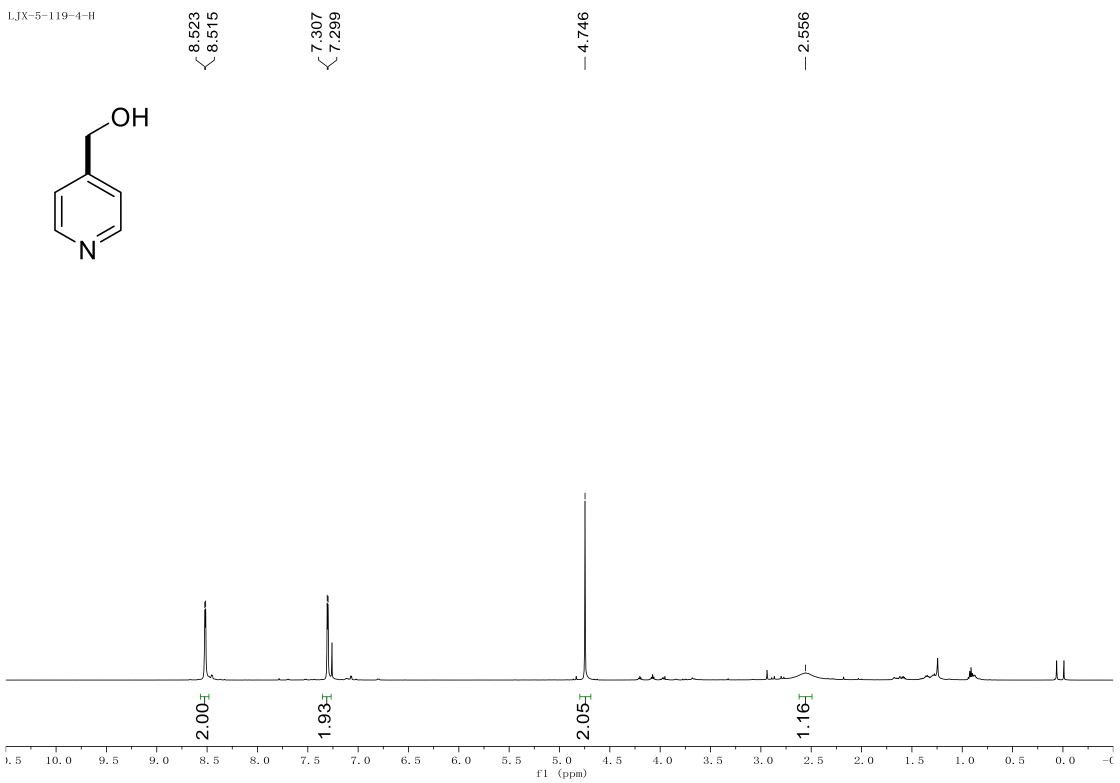

Figure S75. ¹H NMR spectrum of **3w-C**$_4$ (600 MHz) in $CDCl_3$.

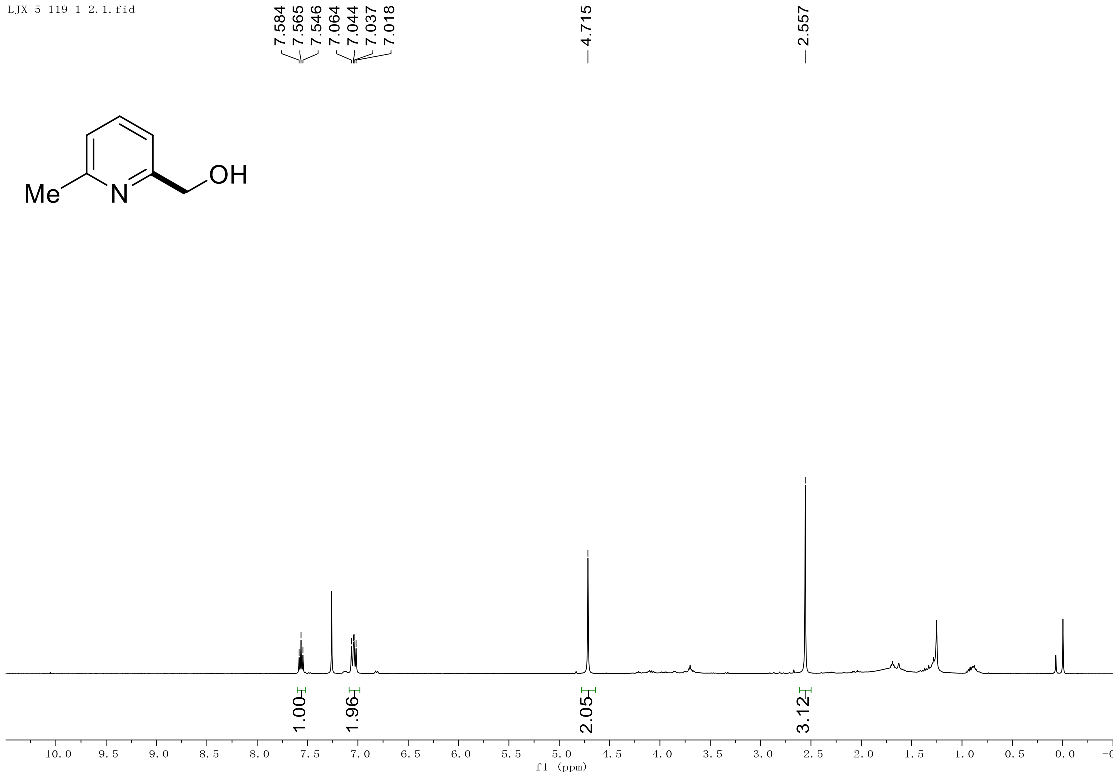

Figure S76. ¹H NMR spectrum of **3x-C**$_2$ (400 MHz) in $CDCl_3$.

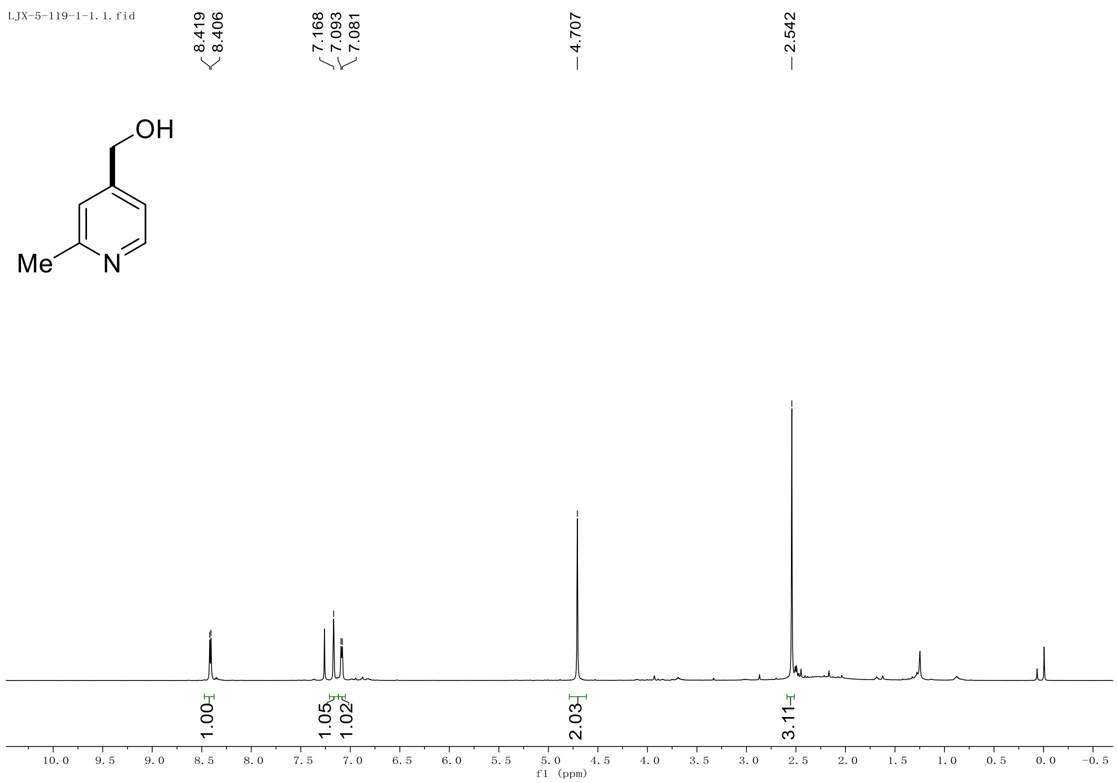

Figure S77. ^{1}H NMR spectrum of **3x-C4** (400 MHz) in $CDCl_3$.

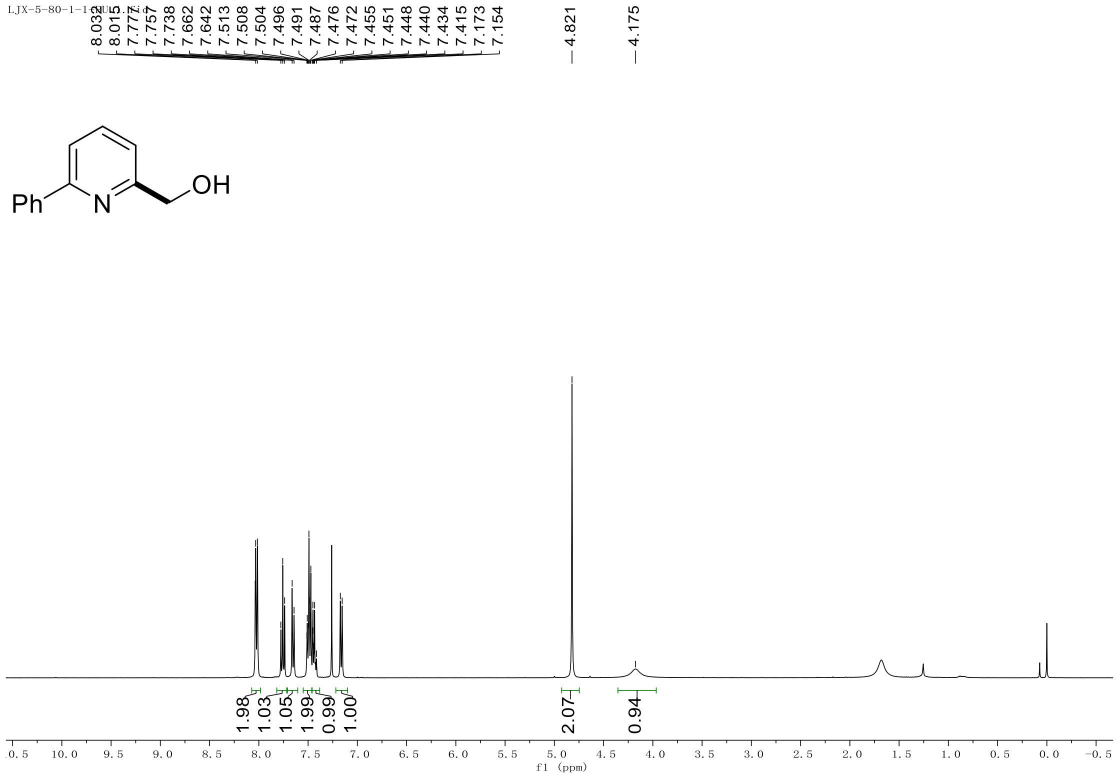

Figure S78. ^{1}H NMR spectrum of **3y-C2** (400 MHz) in $CDCl_3$.

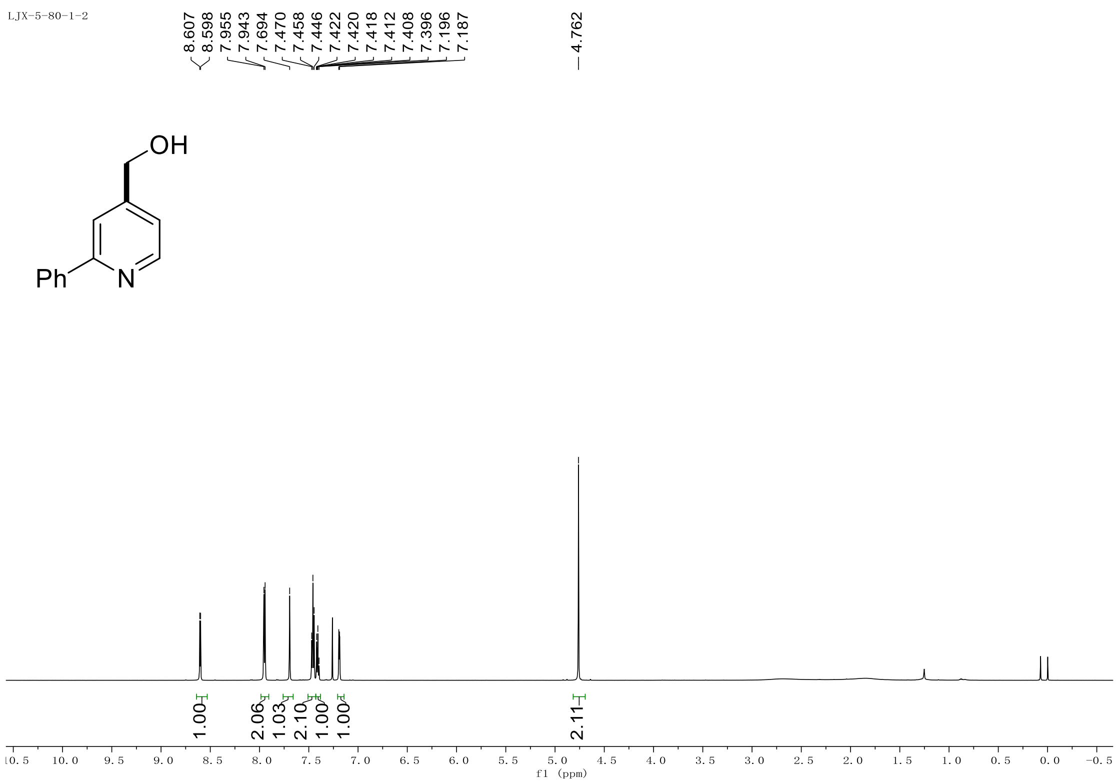

Figure S79. ^{1}H NMR spectrum of **3y-C4** (600 MHz) in $CDCl_3$.

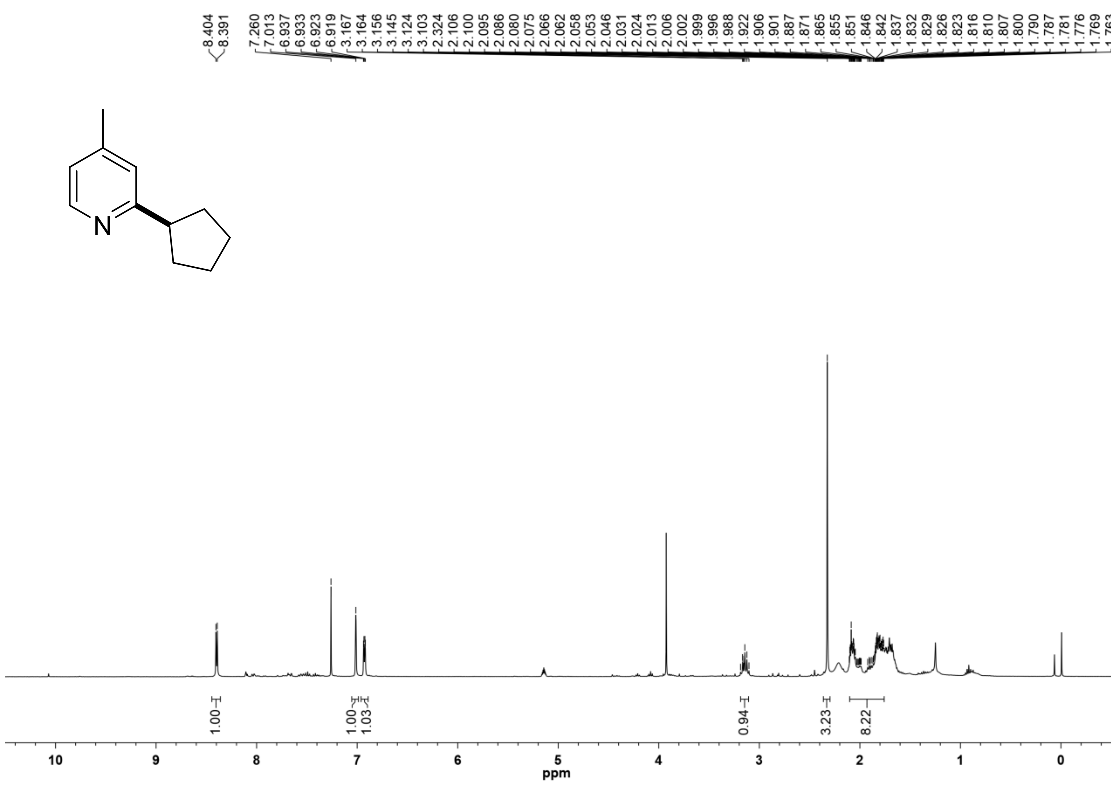

Figure S80. ^{1}H NMR spectrum of **3z** (400 MHz) in $CDCl_3$.

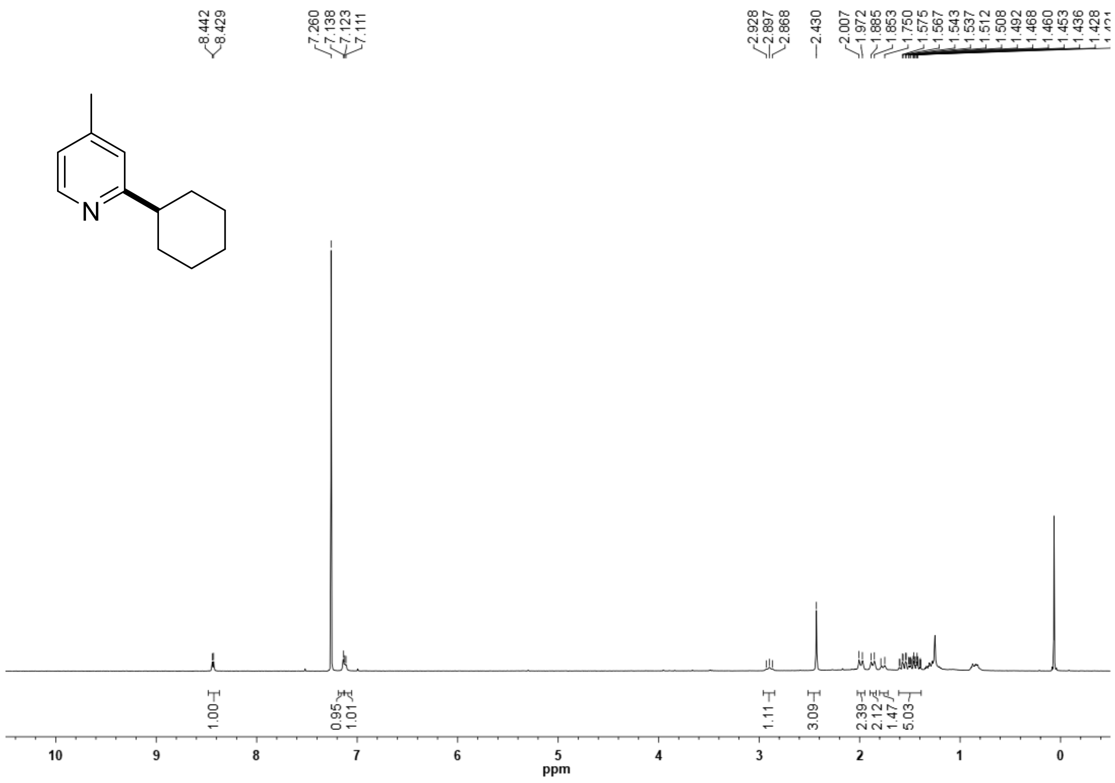

Figure S81. ^{1}H NMR spectrum of **3aa** (400 MHz) in $CDCl_3$.

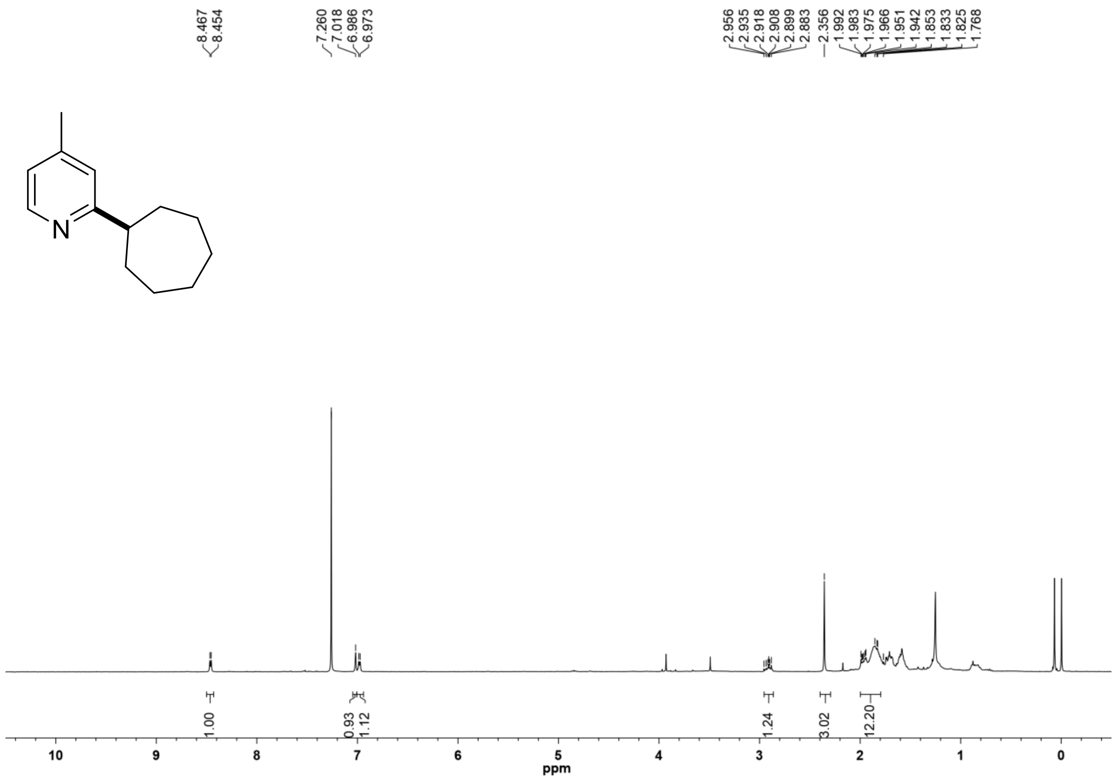

Figure S82. ^{1}H NMR spectrum of **3ab** (400 MHz) in $CDCl_3$.

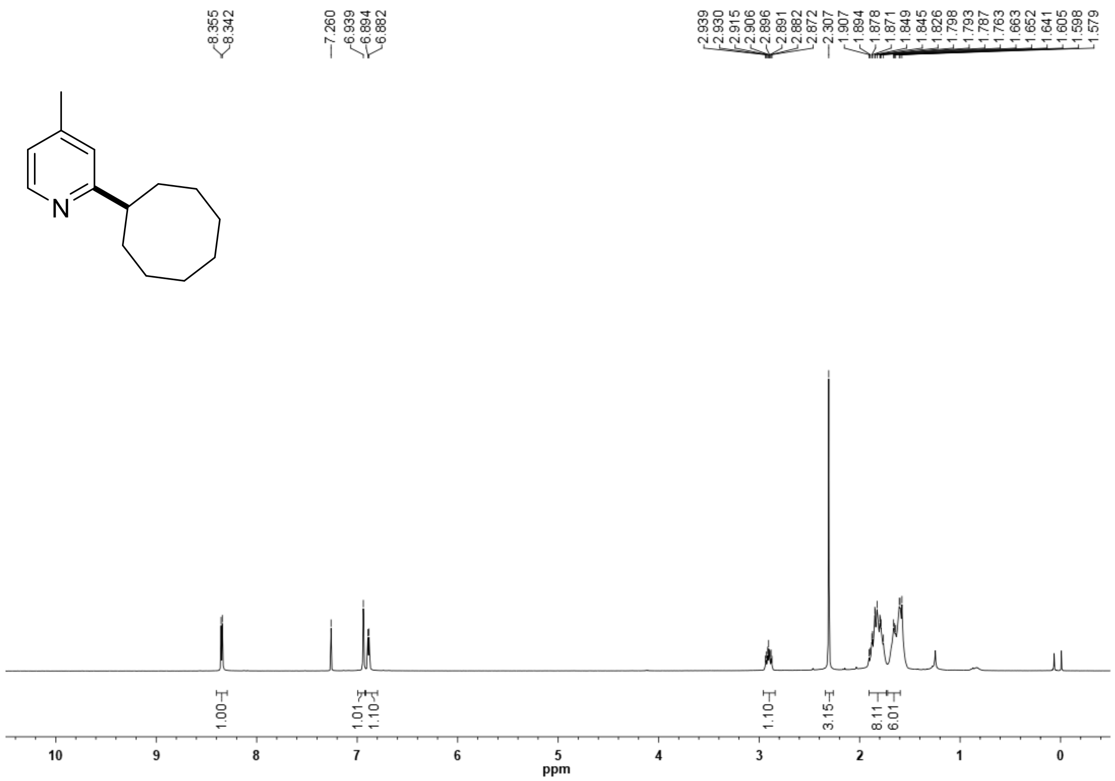

Figure S83. ^{1}H NMR spectrum of **3ac** (400 MHz) in $CDCl_3$.

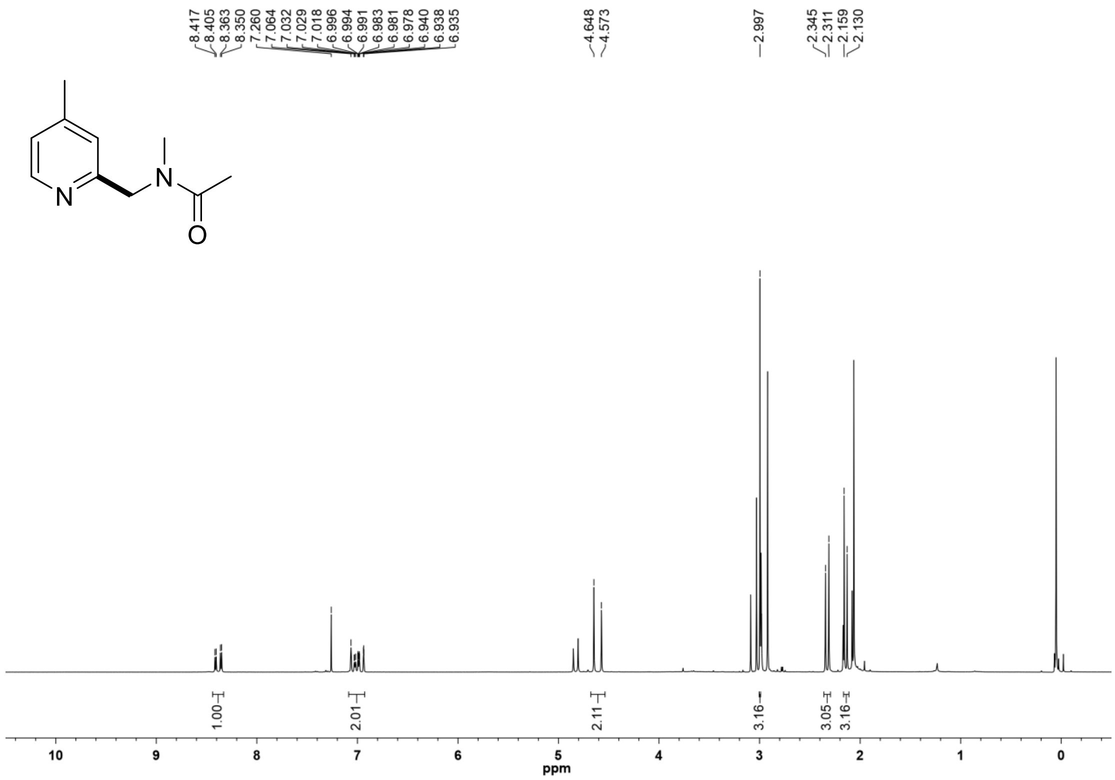

Figure S84. ^{1}H NMR spectrum of **3ad** (400 MHz) in $CDCl_3$.

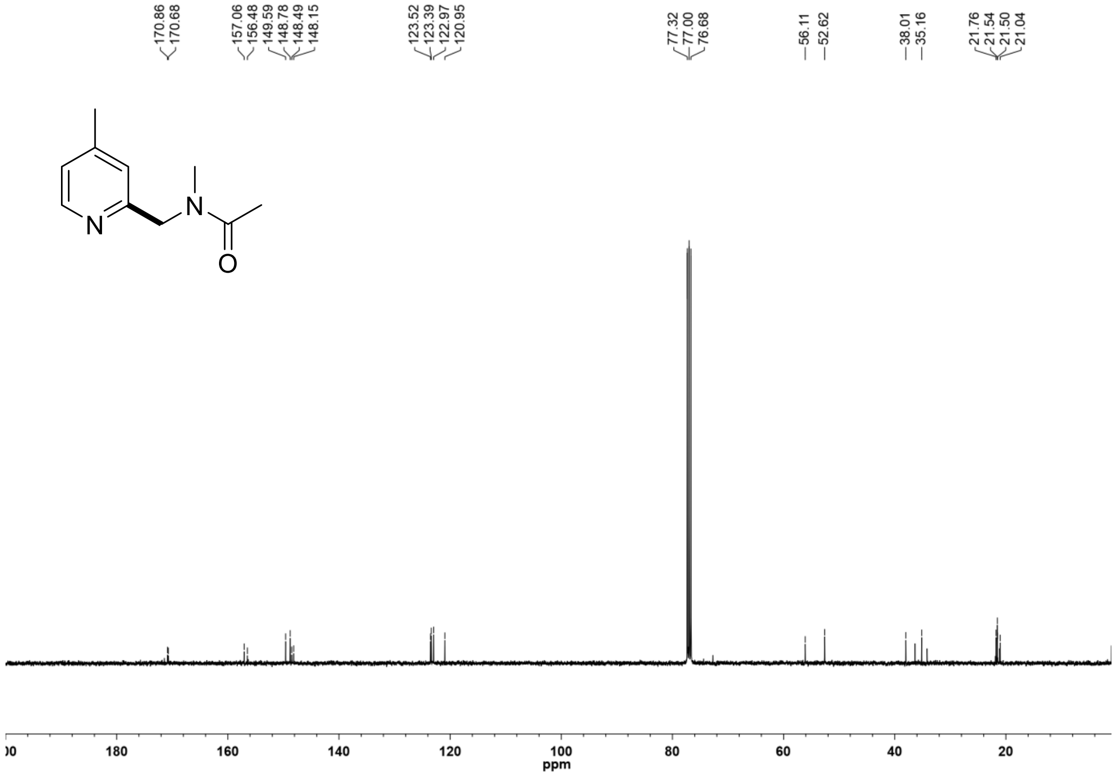

Figure S85. ^{13}C NMR spectrum of **3ad** (100 MHz) in $CDCl_3$.

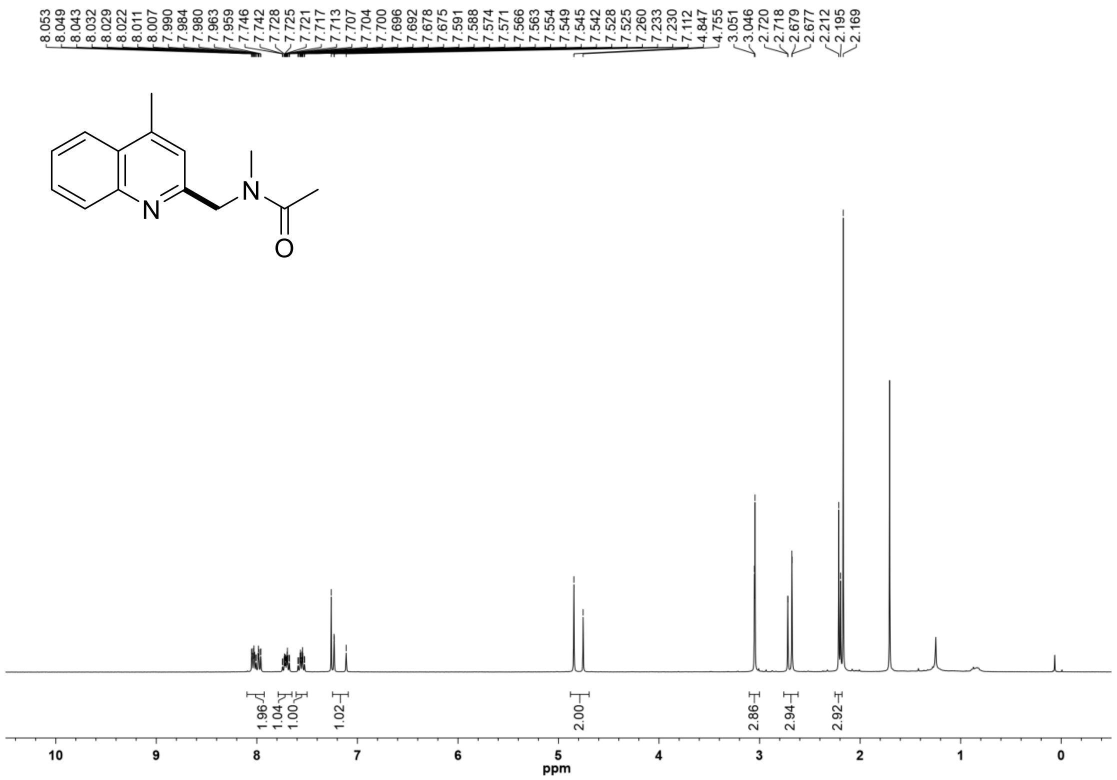

Figure S86. ^{1}H NMR spectrum of **3ae** (100 MHz) in $CDCl_3$.

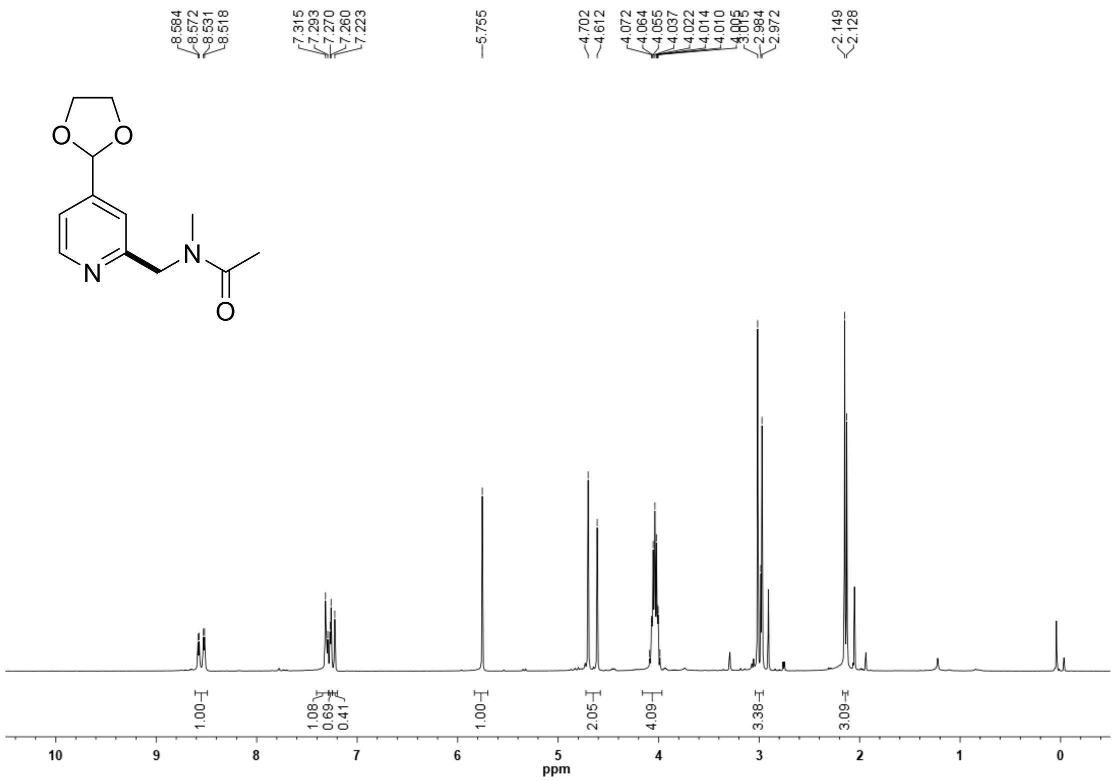

Figure S87. ^{1}H NMR spectrum of **3af** (400 MHz) in $CDCl_3$.

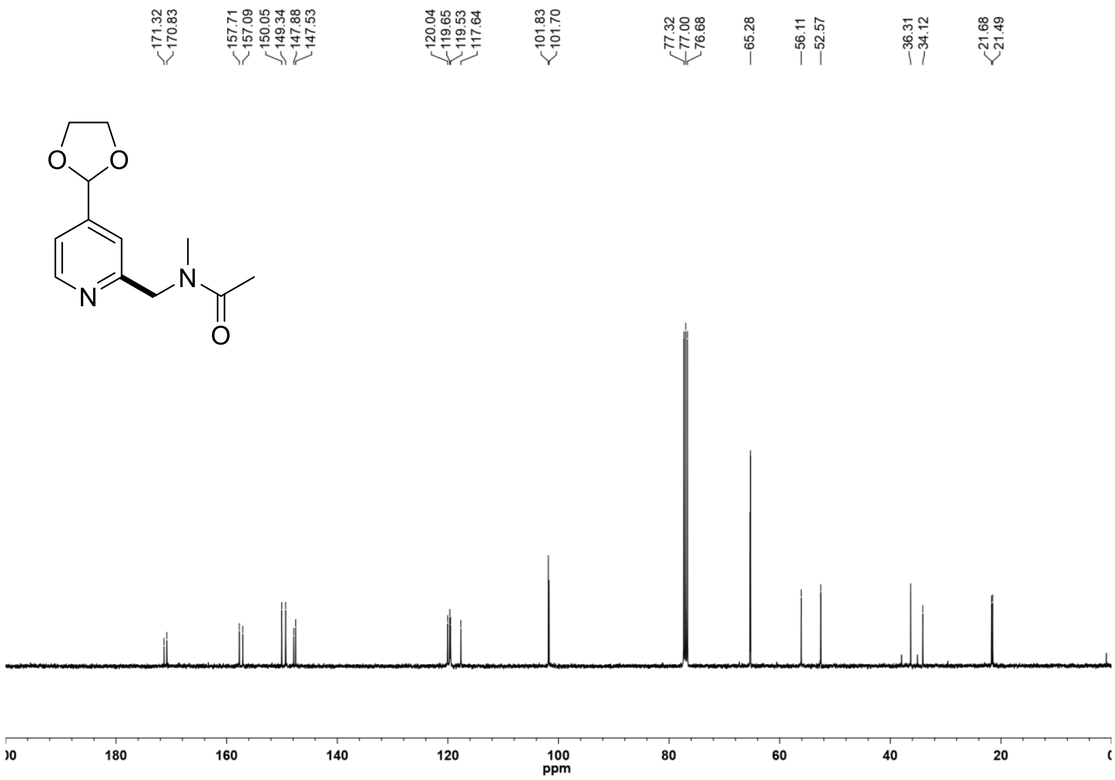

Figure S88. ^{13}C NMR spectrum of **3af** (100 MHz) in $CDCl_3$.

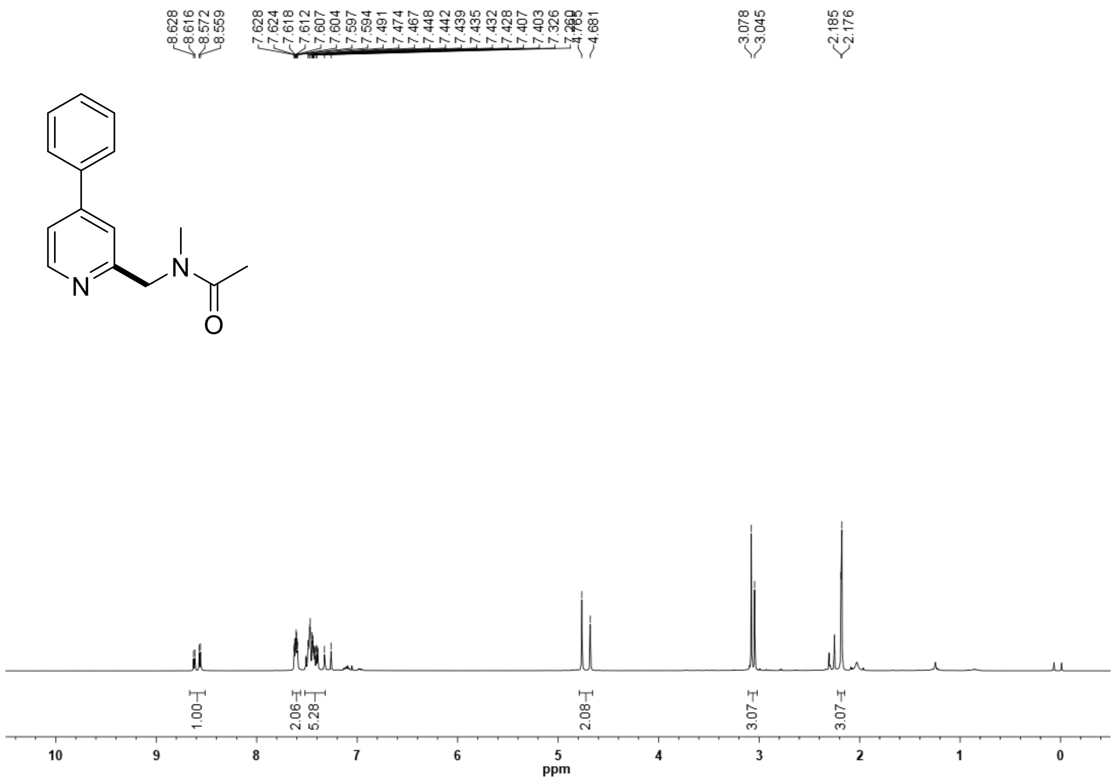

Figure S89. ^{1}H NMR spectrum of **3ag** (400 MHz) in $CDCl_3$.

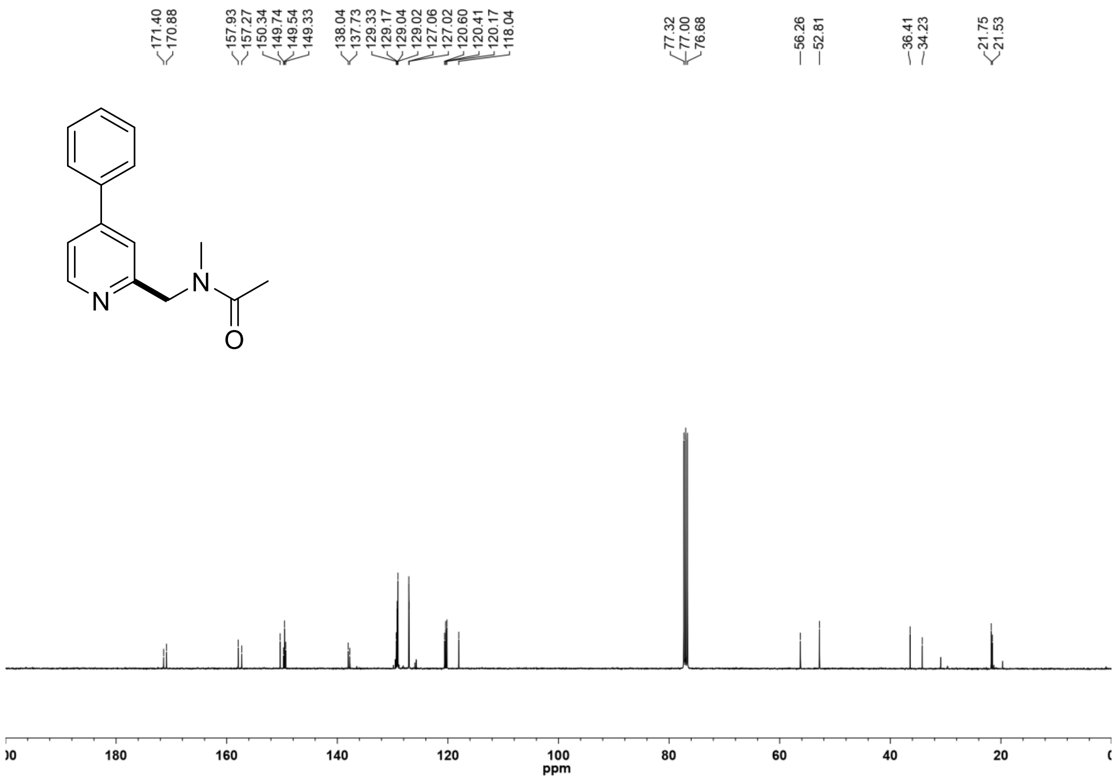

Figure S90. ^{13}C NMR spectrum of **3ag** (100 MHz) in $CDCl_3$.

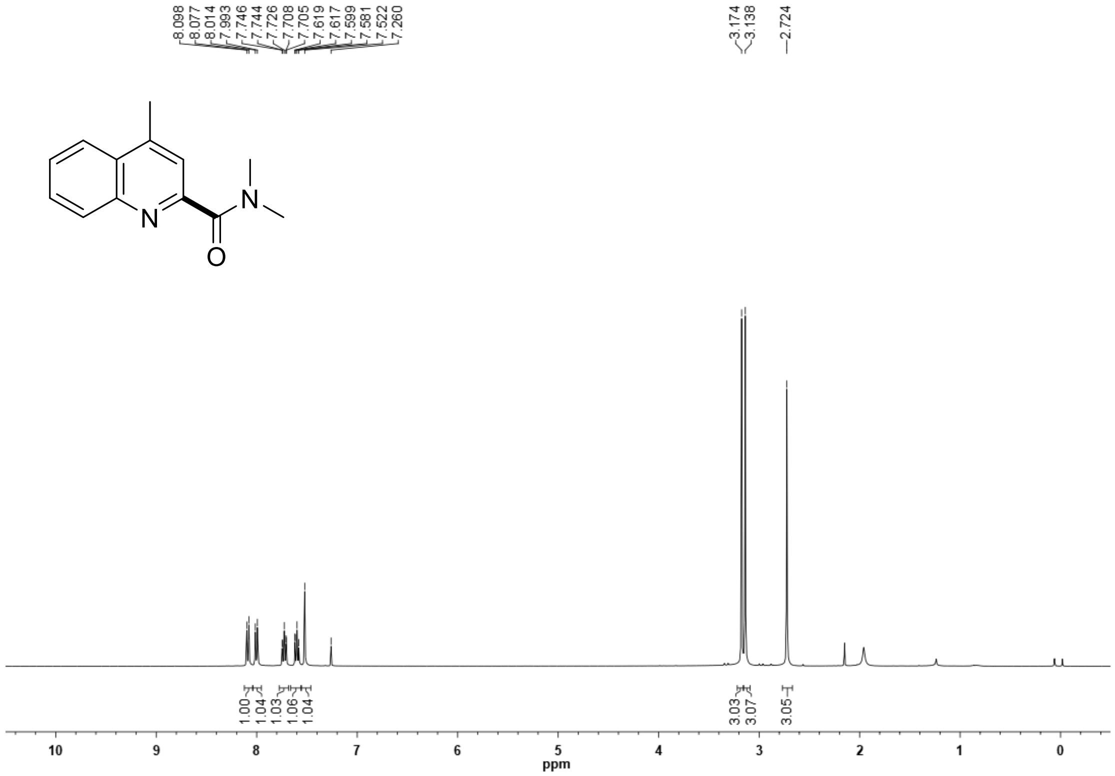

Figure S91. ^{1}H NMR spectrum of **3ah** (400 MHz) in $CDCl_3$.

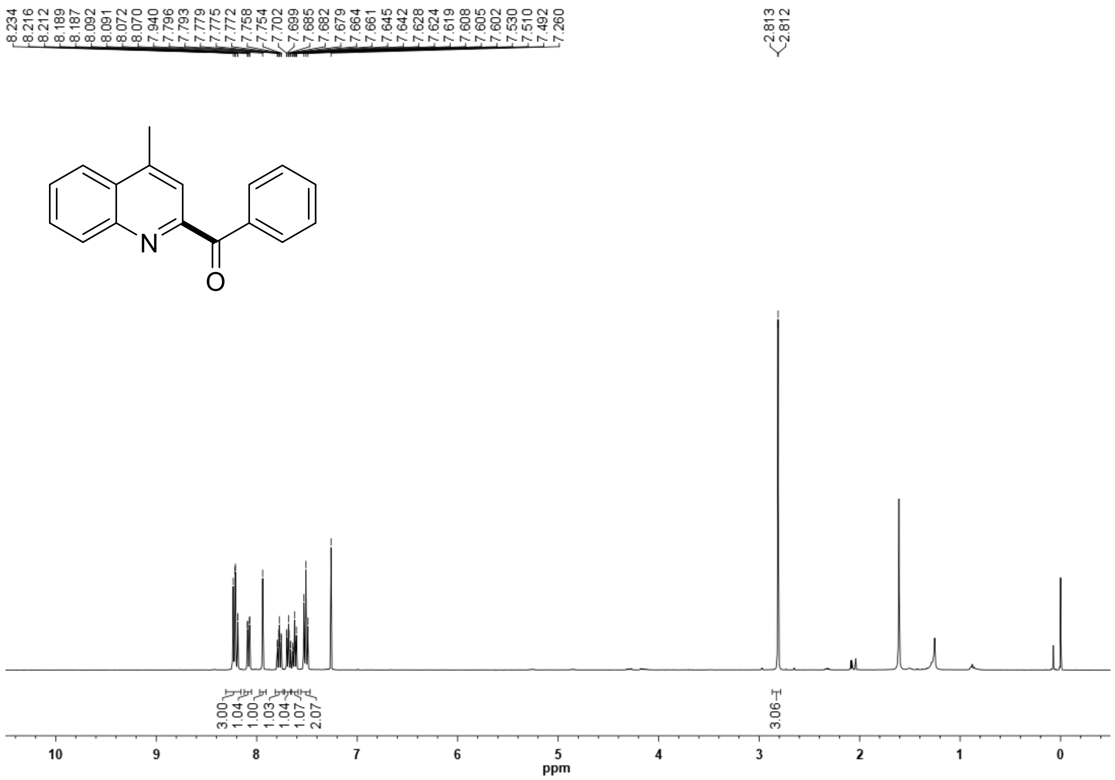

Figure S92. ^{1}H NMR spectrum of **3ai** (400 MHz) in $CDCl_3$.

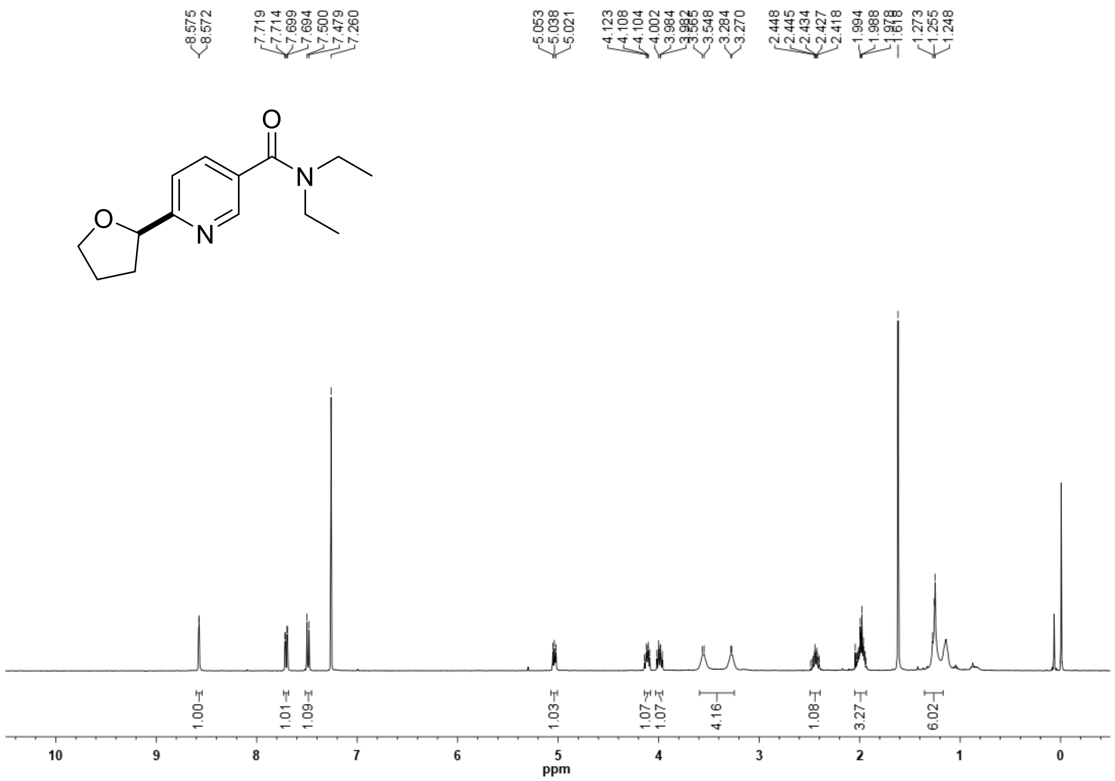

Figure S93. ^{1}H NMR spectrum of **3aj** (400 MHz) in $CDCl_3$.

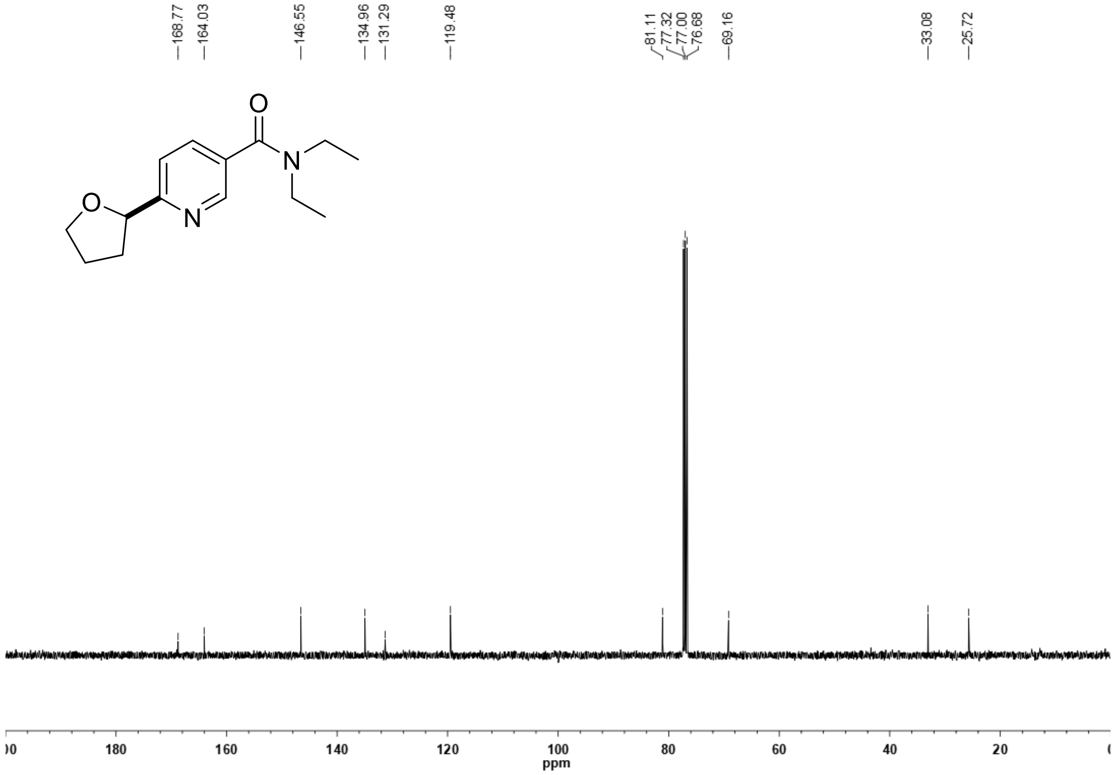

Figure S94.^{13}C NMR spectrum of **3aj** (100 MHz) in $CDCl_3$.

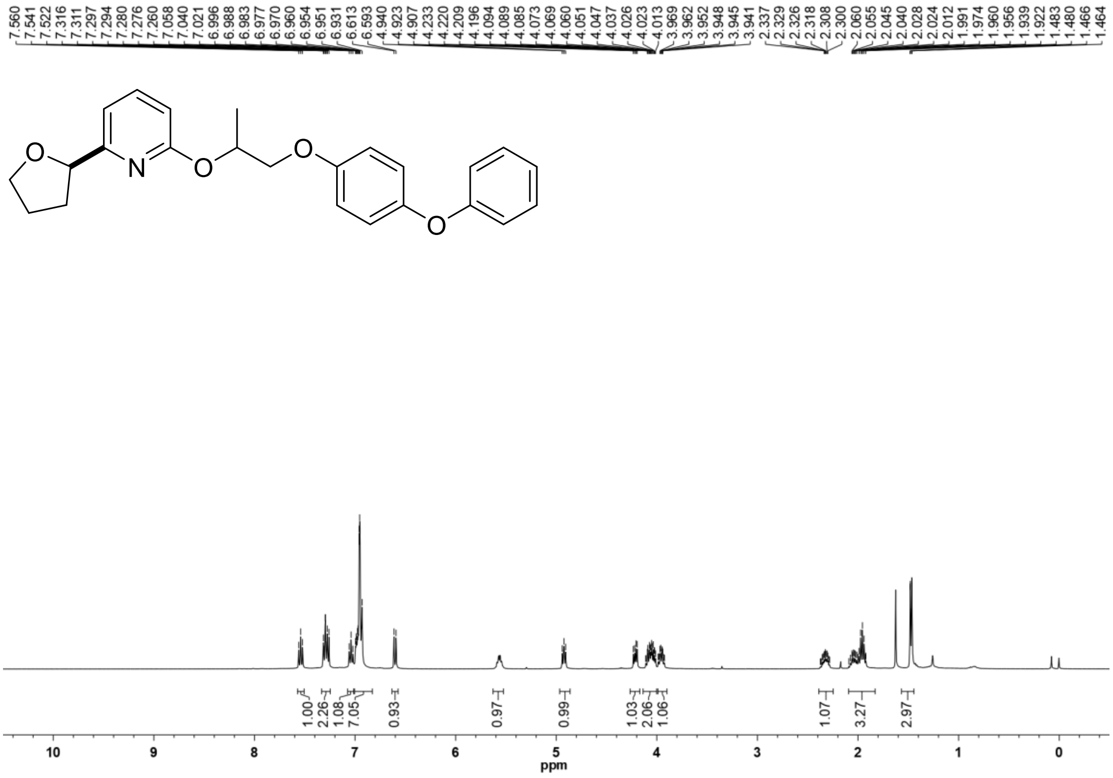

Figure S95. ^{1}H NMR spectrum of **3ak-C$_2$** (400 MHz) in $CDCl_3$.

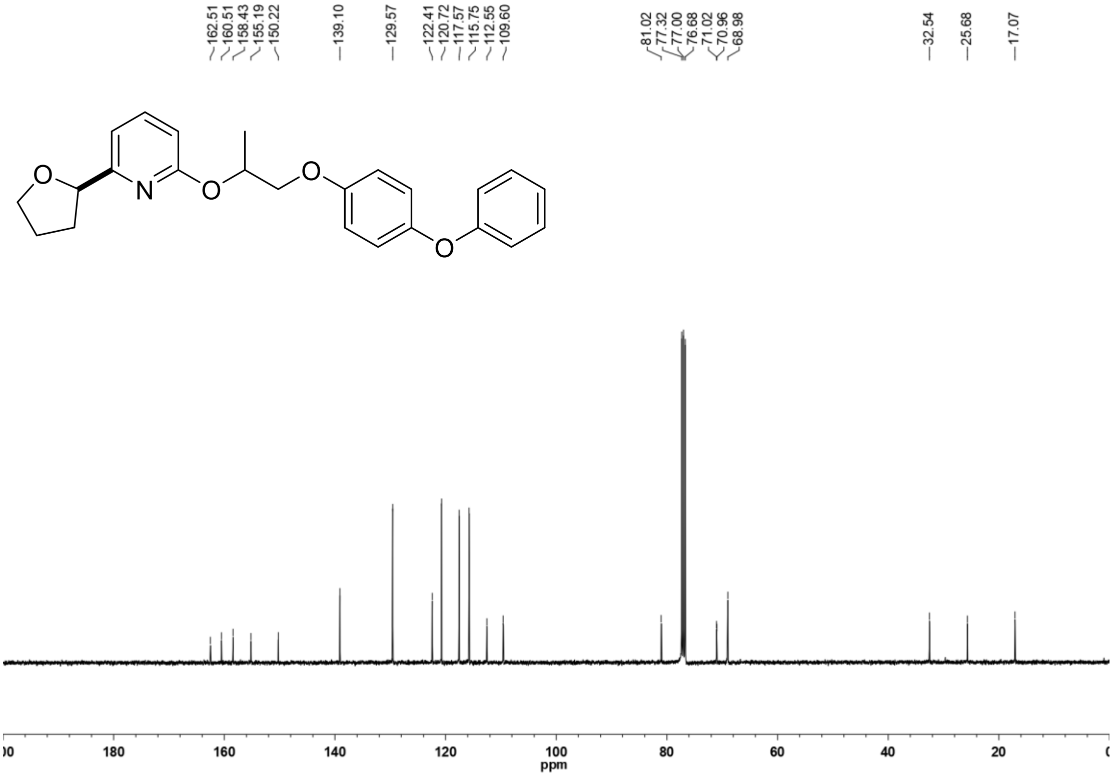

Figure S96. ^{13}C NMR spectrum of **3ak-C$_2$** (100 MHz) in $CDCl_3$.

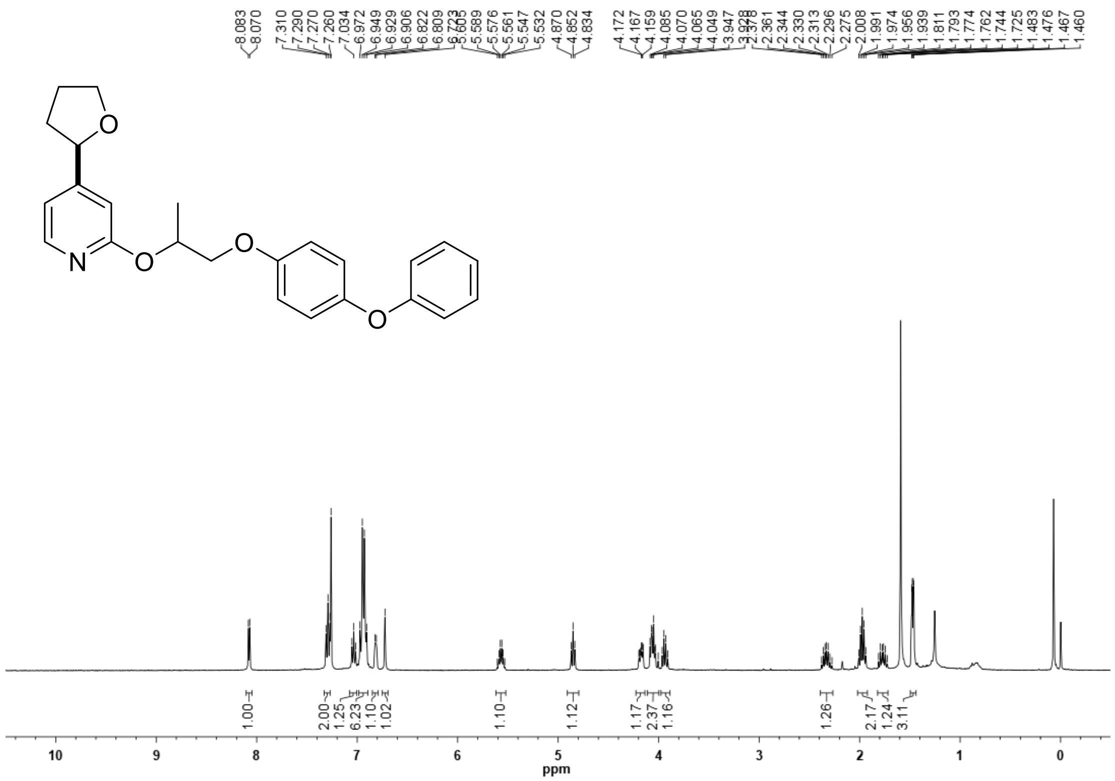

Figure S97. ^{1}H NMR spectrum of **3ak-C$_4$** (400 MHz) in $CDCl_3$.

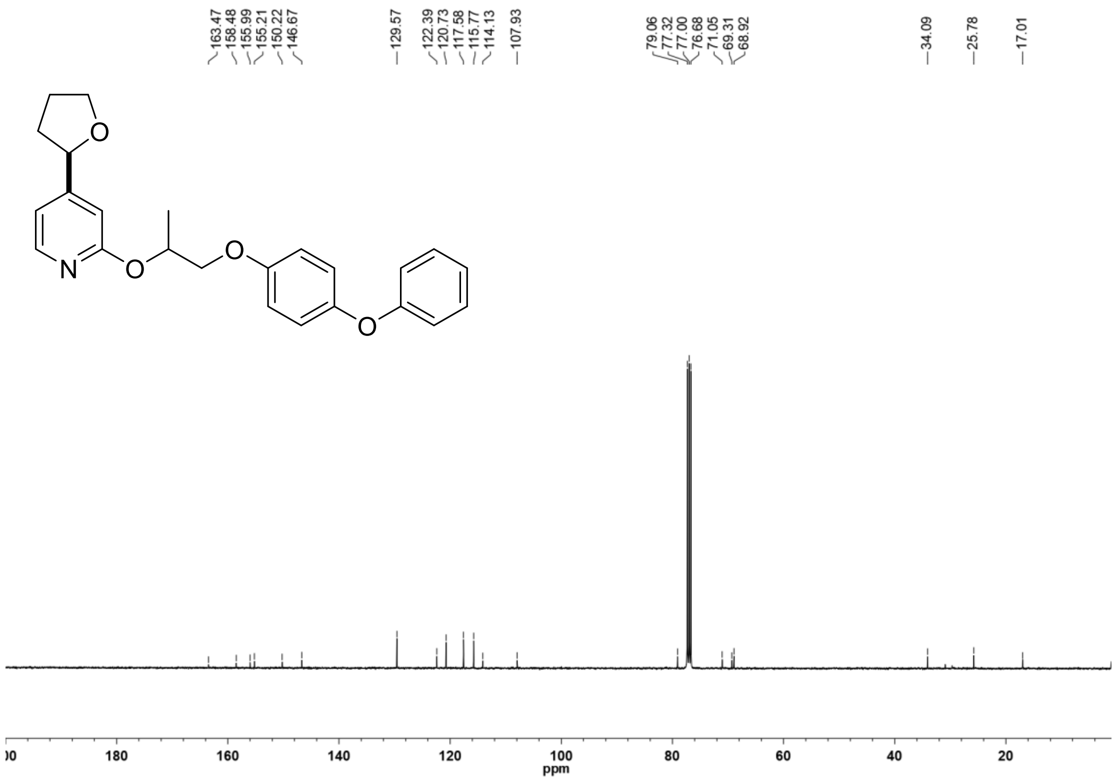

Figure S98. ^{13}C NMR spectrum of **3ak-C$_4$** (100 MHz) in $CDCl_3$.

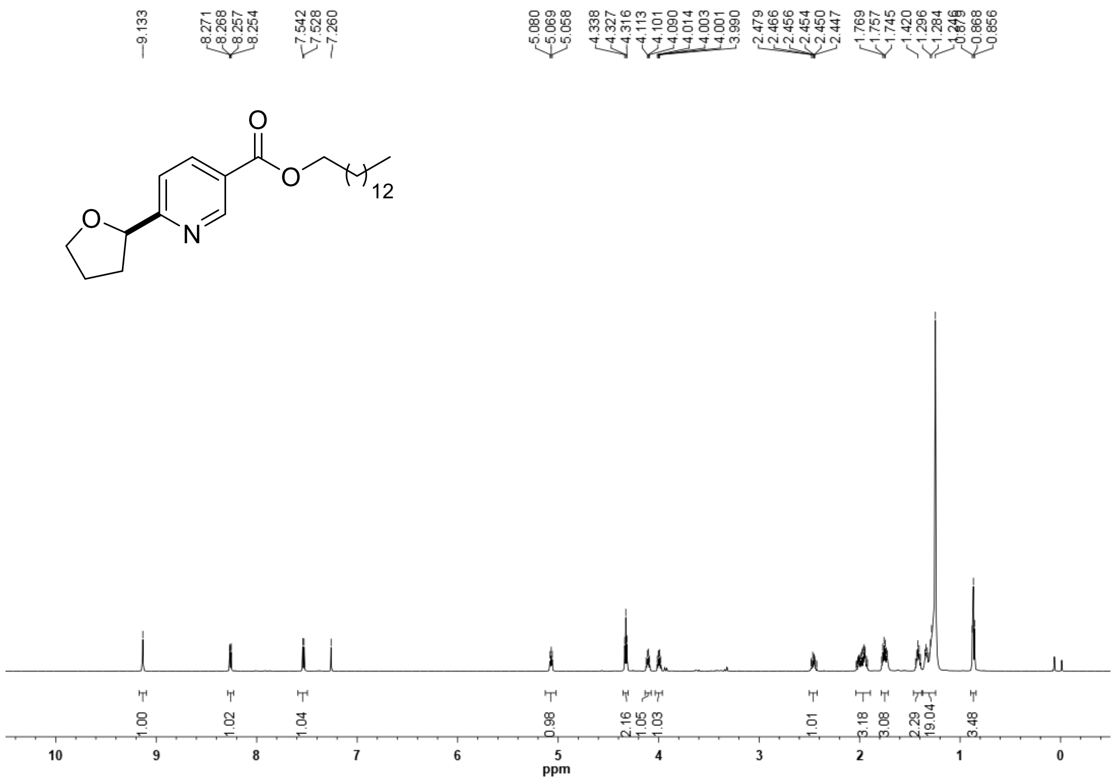

Figure S99. ^{1}H NMR spectrum of **3al** (400 MHz) in $CDCl_3$.

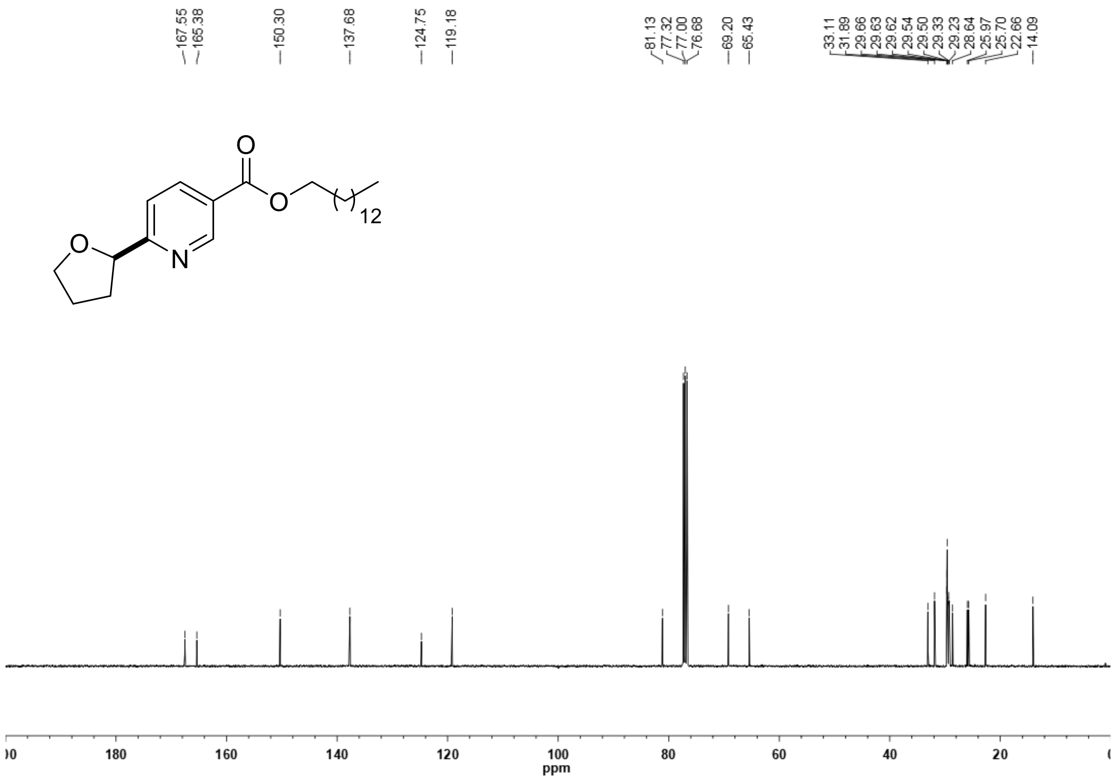

Figure S100. ^{13}C NMR spectrum of **3al** (100 MHz) in $CDCl_3$.

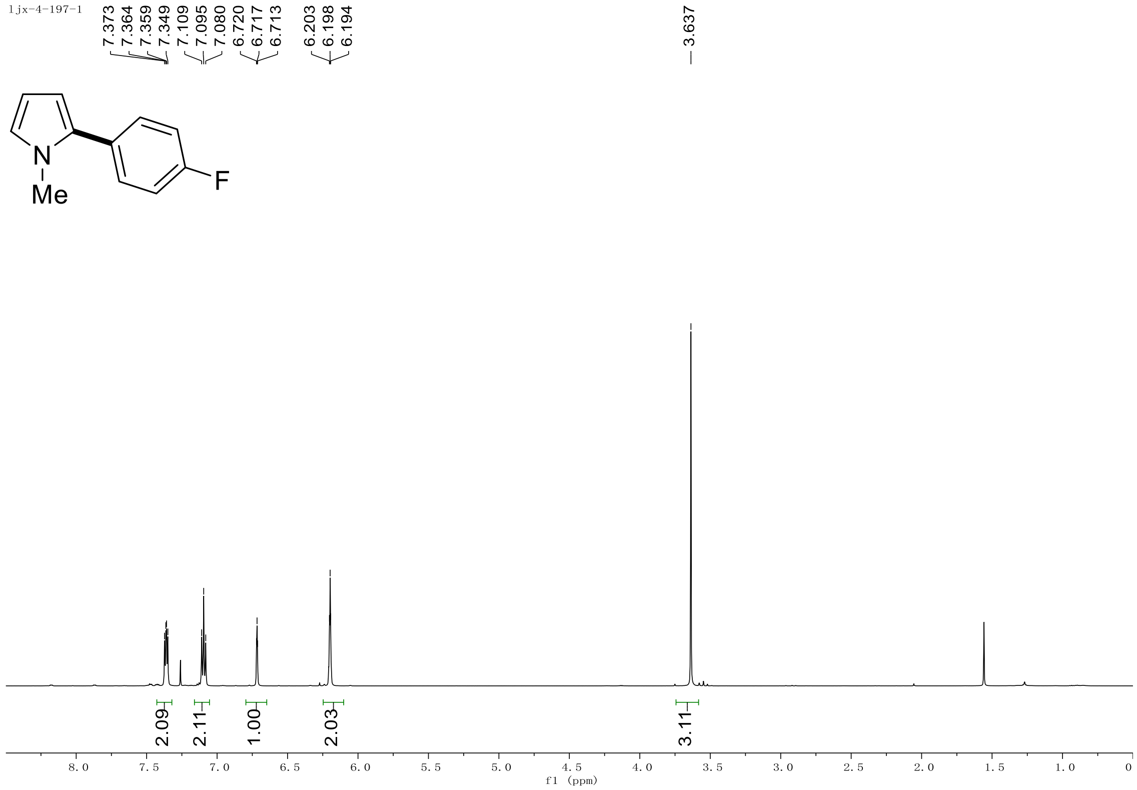

Figure S101. ^{1}H NMR spectrum of **6a** (600 MHz) in $CDCl_3$.

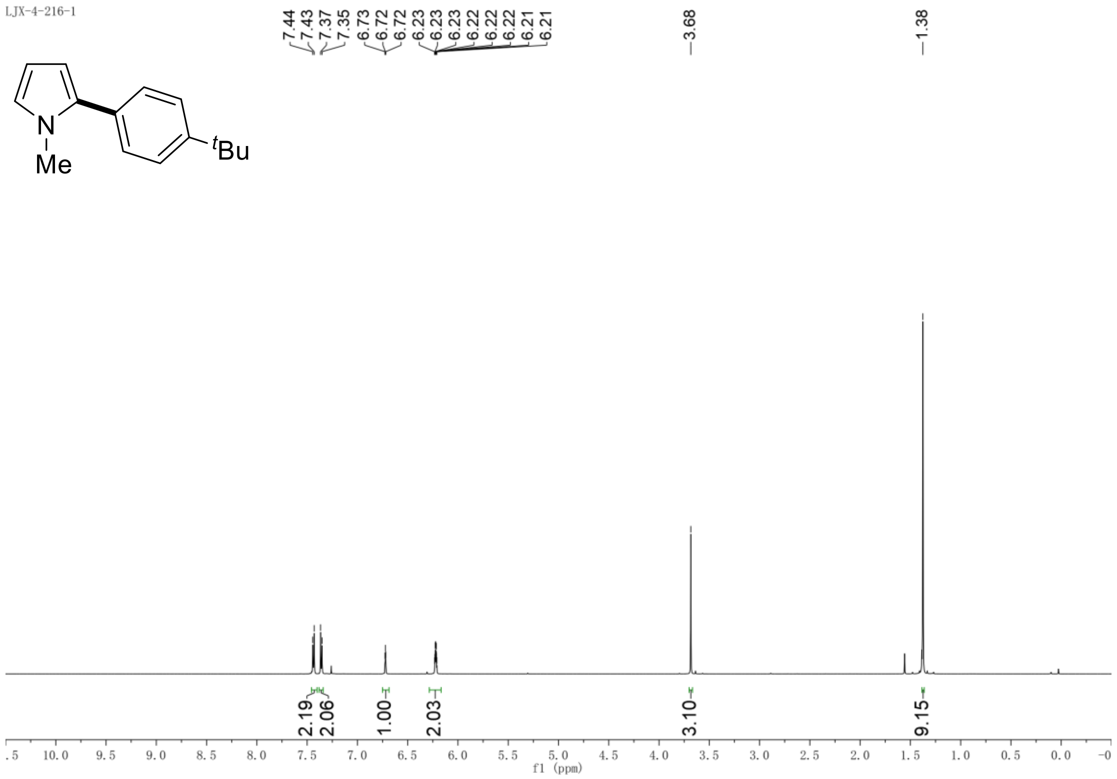

Figure S102. ^{1}H NMR spectrum of **6b** (600 MHz) in $CDCl_3$.

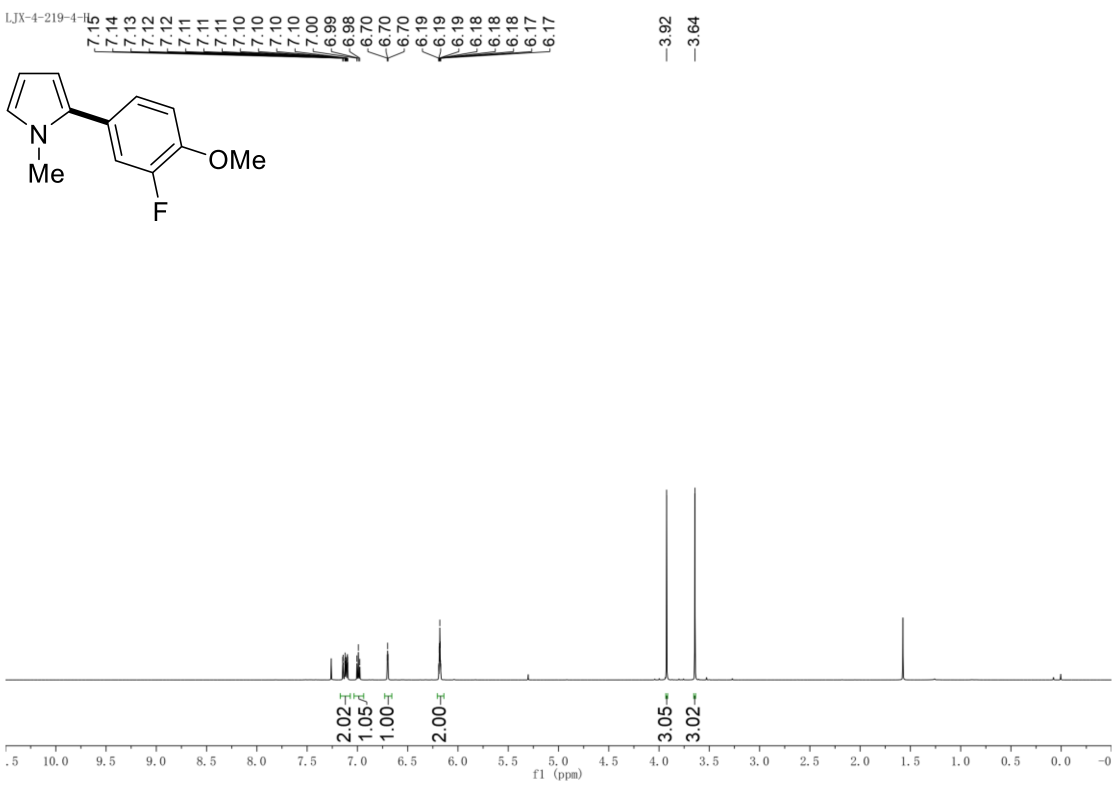

Figure S103. ^{1}H NMR spectrum of **6c** (600 MHz) in $CDCl_3$.

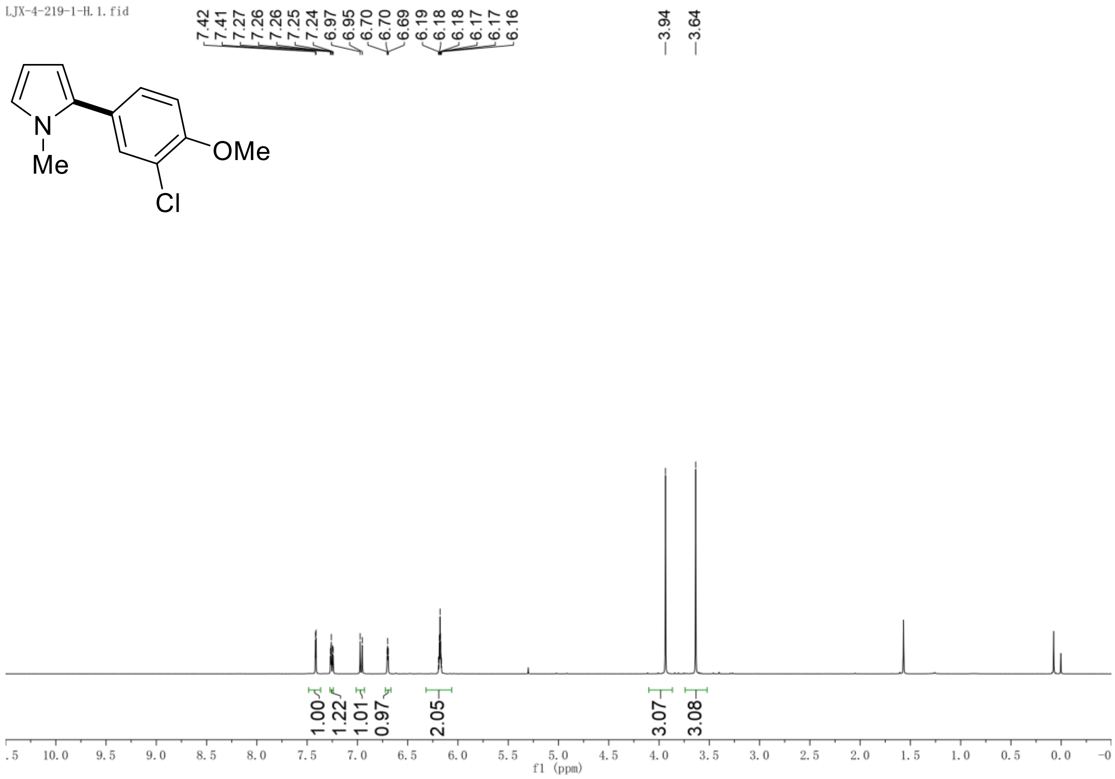

Figure S104. ^{1}H NMR spectrum of **6d** (600 MHz) in $CDCl_3$.

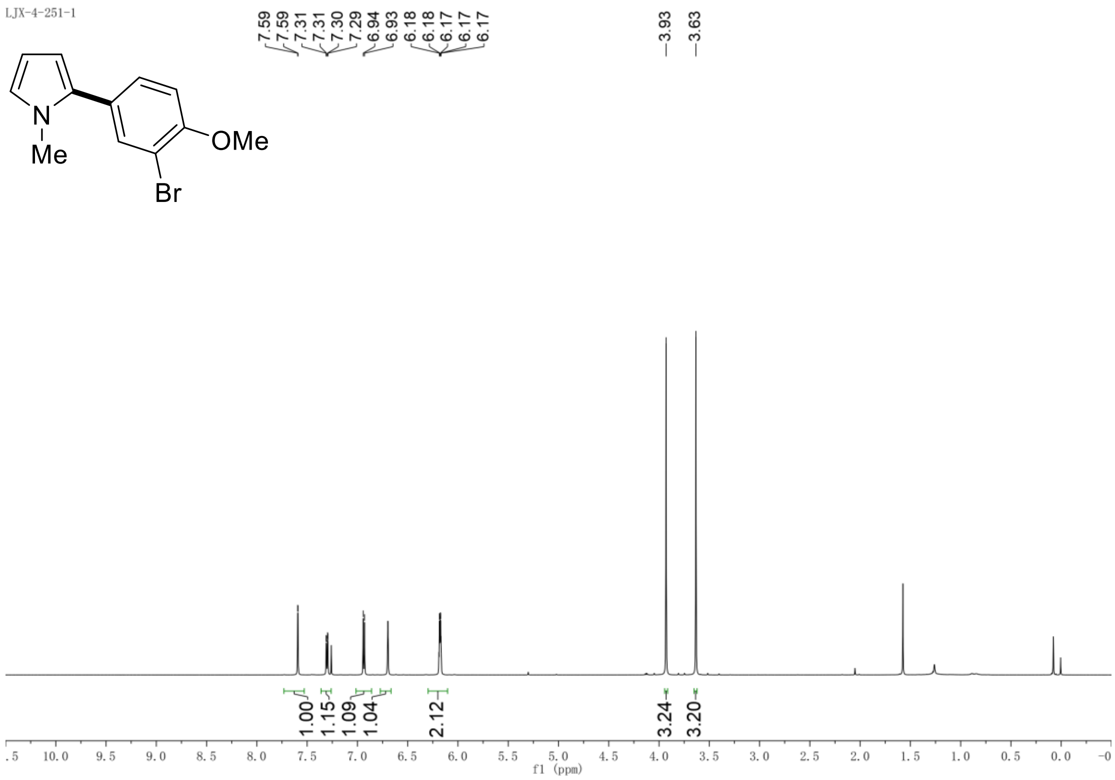

Figure S105. ^{1}H NMR spectrum of **6e** (600 MHz) in $CDCl_3$.

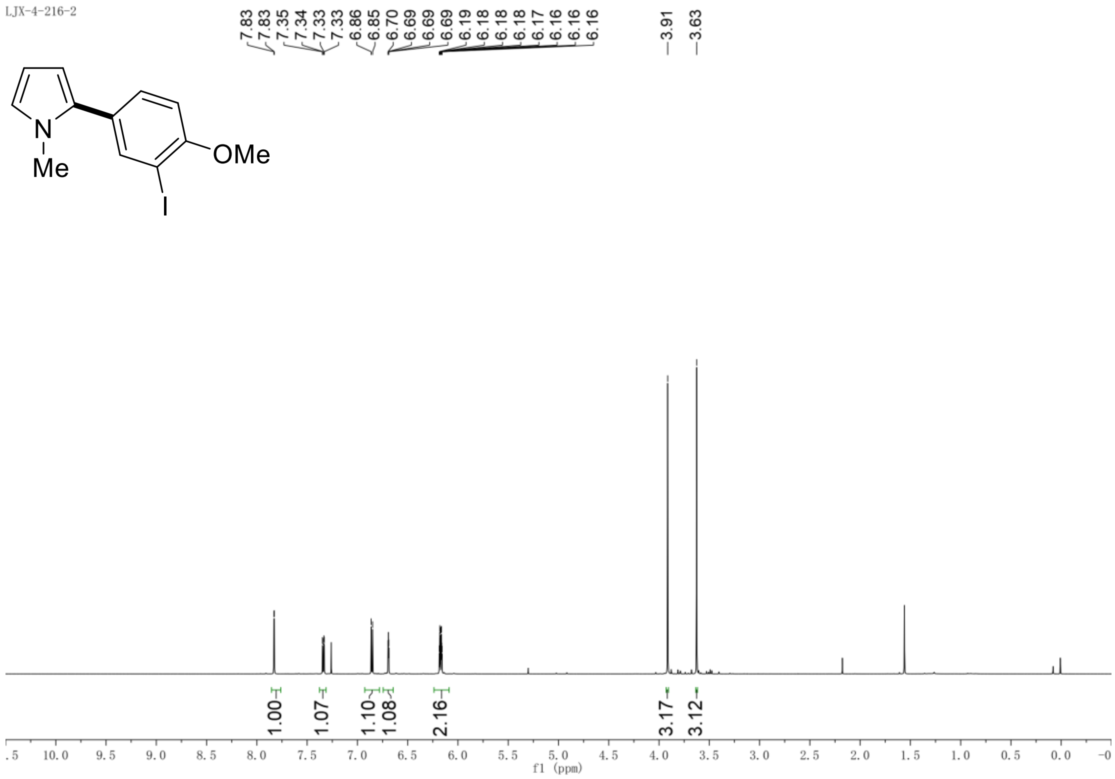

Figure S106. ^{1}H NMR spectrum of **6f** (600 MHz) in $CDCl_3$.

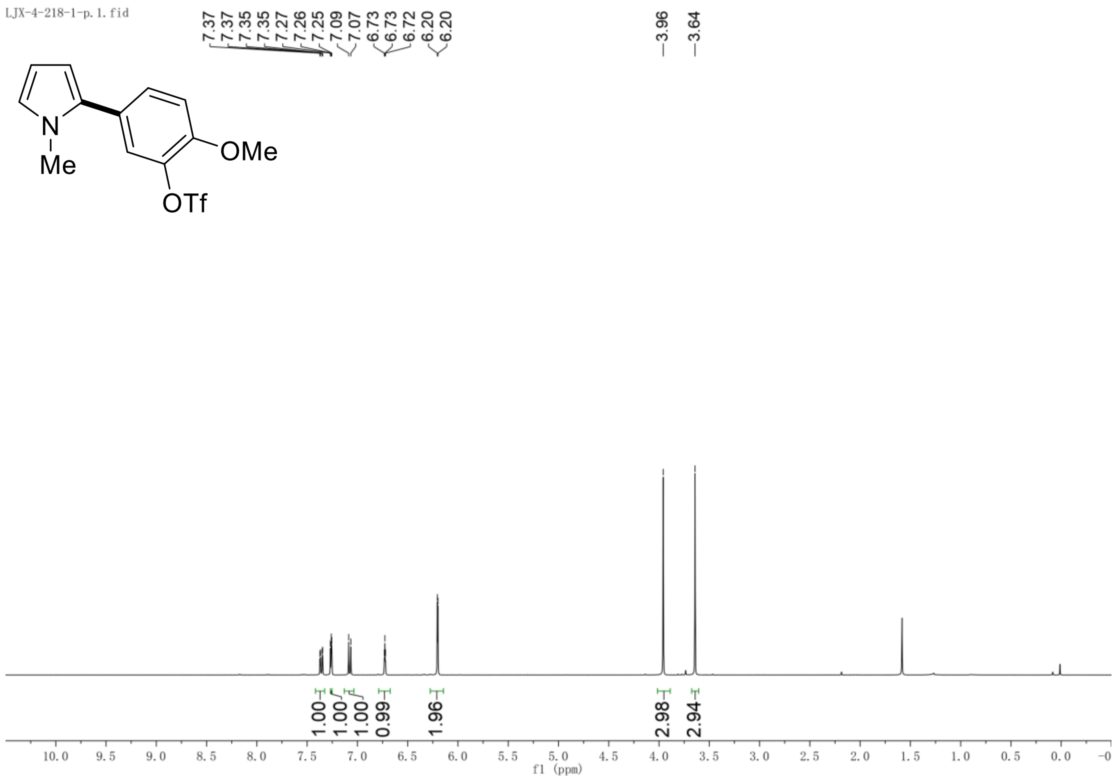

Figure S107. ^{1}H NMR spectrum of **6g** (400 MHz) in $CDCl_3$.

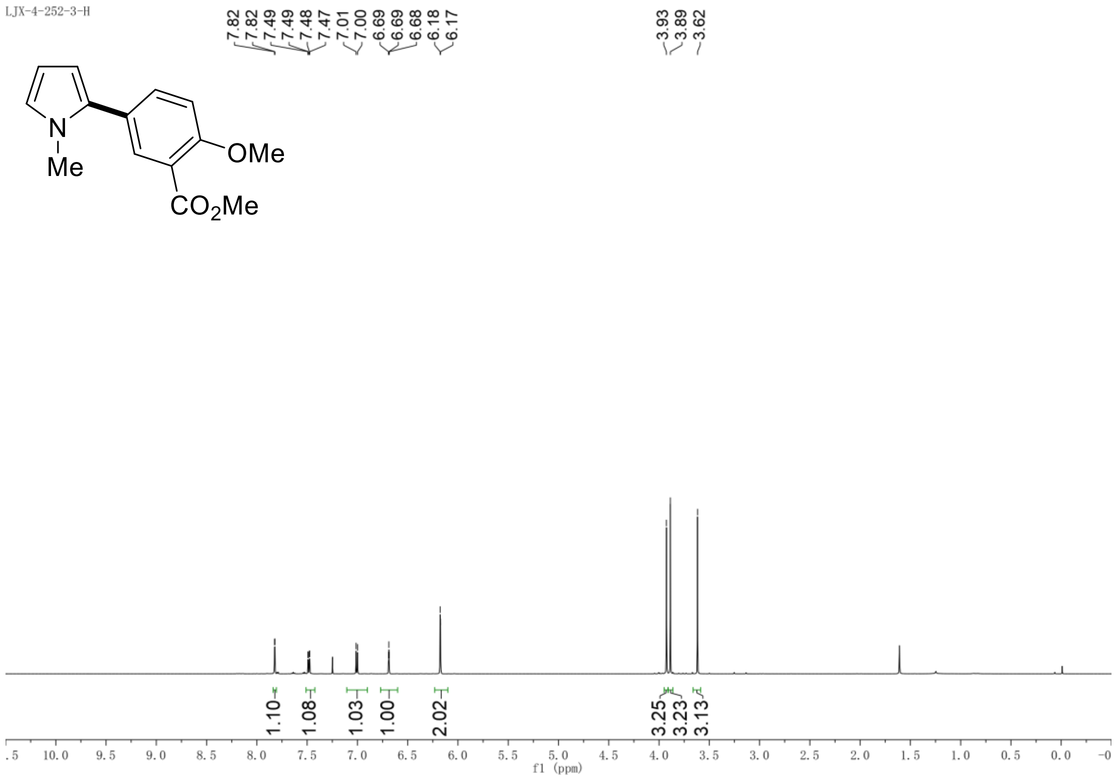

Figure S108. ^{1}H NMR spectrum of **6h** (600 MHz) in $CDCl_3$.

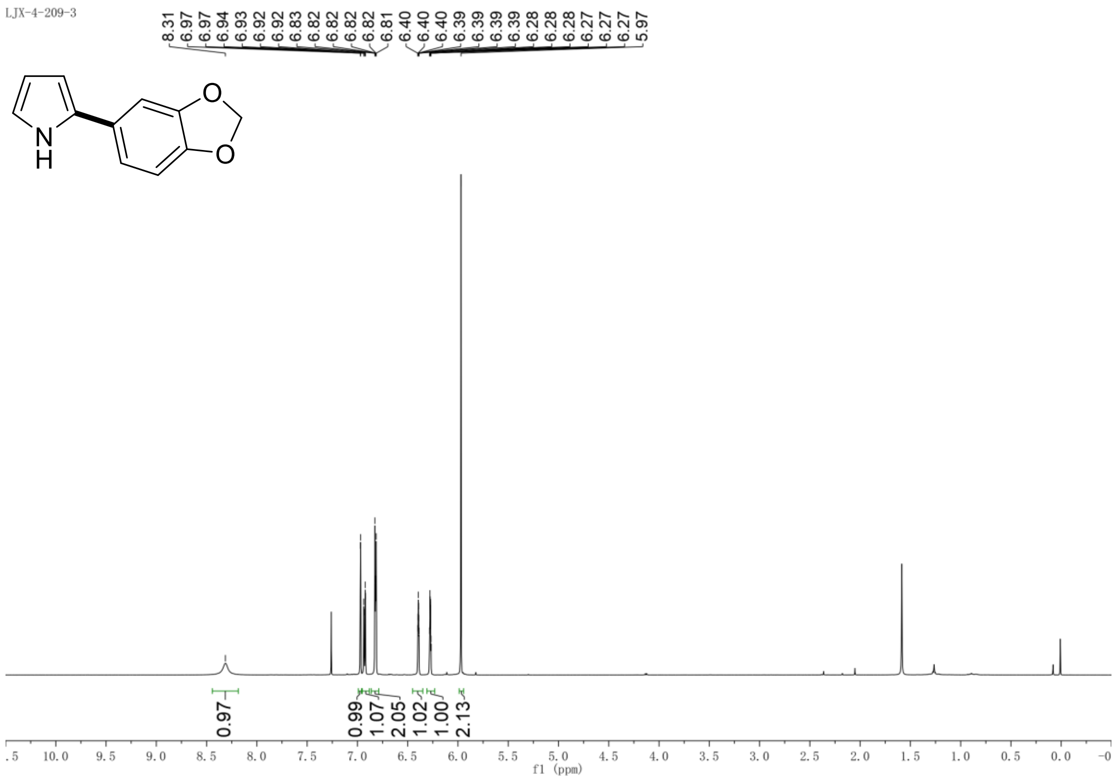

Figure S109. ^{1}H NMR spectrum of **6i** (600 MHz) in $CDCl_3$.

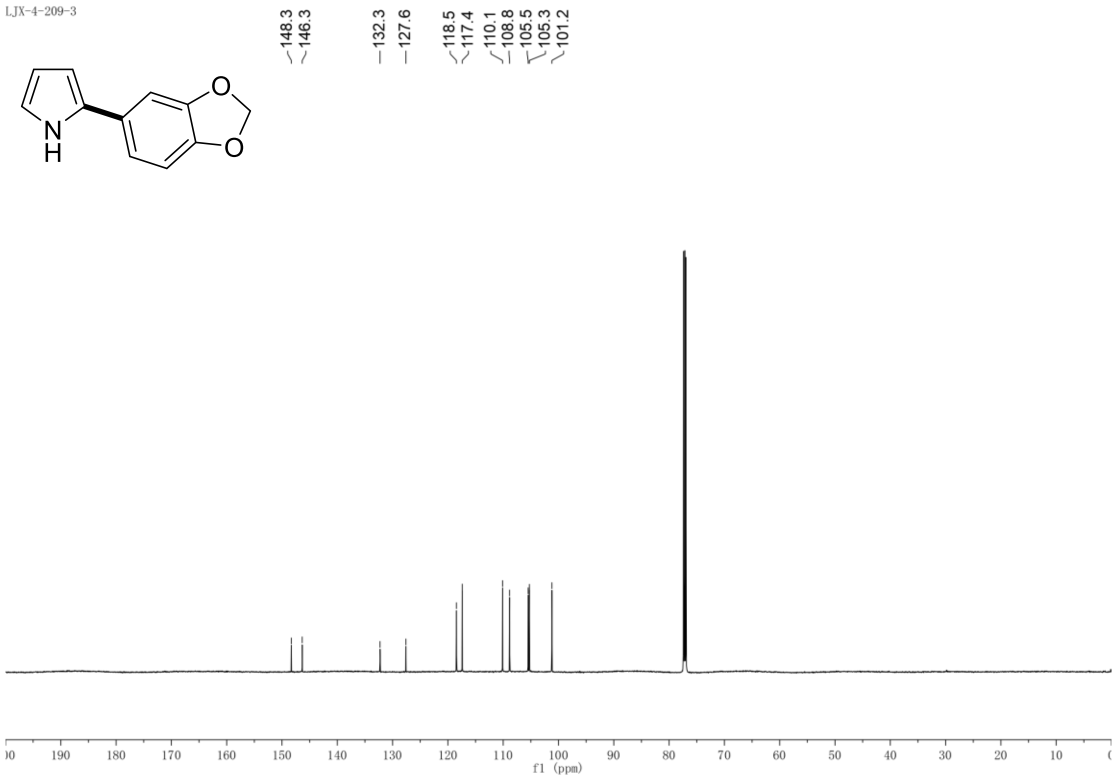

Figure S110. ^{13}C NMR spectrum of **6i** (105 MHz) in $CDCl_3$.

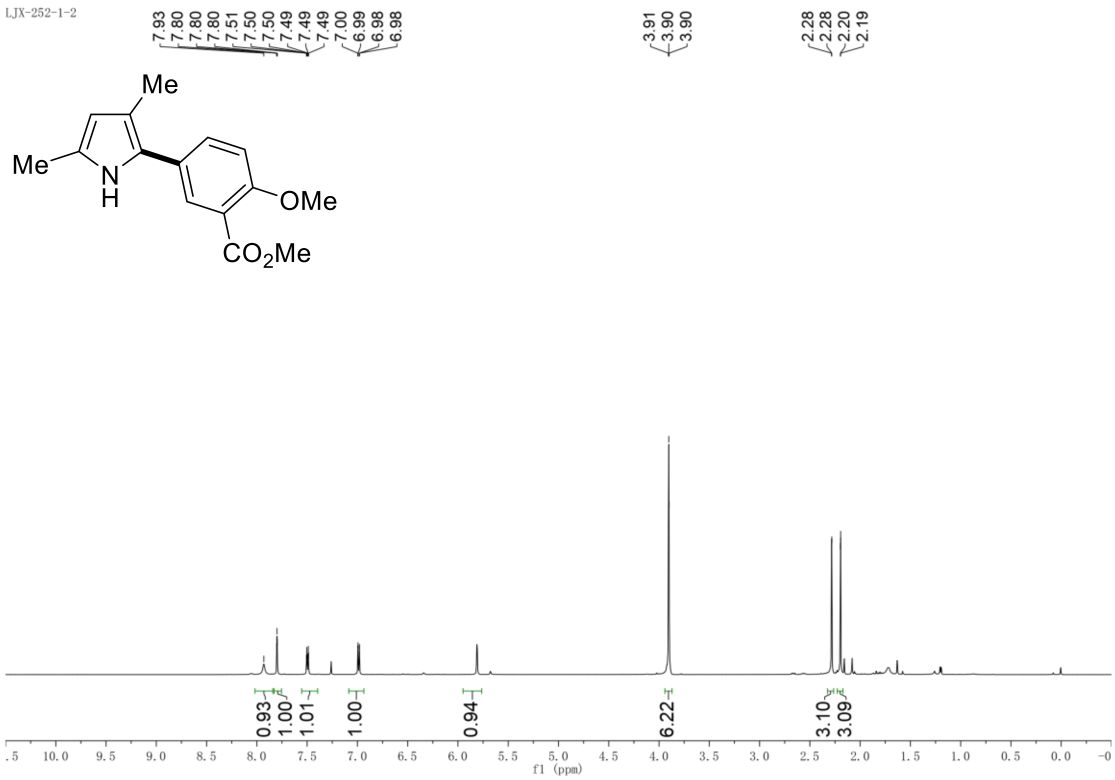

Figure S111. ^{1}H NMR spectrum of **6j** (600 MHz) in $CDCl_3$.

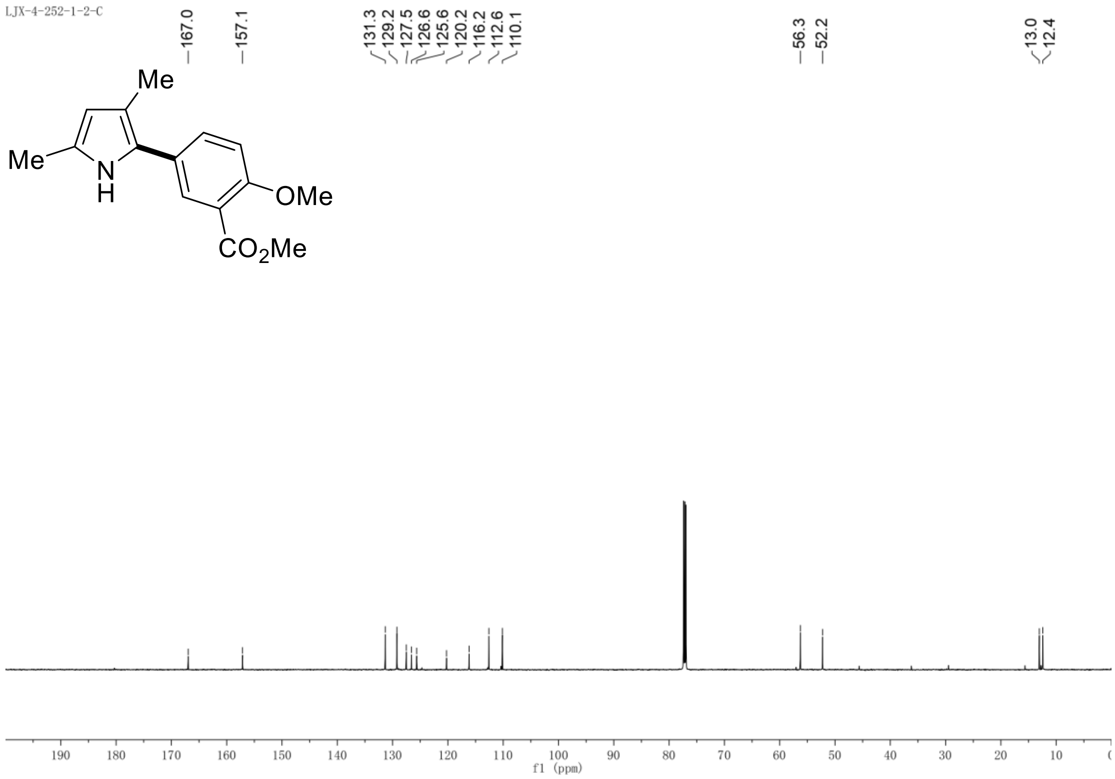

Figure S112. ^{13}C NMR spectrum of **6j** (105 MHz) in $CDCl_3$.

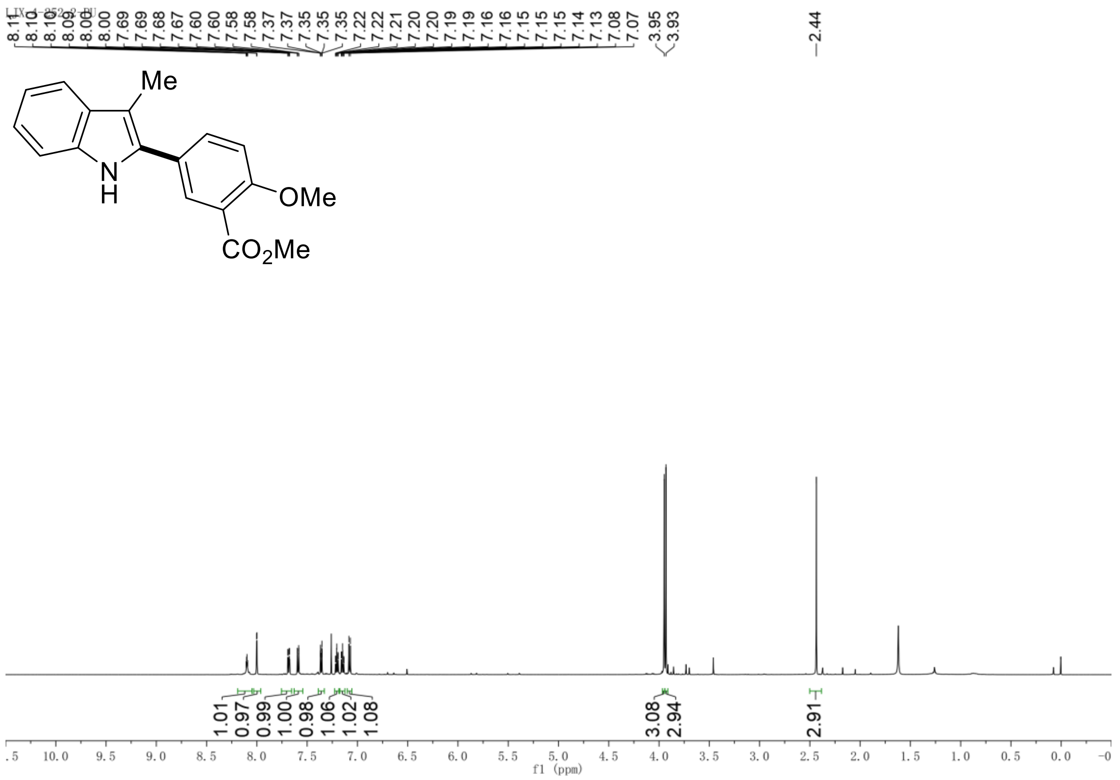

Figure S113. ^{1}H NMR spectrum of **6k** (600 MHz) in $CDCl_3$.

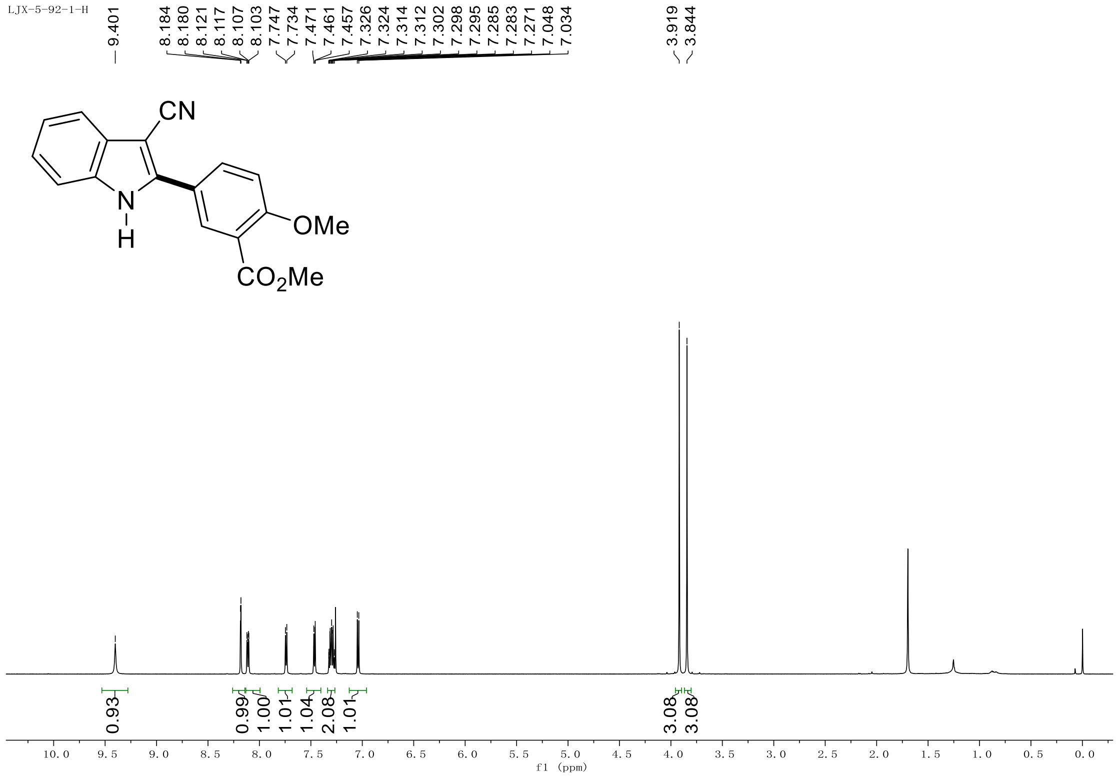

Figure S114. ^{1}H NMR spectrum of **6l** (600 MHz) in $CDCl_3$.

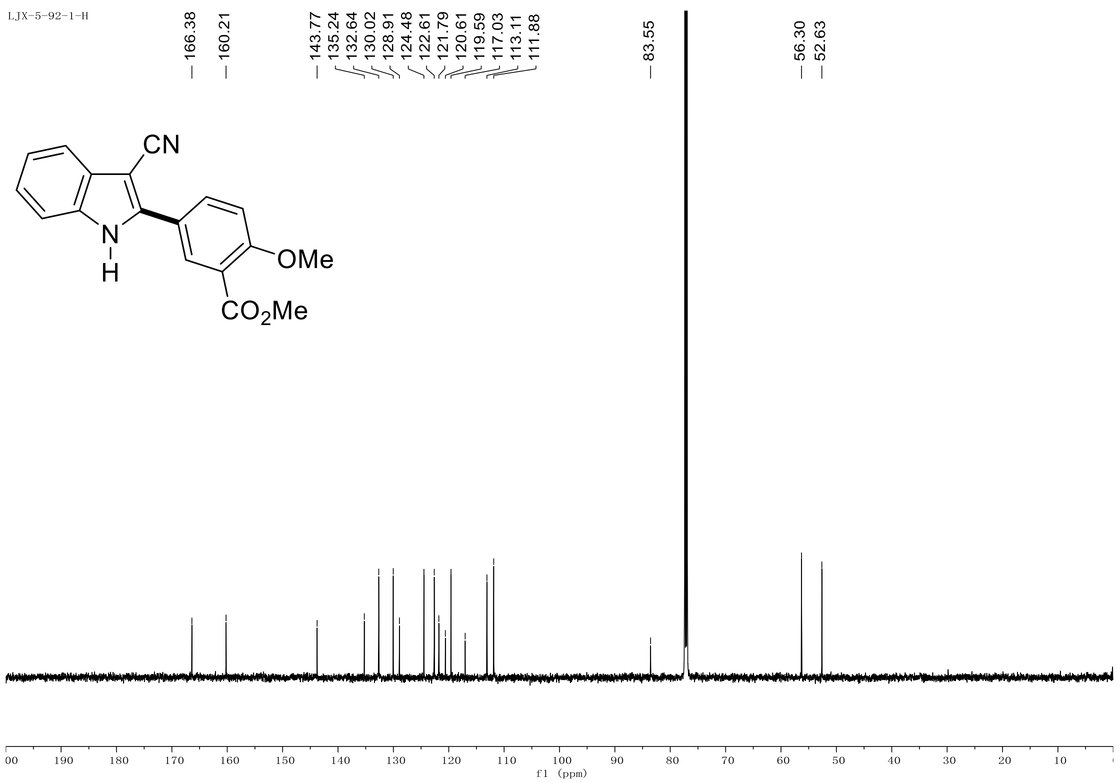

Figure S115. ^{13}C NMR spectrum of **6l** (105 MHz) in $CDCl_3$.

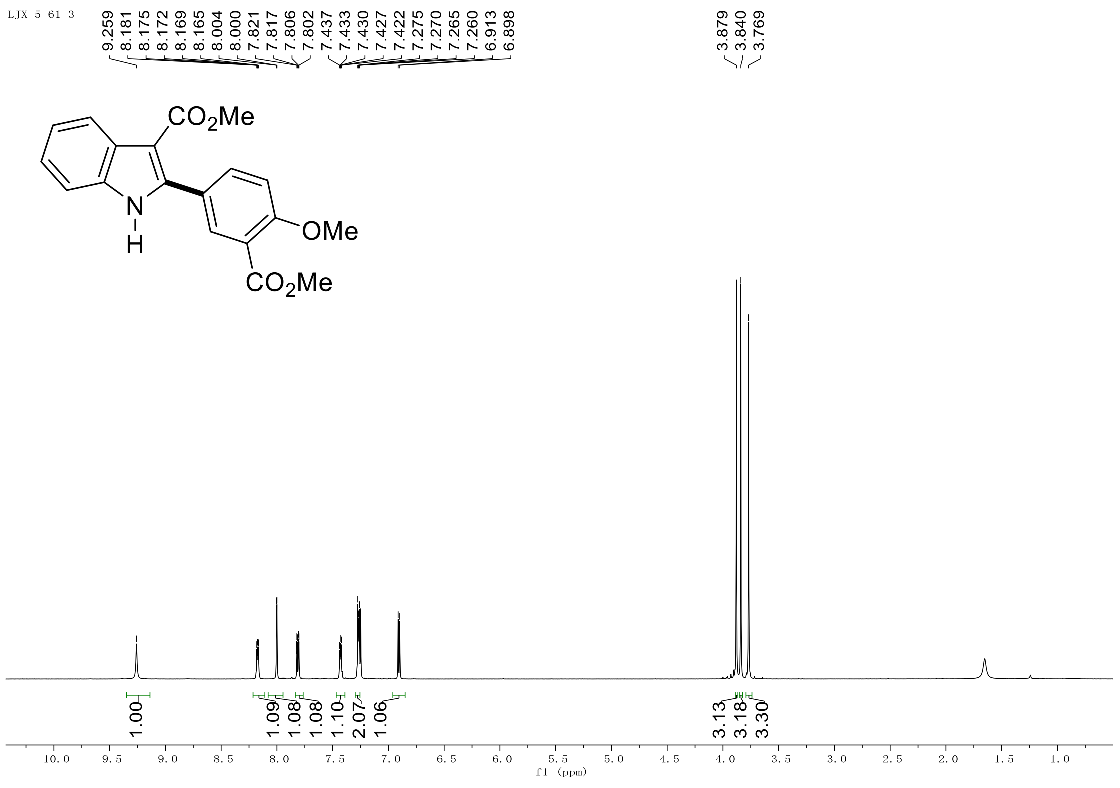

Figure S116. ^{1}H NMR spectrum of **6m** (600 MHz) in $CDCl_3$.

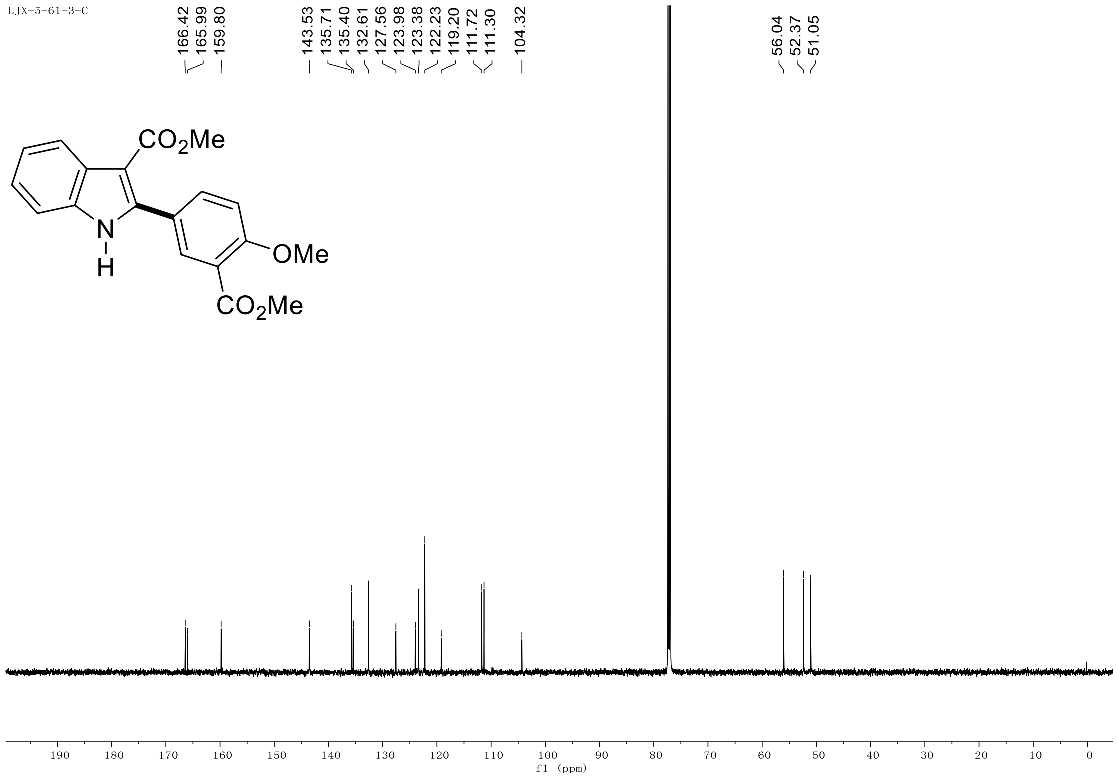

Figure S117. ^{13}C NMR spectrum of **6m** (105 MHz) in $CDCl_3$

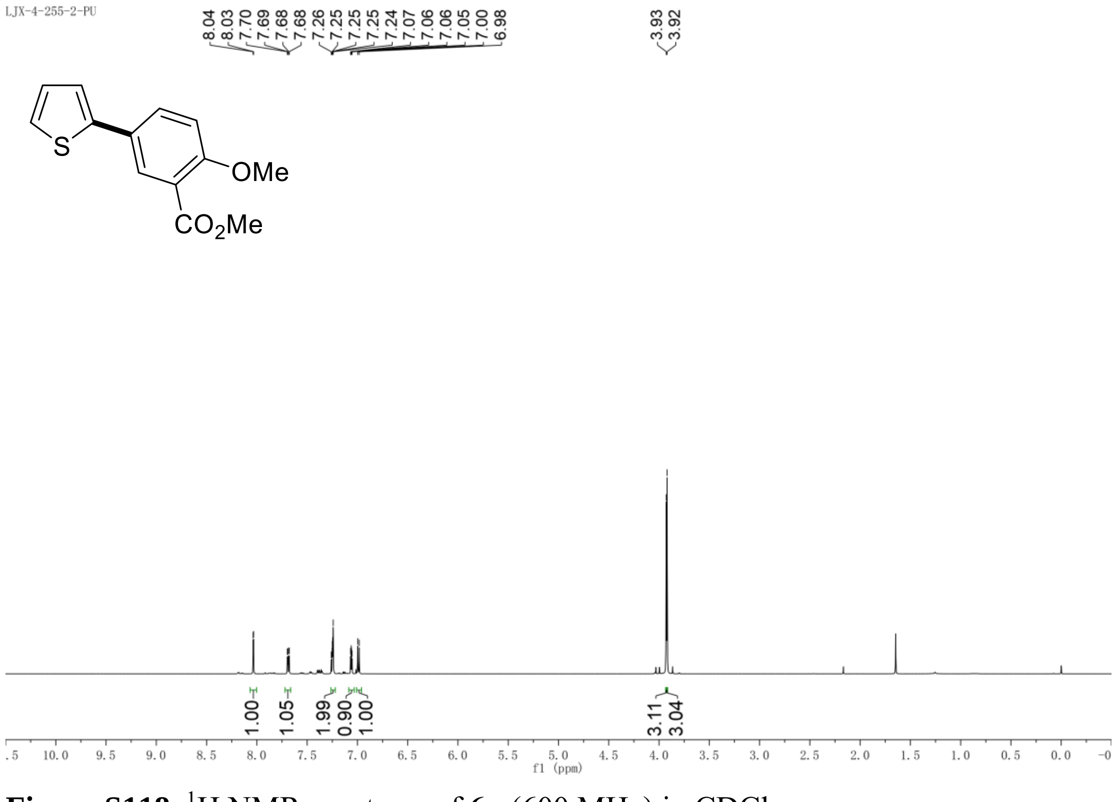

Figure S118. ^{1}H NMR spectrum of **6n** (600 MHz) in $CDCl_3$.

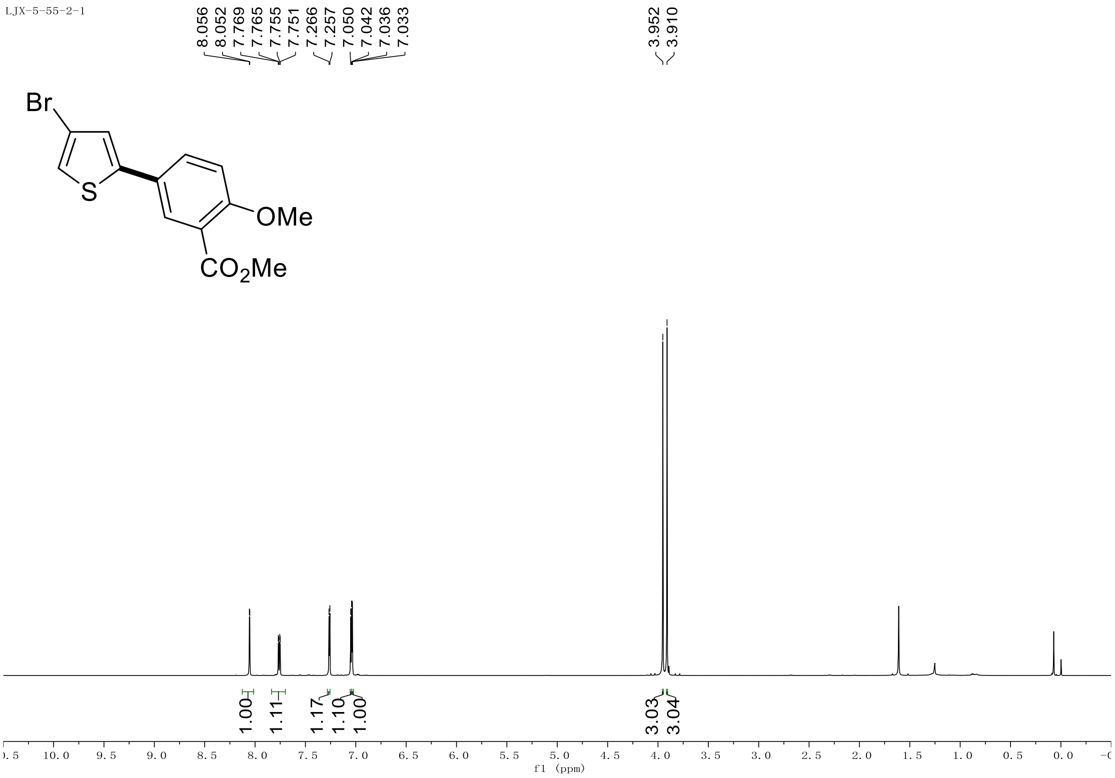

Figure S119. ^{1}H NMR spectrum of **6o** (600 MHz) in $CDCl_3$.

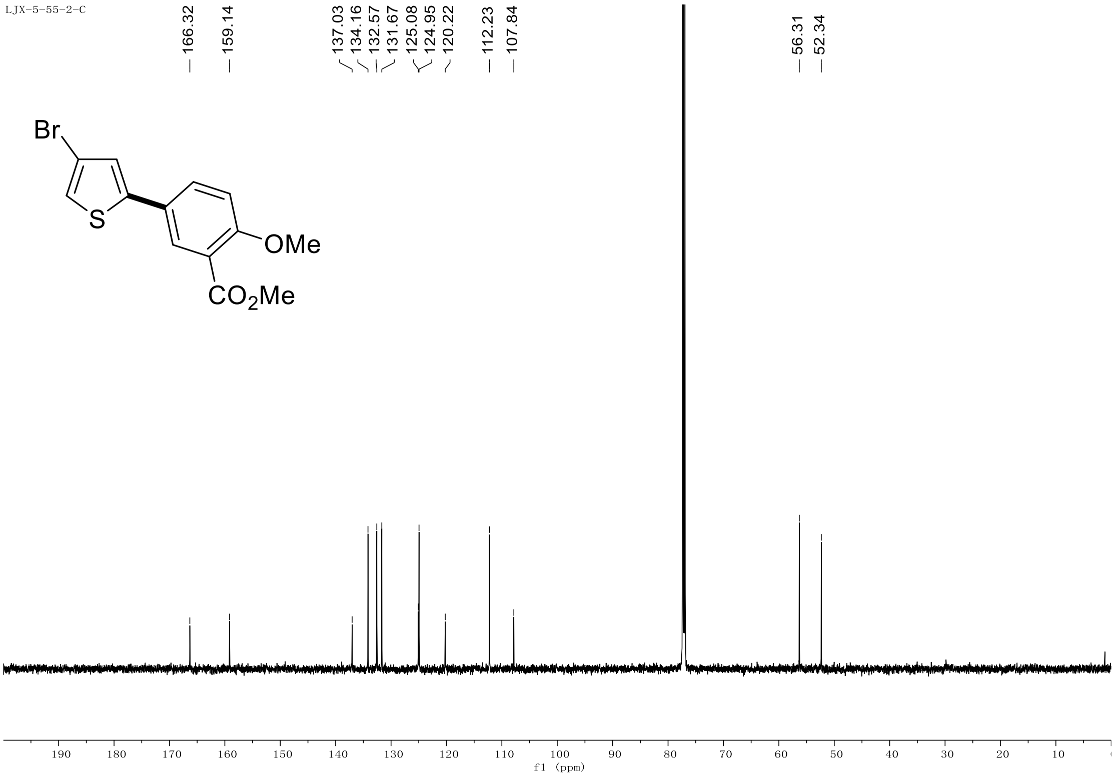

Figure S120. ^{13}C NMR spectrum of **6o** (105 MHz) in $CDCl_3$.

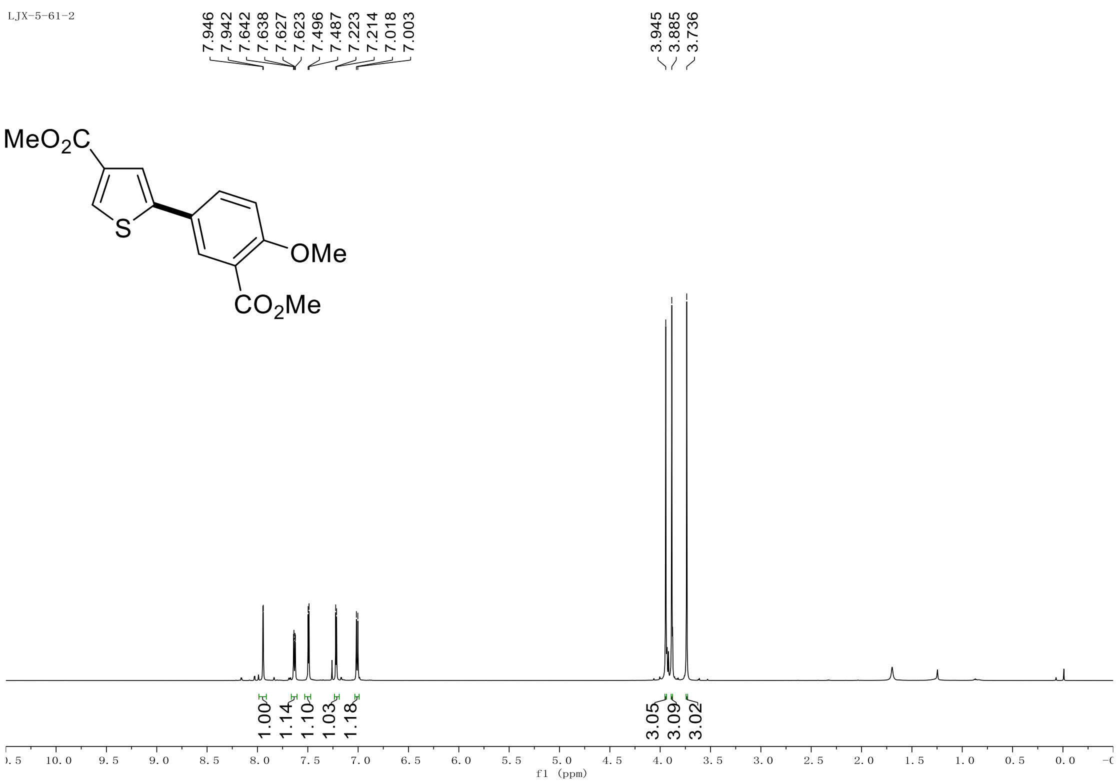

Figure S121. ^{1}H NMR spectrum of **6p** (600 MHz) in $CDCl_3$.

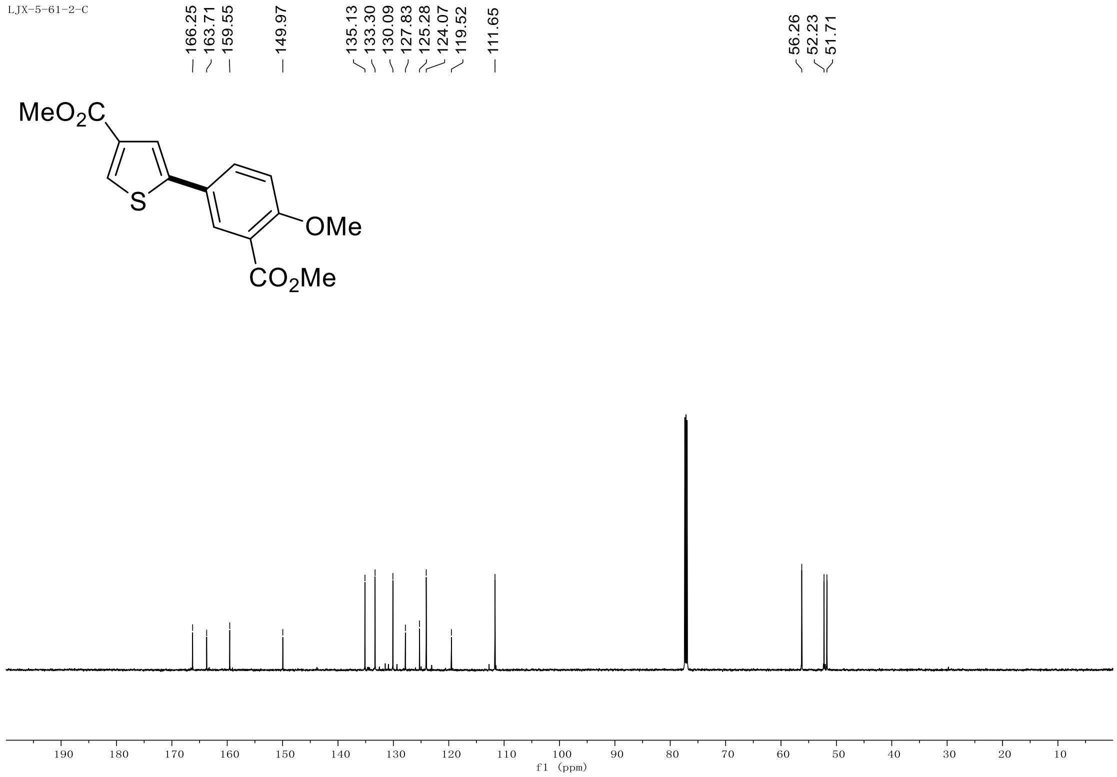

Figure S122. ^{13}C NMR spectrum of **6p** (105 MHz) in $CDCl_3$.

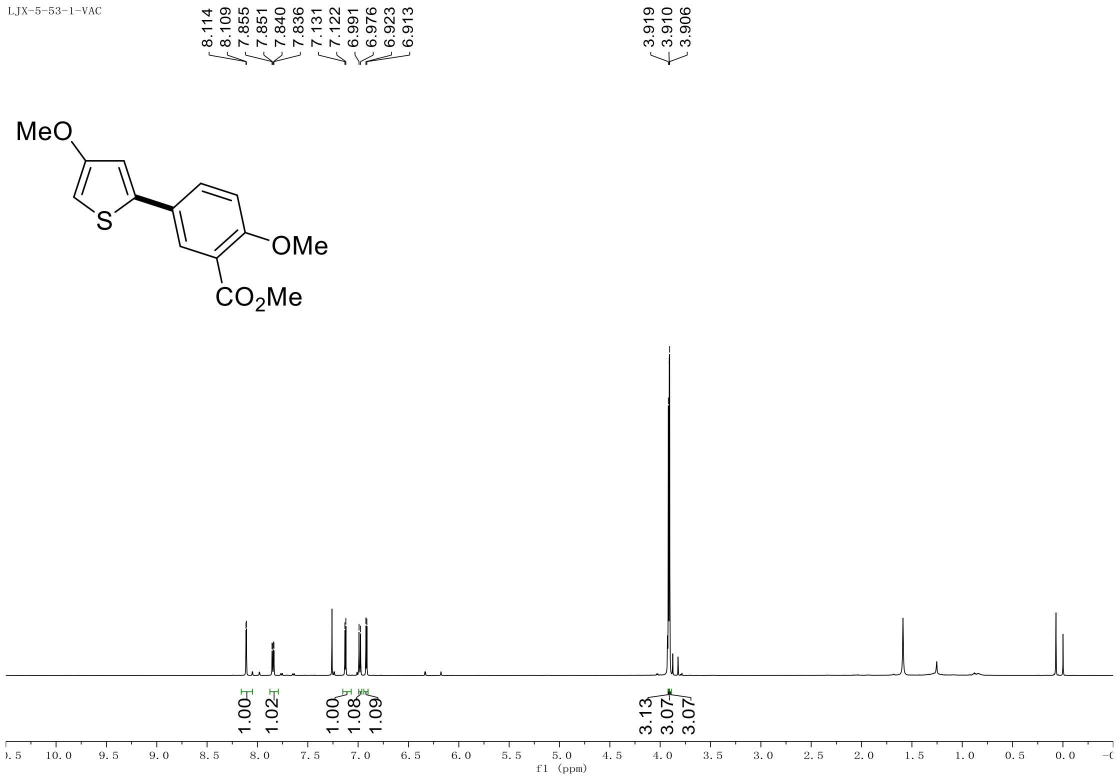

Figure S123. ^{1}H NMR spectrum of **6q** (600 MHz) in $CDCl_3$.

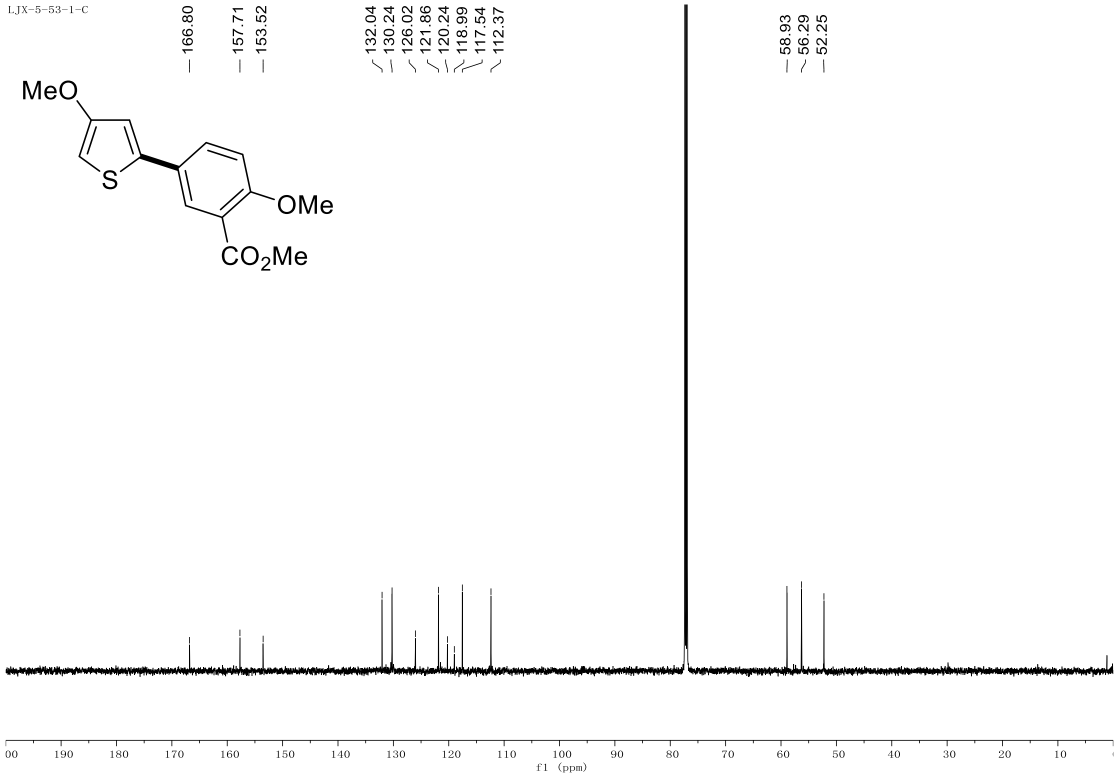

Figure S124. ^{13}C NMR spectrum of **6q** (105 MHz) in $CDCl_3$.

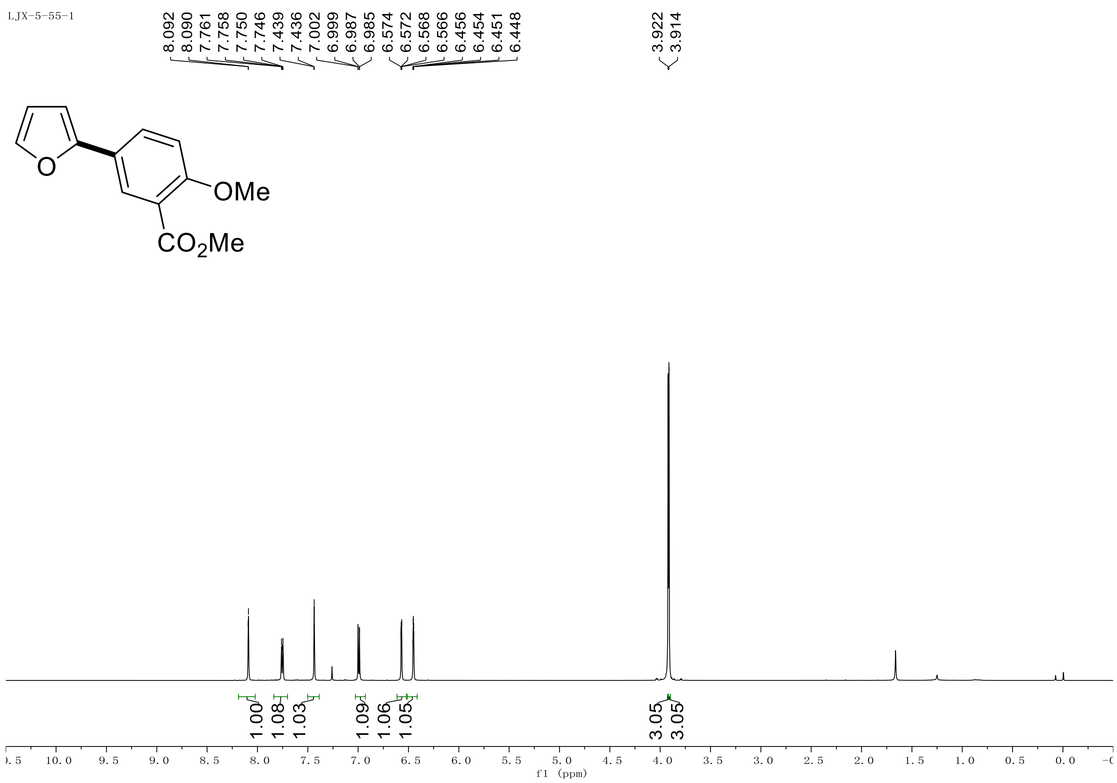

Figure S125. ^{1}H NMR spectrum of **6r** (600 MHz) in $CDCl_3$.

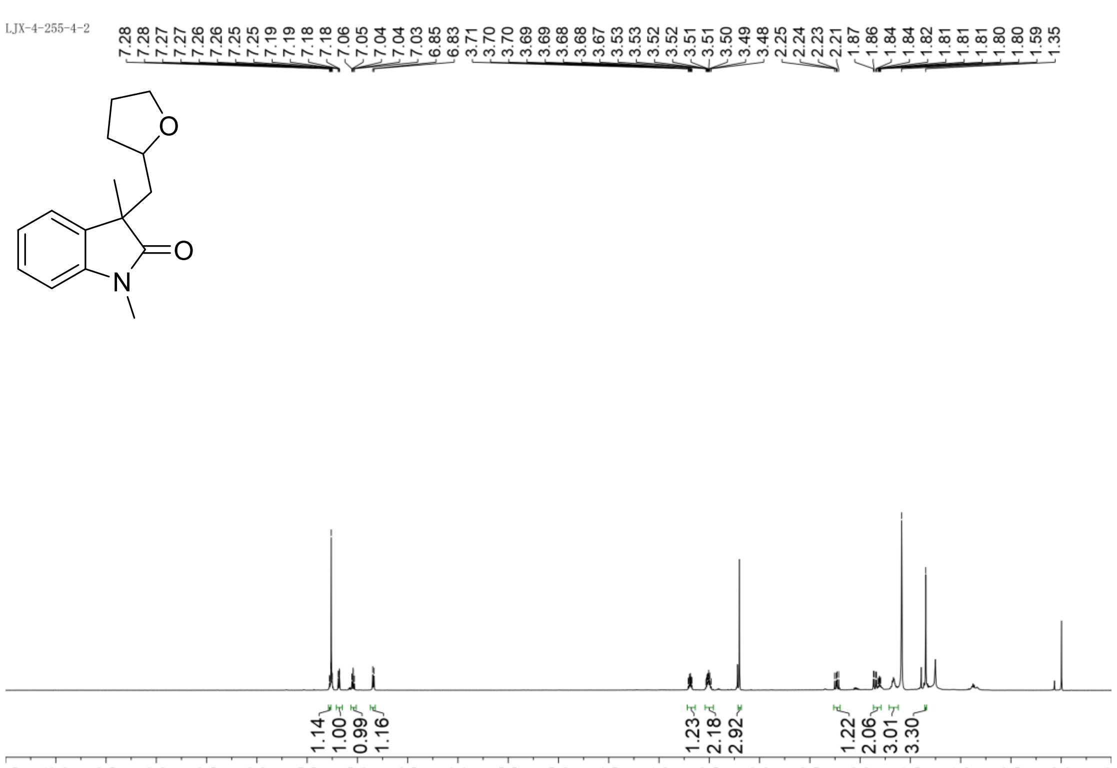

Figure S126. ^{1}H NMR spectrum of **10** (600 MHz) in $CDCl_3$.

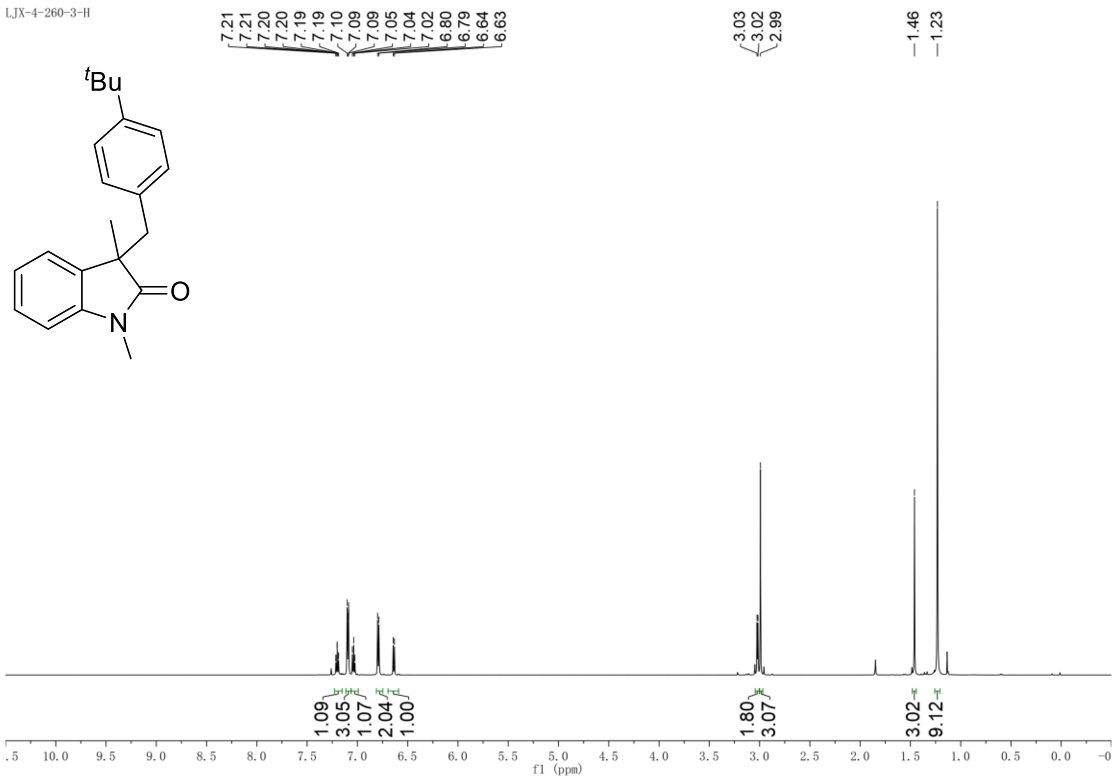

Figure S127. ^{1}H NMR spectrum of **11** (600 MHz) in $CDCl_3$.

6. Reference

1. Ma, X.; Dang, H.; Rose, J. A.; Rablen, P.; Herzon, S. B., Hydroheteroarylation of Unactivated Alkenes Using N-Methoxyheteroarenium Salts. *J. Am. Chem. Soc.* **2017,** *139* (16), 5998-6007.

2. Dai, G.; He, Y.; Niu, Z.; He, P.; Zhang, C.; Zhao, Y.; Zhang, X.; Zhou, H., A Dual-Ion Organic Symmetric Battery Constructed from Phenazine-Based Artificial Bipolar Molecules. *Angew. Chem. Int. Ed.* **2019,** *58* (29), 9902-9906.

3. Ballistreri, F. P.; Pappalardo, A.; Tomaselli, G. A.; Toscano, R. M.; Sfrazzetto, G. T., Heteroditopic Chiral Uranyl–Salen Receptor for Molecular Recognition of Amino Acid Ammonium Salts. *Eur. J. Org. Chem.* **2010,** *2010* (20), 3806-3810.

4. Shen, L.; Gao, X.; Luan, N.; Liu, Z.; Li, J.; Zou, D.; Wu, Y.; Wu, Y. J. O.; Chemistry, B., External oxidant-free alkylation of quinoline and pyridine derivatives. *Org. Biomol. Chem.* **2020,** *18* (9), 1738-1742.

5. Li, X.; Liu, C.; Guo, S.; Wang, W.; Zhang, Y., PIFA-Mediated Cross-Dehydrogenative Coupling of N-Heteroarenes with Cyclic Ethers: Ethanol as an Efficient Promoter. *Eur. J. Org. Chem.* **2021,** *2021* (3), 411-421.

6. Sun, W.; Xie, Z.; Liu, J.; Wang, L. J. O.; Chemistry, B., Oxidative cross-coupling of pyridine N-oxides and ethers between C (sp 2)–H/C (sp 3)–H bonds under transition-metal-free conditions. *Org. Biomol. Chem.* **2015,** *13* (15), 4596-4604.

7. Bhakat, M.; Biswas, P.; Dey, J.; Guin, J., Heteroarylation of Ethers, Amides, and Alcohols with Light and O2. *Org. Lett.* **2021,** *23* (17), 6886-6890.

8. Lipp, B.; Nauth, A. M.; Opatz, T., Transition-Metal-Free Decarboxylative Photoredox Coupling of Carboxylic Acids and Alcohols with Aromatic Nitriles. *J.Org.Chem.* **2016,** *81* (15), 6875-6882.

9. Wu, Y.-H.; Wang, N.-X.; Zhang, T.; Zhang, L.-Y.; Gao, X.-W.; Xu, B.-C.; Xing, Y.; Chi, J.-Y., Rare-Earth Y(OTf)3 Catalyzed Coupling Reaction of Ethers with Azaarenes. *Org. Lett.* **2019,** *21* (18), 7450-7454.

10. Li, L.; Song, X.; Qi, M.-F.; Sun, B., Weak Brønsted base-promoted photoredox catalysis for CH alkylation of heteroarenes mediated by triplet excited diaryl ketone. *Tetrahedron Lett.* **2022,** *99*, 153846.

11. Zhou, Z.; Wu, Y.; Yang, P.; Deng, S.; Zhang, Q.; Li, D., Silver-Catalyzed Cross Dehydrogenative Coupling between Heteroarenes and Cyclic Ethers under Mild Conditions. *ChemistrySelect* **2021,** *6* (11), 2770-2773.

12. Zhou, J.; Zou, Y.; Zhou, P.; Chen, Z.; Li, J., Copper-catalyzed versatile C(sp3)–H arylation: synthetic scope and regioselectivity investigations. *Org. Chem. Front.* **2019,** *6* (10), 1594-1598.

13. Huang, C.-Y.; Li, J.; Liu, W.; Li, C.-J., Diacetyl as a “traceless” visible light photosensitizer in metal-free cross-dehydrogenative coupling reactions. *Chem. Sci.* **2019,** *10* (19), 5018-5024.

14. Rammal, F.; Gao, D.; Boujnah, S.; Hussein, A. A.; Lalevée, J.; Gaumont, A.-C.; Morlet-Savary, F.; Lakhdar, S., Photochemical C–H Silylation and Hydroxymethylation of Pyridines and Related Structures: Synthetic Scope and Mechanisms. *ACS Catal.* **2020,** *10* (22), 13710-13717.

15. Lebedev, Y.; Polishchuk, I.; Maity, B.; Dinis Veloso Guerreiro, M.; Cavallo, L.; Rueping, M. J. J. o. t. A. C. S., Asymmetric hydroboration of heteroaryl ketones by aluminum catalysis. *J. Am. Chem. Soc.* **2019,** *141* (49), 19415-19423.

16. Huff, C. A.; Cohen, R. D.; Dykstra, K. D.; Streckfuss, E.; DiRocco, D. A.; Krska, S. W., Photoredox-Catalyzed Hydroxymethylation of Heteroaromatic Bases. *J.Org.Chem.* **2016,** *81* (16), 6980-6987.

17. Rammal, F.; Gao, D.; Boujnah, S.; Gaumont, A. C.; Hussein, A. A.; Lakhdar, S., Visible-Light-Mediated C–H Alkylation of Pyridine Derivatives. *Org. Lett.* **2020,** *22* (19), 7671-7675.
18. Deng, G.; Li, C.-J., Sc(OTf)3-Catalyzed Direct Alkylation of Quinolines and Pyridines with Alkanes. *Org. Lett.* **2009,** *11* (5), 1171-1174.
19. Lai, X.-L.; Shu, X.-M.; Song, J.; Xu, H.-C., Electrophotocatalytic Decarboxylative C−H Functionalization of Heteroarenes. *Angew. Chem. Int. Ed.* **2020,** *59* (26), 10626-10632.
20. Chen, P.; Nan, J.; Hu, Y.; Ma, Q.; Ma, Y., RuII-Catalyzed/NH2-Assisted Selective Alkenyl C–H [5 + 1] Annulation of Alkenylanilines with Sulfoxonium Ylides to Quinolines. *Org. Lett.* **2019,** *21* (12), 4812-4815.
21. Pagire, S. K.; Hossain, A.; Reiser, O., Temperature Controlled Selective C–S or C–C Bond Formation: Photocatalytic Sulfonylation versus Arylation of Unactivated Heterocycles Utilizing Aryl Sulfonyl Chlorides. *Org. Lett.* **2018,** *20* (3), 648-651.
22. Shen, N.; Li, R.; Liu, C.; Shen, X.; Guan, W.; Shang, R., Photocatalytic Cross-Couplings of Aryl Halides Enabled by o-Phosphinophenolate and o-Phosphinothiophenolate. *ACS Catal.* **2022,** *12* (5), 2788-2795.
23. Aukland, M. H.; Šiaučiulis, M.; West, A.; Perry, G. J. P.; Procter, D. J., Metal-free photoredox-catalysed formal C–H/C–H coupling of arenes enabled by interrupted Pummerer activation. *Nat. Catal.* **2020,** *3* (2), 163-169.
24. Xia, D.; Li, Y.; Miao, T.; Li, P.; Wang, L., Visible-light-induced dual C–C bond formation via selective C(sp3)–H bond cleavage: efficient access to alkylated oxindoles from activated alkenes and simple ethers under metal-free conditions. *Green Chem.* **2017,** *19* (7), 1732-1739.
25. Wang, X.; Xun, X.; Song, H.; Liu, Y.; Wang, Q., Palladium Metallaphotoredox-Catalyzed 2-Arylation of Indole Derivatives. *Org. Lett.* **2022,** *24* (25), 4580-4585.
26. Wang, Z.; Chen, J.; Lin, Z.; Quan, Y., Photoinduced Dehydrogenative Borylation via Dihydrogen Bond Bridged Electron Donor and Acceptor Complexes. *Chem. Eur.J.* **2023,** *29* (9), e202203053.
